# Incidence Trends of Inherited Anemias at the Global, Regional, and National Levels Over Three Decades

**DOI:** 10.1007/s44197-023-00170-9

**Published:** 2023-12-11

**Authors:** Hongwei Tang, Nan Zhang, Xinlei Liu, Hongbo Xiao, Hanyue Zhang, Kang Zhou, Jianchuan Deng

**Affiliations:** 1 Department of Hematology, The Second Affiliated Hospital of Chongqing Medical University, 76 Linjiang Road, Chongqing, 400010 People’s Republic of China; 2 Department of Hematology, Zhongnan Hospital of Wuhan University, Wuhan, 430061 People’s Republic of China

**Keywords:** Inherited anemias, Sickle cell disease, Thalassemias, G6PD deficiency, Global Burden of Diseases

## Abstract

Inherited anemia continues to pose a significant public health concern on a global scale, owing to its extensive geographical prevalence, substantial patient population, and profound ramifications. Here, we investigated detailed information on inherited anemias (including thalassemias, thalassemias trait, sickle cell disease, sickle cell trait, G6PD deficiency, and G6PD trait) for the period 1990–2019 from the Global Burden of Disease study. Over the course of three decades, there has been a persistent rise in the incidence of inherited anemias worldwide, culminating in a total of 44,896,026 incident cases in 2019. However, the prevalence of inherited anemias has exhibited a consistent downward trend over successive years. Significantly, these inherited anemias primarily impact females, exhibiting a male-to-female ratio of 1:1.88. Among males, the most prevalent inherited anemia is G6PD deficiency, whereas G6PD trait prevails among females. The incidence rates of inherited anemias and their temporal trend exhibited significant variations across different regions, with Central Sub-Saharan Africa displaying the highest incidence rates and Central Latin America experiencing the most substantial decline. The findings of this study suggest a significant correlation between the Socio-Demographic index (SDI) and incidence rates of inherited anemias, particularly in regions with lower SDI levels such as Africa and South Asia. These results contribute valuable insights for the analysis of global trends in the burden of inherited anemias.

**Supplementary Information:**

The online version contains supplementary material available at 10.1007/s44197-023-00170-9.

## Introduction

Anemia is a prevalent global phenomenon, particularly prevalent in underdeveloped and developing countries [1]. Iron deficiency anemia is recognized as the most prevalent type of anemia, resulting from insufficient dietary iron intake, impaired iron absorption, and chronic blood loss, affecting a staggering 1.6 billion individuals, constituting approximately 24.8% of the global population [2, 3]. In addition to iron deficiency anemia, anemia stemming from diverse genetic disorders must not be disregarded [4, 5]. Inherited anemias can be categorized into various subtypes, including thalassemias, sickle cell disease, and glucose-6-phosphate dehydrogenase (G6PD) deficiency, due to distinct etiological mechanisms [6]. Despite this, the implementation of preventive and control strategies for inherited anemias has not been widely adopted globally, resulting in an alarming annual occurrence of over 300,000 stillbirths of children with severe anemia worldwide [7]. Most patients in areas with poor healthcare resources do not have access to effective care, and the ongoing global COVID-19 pandemic has undoubtedly exacerbated inequalities in healthcare resources [8]. Therefore, a comprehensive and accurate understanding of the scale and trends of inherited anemia through evidence-based epidemiological studies is necessary to help improve prevention and control strategies in different countries.

Clinically, anemia is usually classified according to etiology and cytology, among which inherited anemias mainly belong to hemolytic anemia, and the disease is closely related to geographical distribution [9, 10]. Thalassemias, characterized by deficiencies in the production of hemoglobin α-globin and β-globin polypeptides, are predominantly endemic in tropical and subtropical regions. The prevalence of thalassemias often coincides with that of malaria due to the historical invasion of human red blood cells by the plasmodium parasite [11, 12]. Sickle cell disease is caused by an abnormal structure of a globulin peptide chain, most of which is found in sub-Saharan Africa [13]. Moreover, carriers of sickle cell are also risk factors for clinical complications such as chronic kidney disease and venous thromboembolism [14]. G6PD deficiency, a prevalent human enzyme disorder, arises from an inherited mutation in an X-linked gene [15]. The investigation, through genetic variation screening, revealed that the highest occurrence of this condition among males was observed in Africans, followed by Asians, Middle Easterners, and Europeans [16]. Carriers of G6PD deficiency are mostly asymptomatic but are also at great risk of acute hemolytic anemia after exposure to some drugs or infection [17]. Despite the considerable advancements in gene therapy and hematopoietic stem cell transplantation, prevention remains the primary approach for mitigating inherited anemias.

The Global Burden of Disease (GBD) project, providing the best possible comparable estimates of ill health, injury, and risk factors, is an important achievement of the long-term collaboration between governments around the world [18]. A key benefit of this resource is that it provides insights into the epidemiological dynamics of thalassemias, sickle cell disease, and G6PD deficiency. In this study, we extracted epidemiological data on the incidence of inherited anemias based on subtype, sex, and regions from GBD. The disease burden of inherited anemias was further assessed by determining time trends of inherited anemias generated by specific aetiologies at global, regional, and country levels between 1990 and 2019. The findings of this research hold significant value as they expand upon prior investigations and offer valuable insights for the formulation of preventive strategies targeting inherited anemias in diverse countries.

## Methods

### Overview

The GBD provides comprehensive assessments of incidence, prevalence, and mortality for 204 countries and territories each year, enclosing comprehensive updates of descriptive as well. The input data of GBD from various sources are inclusive but not limited to censuses, household surveys, civil registration, and statistics and disease registries. And the detailed processing of the dataset was seen in the GBD 2019 which covered data collection, refinement statistics, and final generation [2, 19]. The utilization of DisMod-MR 2.1, a Bayesian meta-regression modeling tool, facilitates the assessment of data agreement and reliability through cross-validation analysis. This tool capitalizes on the annual data update, effective data integration, and advancements in methodology within the GBD dataset, enabling convenient access to epidemiological levels and trends of diseases. More detailed information about those data is provided at the Global Health Data Exchange (GHDx) query tool (https://vizhub.healthdata.org/gbd-results).

### Data Collection

Data on incident cases and incidence rates (per 100,000 population) of inherited anemias were collected from 204 countries spanning the period from 1990 to 2019. The data was analyzed based on gender, region, and subtype. All 204 countries and territories were classified into 21 GBD regions based on geographical contiguity, as outlined in Table 1. The inherited anemias subtypes examined in this study encompassed thalassemias, thalassemias trait, sickle cell disease, sickle cell trait, G6PD deficiency, and G6PD trait. Moreover, to investigate the association between regional and national degrees of socio-development status and inherited anemias incidence, the Socio-Demographic Index (SDI) was utilized.

**Table 1:** The incident cases and incidence rates of inherited anemias in 1990 and 2019, along with their temporal trend

Characteristics	1990		2019		1990–2019
	Incident cases No. × 10^3^ (95% UI)	Incidence rates per 100,000 (95% UI)	Incident cases No. × 10^3^ (95% UI)	Incidence rates per 100,000 (95% UI)	EAPC (95%CI)
Global	41,440.09 (39,375.43 to 43,536.78)	774.6 (736.01 to 813.79)	44,896.03 (42,412.19 to 47,356.9)	580.24 (548.14 to 612.05)	− 0.99 (− 1.06 to − 0.91)
Sex					
Male	14,082.89 (13,157.29 to 15,089.04)	522.8 (488.44 to 560.15)	15,608.25 (14,465.09 to 16,780.64)	402.17 (372.72 to 432.38)	− 0.89 (− 0.94 to − 0.84)
Female	27,357.2 (26,224.46 to 28,457.5)	1029.98 (987.33 to 1071.4)	29,287.77 (27,944.5 to 30,595.42)	759.44 (724.61 to 793.35)	− 1.05 (− 1.15 to − 0.95)
Region					
Andean Latin America	187.28 (168.42 to 207)	490.56 (441.16 to 542.21)	232.29 (205.72 to 261.22)	365.26 (323.47 to 410.75)	− 1.33 (− 1.45 to − 1.22)
Australasia	27.15 (24.59 to 29.81)	133.89 (121.26 to 147.02)	24.3 (21.56 to 27.06)	83.62 (74.18 to 93.1)	− 1.47 (− 1.7 to − 1.24)
Caribbean	257.02 (228 to 289.03)	728.65 (646.38 to 819.38)	250.12 (222.49 to 281.15)	530.29 (471.7 to 596.08)	− 1.06 (− 1.15 to − 0.97)
Central Asia	279.45 (251.95 to 308.37)	403.44 (363.75 to 445.2)	270.09 (243.96 to 297.87)	288.77 (260.83 to 318.48)	− 0.46 (− 0.8 to − 0.12)
Central Europe	241.05 (221.32 to 262.34)	196.03 (179.99 to 213.34)	160.05 (146.82 to 174.19)	140.12 (128.53 to 152.5)	− 0.91 (− 1.15 to − 0.66)
Central Latin America	925.1 (867.7 to 987.09)	563.66 (528.69 to 601.43)	667.07 (618.5 to 719.4)	266.81 (247.38 to 287.74)	− 2.6 (− 2.75 to − 2.46)
Central Sub-Saharan Africa	1503.83 (1368.46 to 1641.56)	2708.64 (2464.82 to 2956.71)	2559.22 (2333.62 to 2794.75)	1945.52 (1774.02 to 2124.57)	− 1.06 (− 1.22 to − 0.9)
East Asia	6352.57 (5927.25 to 6921.31)	518.52 (483.8 to 564.94)	4129.75 (3890.82 to 4448.2)	280.52 (264.29 to 302.15)	− 2.1 (− 2.41 to − 1.79)
Eastern Europe	364.18 (348.85 to 383.04)	160.78 (154.01 to 169.11)	290.06 (278.06 to 304.55)	138.15 (132.43 to 145.05)	0.88 (0.4 to 1.37)
Eastern Sub-Saharan Africa	3597.16 (3367.81 to 3838.38)	1891.58 (1770.98 to 2018.43)	5268.18 (4894.93 to 5654.12)	1279.38 (1188.73 to 1373.1)	− 1.36 (− 1.5 to − 1.21)
High-income Asia Pacific	140.25 (130.88 to 149.63)	80.83 (75.43 to 86.24)	97.6 (91.16 to 104.71)	52.11 (48.67 to 55.91)	− 1.57 (− 1.76 to − 1.38)
High-income North America	1367.59 (1302.87 to 1442.81)	486.82 (463.78 to 513.59)	837.61 (815.22 to 858.82)	229.76 (223.62 to 235.58)	− 1.84 (− 2.25 to − 1.43)
North Africa and Middle East	4082.89 (3849.23 to 4333.1)	1183.35 (1115.63 to 1255.87)	4086.66 (3778.34 to 4421.27)	671.36 (620.71 to 726.33)	− 1.83 (− 1.88 to − 1.78)
Oceania	57.08 (52.46 to 61.71)	882.29 (810.82 to 953.88)	117.14 (105.27 to 129.69)	882.31 (792.9 to 976.82)	− 0.06 (− 0.2 to 0.08)
South Asia	11,702.94 (11,017.99 to 12,471.47)	1066.21 (1003.8 to 1136.22)	12,939.35 (12,239.19 to 13,695.92)	716.78 (678 to 758.69)	− 1.71 (− 1.99 to − 1.42)
Southeast Asia	3430.66 (3242.47 to 3620.48)	734.95 (694.63 to 775.61)	2622.51 (2463.79 to 2787.09)	389.22 (365.67 to 413.65)	− 1.99 (− 2.11 to − 1.86)
Southern Latin America	104.1 (91.41 to 117.75)	210.12 (184.51 to 237.67)	92.72 (81.71 to 105.14)	138.9 (122.4 to 157.51)	− 1.19 (− 1.36 to − 1.01)
Southern Sub-Saharan Africa	451.62 (429.3 to 476.06)	860.35 (817.82 to 906.9)	473.39 (449.31 to 499.35)	602.47 (571.82 to 635.5)	− 1.03 (− 1.14 to − 0.93)
Tropical Latin America	612 (596.87 to 626.72)	400.31 (390.42 to 409.94)	596.03 (581.53 to 609.68)	266.56 (260.08 to 272.67)	− 0.94 (− 1.19 to − 0.7)
Western Europe	945.7 (887.52 to 1004.3)	245.9 (230.77 to 261.13)	807.33 (752.81 to 863.51)	185.04 (172.54 to 197.91)	− 0.79 (− 0.88 to − 0.69)
Western Sub-Saharan Africa	4810.47 (4540.76 to 5094.16)	2497.9 (2357.85 to 2645.21)	8374.55 (7828.37 to 8951.13)	1835.26 (1715.57 to 1961.62)	− 0.97 (− 1.07 to − 0.86)

*EAPC* estimated annual percentage change, *UI* uncertainty interval, *CI* confidence interval

### SDI Definition and Calculation

SDI is a summary indicator used to represent the social and economic conditions that can impact health outcomes in a given location [20]. It comprises three indices: lag-distributed income (LDI) per capita, mean education for those aged 15 years or older (EDU15+), and total fertility rate for those younger than 25 years of age (TFU25). Each index ranges from 0 to 1, indicating the thresholds beyond which health outcomes no longer worsen or improve. A location with an SDI of 0 indicates a theoretical minimum level of development status relevant to health outcomes, while a location with an SDI of 1 indicates a theoretical maximum level.

### Statistical Analysis

We used incidence rates and estimated annual percentage change (EAPC) as the quantification metrics of inherited anemia incidence trends [21]. Among these, the EAPC serves as a valuable instrument for assessing the occurrence rates during designated time intervals, effectively quantifying alterations in the trends of disease incidence over time, and facilitating comparability across various subcategories of hereditary anemias. By fitting a regression line to the natural logarithm of rates using the calendar year as a regressor variable, we get each EAPC value and its 95% confidence interval (CI) of the identified trends. The equation is rendered thus:

$$\begin{aligned} & {\text{y}} = {{\upalpha }} + {{\upbeta }}{\text{x}} + {{\upvarepsilon }} \\ & {\text{EAPC}} = 100{{\% }} \times (e^\beta - 1) \\ \end{aligned}$$

where y = ln(incidence rates), and x = calendar year.

The presence of both the EAPC value and the lower limit value of the 95% confidence interval being greater than 0 signifies an upward trend in incidence rates. Conversely, if the EAPC value and the upper limit value of the 95% confidence interval are both less than 0, it indicates a downward trend in incidence rates. If the 95% confidence interval of the resulting value encompasses 0, it suggests that there have been no significant changes in incidence rates over time. All calculations, statistical tests, and visual representations were performed using R software (version 3.5.2) and GraphPad Prism (version 8.02). Statistical significance is defined as *p* values below 0.05.

## Results

### Global Burden of Inherited Anemias

**Fig. 1:**
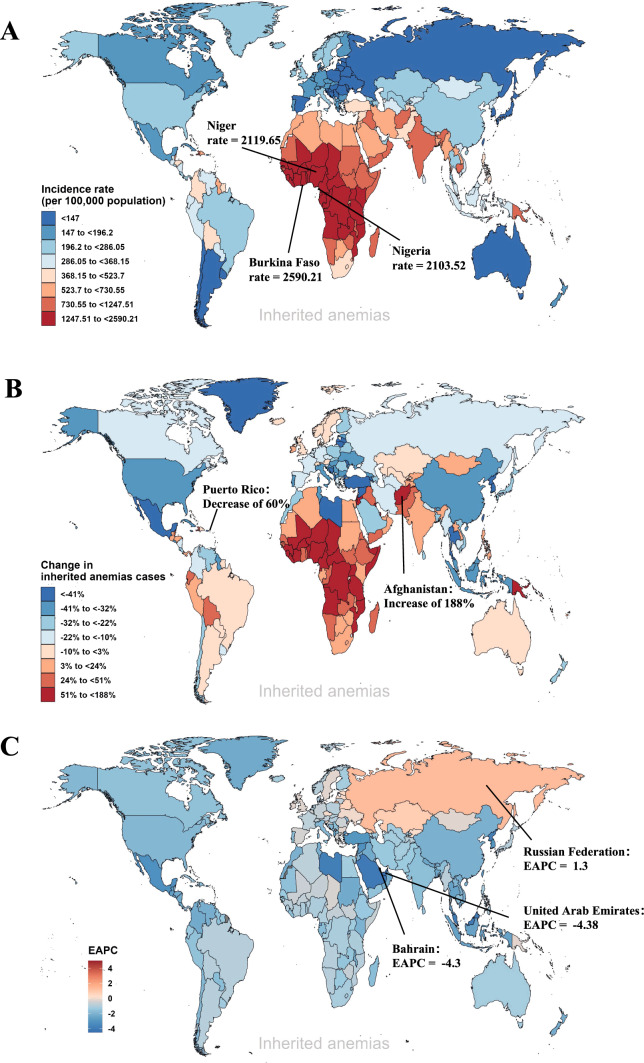
The incidence of inherited anemias across various countries and territories, encompassing both genders. **A** The incidence rate of inherited anemias in 2019. **B** The percentage change in incident cases of inherited anemias between 1990 and 2019. **C** The EAPCs in incidence rate of inherited anemias from 1990 to 2019. EAPCs, estimated annual percentage change

The occurrence of inherited anemias exhibited an irregular distribution, displaying significant variations across different countries and regions. Based on statistical data, the global incident cases of inherited anemias increased by 8.34% from 41,440,085 cases in 1990 to 44,896,026 cases in 2019. The most marked increase was observed in Afghanistan (percent change = 188%), Qatar (percent change = 158%) and Niger (percent change = 153%), with additional details by country given in Fig. 1B. In relation to the incidence rates per 100,000 population, the global prevalence of inherited anemias in 2019 was recorded at 580.24, exhibiting a notable reduction of approximately 25% when compared to the incidence rates observed in 1990. When arranged in descending order based on their respective incidence rates, the three countries occupying the highest ranks are Burkina Faso (2590.21 in 2019), Niger (2119.65 in 2019), and Nigeria (2103.52 in 2019), respectively (Fig. 1A). On a global scale, the majority of nations exhibited a declining pattern, wherein an average annual decrease of 0.99% was observed worldwide. Among those countries, the decreasing trend is particularly striking in the United Arab Emirates (EAPC = − 4.38), followed by Bahrain (EAPC = − 4.3) and Saudi Arabia (EAPC = − 4.05) (Fig. 1C). Besides, only 10 out of 204 countries found an upward trend of inherited anemia incidence rates from 1990 to 2019 (Table S7). According to the results of the hierarchical cluster analysis, the incidence rate trends of inherited anemias in 204 countries or regions were categorized into four groups including stable or low decrease, minor decrease, middle decrease, and significant increase, as shown in Figure S1.

**Fig. 2:**
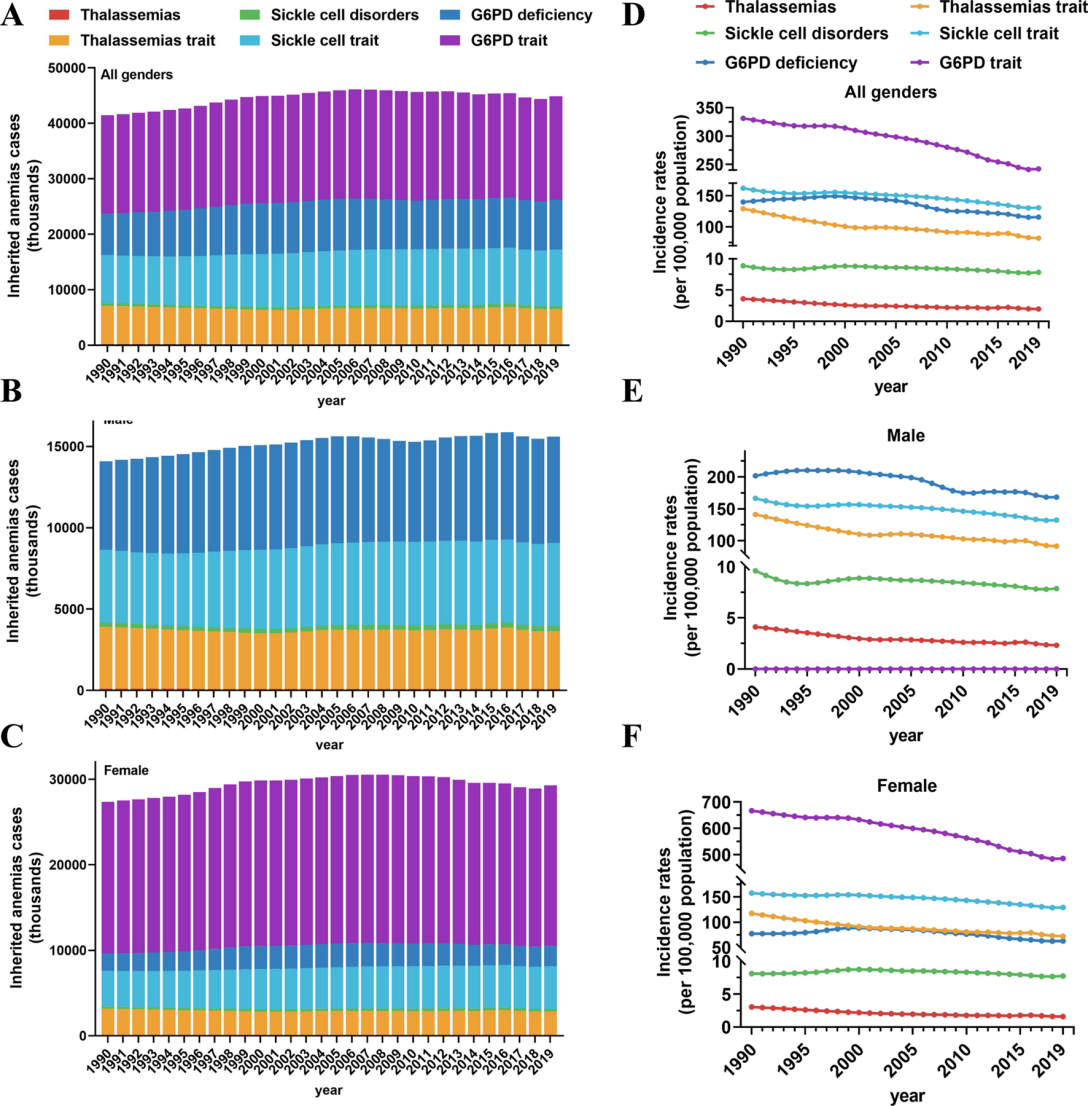
The number and incidence rate of inherited anemias by six causes from 1990 to 2019, broken down by gender. **A** Global inherited anemias incident cases for all genders combined. **B** The global incidence of male inherited anemias. **C** The global incidence of female inherited anemias. **D** The incidence rates of inherited anemias for all genders. **E** The incidence rates of inherited anemias for males. **F** The incidence rates of inherited anemias for females

**Fig. 3:**
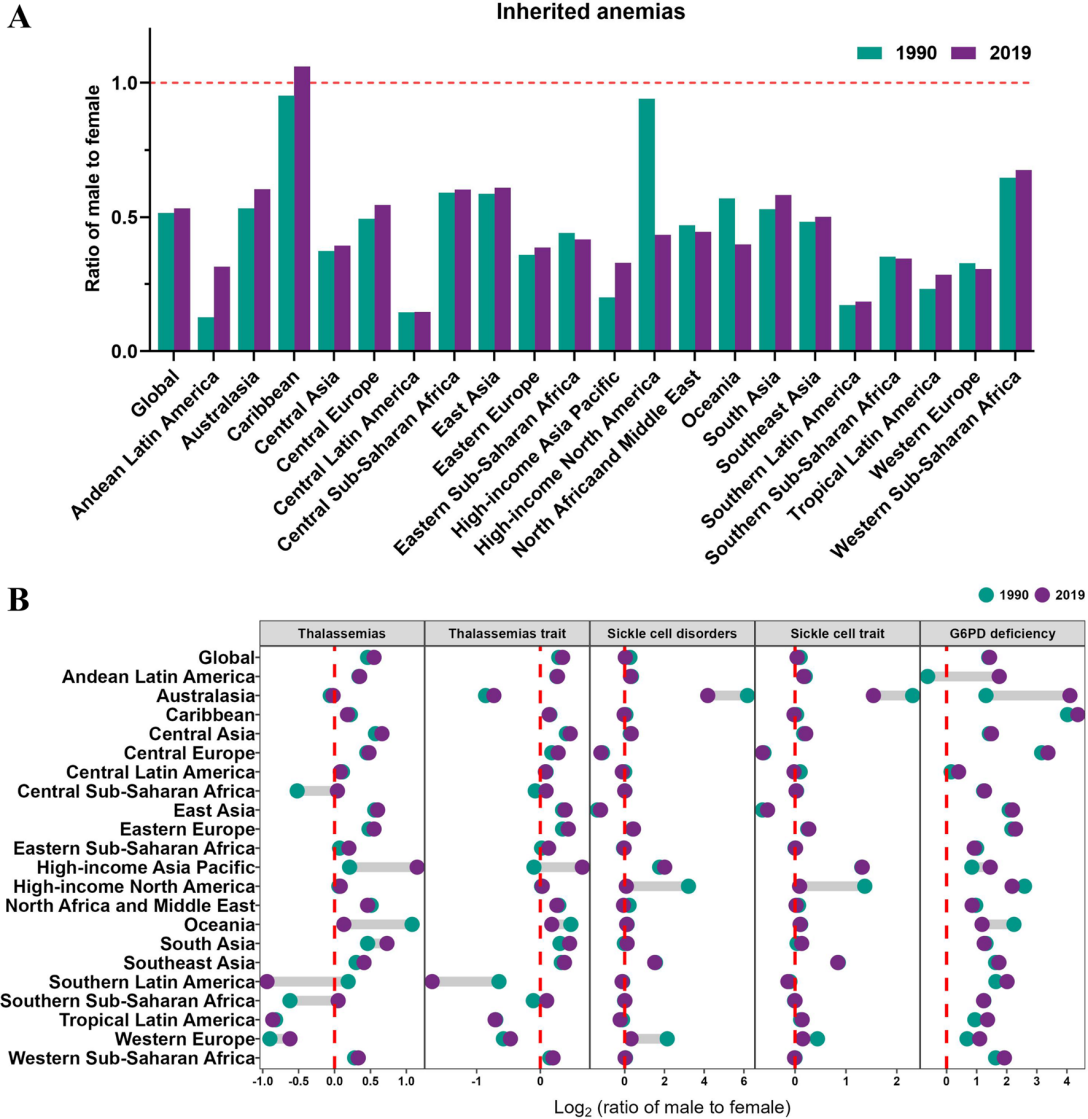
The global incidence of inherited anemias stratified by gender from 1990 to 2019. **A** The sex ratio of inherited anemias cases by region. **B** The logarithm of the male-to-female sex ratio among five causes of inherited anemias. Since G6PD deficiency is inherited in an X-linked sex chromosome pattern, sex differences in G6PD traits are not discussed

The results of our study indicate notable gender disparities in the prevalence of six subtypes of inherited anemias, with a significantly higher incidence observed among females (27,357,195 cases) compared to males (14,082,890 cases) in 1990. Encouragingly, our findings also suggest a gradual reduction in this gender gap over a span of three decades, with a steeper decline in the disease prevalence among females (EAPC = − 1.05) compared to males (EAPC = − 0.89) (Table 1). Overall, the pooled data from six subtypes (including thalassemias, thalassemias trait, sickle cell disease, sickle cell trait, G6PD deficiency, and G6PD trait) showed the number of incident cases (18,731,778 cases) and incidence rates (242.09 in 2019) of G6PD trait remained the highest among different subtypes of inherited anemias till 2019 (Fig. 2A, D, Table S6). Furthermore, of females with inherited anemias, the G6PD trait alone accounted for the vast majority. By contrast, G6PD deficiency, sickle cell trait, and thalassemia trait were more common in males (Fig. 2B, C). The trend lines of the six subtypes of inherited anemias showed a general decrease, with the most pronounced decrease of thalassemias both in men (EAPC = − 1.74)and women (EAPC = − 2.11). Besides, since G6PD deficiency is inherited in an X-linked sex chromosome pattern, all the patients were female (Fig. 2E–F, Table S6). In terms of geographical regions, the global sex ratio (male-to-female) of inherited anemias exhibited fluctuations around 0.5, with values of 0.51 in 1990 and 0.53 in 2019. The majority of regions maintained a stable or slightly increasing trend, with the Caribbean region displaying the highest male-to-female ratio of 1.06 in 2019, making it the sole region with a surplus of males. Additionally, six out of the 21 regions experienced a decline in ratio from 1990 to 2019, with the most significant decrease observed in High-income North America, dropping from 0.94 in 1990 to 0.43 in 2019 (Fig. 3A). Significant regional disparities were evident in the sex ratio. In the year 2019, the prevalence of thalassemias, thalassemias trait, sickle cell disease, sickle cell trait, and G6PD deficiency exhibited the highest sex ratios in the High-income Asia Pacific, High-income Asia Pacific, Australasia, Australasia, and the Caribbean regions, respectively (Fig. 3B).

**Fig. 4:**
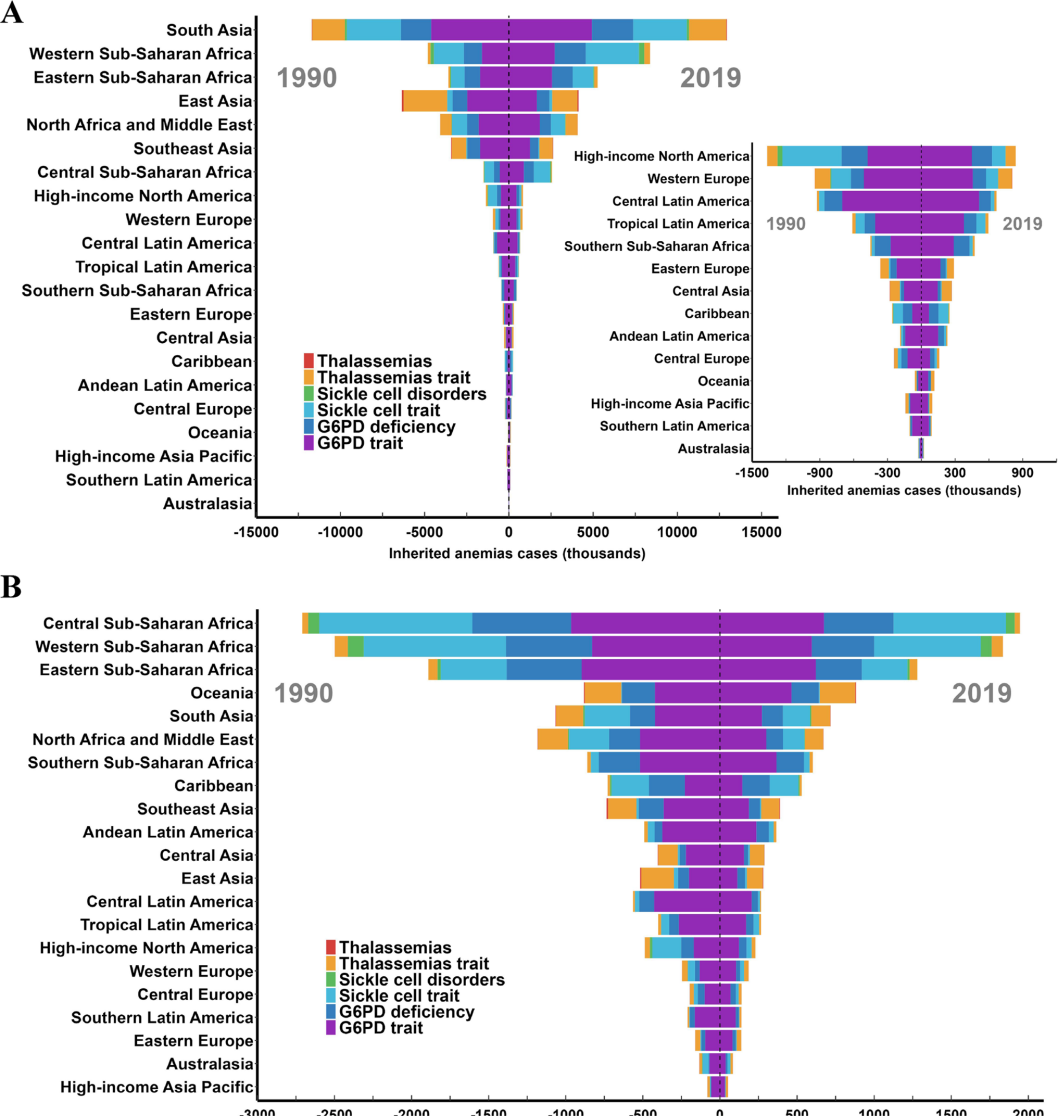
The global incidence of inherited anemias by six causes and regions for both sexes combined. **A** Inherited anemias incident cases by six causes and regions between 1990 and 2019. **B** The incidence rates (per 100 000 population) of inherited anemias at a regional level in 1990 and 2019. For each group, the left column shows case data in 1990 and the right column shows data from 2019. Certain regions are magnified to the right of the panel. The order is based on the incidence of inherited anemias in 2019

**Fig. 5:**
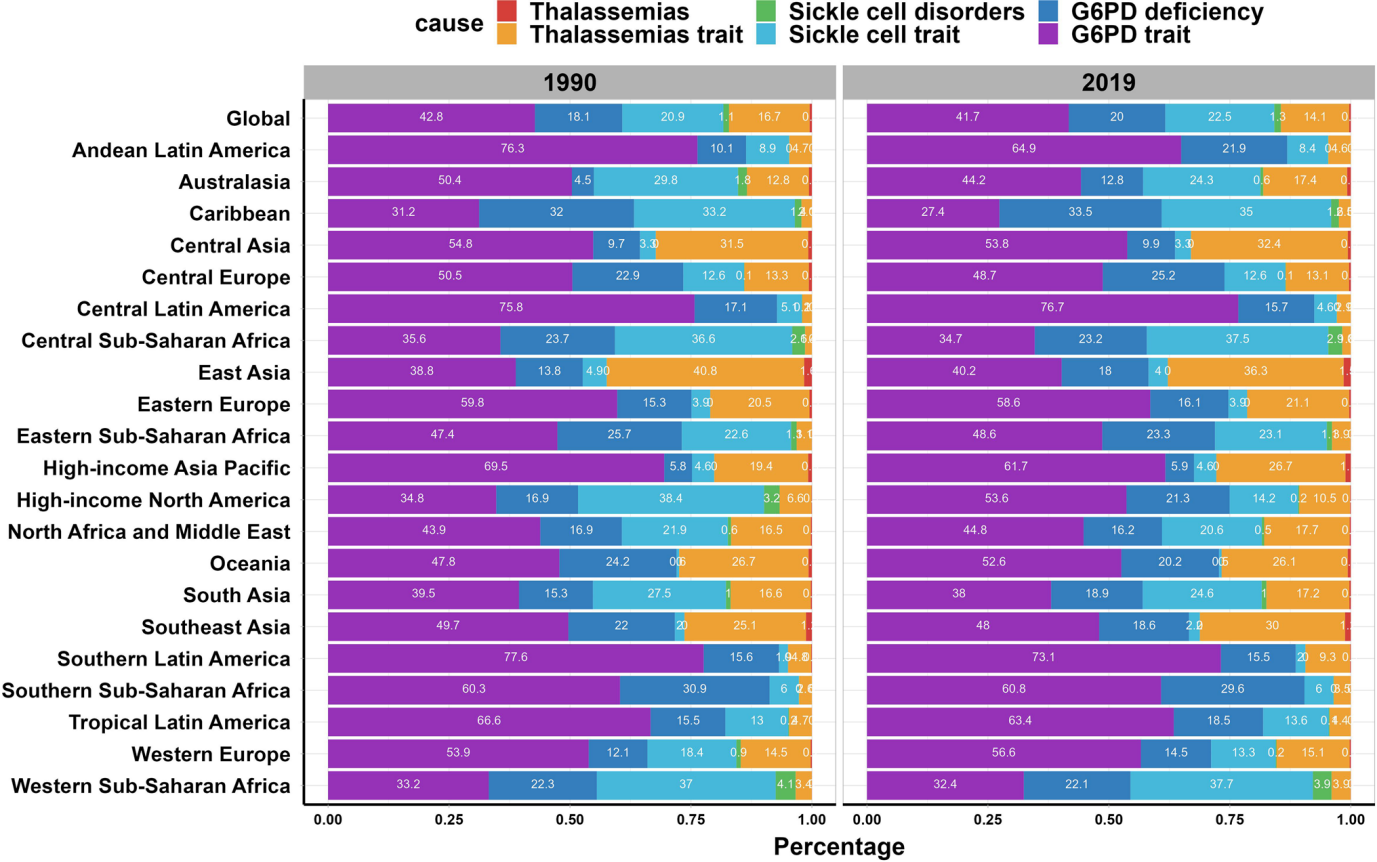
Contribution of specific causes to inherited anemias incident cases, by regions for both sexes combined in 1990 and 2019

**Fig. 6:**
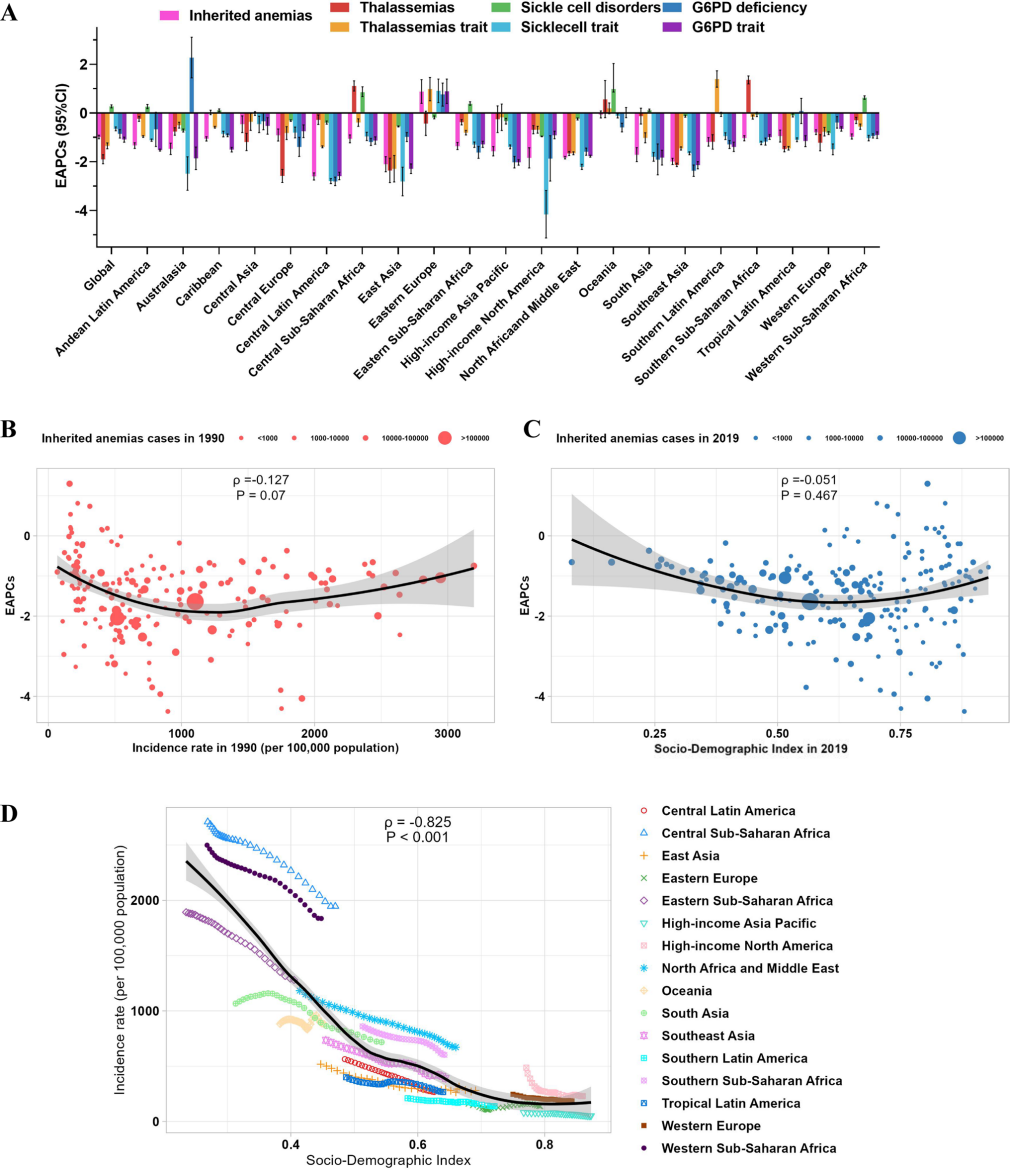
The EAPCs of inherited anemias at global, regional, and national levels. **A** The EAPCs of inherited anemias from 1990 to 2019, both sexes, by region, and by six causes. **B** The correlation between EAPCs and inherited anemias incidence rates in 1990. **C** The correlation between EAPCs and SDI in 2019. **D** The incidence rates of inherited anemias by region from 1990 to 2019 based on SDI levels. EAPCs, estimated annual percentage change. G6PD, glucose-6-phosphate dehydrogenase. SDI, Socio-Demographic Index

For different regions, we drew the graphs to highlight the significant differences over the regions distributed by geographical location. Among the 21 GBD regions, high incident cases were observed in most African and Asian regions such as South Asia and Western Sub-Saharan Africa. In terms of incidence rates, it was observed that African regions, specifically Central Sub-Saharan Africa, Western Sub-Saharan Africa, and Eastern Sub-Saharan Africa, exhibited significantly higher incidence rates compared to the remaining 18 regions (Fig. 4). The proportions of different subtypes of inherited anemias at the global and regional levels in 1990 and 2019 are shown in Fig. 5. Globally, the G6PD trait, G6PD deficiency and sickle cell trait were the most common subtypes of inherited anemias, which accounted for more than 80% of inherited anemias incident cases, followed by thalassemias trait and sickle cell disorder. While the proportions of these subtypes remained relatively stable in most regions according to the Global Burden of Disease (GBD) data, significant changes were observed in certain regions. For example, in the context of Andean Latin America, there has been a notable decline in the percentage of G6PD trait subtypes associated with inherited anemias, decreasing from 76.3% in 1990 to 64.9% in 2019. Conversely, the proportion of G6PD deficiency has shown an increase, rising from 10.1% in 1990 to 21.9% in 2019 (Fig. 5). In terms of incidence rates, there has been a consistent global decline in the occurrence of inherited anemias (EAPC = − 0.99), although the extent of this decline has varied across different regions. Notably, Eastern Europe stands out as the only region among the 21 regions studied that has experienced an upward trend in incidence rates, with an EAPC value of 0.88 (Fig. 6A, Table 1).

### Anemias Caused by the Abnormal Number of the Globin Peptide Chain: Thalassemias

Globally, thalassemia comprised only 0.4% of the total incident cases of inherited anemias in 2019. The highest number of thalassemia cases occurred in East Asia (60,561 cases), yet only accounted for 1.5% of the total region cases. None of the 21 GBD regions had a proportion exceeding 2% in any district (Fig. 5 and Table S1). Besides, the largest increase in the absolute number of thalassemia cases was seen in Somalia (percent change = 4.84%), followed by Angola and Mozambique (Figure S3). In terms of countries, relatively higher incidence rates were observed in Cambodia (20.63 in 2019), Lao People’s Democratic (20.21 in 2019), and Thailand (11.08 in 2019) (Figure S2). From 1990 to 2019, there was a discernible decline in the incidence rates of thalassemia, as indicated by the global EAPC value of − 1.9. Among the countries analyzed, South Sudan exhibited the most significant increase in incidence rates (EAPC = 2.17), followed by Somalia and Burundi (Figure S4 and Table S7).

In 2019, the prevalence of thalassemias trait accounted for 14.1% of inherited anemias, exhibiting a decrease compared to the calculated proportion of 16.7% in 1990. Notably, various regions in Asia, such as Central Asia, East Asia, and Southeast Asia, displayed proportions exceeding 30%. However, it is noteworthy that the thalassemias trait proportions within each region remained relatively stable on a global scale (Fig. 5). The most obvious increase in absolute number was found in Somalia (percent change = 2.61%), Afghanistan (percent change = 1.97%), and Chad (percent change = 1.76%) (Figure S6 and Table S7). According to the GBD regions, the highest incidence rates of the thalassemias trait were observed in Oceania (230.09 in 2019), followed by South Asia (122.94 in 2019) and North Africa and the Middle East (118.56 in 2019). And the most pronounced increase in incidence rates of the thalassemias trait was seen in Eastern Europe (EAPC = 0.92), also the only region which followed a growth trend of incidence rates. (Figure S5 and Table S2). At the national levels, incidence rates of the thalassemias trait in most countries stayed in a downward trend except in 23 countries including Argentina (EAPC = 1.95), Uruguay (EAPC = 1.61) and Russian Federation (EAPC = 1.38) (Figure S7 and Table S7).

### Anemias Caused by the Abnormal Structure of the Globin Peptide Chain: Sickle Cell Disorders

Sickle cell disorders accounted for a mere 1.3% of the overall disease burden of inherited anemias worldwide, with no single region among the 21 GBD regions surpassing 4%. Despite a 27.44% increase in incident cases in 2019 compared to the absolute number in 1990, the incidence rates of sickle cell disorders remained relatively constant, as indicated by the global EAPC value of − 0.31 (Fig. 5 and Table S3). Over the decades, the most remarkable increase in the absolute number of sickle cell disorders could be detected in Afghanistan (percent change = 1.85%) among the 204 countries. On the contrary, Italy (percent change = − 0.98%) and the United States of America (percent change = − 0.96%) found a striking decrease in incident cases of sickle cell disorders (Figure S9, Table S7). Turning to incidence rates, Western Sub-Saharan Africa (70.75 in 2019) and Central Sub-Saharan Africa (55.83 in 2019) showed substantially higher incidence rates than any other GBD regions in 2019 (Figure S8 and Table S3). However, we observed a universal decrease in incidence rates of sickle cell disorders yearly, with the greatest change in High-income North America (EAPC = − 8.16), followed by Australasia (EAPC = − 7) and Western Europe (EAPC = − 5.04). For the country level, The most negative EAPC was observed in Saudi Arabia (EAPC = − 9.5) (Table S3 and Figure S10).

The burden of sickle cell trait accounted for 22.5% of the total global burden of inherited anemias in 2019. With the exception of High-income North America, which experienced a significant change from 38.4% in 1990 to 14.2% in 2019, the majority of GBD regions exhibited either stability or minor changes over time (Fig. 5). Generally, incident cases of sickle cell trait increased by 17% from 8,662,499 cases in 1990 to 10,113,130 cases in 2019. The most prominent case increases were found in Afghanistan, Somalia, and Qatar, with the percentage being 1.84%, 1.62%, and 1.54%, respectively (Figure S12 and Table S4). Considering the variations in incidence rates in each region, we drew graphs with different colors to mark the differences. Significantly higher incidence rates were observed in Central Sub-Saharan Africa (729.86 in 2019) and Western Sub-Saharan Africa (692.02 in 2019) compared to numerous other regions. Among countries, the variations in incidence rates ranged up to a maximum of 100-fold, with Sierra Leone exhibiting the highest rate (7863.46 in 2019) and Palau displaying the lowest rate (1.3 in 2019) (Figure S11, Table S4, and Table S7). In terms of incident trends, the Russian Federation exhibited the most notable rise in incidence rates of the sickle cell trait (EAPC = 1.32), while Saudi Arabia experienced the most substantial decline (EAPC = − 6.16) (Figure S13 and Table S7).

### Anemias Caused by Decreased G6PD Enzyme Activit: G6PD Deficiency

There were a total of 8,956,939 cases G6PD deficiency incident cases in this study, accounting for 20% of the global inherited anemias cases in 2019. The G6PD deficiency proportion oscillated between 10 and 35% in each GBD region (Fig. 5 and Table S5). Furthermore, relatively higher incident cases of G6PD deficiency were observed in males (6,531,655 cases) than in females (2,425,284 cases), and the same applied to the incidence rates (168.3 in males and 62.89 in females). For absolute case number, Bolivia (Plurinational State of) had the maximum increase (percent change = 2.33%) while the maximum reduction in Brunei Darussalam (percent change = − 0.76%) (Figure S15). The incidence rates of G6PD deficiency vary significantly across the GBD regions, with Central Sub-Saharan Africa exhibiting the highest incidence rate (450.87 in 2019) and High-income Asia Pacific displaying the lowest incidence rate (3.08 in 2019) (Figure S14 and Table S5). During 1990–2019, the incidence rates decreased by an average of 0.86%, with negative EAPC in 188 out of 204 countries included. The most significant decrease was found in Malaysia, the United Arab Emirates, and the Republic of Korea, with EAPC values of − 5.29, − 4.88 and − 4.77, respectively (Figure S16 and Table S7).

Among those subtypes of inherited anemias, the G6PD trait presented in the highest proportion of 41.7% in 2019, and all the regions showed relatively high proportions of the G6PD trait over the six subtypes, with the highest percentage of 76.7% in Central Latin America, followed by Southern Latin America (73.1%) and Andean Latin America (64.9%) (Fig. 5). Due to the X-linked incomplete dominant inheritance mode of the G6PD trait, all the patients were female (Fig. 3B and Table S6). The global incident cases of the G6PD trait were 18,731,778 cases in 2019, increasing 5% when compared with the absolute number in 1990, with the highest case number observed in India (3,977,527 cases, 2019). Besides, the increases in the absolute case were prominent in Afghanistan, Niger, Papua New Guinea and Qatar, with the maximum being 1.86% (Figure S18 and Table S7). For GBD regions, some African areas such as Central Sub-Saharan Africa (674.44 in 2019) and Eastern Sub-Saharan Africa (622.06 in 2019) had notably higher incidence rates than others. In terms of countries, some African countries including Burkina Faso, Uganda, and Niger displayed high incidence rates levels (Figure S17 and Table S6). However, varying degrees of decrease in incidence rates were observed in almost all the GBD regions except for Oceania (EAPC = 0.01) and Eastern Europe (EAPC = 0.89). Of these, the Russian Federation showed the highest increase (EAPC = 1.31) over three decades (Figure S19).

### Factors Related to the Incidence

In order to examine the correlation between EAPC values, SDI, and incidence rates, correlation analyses were employed. In a conceptual sense, EAPC values were utilized to measure the average annual change in the incidence rates of inherited anemias, with the objective of quantifying temporal changes in disease rates and projecting future rates. SDI, on the other hand, functioned as a comprehensive indicator of societal development, encompassing factors such as income per capita, average years of schooling, and total fertility rate. No significant correlations were found between EAPC values and incidence rates, and the same is true of EAPC and SDI (Fig. 6B, C). However, a statistically significant negative correlation (ρ =  − 0.825, *p* < 0.001) was observed between the SDI and incidence rates for inherited anemias (Fig. 6D). This finding suggests that public health interventions implemented in regions with higher SDI scores tend to yield more favorable outcomes for these diseases. Notably, all subtypes of inherited anemias, except for thalassemias and thalassemias trait, exhibited strong negative associations with SDI. It is worth noting that while possible correlations between thalassemias and thalassemias trait and SDI may still exist, they may be overshadowed by stronger correlations with geographical distribution (Figure S20–S25).

## Discussion

Our study elaborated and analyzed the global trends of incidence for inherited anemias by gender, region, and country. According to previous studies, inherited anemias embrace a highly heterogeneous group of rare/low-frequency disorders with remarkable incident differences across regions [10, 11, 22–24]. This study encompassed a range of diseases, including thalassemias, thalassemias trait, sickle cell disease, sickle cell trait, G6PD deficiency, and G6PD trait [6, 25]. Over the period from 1990 to 2019, there was a 25% decrease in the incidence rates of inherited anemias, with varying levels of declining trends observed for each type. The initial success can be attributed to the continuous efforts in public health education, widespread premarital and prenatal screening, and global genetic counseling initiatives [26–29].

In relation to the prevalence of inherited anemias worldwide, the year 2019 revealed a heightened probability of occurrences in females, as evidenced by a male-to-female ratio of 1:1.88 (Table 1). This finding underscores the importance of conducting comprehensive subtyping analysis for hereditary anemias. In a noteworthy manner, it was observed that the entirety of the G6PD trait patients in the year 2019 amounted to 18,731,778 cases, all of which were females due to the X-linked inheritance pattern. Conversely, the remaining five subtypes exhibited a nearly equal representation of genders, albeit with notably lower proportions of females in thalassemias and G6PD deficiency. This further underscores the reasons behind the imbalance in disease occurrence between males and females. Moreover, there was a notable disparity in the incidence rates of each inherited anemia type between genders, with males exhibiting the highest incidence rates in G6PD deficiency and females in G6PD trait. The substantial presence of G6PD deficiency and G6PD trait patients had a significant impact on the disease composition and sex ratio of global inherited anemias. Consequently, it is imperative to implement targeted prevention and early detection measures, such as public health education, large-scale screening, diagnosis, and genetic counseling, at an early stage to mitigate disease progression and alleviate the burden associated with these specific conditions. Consistent with the results of previous studies, regional and inter-country disparities in the incidence of inherited anemias at different levels of development were evident [2, 30]. For overall inherited anemias, incident trends are stable or declining to varying degrees in the vast majority of the 204 countries covered in this study, and only 16 of those showed significant increases in recent years. This reflects the efforts made by global health authorities and government departments in addressing these conditions. Moreover, the incidence rates correlated significantly and inversely with SDI levels, with lower SDI countries distributed in Africa and South Asia having heavier burdens. National prevention and control plans have not been established due to financial instability and lack of medical facilities, making it more challenging to narrow down the gap of inherited anemias incidence between countries with different SDI levels [22, 31]. Nonetheless, in areas characterized by a lowe SDI, there exist specific measures that can be undertaken. These measures may encompass the establishment of localized or nationwide public health outreach initiatives, the cultivation of collaborative efforts among consortiums, the provision of directives for standardized data collection and analysis, and the promotion of shared methodologies for dissemination and training. Every country is urged to formulate a tailored strategy that aligns with its specific national conditions [32, 33].

This study distinguishes itself by providing a comprehensive perspective on the burden of inherited anemias through the utilization of up-to-date global national statistics. Nevertheless, it is important to acknowledge the presence of limitations, as is the case with numerous diseases assessed by the GBD framework, wherein the accuracy of the inherited anemias model is contingent upon the quality and quantity of available data [20, 34, 35]. The generalizability of current data on inherited anemia may be compromised by limited sample sizes and cognitive/diagnostic difficulties in regions with scarce cases or resources. The uncertainty surrounding the alignment of data with unified quality standards across regions persists. Nevertheless, our study offers a comprehensive evaluation of the burden and trajectory of inherited anemia over a span of three decades, thereby enhancing awareness and global disease surveillance. Additionally, the findings will contribute to the development of policies pertaining to maternal, newborn, and child health.

## Conclusion

Despite the overall decline in incidence rates, the persistent heavy economic burden and significant health consequences associated with inherited anemias remain a cause for concern. The sex ratio of inherited anemia has been altered due to genetic mechanisms and the initial patient count of specific subtypes, resulting in a pattern of female predominance. In addition, some countries and regions with relatively low levels of SDI located in Africa and South Asia showed a higher disease burden and an increasing incidence trend of inherited anemia.

### Supplementary Information

Below is the link to the electronic supplementary material.

Supplementary file1 (PDF 6196 kb)

## Data Availability

The data are available from the Global Burden of Disease Results Tool of the Global Health Data Exchange (http://ghdx.healthdata.org/). All results generated in this study can be found in the supplementary material.

## Abbreviations

CI: Confidence interval
COVID-19: Corona Virus Disease 2019
EAPC: Estimated annual percentage change
GBD: Global Burden of Disease
GHDx: Global Health Data Exchange
G6PD: Glucose-6-phosphate dehydrogenase
SDI: Socio-Demographic Indices
UI: Uncertainty interval
