# Supplementary material for: Incidence Trends of Inherited Anemias at the Global, Regional, and National Levels Over Three Decades
Source: J Epidemiol Glob Health. 2023 Dec 11;14(1):72–85. doi: 10.1007/s44197-023-00170-9 (PMC11043255; doi:10.1007/s44197-023-00170-9)
Supplement: Supplementary file 1 — Supplementary file1 (PDF 6196 kb) [file 44197_2023_170_MOESM1_ESM.pdf]

Supplementary material

Incidence trends of inherited anemias at the global, regional, and national levels over three decades

Supplementary Figure S1. The hierarchical cluster analysis of the temporal trends in incidence rate of inherited anemias at national level. ········ 1

Supplementary Figure S2. The incidence rate of thalassemias in 2019. ········ 2

Supplementary Figure S3. The percentage change in incident cases of thalassemias between 1990 and 2019. ········ 2

Supplementary Figure S4. The EAPCs in incidence rate of thalassemias from 1990 to 2019. EAPCs, estimated annual percentage change. ········ 3

Supplementary Figure S5. The incidence rate of thalassemias trait in 2019. ········ 3

Supplementary Figure S6. The percentage change in incident cases of thalassemias trait between 1990 and 2019. ········ 4

Supplementary Figure S7. The EAPCs in incidence rate of thalassemias trait from 1990 to 2019. EAPCs, estimated annual percentage change. ········ 4

Supplementary Figure S8. The incidence rate of sickle cell disorders in 2019. ········ 5

Supplementary Figure S9. The percentage change in incident cases of sickle cell disorders between 1990 and 2019. ········ 5

Supplementary Figure S10. The EAPCs in incidence rate of sickle cell disorders from 1990 to 2019. EAPCs, estimated annual percentage change. ········ 6

Supplementary Figure S11. The incidence rate of sickle cell trait in 2019. ········ 6

Supplementary Figure S12. The percentage change in incident cases of sickle cell trait between 1990 and 2019. ········ 7

Supplementary Figure S13. The EAPCs in incidence rate of sickle cell trait from 1990 to 2019. EAPCs, estimated annual percentage change. ········ 7

Supplementary Figure S14. The incidence rate of G6PD deficiency in 2019. G6PD, glucose-6-phosphate dehydrogenase. ········ 8

Supplementary Figure S15. The percentage change in incident cases of G6PD deficiency between 1990 and 2019. G6PD, glucose-6-phosphate dehydrogenase. ········ 8

Supplementary Figure S16. The EAPCs in incidence rate of G6PD deficiency from 1990 to 2019. EAPCs, estimated annual percentage change. G6PD, glucose-6-phosphate dehydrogenase. ········ 9

Supplementary Figure S17. The incidence rate of G6PD trait in 2019. G6PD, glucose-6-phosphate dehydrogenase. 9

Supplementary Figure S18. The percentage change in incident cases of G6PD trait between 1990 and 2019. G6PD, glucose-6-phosphate dehydrogenase. 10

Supplementary Figure S19. The EAPCs in incidence rate of G6PD trait from 1990 to 2019. EAPCs, estimated annual percentage change. G6PD, glucose-6-phosphate dehydrogenase. 10

Supplementary Figure S20. The EAPCs in incidence rate of thalassemias from 1990 to 2019, at global, regional and national level for both sexes combined. 11

Supplementary Figure S21. The EAPCs in incidence rate of thalassemias trait from 1990 to 2019, at global, regional and national level for both sexes combined. 12

Supplementary Figure S22. The EAPCs in incidence rate of sickle cell disorders from 1990 to 2019, at global, regional and national level for both sexes combined. 13

Supplementary Figure S23. The EAPCs in incidence rate of sickle cell trait from 1990 to 2019, at global, regional and national level for both sexes combined. 14

Supplementary Figure S24. The EAPCs in incidence rate of G6PD deficiency from 1990 to 2019, at global, regional and national level for both sexes combined. 15

Supplementary Figure S25. The EAPCs in incidence rate of G6PD trait from 1990 to 2019, at global, regional and national level for both sexes combined. 16

Supplementary Table S1. The incident cases and incidence rates of thalassemias in 1990 and 2019, along with their temporal trend. 17

Supplementary Table S2. The incident cases and incidence rates of thalassemias trait in 1990 and 2019, along with their temporal trend. 19

Supplementary Table S3. The incident cases and incidence rates of sickle cell disorders in 1990 and 2019, along with their temporal trend. 21

Supplementary Table S4. The incident cases and incidence rates of sickle cell trait in 1990 and 2019, along with their temporal trend. 23

Supplementary Table S5. The incident cases and incidence rates of G6PD deficiency in 1990 and 2019, along with their temporal trend. 25

Supplementary Table S6. The incident cases and incidence rates of G6PD trait in 1990 and 2019, along with their temporal trend. 27

Supplementary Table S7. The change of inherited anemias cases and incidence rates between 1990 and 2019 at national level, both sexes. 29

Supplementary Figure S1. The hierarchical cluster analysis of the temporal trends in incidence rate of inherited anemias at national level.

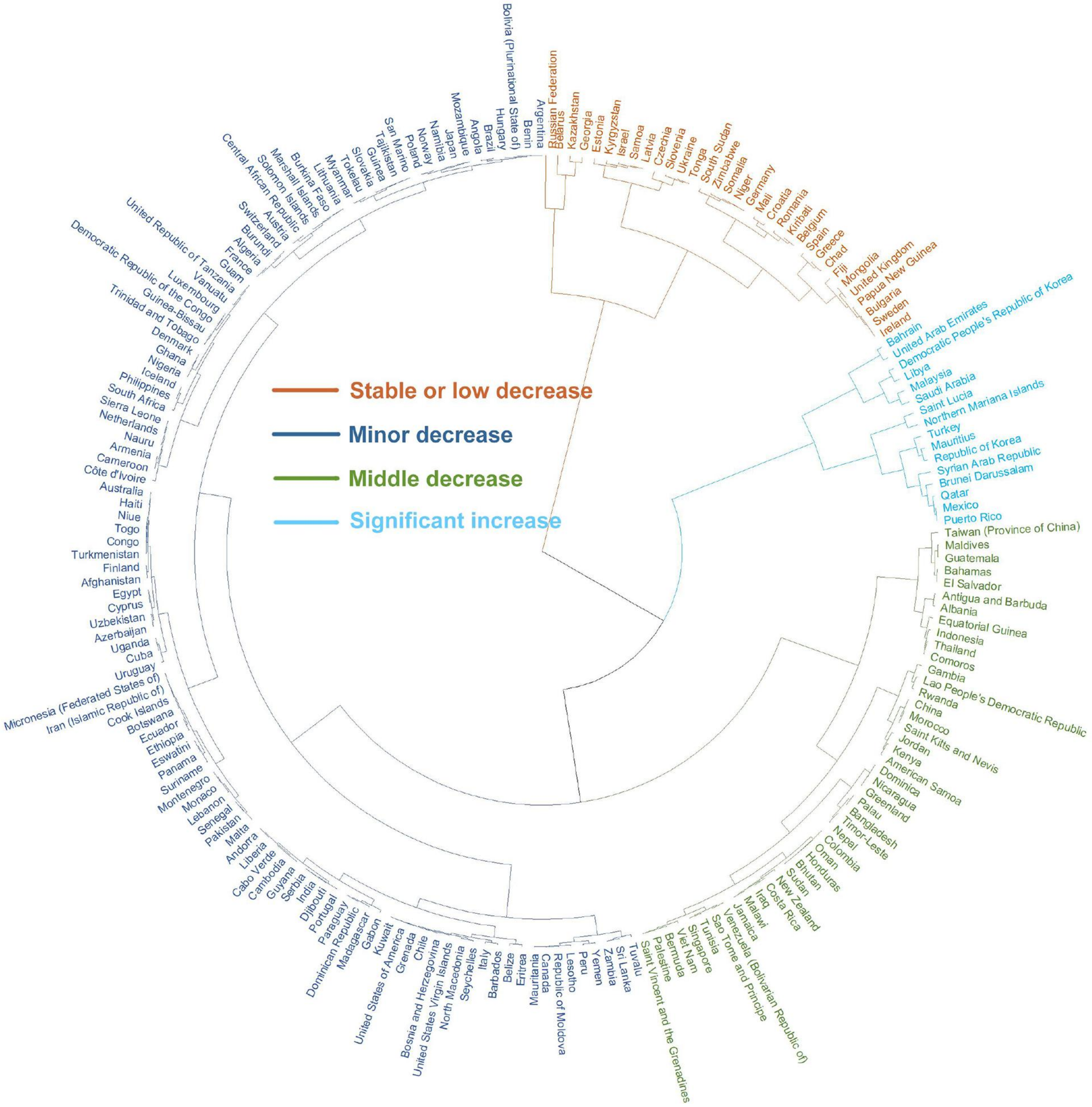

Supplementary Figure S2. The incidence rate of thalassemias in 2019.

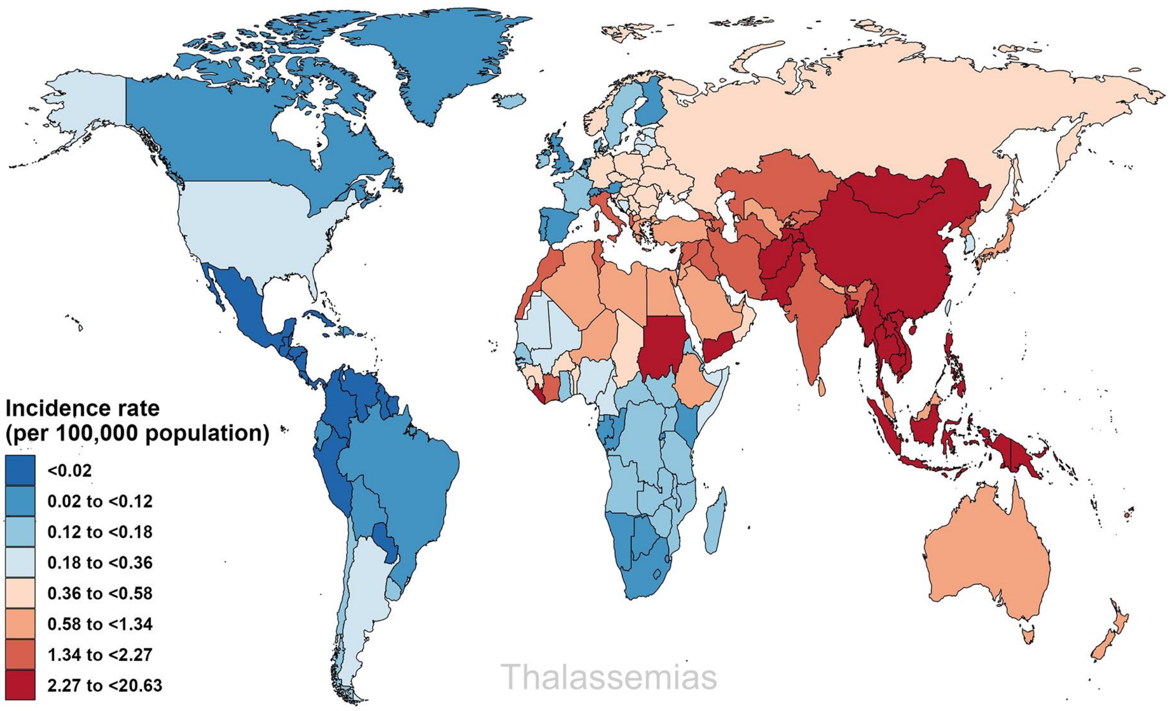

Supplementary Figure S3. The percentage change in incident cases of thalassemias between 1990 and 2019.

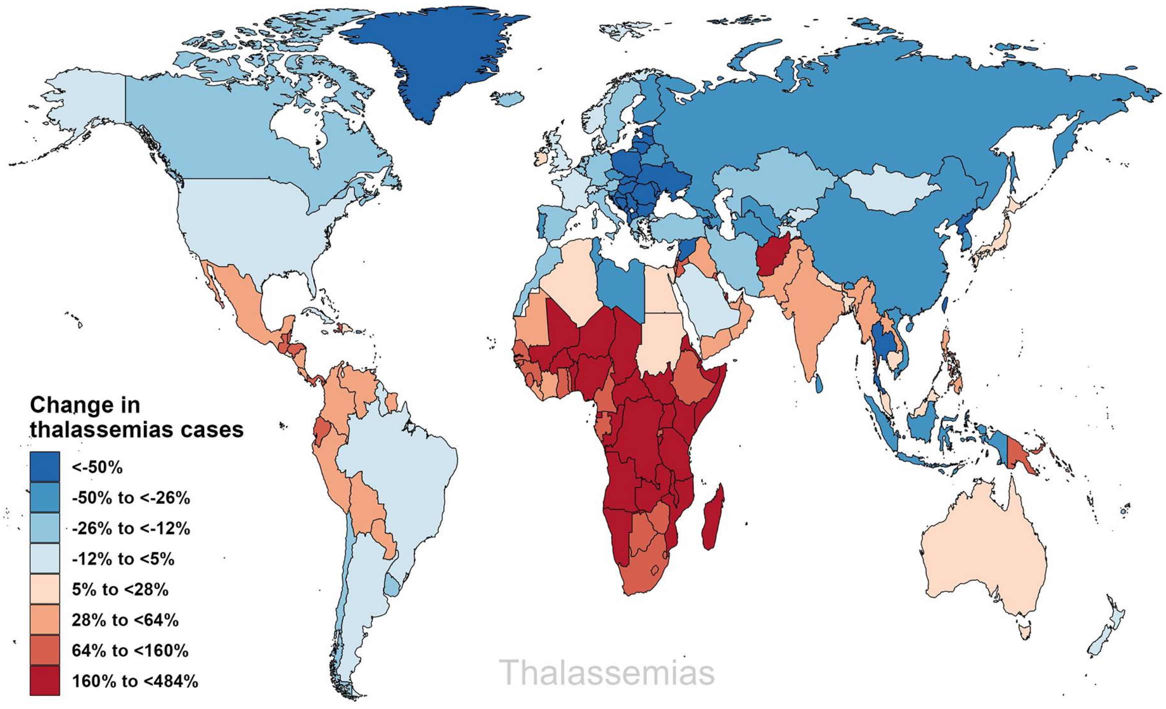

Supplementary Figure S4. The EAPCs in incidence rate of thalassemias from 1990 to 2019.

EAPCs, estimated annual percentage change.

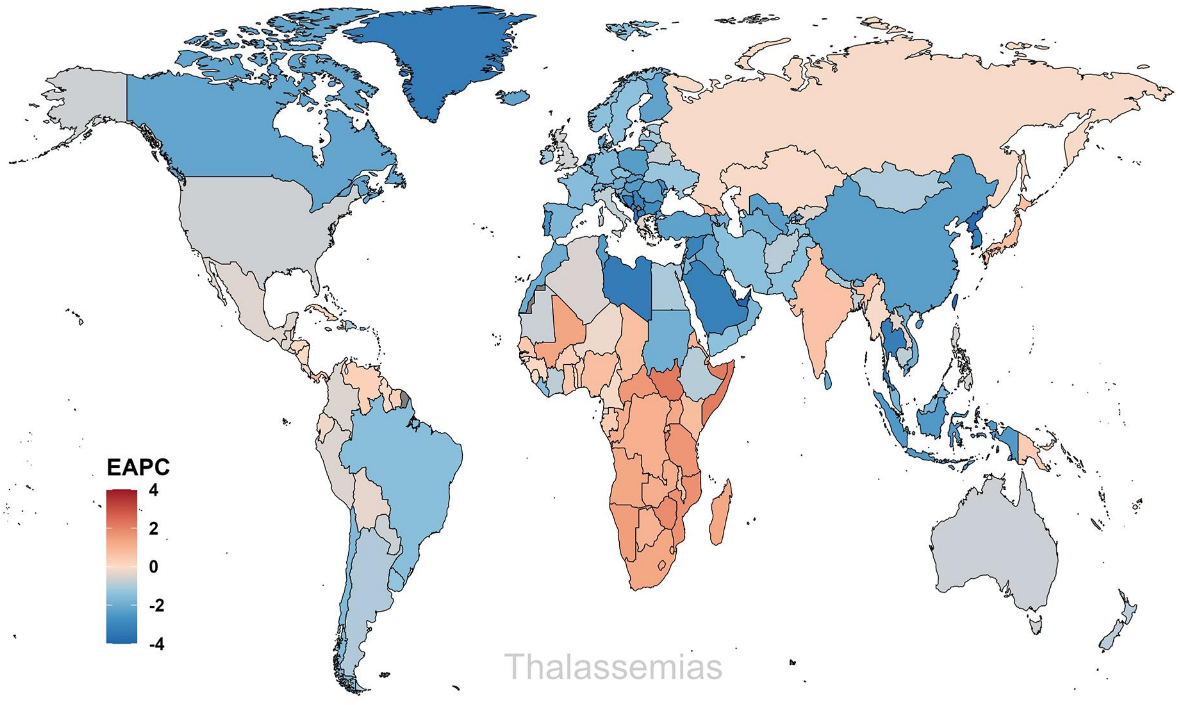

Supplementary Figure S5. The incidence rate of thalassemias trait in 2019.

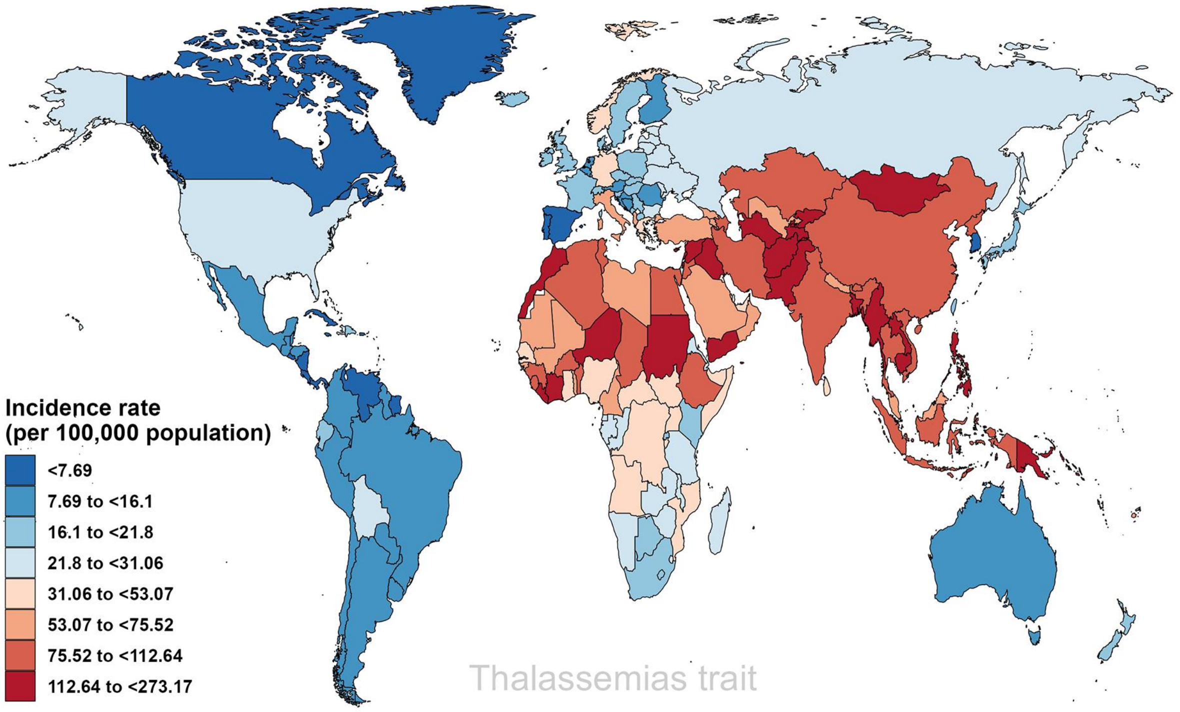

Supplementary Figure S6. The percentage change in incident cases of thalassemias trait between 1990 and 2019.

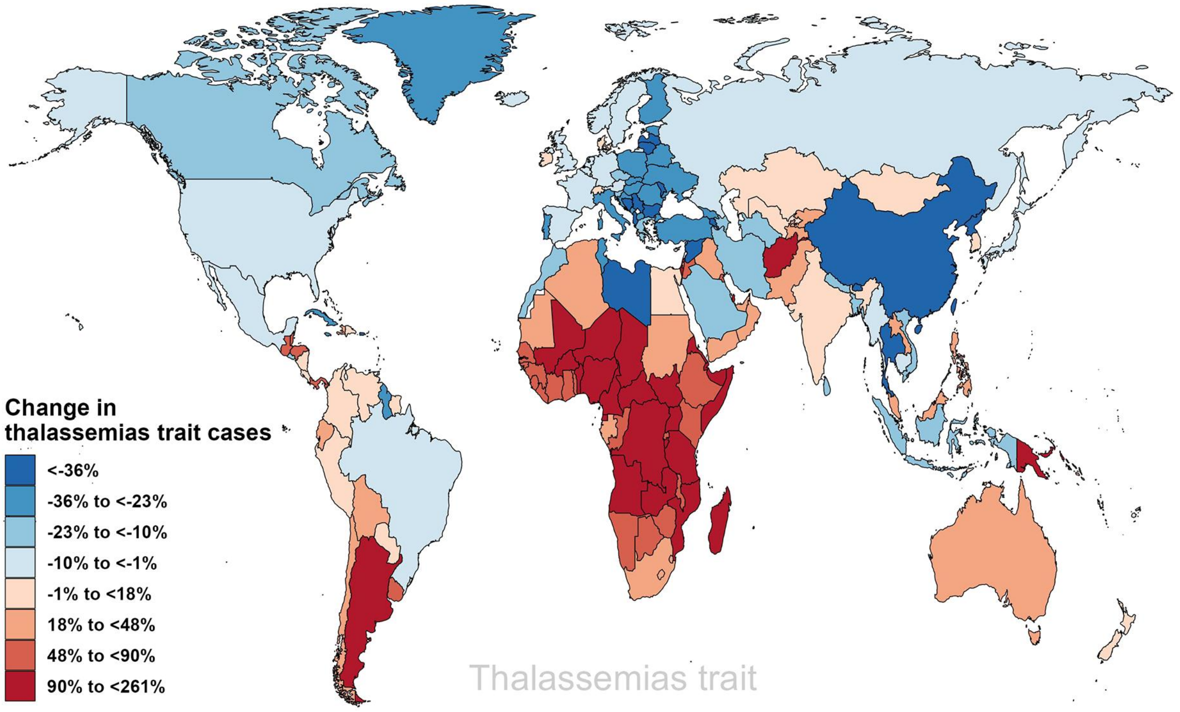

Supplementary Figure S7. The EAPCs in incidence rate of thalassemias trait from 1990 to 2019. EAPCs, estimated annual percentage change.

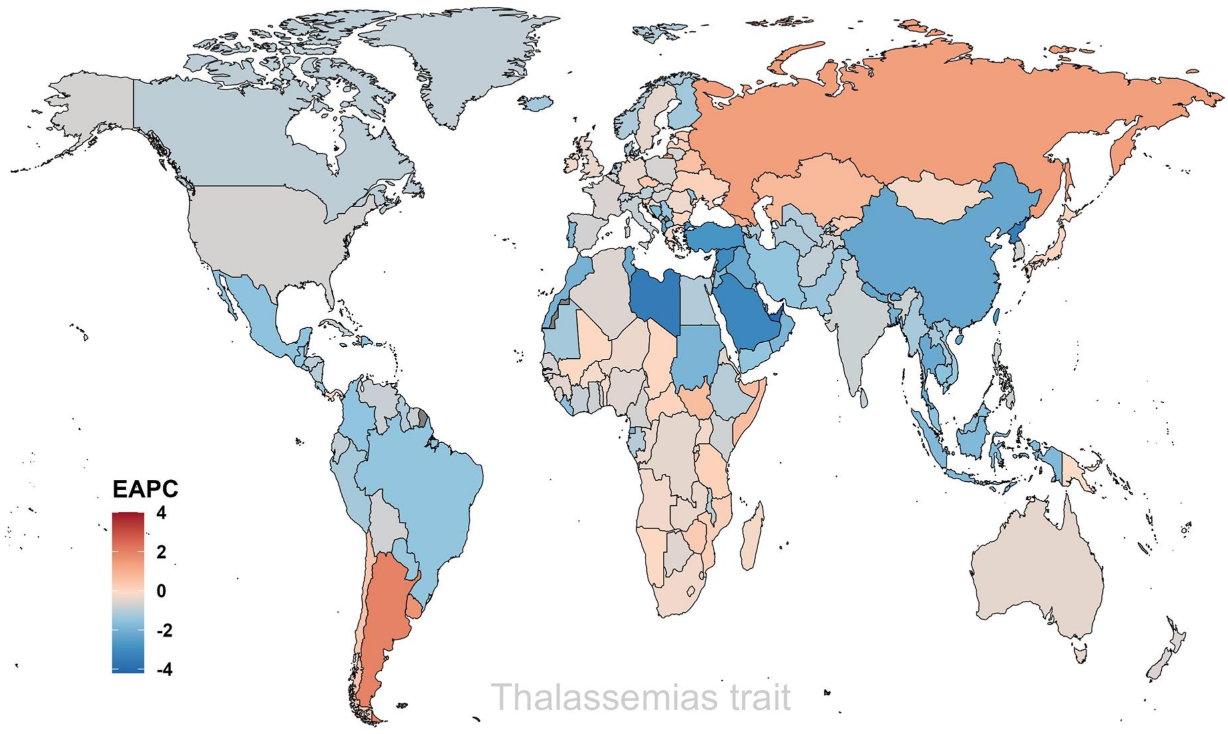

Supplementary Figure S8. The incidence rate of sickle cell disorders in 2019.

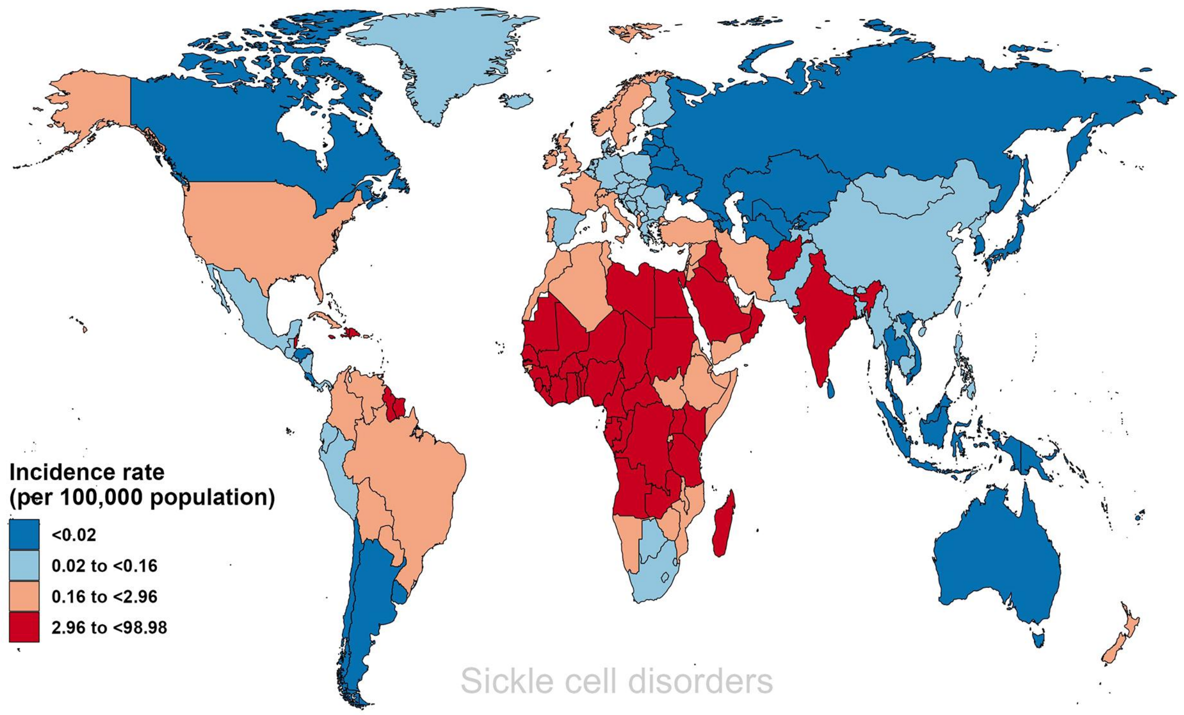

Supplementary Figure S9. The percentage change in incident cases of sickle cell disorders between 1990 and 2019.

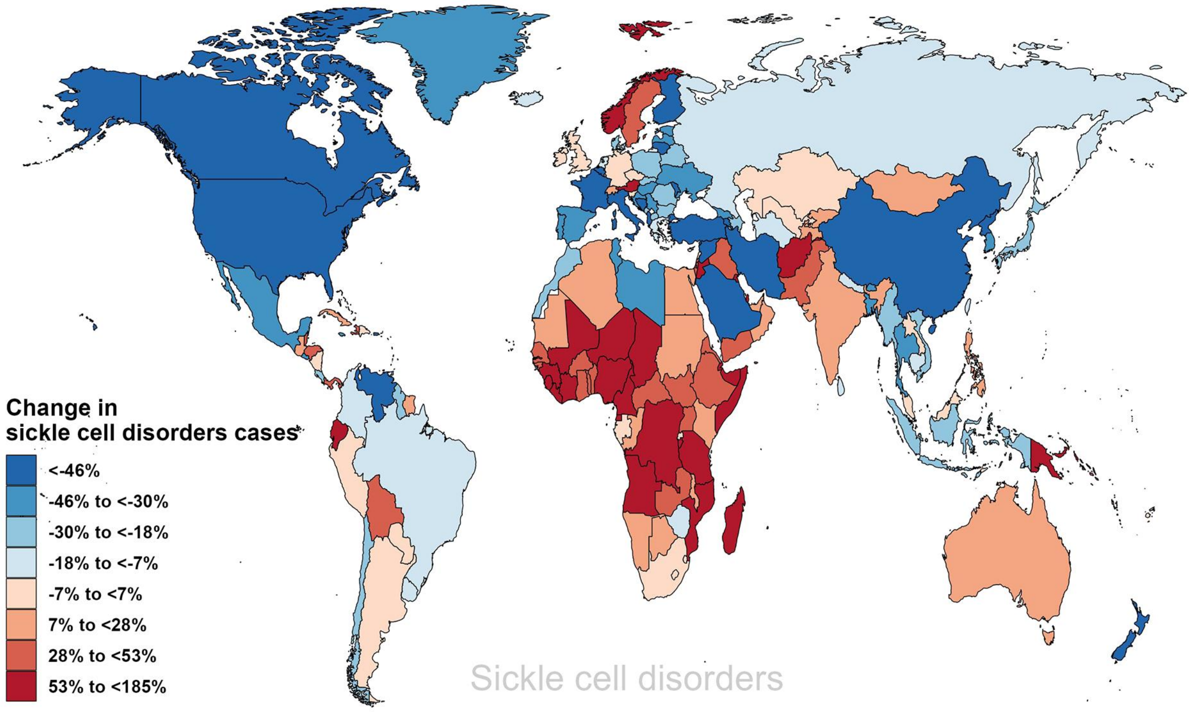

Supplementary Figure S10. The EAPCs in incidence rate of sickle cell disorders from 1990 to 2019. EAPCs, estimated annual percentage change.

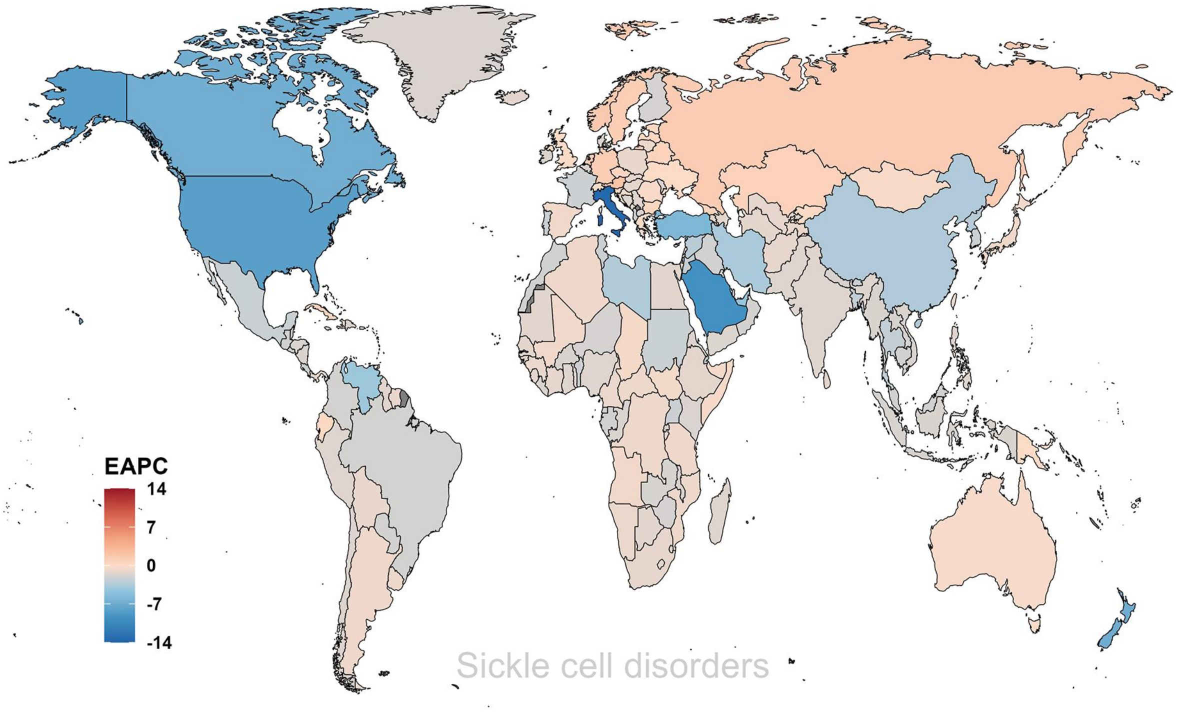

Supplementary Figure S11. The incidence rate of sickle cell trait in 2019.

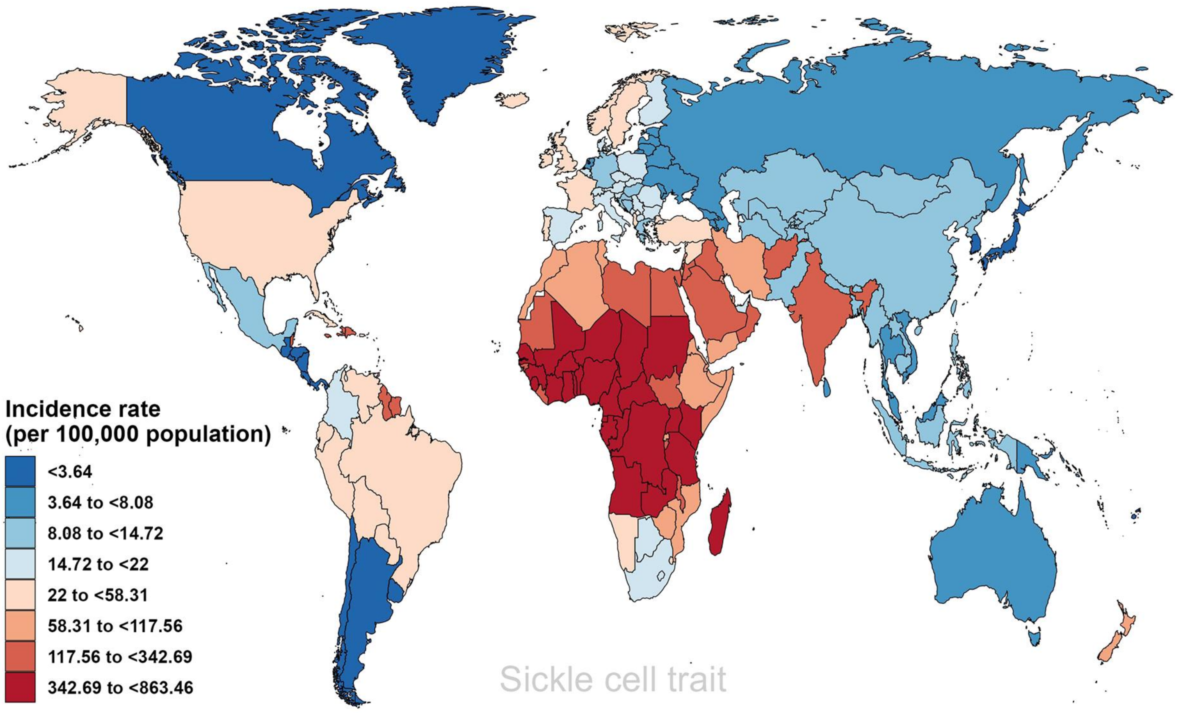

Supplementary Figure S12. The percentage change in incident cases of sickle cell trait between 1990 and 2019.

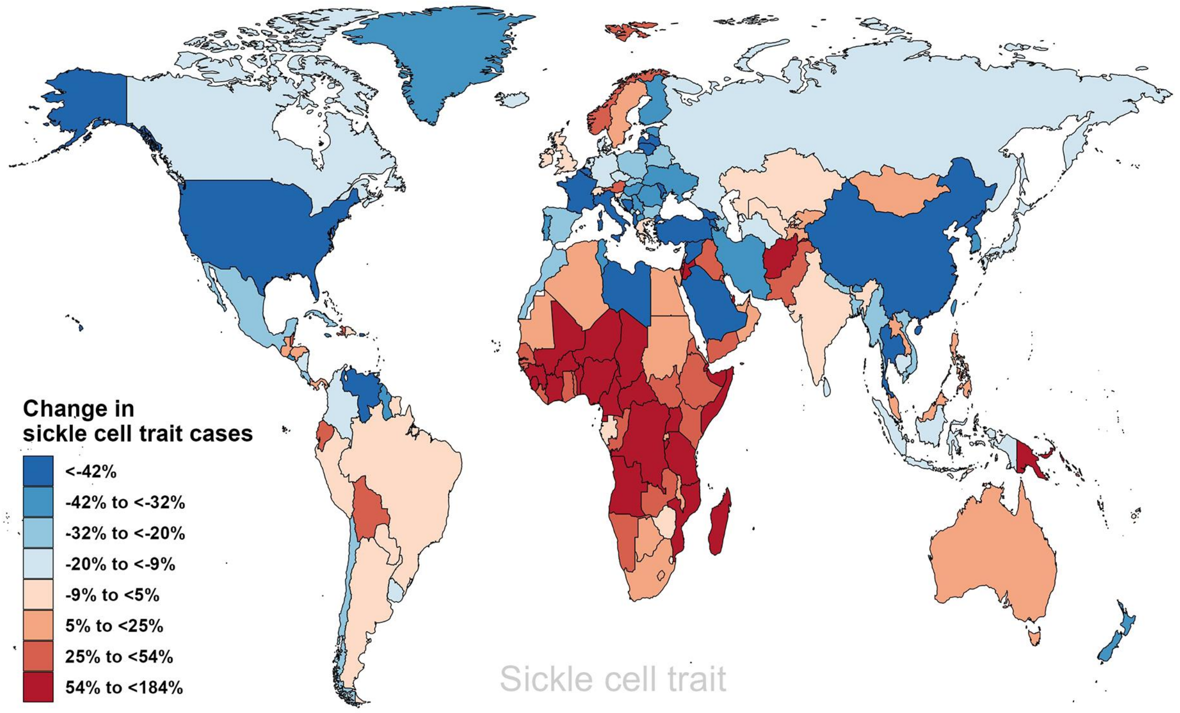

Supplementary Figure S13. The EAPCs in incidence rate of sickle cell trait from 1990 to 2019. EAPCs, estimated annual percentage change.

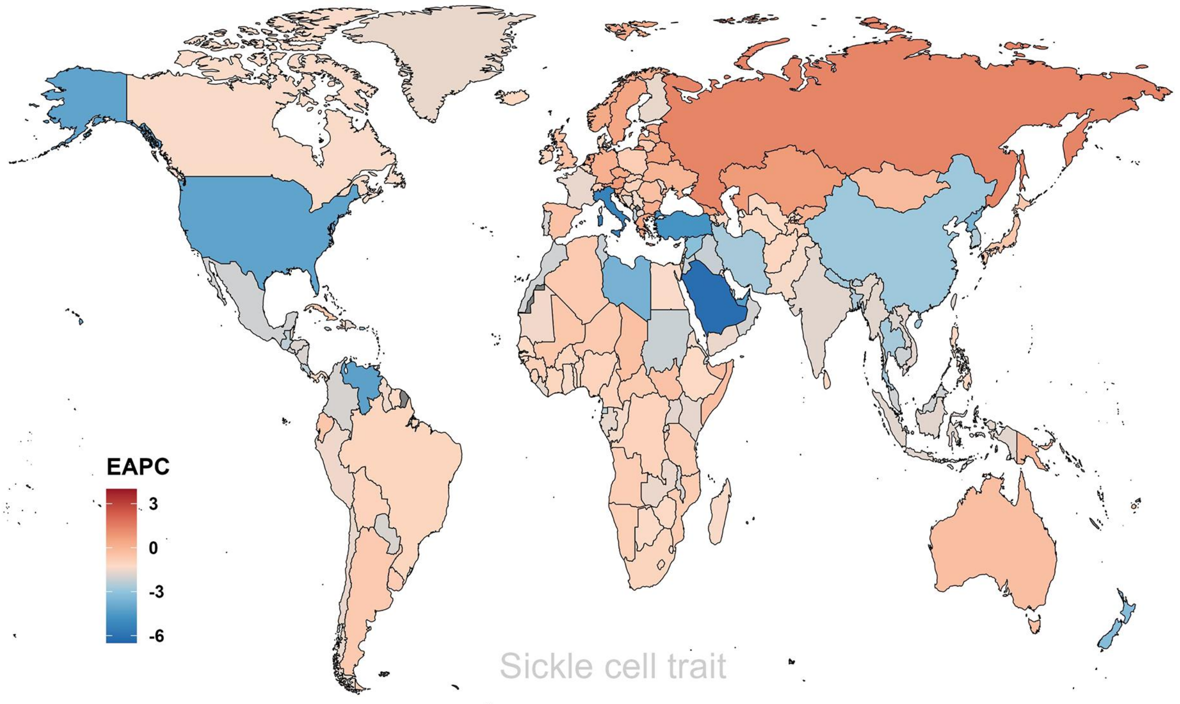

Supplementary Figure S14. The incidence rate of G6PD deficiency in 2019. G6PD, glucose-6-phosphate dehydrogenase.

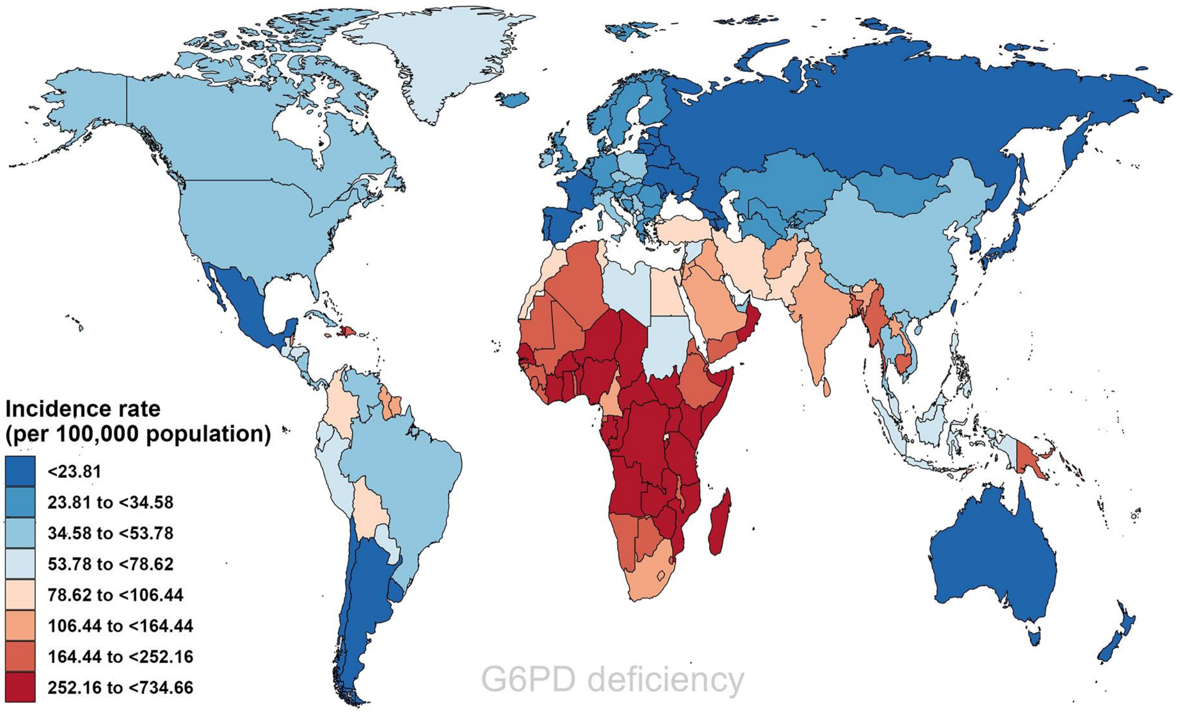

Supplementary Figure S15. The percentage change in incident cases of G6PD deficiency between 1990 and 2019. G6PD, glucose-6-phosphate dehydrogenase.

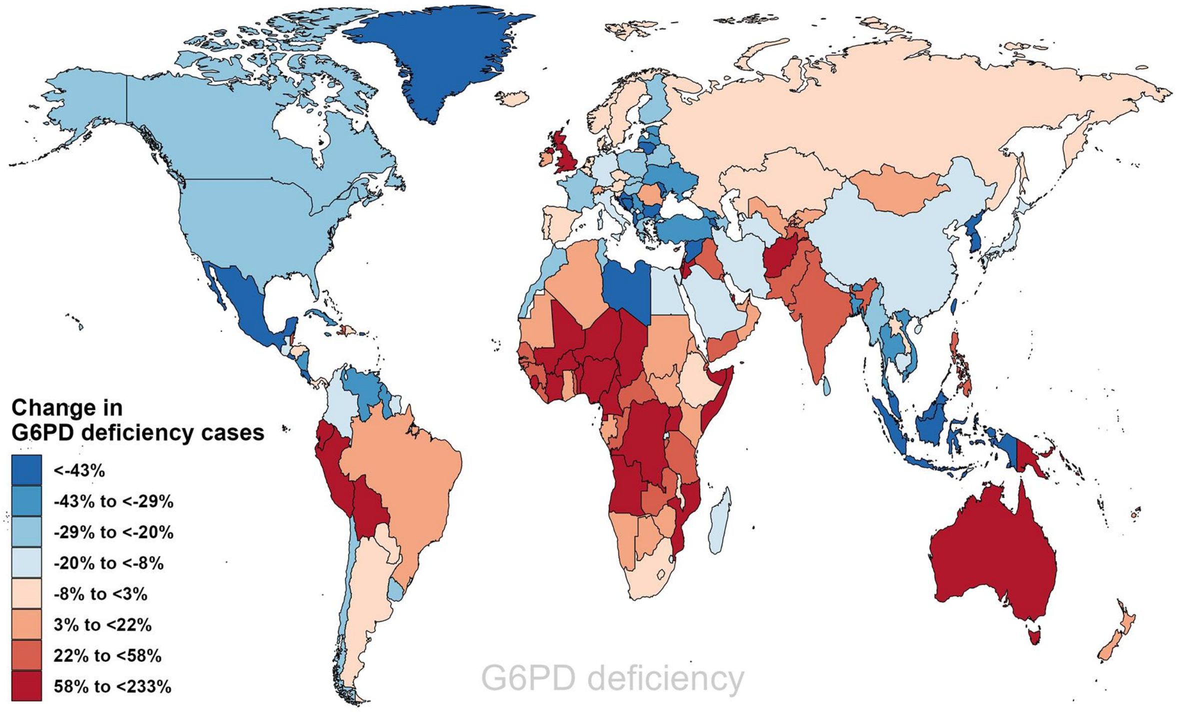

Supplementary Figure S16. The EAPCs in incidence rate of G6PD deficiency from 1990 to 2019. EAPCs, estimated annual percentage change. G6PD, glucose-6-phosphate dehydrogenase.

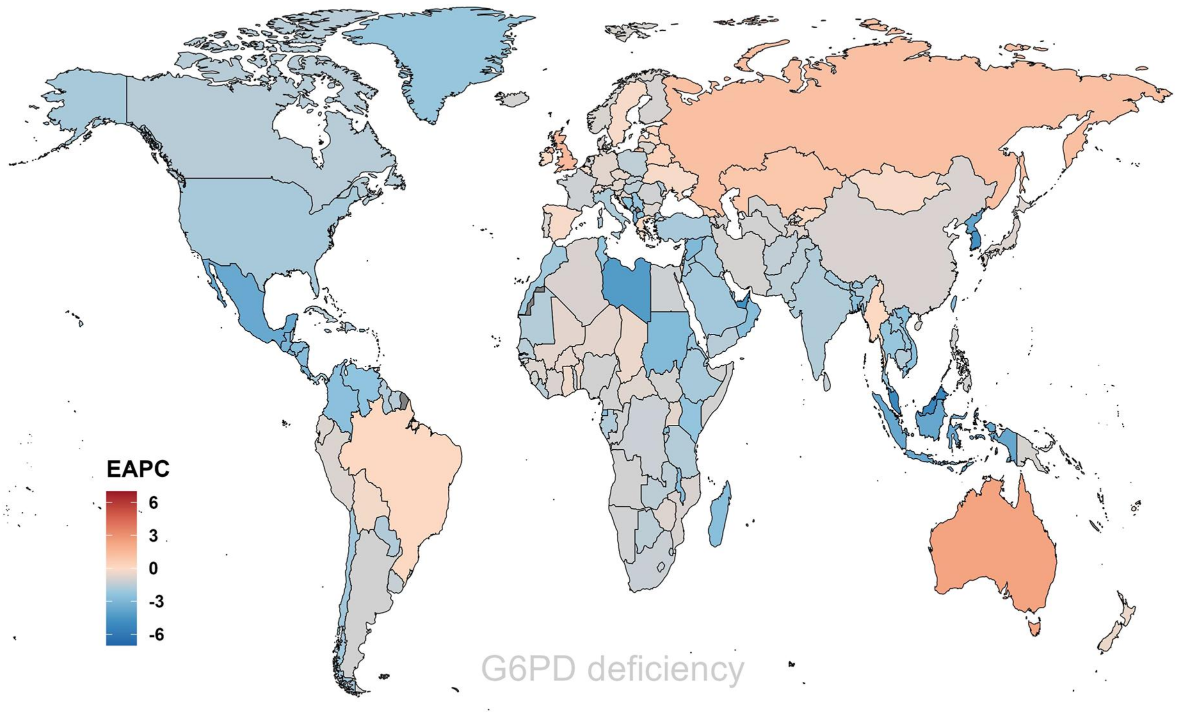

Supplementary Figure S17. The incidence rate of G6PD trait in 2019. G6PD, glucose-6-phosphate dehydrogenase.

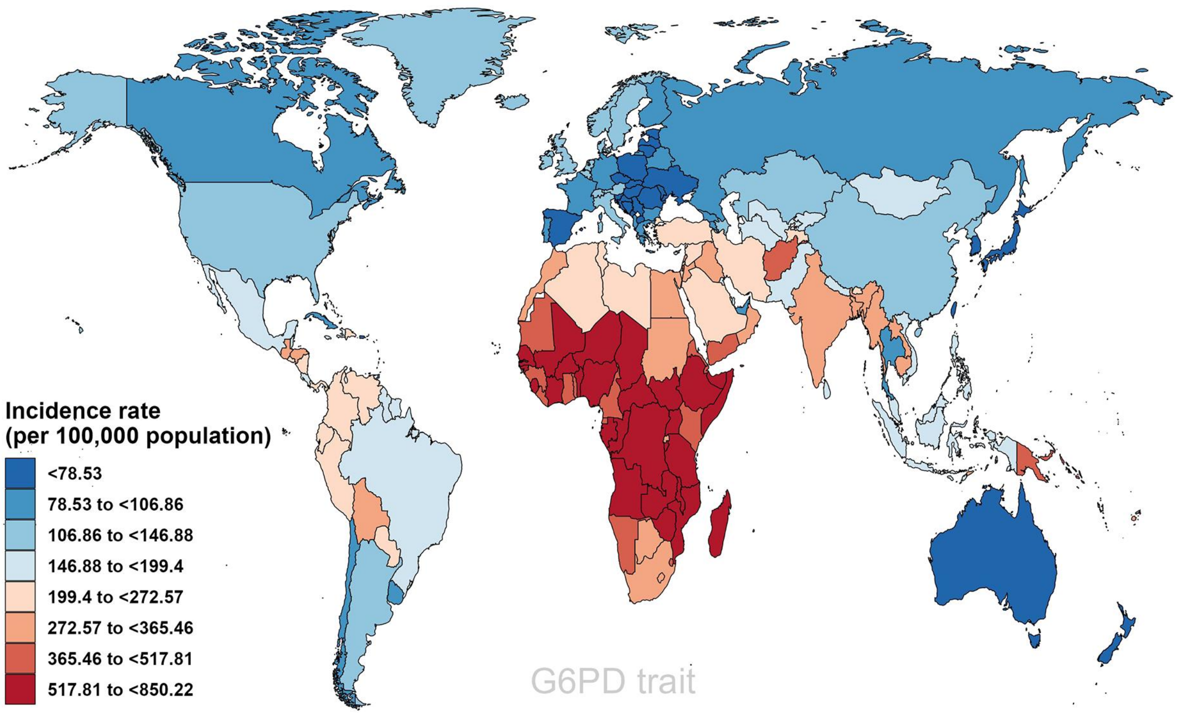

Supplementary Figure S18. The percentage change in incident cases of G6PD trait between 1990 and 2019. G6PD, glucose-6-phosphate dehydrogenase.

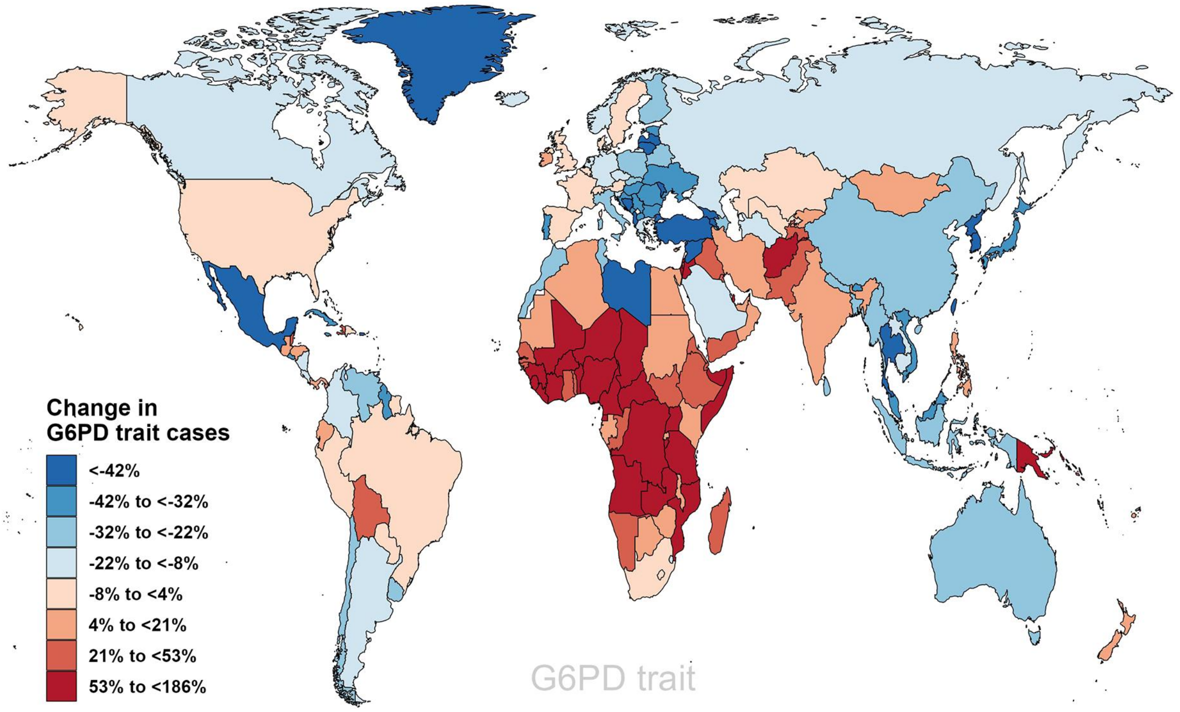

Supplementary Figure S19. The EAPCs in incidence rate of G6PD trait from 1990 to 2019. EAPCs, estimated annual percentage change. G6PD, glucose-6-phosphate dehydrogenase.

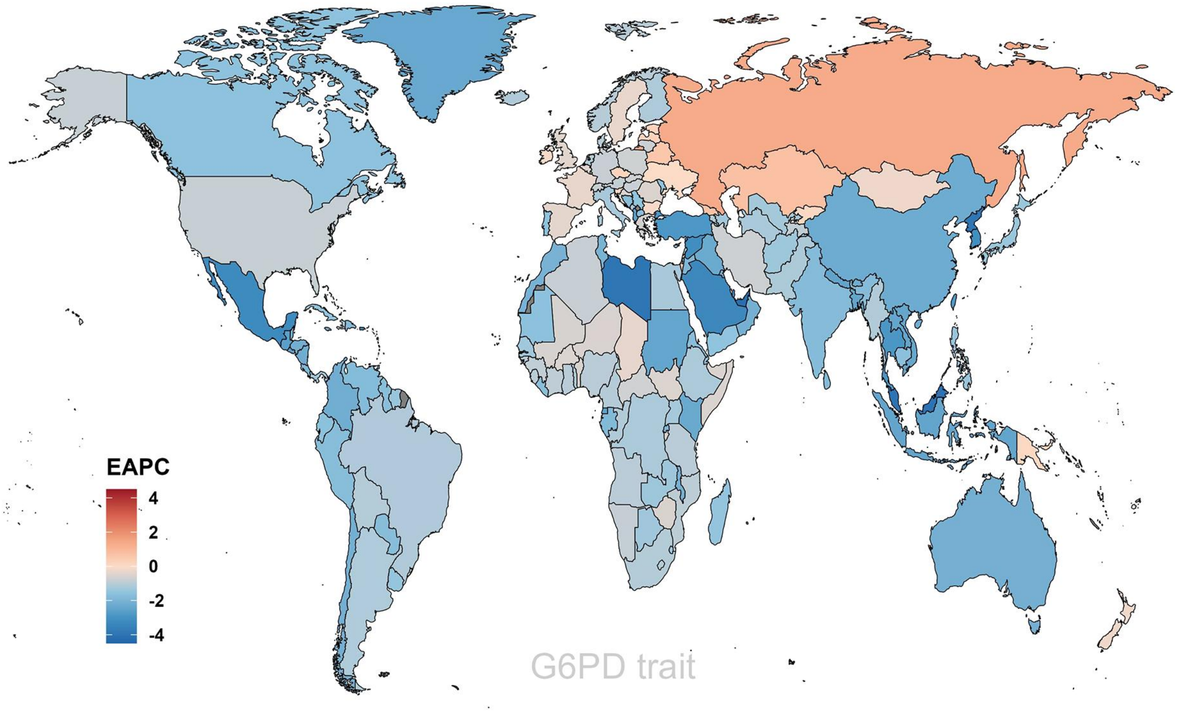

Supplementary Figure S20. The EAPCs in incidence rate of thalassemias from 1990 to 2019, at global, regional and national level for both sexes combined.

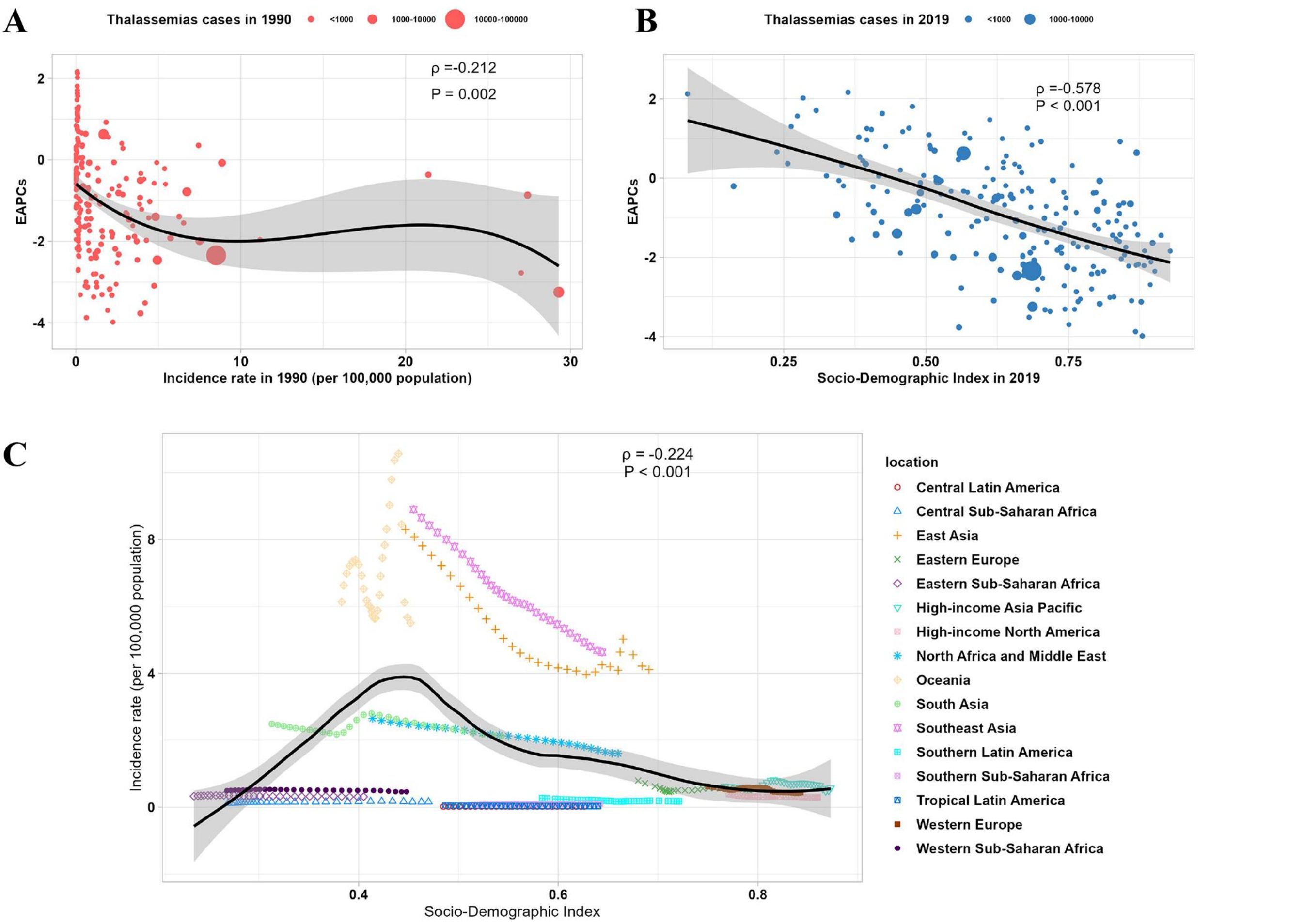

(A) The correlation between EAPCs and thalassemias incidence rate in 1990. (B) The correlation between EAPCs and SDI in 2019. (C) Incidence rate of thalassemias by region from 1990 to 2019 based on SDI levels. EAPCs, estimated annual percentage change. SDI, Socio-Demographic Index.

Supplementary Figure S21. The EAPCs in incidence rate of thalassemias trait from 1990 to 2019, at global, regional and national level for both sexes combined.

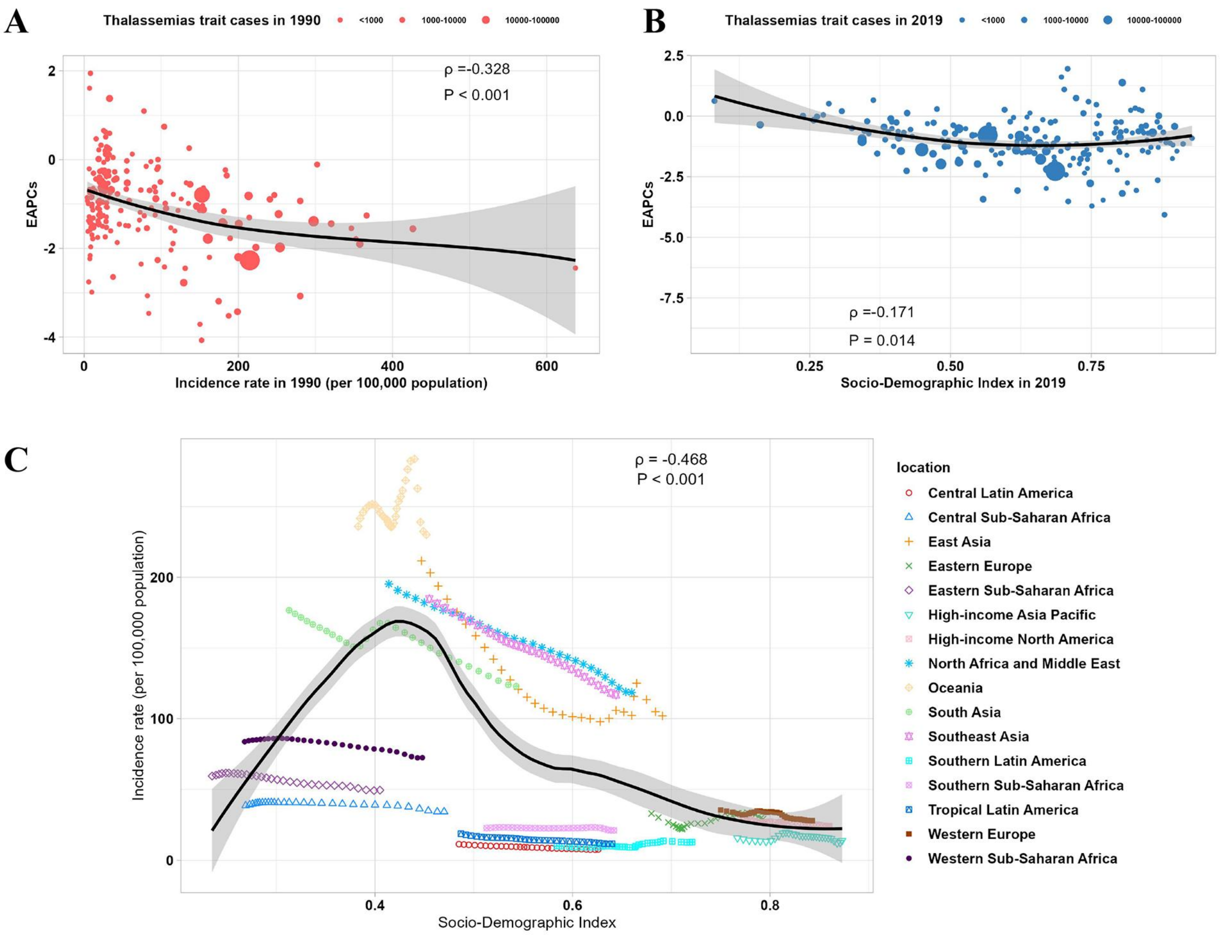

(A) The correlation between EAPCs and thalassemias trait incidence rate in 1990. (B) The correlation between EAPCs and SDI in 2019. (C) Incidence rate of thalassemias trait by region from 1990 to 2019 based on SDI levels. EAPCs, estimated annual percentage change. SDI, Socio-Demographic Index.

Supplementary Figure S22. The EAPCs in incidence rate of sickle cell disorders from 1990 to 2019, at global, regional and national level for both sexes combined.

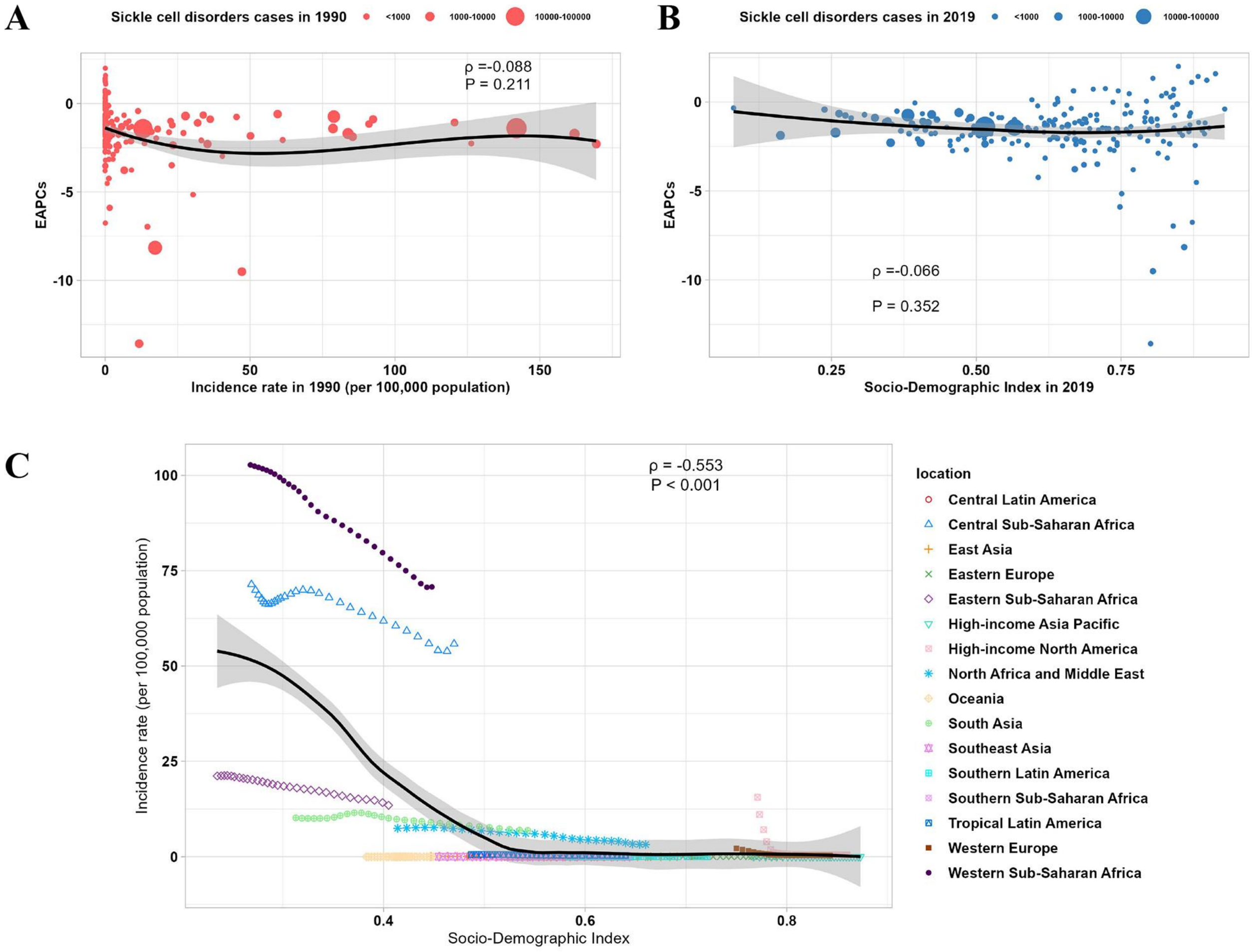

(A) The correlation between EAPCs and sickle cell disorders incidence rate in 1990. (B) The correlation between EAPCs and SDI in 2019. (C) Incidence rate of sickle cell disorders by region from 1990 to 2019 based on SDI levels. EAPCs, estimated annual percentage change. SDI, Socio-Demographic Index.

Supplementary Figure S23. The EAPCs in incidence rate of sickle cell trait from 1990 to 2019, at global, regional and national level for both sexes combined.

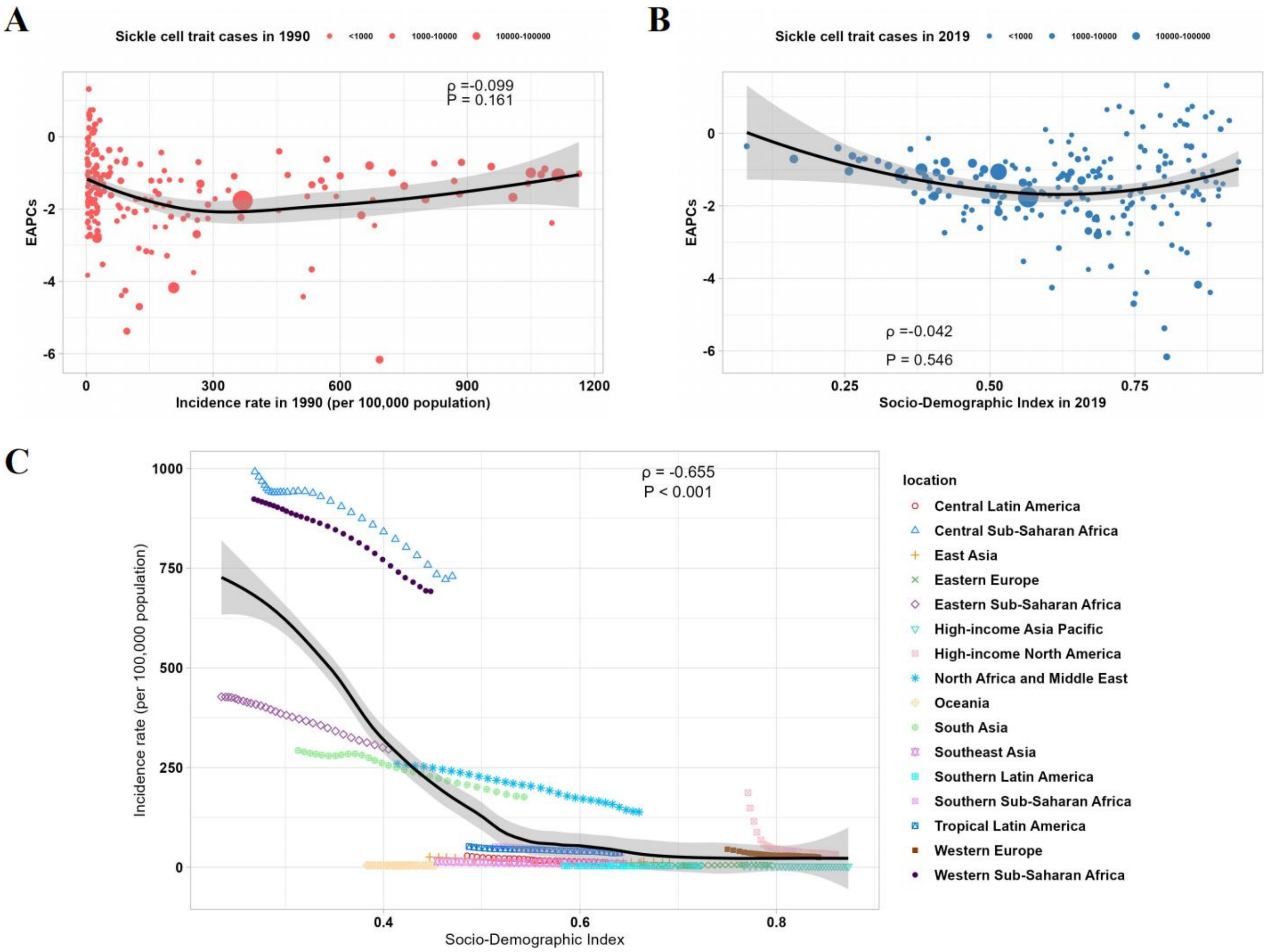

(A) The correlation between EAPCs and sickle cell trait incidence rate in 1990. (B) The correlation between EAPCs and SDI in 2019. (C) Incidence rate of sickle cell trait by region from 1990 to 2019 based on SDI levels. EAPCs, estimated annual percentage change. SDI, Socio-Demographic Index.

Supplementary Figure S24. The EAPCs in incidence rate of G6PD deficiency from 1990 to 2019, at global, regional and national level for both sexes combined.

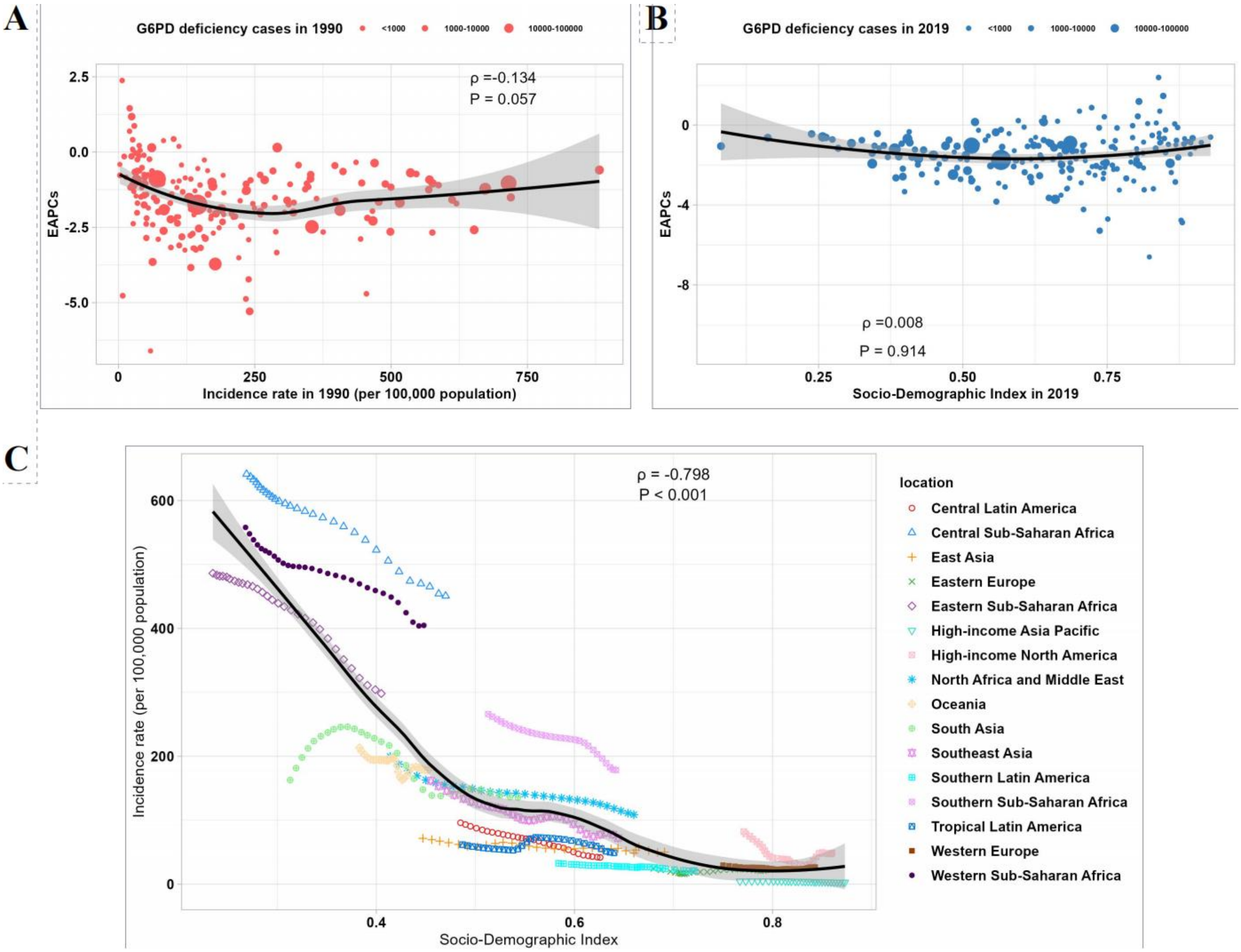

(A) The correlation between EAPCs and G6PD deficiency incidence rate in 1990. (B) The correlation between EAPCs and SDI in 2019. (C) Incidence rate of G6PD deficiency by region from 1990 to 2019 based on SDI levels. EAPCs, estimated annual percentage change. SDI, Socio-Demographic Index.

Supplementary Figure S25. The EAPCs in incidence rate of G6PD trait from 1990 to 2019, at global, regional and national level for both sexes combined.

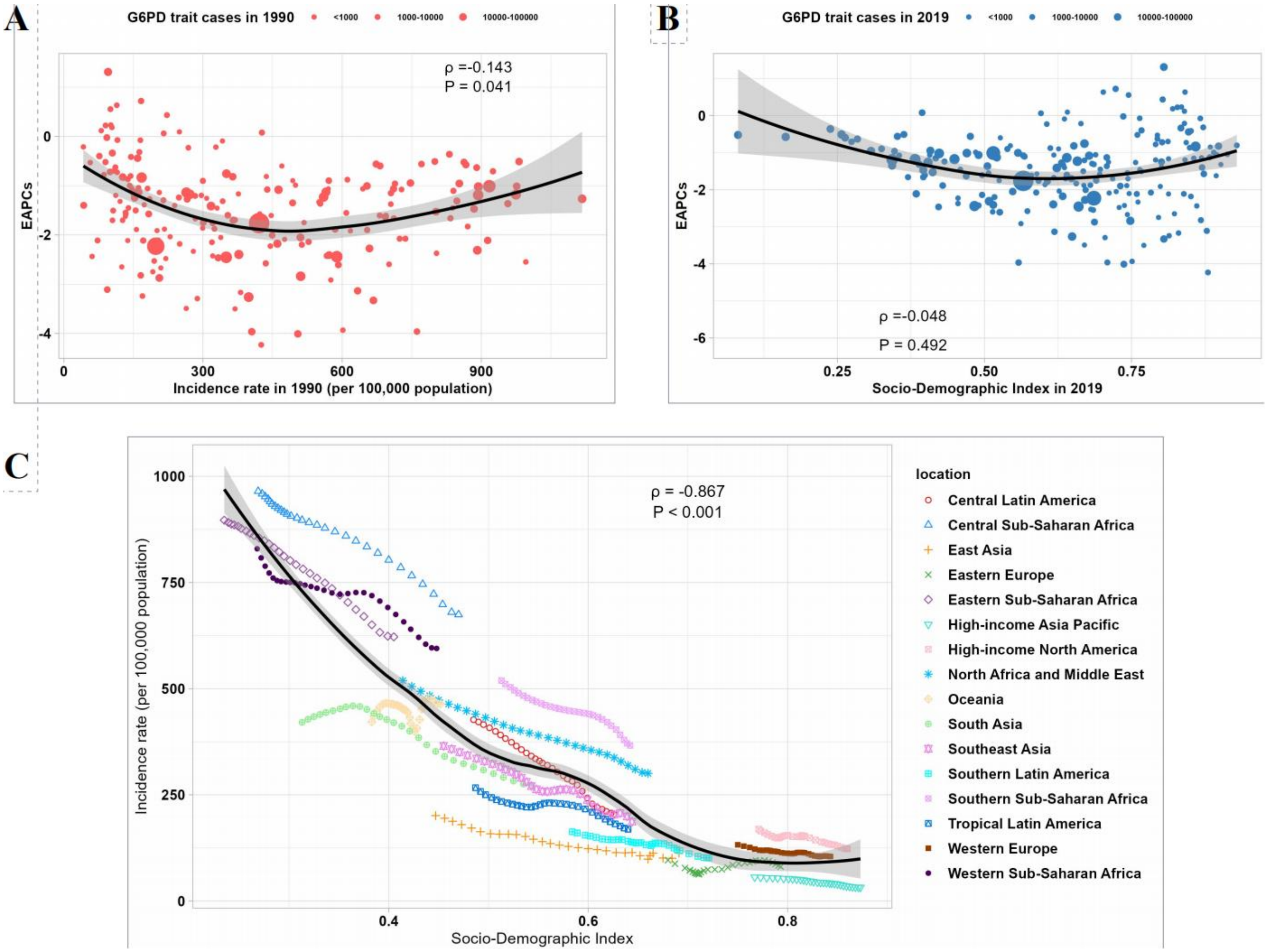

(A) The correlation between EAPCs and G6PD trait incidence rate in 1990. (B) The correlation between EAPCs and SDI in 2019. (C) Incidence rate of G6PD trait by region from 1990 to 2019 based on SDI levels. EAPCs, estimated annual percentage change. SDI, Socio-Demographic Index.

Supplementary Table S1. The incident cases and incidence rates of thalassemias in 1990 and 2019, along with their temporal trend.

Characteristics	1990		2019		1990-2019
	Incident cases No.×10^3(95% UI)	Incidence rates per 100000 (95% UI)	Incident cases No.×10^3(95% UI)	Incidence rates per 100000 (95% UI)	EAPC (95%CI)
**Global**	192.08 (150.02 to 253.13)	3.59 (2.8 to 4.73)	151.9 (118.84 to 200.99)	1.96 (1.54 to 2.6)	-1.9 (-2.08 to -1.71)
**Sex**					
Male	111.07 (85.67 to 146.49)	4.12 (3.18 to 5.44)	90.29 (70.08 to 120.52)	2.33 (1.81 to 3.11)	-1.74 (-1.93 to -1.56)
Female	81.01 (63.6 to 105.05)	3.05 (2.39 to 3.96)	61.6 (48.6 to 80.01)	1.6 (1.26 to 2.07)	-2.11 (-2.31 to -1.91)
**Region**					
Andean Latin America	0.01 (0.01 to 0.02)	0.04 (0.03 to 0.05)	0.02 (0.02 to 0.03)	0.04 (0.03 to 0.04)	-0.24 (-0.34 to -0.14)
Australasia	0.17 (0.11 to 0.26)	0.82 (0.55 to 1.29)	0.18 (0.12 to 0.28)	0.63 (0.43 to 0.97)	-0.76 (-0.91 to -0.6)
Caribbean	0.01 (0.01 to 0.01)	0.03 (0.02 to 0.04)	0.01 (0.01 to 0.02)	0.03 (0.02 to 0.03)	0.02 (-0.07 to 0.11)
Central Asia	2.08 (1.54 to 2.82)	3.01 (2.23 to 4.08)	1.62 (1.23 to 2.15)	1.73 (1.31 to 2.3)	-1.19 (-1.54 to -0.84)
Central Europe	1.53 (1.03 to 2.33)	1.24 (0.84 to 1.89)	0.58 (0.41 to 0.89)	0.51 (0.36 to 0.78)	-2.58 (-2.85 to -2.31)
Central Latin America	0.03 (0.02 to 0.05)	0.02 (0.01 to 0.03)	0.04 (0.02 to 0.06)	0.02 (0.01 to 0.02)	-0.28 (-0.47 to -0.08)
Central Sub-Saharan Africa	0.06 (0.04 to 0.09)	0.11 (0.07 to 0.17)	0.21 (0.12 to 0.32)	0.16 (0.09 to 0.24)	1.11 (0.9 to 1.32)
East Asia	101.64 (72.42 to 144.03)	8.3 (5.91 to 11.76)	60.56 (43.52 to 86.44)	4.11 (2.96 to 5.87)	-2.36 (-2.86 to -1.85)
Eastern Europe	1.8 (1.25 to 2.64)	0.79 (0.55 to 1.17)	0.96 (0.7 to 1.37)	0.46 (0.33 to 0.65)	-0.43 (-0.92 to 0.07)

Eastern Sub-Saharan Africa	0.62 (0.44 to 0.86)	0.33 (0.23 to 0.45)	1.28 (0.9 to 1.73)	0.31 (0.22 to 0.42)	-0.38 (-0.49 to -0.27)
High-income Asia Pacific	1.08 (0.78 to 1.57)	0.62 (0.45 to 0.9)	1.07 (0.78 to 1.52)	0.57 (0.42 to 0.81)	-0.26 (-0.8 to 0.29)
High-income North America	1.1 (0.84 to 1.48)	0.39 (0.3 to 0.53)	1.07 (0.87 to 1.38)	0.29 (0.24 to 0.38)	-0.69 (-0.85 to -0.52)
North Africa and Middle East	9.15 (6.92 to 12.31)	2.65 (2.01 to 3.57)	9.76 (7.51 to 12.86)	1.6 (1.23 to 2.11)	-1.66 (-1.74 to -1.58)
Oceania	0.4 (0.26 to 0.55)	6.13 (4.05 to 8.51)	0.73 (0.49 to 1.03)	5.5 (3.7 to 7.75)	0.56 (-0.19 to 1.33)
South Asia	27.3 (20.7 to 36.31)	2.49 (1.89 to 3.31)	38.3 (28.42 to 51.93)	2.12 (1.57 to 2.88)	-0.13 (-0.45 to 0.2)
Southeast Asia	41.53 (34.5 to 51.9)	8.9 (7.39 to 11.12)	31.21 (25.91 to 38.32)	4.63 (3.85 to 5.69)	-2.13 (-2.2 to -2.06)
Southern Latin America	0.13 (0.09 to 0.2)	0.27 (0.17 to 0.41)	0.12 (0.09 to 0.17)	0.18 (0.13 to 0.25)	-1.18 (-1.48 to -0.89)
Southern Sub-Saharan Africa	0.03 (0.02 to 0.05)	0.07 (0.04 to 0.1)	0.08 (0.05 to 0.12)	0.1 (0.06 to 0.15)	1.36 (1.19 to 1.52)
Tropical Latin America	0.09 (0.06 to 0.12)	0.06 (0.04 to 0.08)	0.08 (0.06 to 0.1)	0.03 (0.03 to 0.04)	-1.48 (-1.59 to -1.38)
Western Europe	2.36 (1.72 to 3.12)	0.61 (0.45 to 0.81)	1.88 (1.42 to 2.43)	0.43 (0.32 to 0.56)	-1.22 (-1.55 to -0.89)
Western Sub-Saharan Africa	0.95 (0.73 to 1.24)	0.49 (0.38 to 0.64)	2.11 (1.6 to 2.69)	0.46 (0.35 to 0.59)	-0.3 (-0.43 to -0.18)

Abbreviations: EAPC, estimated annual percentage change; UI, uncertainty interval. CI, confidence interval.

Supplementary Table S2. The incident cases and incidence rates of thalassemias trait in 1990 and 2019, along with their temporal trend.

Characteristics	1990		2019		1990-2019
	**Incident cases No.×10^3(95% UI)**	**Incidence rates per 100000 (95% UI)**	**Incident cases No.×10^3(95% UI)**	**Incidence rates per 100000 (95% UI)**	**EAPC (95%CI)**
**Global**	6910.25 (6002.38 to 8062.8)	129.17 (112.2 to 150.71)	6337.27 (5491.79 to 7335.76)	81.9 (70.98 to 94.81)	-1.35 (-1.47 to -1.23)
**Sex**					
Male	3798.41 (3302.11 to 4438.7)	141.01 (122.58 to 164.78)	3547.49 (3067.29 to 4121.63)	91.41 (79.03 to 106.2)	-1.24 (-1.37 to -1.12)
Female	3111.84 (2707.23 to 3621.25)	117.16 (101.93 to 136.34)	2789.78 (2430.36 to 3225.03)	72.34 (63.02 to 83.63)	-1.48 (-1.6 to -1.36)
**Region**					
Andean Latin America	8.72 (7.65 to 9.97)	22.84 (20.05 to 26.11)	10.78 (9.45 to 12.12)	16.94 (14.85 to 19.06)	-0.96 (-1 to -0.93)
Australasia	3.48 (3.07 to 3.96)	17.14 (15.14 to 19.54)	4.22 (3.75 to 4.73)	14.52 (12.91 to 16.29)	-0.5 (-0.62 to -0.38)
Caribbean	5.52 (4.8 to 6.36)	15.65 (13.6 to 18.03)	6.2 (5.39 to 7.13)	13.14 (11.43 to 15.11)	-0.59 (-0.62 to -0.55)
Central Asia	88.11 (76.24 to 102.68)	127.21 (110.07 to 148.24)	87.64 (76.28 to 102.1)	93.7 (81.56 to 109.16)	-0.36 (-0.71 to -0.02)
Central Europe	32.12 (27.34 to 38.65)	26.12 (22.24 to 31.43)	20.95 (17.88 to 24.82)	18.34 (15.65 to 21.73)	-0.82 (-1.09 to -0.56)
Central Latin America	18.68 (13.54 to 25)	11.38 (8.25 to 15.23)	19.14 (14.52 to 24.57)	7.66 (5.81 to 9.83)	-1.39 (-1.43 to -1.35)
Central Sub-Saharan Africa	21.48 (17.89 to 25.41)	38.68 (32.21 to 45.77)	45.21 (37.27 to 53.84)	34.37 (28.34 to 40.93)	-0.39 (-0.55 to -0.24)
East Asia	2592.39 (2205.53 to 3108.19)	211.6 (180.02 to 253.7)	1500.93 (1283.84 to 1777.41)	101.95 (87.21 to 120.73)	-2.29 (-2.83 to -1.74)

Eastern Europe	74.77 (62.24 to 91.6)	33.01 (27.48 to 40.44)	61.16 (51.19 to 74.1)	29.13 (24.38 to 35.29)	0.98 (0.5 to 1.46)
Eastern Sub-Saharan Africa	113.12 (96.03 to 131.05)	59.49 (50.5 to 68.91)	203.88 (173.25 to 236.97)	49.51 (42.07 to 57.55)	-0.81 (-0.9 to -0.71)
High-income Asia Pacific	27.25 (21.04 to 33.88)	15.7 (12.13 to 19.52)	26.05 (20.85 to 31.16)	13.91 (11.13 to 16.64)	-0.18 (-0.72 to 0.37)
High-income North America	90.37 (77.65 to 105)	32.17 (27.64 to 37.38)	88.32 (79.01 to 98.97)	24.23 (21.67 to 27.15)	-0.69 (-0.83 to -0.55)
North Africa and Middle East	673.7 (592.9 to 772.2)	195.26 (171.84 to 223.81)	721.7 (639.68 to 818.46)	118.56 (105.09 to 134.46)	-1.65 (-1.71 to -1.59)
Oceania	15.27 (13.08 to 17.5)	235.95 (202.1 to 270.43)	30.55 (26.35 to 35.11)	230.09 (198.47 to 264.44)	0.19 (-0.03 to 0.41)
South Asia	1939.35 (1658.74 to 2266.66)	176.69 (151.12 to 206.51)	2219.25 (1883.56 to 2617.53)	122.94 (104.34 to 145)	-1.02 (-1.22 to -0.81)
Southeast Asia	862.28 (769.55 to 972.62)	184.72 (164.86 to 208.36)	787.29 (702.45 to 891.94)	116.85 (104.25 to 132.38)	-1.45 (-1.52 to -1.39)
Southern Latin America	5.01 (4.37 to 5.77)	10.1 (8.83 to 11.66)	8.58 (7.41 to 9.78)	12.85 (11.1 to 14.65)	1.39 (1.06 to 1.73)
Southern Sub-Saharan Africa	11.95 (10.14 to 14.04)	22.77 (19.32 to 26.75)	16.64 (13.67 to 19.63)	21.17 (17.4 to 24.99)	-0.16 (-0.25 to -0.08)
Tropical Latin America	28.76 (24.4 to 33.58)	18.81 (15.96 to 21.96)	26.16 (22.34 to 30.07)	11.7 (9.99 to 13.45)	-1.45 (-1.52 to -1.37)
Western Europe	136.73 (118.77 to 156.38)	35.55 (30.88 to 40.66)	122 (106.57 to 138.61)	27.96 (24.43 to 31.77)	-0.77 (-0.94 to -0.59)
Western Sub-Saharan Africa	161.2 (140.25 to 187.33)	83.71 (72.83 to 97.28)	330.63 (287.22 to 379.33)	72.46 (62.94 to 83.13)	-0.56 (-0.67 to -0.45)

Abbreviations: EAPC, estimated annual percentage change; UI, uncertainty interval. CI, confidence interval.

Supplementary Table S3. The incident cases and incidence rates of sickle cell disorders in 1990 and 2019, along with their temporal trend.

Characteristics	1990		2019		1990-2019
	Incident cases No.×10^3(95% UI)	Incidence rates per 100000 (95% UI)	Incident cases No.×10^3(95% UI)	Incidence rates per 100000 (95% UI)	EAPC (95%CI)
**Global**	474.73 (373.95 to 596)	8.87 (6.99 to 11.14)	605.01 (466.3 to 780.99)	7.82 (6.03 to 10.09)	-0.31 (-0.42 to -0.19)
**Sex**					
Male	259.08 (207.08 to 319.81)	9.62 (7.69 to 11.87)	305.82 (236.65 to 393.15)	7.88 (6.1 to 10.13)	-0.41 (-0.53 to -0.29)
Female	215.65 (165.67 to 278.46)	8.12 (6.24 to 10.48)	299.2 (229.39 to 387.34)	7.76 (5.95 to 10.04)	-0.2 (-0.34 to -0.06)
**Region**					
Andean Latin America	0.06 (0.05 to 0.09)	0.16 (0.12 to 0.22)	0.08 (0.06 to 0.11)	0.12 (0.09 to 0.17)	-0.86 (-0.92 to -0.8)
Australasia	0.5 (0.33 to 0.76)	2.48 (1.62 to 3.73)	0.14 (0.1 to 0.19)	0.47 (0.33 to 0.66)	-7 (-9.16 to -4.79)
Caribbean	3.53 (2.97 to 4.18)	10 (8.42 to 11.85)	3.92 (3.3 to 4.76)	8.31 (6.99 to 10.08)	-0.62 (-0.77 to -0.47)
Central Asia	0.01 (0.01 to 0.02)	0.02 (0.01 to 0.02)	0.01 (0.01 to 0.02)	0.01 (0.01 to 0.02)	-0.45 (-0.78 to -0.11)
Central Europe	0.16 (0.12 to 0.21)	0.13 (0.1 to 0.17)	0.11 (0.08 to 0.14)	0.1 (0.07 to 0.13)	-0.84 (-1.05 to -0.64)
Central Latin America	0.48 (0.37 to 0.63)	0.29 (0.22 to 0.38)	0.29 (0.21 to 0.38)	0.12 (0.08 to 0.15)	-3.04 (-3.17 to -2.9)
Central Sub-Saharan Africa	39.66 (31.37 to 49.56)	71.43 (56.5 to 89.27)	73.44 (59.26 to 92.12)	55.83 (45.05 to 70.03)	-0.76 (-0.96 to -0.56)
East Asia	1.13 (0.78 to 1.61)	0.09 (0.06 to 0.13)	0.51 (0.36 to 0.71)	0.03 (0.02 to 0.05)	-3.5 (-4.16 to -2.83)
Eastern Europe	0.02 (0.01 to 0.03)	0.01 (0.01 to 0.01)	0.02 (0.01 to 0.02)	0.01 (0 to 0.01)	0.92 (0.46 to 1.38)

Eastern Sub-Saharan Africa	40.22 (30.96 to 52.61)	21.15 (16.28 to 27.66)	55.47 (43.1 to 71.18)	13.47 (10.47 to 17.28)	-1.51 (-1.63 to -1.38)
High-income Asia Pacific	0.01 (0.01 to 0.02)	0.01 (0 to 0.01)	0.01 (0.01 to 0.01)	0 (0 to 0.01)	-1.48 (-1.57 to -1.4)
High-income North America	43.77 (32.67 to 58.09)	15.58 (11.63 to 20.68)	1.7 (1.52 to 1.89)	0.47 (0.42 to 0.52)	-8.16 (-10.49 to -5.77)
North Africa and Middle East	25.73 (22.24 to 30.11)	7.46 (6.44 to 8.73)	19.47 (16.06 to 23.54)	3.2 (2.64 to 3.87)	-3.15 (-3.42 to -2.88)
Oceania	0 (0 to 0)	0 (0 to 0.01)	0 (0 to 0)	0 (0 to 0.01)	0 (-0.12 to 0.11)
South Asia	111.89 (75.34 to 162.73)	10.19 (6.86 to 14.83)	124.43 (83.25 to 179.19)	6.89 (4.61 to 9.93)	-1.46 (-1.8 to -1.12)
Southeast Asia	0.1 (0.08 to 0.15)	0.02 (0.02 to 0.03)	0.09 (0.07 to 0.13)	0.01 (0.01 to 0.02)	-1.65 (-1.72 to -1.59)
Southern Latin America	0 (0 to 0)	0 (0 to 0.01)	0 (0 to 0)	0 (0 to 0)	-0.93 (-1.08 to -0.79)
Southern Sub-Saharan Africa	0.21 (0.17 to 0.27)	0.41 (0.32 to 0.52)	0.2 (0.15 to 0.26)	0.26 (0.2 to 0.33)	-1.87 (-2.06 to -1.68)
Tropical Latin America	0.92 (0.75 to 1.12)	0.6 (0.49 to 0.73)	0.83 (0.69 to 1.01)	0.37 (0.31 to 0.45)	-2.04 (-2.19 to -1.89)
Western Europe	8.41 (6.16 to 11.3)	2.19 (1.6 to 2.94)	1.44 (1.11 to 1.84)	0.33 (0.26 to 0.42)	-5.04 (-6.17 to -3.9)
Western Sub-Saharan Africa	197.91 (158.6 to 246.78)	102.77 (82.35 to 128.14)	322.85 (248.23 to 412.82)	70.75 (54.4 to 90.47)	-1.4 (-1.5 to -1.3)

Abbreviations: EAPC, estimated annual percentage change; UI, uncertainty interval. CI, confidence interval.

Supplementary Table S4. The incident cases and incidence rates of sickle cell trait in 1990 and 2019, along with their temporal trend.

Characteristics	1990		2019		1990-2019
	Incident cases No.×10^3(95% UI)	Incidence rates per 100000 (95% UI)	Incident cases No.×10^3(95% UI)	Incidence rates per 100000 (95% UI)	EAPC (95%CI)
**Global**	8662.5 (7659.58 to 9796.58)	161.92 (143.17 to 183.12)	10113.13 (8915.24 to 11439.3)	130.7 (115.22 to 147.84)	-0.65 (-0.73 to -0.57)
**Sex**					
Male	4481.61 (3959.48 to 5054)	166.37 (146.99 to 187.62)	5133 (4526.28 to 5817.39)	132.26 (116.63 to 149.89)	-0.65 (-0.74 to -0.57)
Female	4180.89 (3684.59 to 4746.43)	157.41 (138.72 to 178.7)	4980.13 (4393.39 to 5628.01)	129.14 (113.92 to 145.94)	-0.65 (-0.73 to -0.57)
**Region**					
Andean Latin America	16.67 (14.11 to 19.62)	43.66 (36.95 to 51.38)	19.61 (16.68 to 22.99)	30.83 (26.23 to 36.15)	-1.12 (-1.16 to -1.07)
Australasia	8.09 (6.87 to 9.58)	39.9 (33.89 to 47.22)	5.89 (5.07 to 6.91)	20.28 (17.45 to 23.78)	-2.49 (-3.17 to -1.8)
Caribbean	85.42 (76.98 to 94.85)	242.17 (218.24 to 268.88)	87.65 (78.8 to 97.96)	185.84 (167.07 to 207.69)	-0.86 (-0.98 to -0.75)
Central Asia	9.11 (7.29 to 11.19)	13.16 (10.52 to 16.15)	8.82 (7.04 to 10.76)	9.43 (7.53 to 11.5)	-0.46 (-0.8 to -0.13)
Central Europe	30.27 (26.2 to 34.72)	24.61 (21.31 to 28.23)	20.13 (17.32 to 23.09)	17.62 (15.16 to 20.21)	-0.8 (-1.02 to -0.58)
Central Latin America	47.35 (40.41 to 56.26)	28.85 (24.62 to 34.28)	30.8 (26.42 to 36.51)	12.32 (10.57 to 14.6)	-2.79 (-2.9 to -2.69)
Central Sub-Saharan Africa	550.82 (495.39 to 609.03)	992.11 (892.28 to 1096.95)	960.08 (872.88 to 1062.1)	729.86 (663.56 to 807.41)	-0.96 (-1.13 to -0.79)
East Asia	313.85 (258.22 to 377.77)	25.62 (21.08 to 30.84)	164.15 (137.37 to 196.44)	11.15 (9.33 to 13.34)	-2.81 (-3.4 to -2.22)

Eastern Europe	14.12 (11.42 to 17.14)	6.24 (5.04 to 7.57)	11.24 (9.18 to 13.64)	5.35 (4.37 to 6.5)	0.91 (0.43 to 1.39)
Eastern Sub-Saharan Africa	813.24 (732.67 to 906.27)	427.64 (385.28 to 476.56)	1217.76 (1105.38 to 1350.7)	295.73 (268.44 to 328.02)	-1.3 (-1.41 to -1.19)
High-income Asia Pacific	6.41 (4.96 to 8.03)	3.69 (2.86 to 4.63)	4.5 (3.57 to 5.54)	2.4 (1.91 to 2.96)	-1.39 (-1.47 to -1.31)
High-income North America	525.73 (471.79 to 584.4)	187.14 (167.94 to 208.03)	118.78 (111.47 to 126.88)	32.58 (30.58 to 34.8)	-4.16 (-5.13 to -3.18)
North Africa and Middle East	894.65 (826.66 to 975.47)	259.3 (239.59 to 282.72)	843.71 (771.36 to 926.26)	138.61 (126.72 to 152.17)	-2.21 (-2.31 to -2.1)
Oceania	0.31 (0.14 to 0.49)	4.87 (2.15 to 7.57)	0.61 (0.26 to 1.02)	4.6 (1.95 to 7.69)	-0.12 (-0.2 to -0.03)
South Asia	3217.47 (2690.66 to 3833.2)	293.13 (245.13 to 349.23)	3184.15 (2666.87 to 3774.71)	176.39 (147.73 to 209.1)	-1.8 (-1.98 to -1.62)
Southeast Asia	67.6 (57.73 to 79.23)	14.48 (12.37 to 16.97)	58.63 (50.1 to 68.68)	8.7 (7.44 to 10.19)	-1.65 (-1.71 to -1.59)
Southern Latin America	1.95 (1.1 to 2.95)	3.94 (2.22 to 5.95)	1.84 (1.06 to 2.75)	2.76 (1.58 to 4.12)	-0.95 (-1.09 to -0.81)
Southern Sub-Saharan Africa	27.27 (23.72 to 31.4)	51.95 (45.18 to 59.81)	28.44 (24.6 to 32.88)	36.19 (31.3 to 41.85)	-1.23 (-1.3 to -1.16)
Tropical Latin America	79.62 (71.23 to 88.77)	52.08 (46.59 to 58.07)	80.92 (72.94 to 89.7)	36.19 (32.62 to 40.12)	-1.1 (-1.18 to -1.02)
Western Europe	174.09 (153.59 to 197.35)	45.27 (39.94 to 51.31)	107.63 (91.74 to 124.9)	24.67 (21.03 to 28.63)	-1.49 (-1.71 to -1.27)
Western Sub-Saharan Africa	1778.46 (1586.41 to 1983.54)	923.49 (823.77 to 1029.98)	3157.8 (2776.44 to 3575.19)	692.02 (608.45 to 783.49)	-1.03 (-1.14 to -0.92)

Abbreviations: EAPC, estimated annual percentage change; UI, uncertainty interval. CI, confidence interval.

Supplementary Table S5. The incident cases and incidence rates of G6PD deficiency in 1990 and 2019, along with their temporal trend.

Characteristics	1990		2019		1990-2019
	Incident cases No.×10^3(95% UI)	Incidence rates per 100000 (95% UI)	Incident cases No.×10^3(95% UI)	Incidence rates per 100000 (95% UI)	EAPC (95%CI)
**Global**	7484.69 (6819.44 to 8244.42)	139.9 (127.47 to 154.11)	8956.94 (8050.43 to 10025.58)	115.76 (104.04 to 129.57)	-0.86 (-1.03 to -0.68)
**Sex**					
Male	5432.72 (4961.98 to 5966.03)	201.68 (184.2 to 221.48)	6531.66 (5873.55 to 7257.53)	168.3 (151.34 to 187)	-0.86 (-0.99 to -0.73)
Female	2051.97 (1854.5 to 2285.62)	77.26 (69.82 to 86.05)	2425.28 (2169.68 to 2729.62)	62.89 (56.26 to 70.78)	-0.83 (-1.16 to -0.5)
**Region**					
Andean Latin America	18.85 (14.68 to 23.81)	49.38 (38.45 to 62.38)	50.98 (39.97 to 64.12)	80.17 (62.85 to 100.82)	-0.67 (-1.37 to 0.03)
Australasia	1.23 (0.96 to 1.55)	6.09 (4.73 to 7.65)	3.12 (2.42 to 3.87)	10.74 (8.32 to 13.31)	2.27 (1.44 to 3.11)
Caribbean	82.31 (63.87 to 101.95)	233.36 (181.07 to 289.03)	83.9 (64.94 to 103.79)	177.87 (137.67 to 220.04)	-0.92 (-0.97 to -0.86)
Central Asia	27.02 (20.88 to 34.07)	39.02 (30.14 to 49.19)	26.68 (20.67 to 33.79)	28.52 (22.1 to 36.13)	-0.34 (-0.69 to 0.01)
Central Europe	55.25 (46.83 to 65.12)	44.93 (38.09 to 52.95)	40.29 (34.19 to 47.42)	35.27 (29.93 to 41.51)	-1.39 (-1.78 to -1)
Central Latin America	157.78 (134.53 to 185.49)	96.14 (81.97 to 113.02)	104.85 (86.4 to 125.29)	41.93 (34.56 to 50.11)	-2.81 (-2.98 to -2.63)
Central Sub-Saharan Africa	356.1 (276.15 to 442.58)	641.4 (497.38 to 797.15)	593.09 (460.02 to 740.72)	450.87 (349.71 to 563.09)	-1.19 (-1.33 to -1.04)
East Asia	879.28 (844.9 to 914.01)	71.77 (68.96 to 74.61)	742.31 (707.82 to 775.12)	50.42 (48.08 to 52.65)	-0.98 (-1.17 to -0.8)

Eastern Europe	55.85 (52.93 to 58.7)	24.66 (23.37 to 25.92)	46.82 (44.52 to 49.1)	22.3 (21.2 to 23.38)	0.76 (0.28 to 1.23)
Eastern Sub-Saharan Africa	924.64 (800.78 to 1066.89)	486.22 (421.09 to 561.03)	1228.27 (1025.02 to 1453.69)	298.28 (248.93 to 353.03)	-1.62 (-1.86 to -1.37)
High-income Asia Pacific	8.07 (7.02 to 9.27)	4.65 (4.05 to 5.34)	5.78 (5.12 to 6.49)	3.08 (2.73 to 3.47)	-2.02 (-2.25 to -1.78)
High-income North America	231.31 (221.2 to 241)	82.34 (78.74 to 85.79)	178.51 (169.73 to 186.98)	48.97 (46.56 to 51.29)	-1.87 (-2.79 to -0.93)
North Africa and Middle East	689.15 (605.74 to 789.89)	199.74 (175.56 to 228.94)	662.38 (532.51 to 814)	108.82 (87.48 to 133.72)	-1.59 (-1.73 to -1.44)
Oceania	13.81 (12.53 to 15.28)	213.5 (193.61 to 236.16)	23.65 (18.28 to 29.57)	178.15 (137.72 to 222.74)	-0.59 (-0.77 to -0.42)
South Asia	1788.98 (1672.95 to 1917.76)	162.99 (152.42 to 174.72)	2450.22 (2330.59 to 2571.13)	135.73 (129.1 to 142.43)	-1.91 (-2.54 to -1.27)
Southeast Asia	755.15 (678.87 to 837.98)	161.77 (145.43 to 179.52)	486.8 (425.85 to 555.55)	72.25 (63.2 to 82.45)	-2.37 (-2.6 to -2.13)
Southern Latin America	16.21 (12.5 to 20.19)	32.71 (25.23 to 40.75)	14.36 (11.08 to 18.27)	21.51 (16.6 to 27.36)	-1.29 (-1.46 to -1.13)
Southern Sub-Saharan Africa	139.66 (126.31 to 154.22)	266.05 (240.62 to 293.8)	140.31 (126.64 to 154.74)	178.57 (161.17 to 196.94)	-1.17 (-1.32 to -1.03)
Tropical Latin America	94.73 (91.17 to 98.22)	61.97 (59.63 to 64.24)	110.09 (105.7 to 114.41)	49.24 (47.27 to 51.17)	0.08 (-0.43 to 0.6)
Western Europe	114.68 (99.16 to 132.11)	29.82 (25.78 to 34.35)	117.38 (101.36 to 135.77)	26.9 (23.23 to 31.12)	-0.39 (-0.62 to -0.15)
Western Sub-Saharan Africa	1074.62 (994.62 to 1164.8)	558.01 (516.47 to 604.84)	1847.16 (1666.24 to 2043.51)	404.8 (365.15 to 447.83)	-0.94 (-1.03 to -0.85)

Abbreviations: EAPC, estimated annual percentage change; UI, uncertainty interval. CI, confidence interval.

Supplementary Table S6. The incident cases and incidence rates of G6PD trait in 1990 and 2019, along with their temporal trend.

Characteristics	1990		2019		1990-2019
	Incident cases No.×10³(95% UI)	Incidence rates per 100000 (95% UI)	Incident cases No.×10³(95% UI)	Incidence rates per 100000 (95% UI)	EAPC (95%CI)
**Global**	17715.84 (17107 to 18350.29)	331.15 (319.77 to 343.01)	18731.78 (17994.33 to 19540.11)	242.09 (232.56 to 252.54)	-1.09 (-1.2 to -0.99)
**Sex**					
Male	-	-	-	-	-
Female	17715.84 (17107 to 18350.29)	666.99 (644.07 to 690.87)	18731.78 (17994.33 to 19540.11)	485.72 (466.6 to 506.68)	-1.11 (-1.22 to -1.01)
**Region**					
Andean Latin America	142.97 (128.91 to 157.47)	374.48 (337.67 to 412.47)	150.82 (135.61 to 166.57)	237.16 (213.23 to 261.92)	-1.52 (-1.56 to -1.48)
Australasia	13.68 (12.07 to 15.47)	67.47 (59.5 to 76.31)	10.75 (9.04 to 12.42)	36.98 (31.11 to 42.72)	-1.86 (-2.32 to -1.4)
Caribbean	80.23 (71.83 to 89.37)	227.46 (203.65 to 253.36)	68.44 (61.11 to 76.48)	145.1 (129.55 to 162.15)	-1.5 (-1.6 to -1.41)
Central Asia	153.1 (136.23 to 170.56)	221.03 (196.67 to 246.24)	145.32 (129.31 to 161.92)	155.37 (138.25 to 173.12)	-0.53 (-0.87 to -0.19)
Central Europe	121.72 (112.51 to 131.68)	98.99 (91.49 to 107.09)	77.99 (72.05 to 84.2)	68.28 (63.07 to 73.71)	-0.75 (-1.01 to -0.49)
Central Latin America	700.78 (667.16 to 735.59)	426.98 (406.5 to 448.19)	511.95 (481.29 to 543.18)	204.76 (192.5 to 217.25)	-2.59 (-2.74 to -2.43)
Central Sub-Saharan Africa	535.71 (494.97 to 573.38)	964.91 (891.52 to 1032.74)	887.19 (817.8 to 948.45)	674.44 (621.69 to 721.01)	-1.13 (-1.28 to -0.98)
East Asia	2464.26 (2419.84 to 2505.21)	201.14 (197.52 to 204.48)	1661.29 (1629.32 to 1691.57)	112.84 (110.67 to 114.9)	-2.29 (-2.48 to -2.09)

Eastern Europe	217.63 (211.93 to 222.92)	96.08 (93.57 to 98.42)	169.86 (165.68 to 173.7)	80.9 (78.9 to 82.72)	0.89 (0.39 to 1.39)
Eastern Sub-Saharan Africa	1705.33 (1622.09 to 1785.98)	896.75 (852.99 to 939.17)	2561.51 (2428.82 to 2695.75)	622.06 (589.84 to 654.66)	-1.29 (-1.41 to -1.16)
High-income Asia Pacific	97.43 (91.49 to 103.51)	56.15 (52.73 to 59.66)	60.19 (56.69 to 63.95)	32.14 (30.27 to 34.15)	-2.02 (-2.14 to -1.91)
High-income North America	475.31 (465.69 to 485.13)	169.2 (165.77 to 172.69)	449.23 (439.45 to 458.2)	123.23 (120.54 to 125.69)	-0.91 (-1.06 to -0.75)
North Africa and Middle East	1790.5 (1682.72 to 1899.05)	518.94 (487.71 to 550.4)	1829.64 (1684.07 to 1972.42)	300.57 (276.66 to 324.03)	-1.77 (-1.81 to -1.73)
Oceania	27.29 (24.54 to 29.98)	421.83 (379.26 to 463.38)	61.6 (55.79 to 67.52)	463.96 (420.23 to 508.55)	0.01 (-0.19 to 0.22)
South Asia	4617.95 (4527.5 to 4717.5)	420.72 (412.48 to 429.79)	4923.01 (4831.32 to 5012.66)	272.71 (267.63 to 277.68)	-1.83 (-2.13 to -1.53)
Southeast Asia	1704 (1627.07 to 1781.8)	365.05 (348.57 to 381.71)	1258.5 (1201.71 to 1317.7)	186.78 (178.35 to 195.57)	-2.14 (-2.29 to -1.99)
Southern Latin America	80.8 (71.79 to 90.45)	163.1 (144.92 to 182.58)	67.82 (60.01 to 76.25)	101.6 (89.9 to 114.23)	-1.39 (-1.58 to -1.2)
Southern Sub-Saharan Africa	272.49 (263.39 to 281.79)	519.1 (501.76 to 536.81)	287.73 (277.45 to 297.64)	366.19 (353.1 to 378.79)	-0.99 (-1.08 to -0.89)
Tropical Latin America	407.88 (400.11 to 415.23)	266.8 (261.71 to 271.6)	377.95 (370.96 to 384.64)	169.03 (165.91 to 172.03)	-1.15 (-1.38 to -0.93)
Western Europe	509.43 (477.26 to 543.5)	132.46 (124.1 to 141.32)	457 (427.34 to 488.02)	104.74 (97.94 to 111.85)	-0.65 (-0.74 to -0.56)
Western Sub-Saharan Africa	1597.33 (1537.16 to 1658.99)	829.44 (798.19 to 861.45)	2714 (2586.26 to 2839.77)	594.77 (566.77 to 622.33)	-0.9 (-1.04 to -0.77)

G6PD deficiency is inherited in an X-linked pattern, data on G6PD trait was not collected.

Abbreviations: EAPC, estimated annual percentage change; UI, uncertainty interval. CI, confidence interval. G6PD, glucose-6-phosphate dehydrogenase.

Supplementary Table S7. The change of inherited anemias cases and incidence rates between 1990 and 2019 at national level, both sexes.

Regions	Causes	Cases in 1990 No.×103 (95% UI)	Cases in 2019 No.×103 (95% UI)	Change in absolute number (%)	Rates in 1990 per 100000 (95% UI)	Rates in 2019 per 100000 (95% UI)	EAPC from 1990 and 2019 (95%CI)
Afghanistan	Inherited anemias	148 (136 to 161)	427 (392 to 464)	1.88 (1.83 to 1.95)	1298.78 (1191.73 to 1413.21)	1116.16 (1024.25 to 1212.88)	-1.19 (-1.4 to -0.97)
Afghanistan	Thalassemias	0 (0 to 1)	1 (1 to 2)	1.99 (1.7 to 2.32)	3.89 (3 to 5.14)	3.47 (2.68 to 4.55)	-0.93 (-1.09 to -0.77)
Afghanistan	Thalassemias trait	32 (28 to 37)	95 (84 to 108)	1.97 (1.84 to 2.12)	280.25 (248.44 to 320.42)	248.61 (220.26 to 282.42)	-0.93 (-1.09 to -0.77)
Afghanistan	Sickle cell disorders	1 (1 to 1)	2 (2 to 3)	1.85 (1.62 to 2.14)	7.27 (5.51 to 9.44)	6.18 (4.59 to 8.01)	-1.12 (-1.29 to -0.94)
Afghanistan	Sickle cell trait	40 (35 to 45)	113 (99 to 129)	1.84 (1.73 to 1.98)	349.59 (306.49 to 397.25)	296.28 (257.44 to 336.68)	-1.09 (-1.25 to -0.93)
Afghanistan	G6PD deficiency	19 (15 to 24)	54 (41 to 67)	1.85 (1.63 to 2.07)	164.59 (126.99 to 207.03)	140.12 (108.36 to 175.13)	-1.41 (-1.7 to -1.12)
Afghanistan	G6PD trait	56 (50 to 62)	161 (144 to 177)	1.86 (1.77 to 1.97)	493.17 (440.43 to 542.65)	421.49 (376.94 to 463.22)	-1.35 (-1.64 to -1.05)
Albania	Inherited anemias	17 (15 to 18)	7 (6 to 8)	-0.57 (-0.57 to -0.56)	499.45 (450.37 to 553.6)	263.14 (237.45 to 291.41)	-2.59 (-2.9 to -2.28)
Albania	Thalassemias	0 (0 to 0)	0 (0 to 0)	-0.69 (-0.72 to -0.65)	4.19 (3.04 to 5.96)	1.58 (1.2 to 2.12)	-3.51 (-3.85 to -3.18)
Albania	Thalassemias trait	4 (4 to 5)	2 (2 to 2)	-0.57 (-0.59 to -0.55)	131.04 (112.02 to 152.1)	68.47 (59.26 to 78.6)	-2.45 (-2.78 to -2.11)
Albania	Sickle cell disorders	0 (0 to 0)	0 (0 to 0)	-0.51 (-0.55 to -0.46)	0.53 (0.41 to 0.69)	0.32 (0.24 to 0.4)	-2.16 (-2.49 to -1.82)
Albania	Sickle cell trait	2 (2 to 3)	1 (1 to 1)	-0.55 (-0.57 to -0.52)	72.68 (63.4 to 83.14)	40.12 (35.09 to 45.49)	-2.33 (-2.65 to -2.01)
Albania	G6PD deficiency	3 (2 to 4)	1 (1 to 2)	-0.54 (-0.54 to -0.54)	96.21 (74.26 to 120.53)	53.99 (41.68 to 67.65)	-3.17 (-3.52 to -2.81)

Regions	Causes	Cases in 1990 No.×103 (95% UI)	Cases in 2019 No.×103 (95% UI)	Change in absolute number (%)	Rates in 1990 per 100000 (95% UI)	Rates in 2019 per 100000 (95% UI)	EAPC from 1990 and 2019 (95%CI)
Albania	G6PD trait	6 (6 to 7)	3 (2 to 3)	−0.58 (−0.58 to −0.58)	194.8 (172.06 to 218.68)	98.66 (87.15 to 110.76)	−2.53 (−2.87 to −2.19)
Algeria	Inherited anemias	218 (194 to 245)	247 (220 to 276)	0.13 (0.11 to 0.16)	863.16 (765.86 to 967.67)	590.38 (524.71 to 660.55)	−0.8 (−1.02 to −0.58)
Algeria	Thalassemias	0 (0 to 0)	0 (0 to 1)	0.24 (0.13 to 0.36)	1.37 (1.06 to 1.77)	1.02 (0.8 to 1.3)	−0.51 (−0.74 to −0.28)
Algeria	Thalassemias trait	35 (31 to 40)	42 (38 to 48)	0.22 (0.16 to 0.27)	137.45 (121.98 to 156.37)	100.96 (89.67 to 113.65)	−0.56 (−0.78 to −0.33)
Algeria	Sickle cell disorders	0 (0 to 0)	0 (0 to 0)	0.17 (0.1 to 0.23)	0.77 (0.64 to 0.92)	0.54 (0.46 to 0.64)	−0.71 (−0.95 to −0.47)
Algeria	Sickle cell trait	23 (21 to 25)	27 (24 to 29)	0.16 (0.13 to 0.19)	91.41 (83.26 to 100.18)	64.01 (58.43 to 69.75)	−0.71 (−0.95 to −0.48)
Algeria	G6PD deficiency	68 (52 to 84)	75 (57 to 92)	0.1 (0.01 to 0.18)	268.12 (207.27 to 333)	178.12 (137.22 to 219.39)	−0.93 (−1.14 to −0.71)
Algeria	G6PD trait	92 (83 to 102)	103 (92 to 114)	0.12 (0.1 to 0.15)	364.03 (327.58 to 401.7)	245.71 (220.9 to 271.52)	−0.82 (−1.04 to −0.59)
American Samoa	Inherited anemias	0 (0 to 0)	0 (0 to 0)	−0.33 (−0.35 to −0.3)	751.12 (668.13 to 839.8)	442.37 (391.95 to 495.8)	−2.01 (−2.21 to −1.8)
American Samoa	Thalassemias	0 (0 to 0)	0 (0 to 0)	−0.46 (−0.53 to −0.38)	3.45 (1.87 to 5.33)	1.64 (0.88 to 2.57)	−1.62 (−2.57 to −0.66)
American Samoa	Thalassemias trait	0 (0 to 0)	0 (0 to 0)	−0.41 (−0.45 to −0.37)	112.74 (86.59 to 138.13)	57.99 (44.21 to 71.29)	−2.04 (−2.41 to −1.65)
American Samoa	Sickle cell disorders	0 (0 to 0)	0 (0 to 0)	−0.37 (−0.4 to −0.03)	0 (0 to 0)	0 (0 to 0)	−2.28 (−2.38 to −2.19)
American Samoa	Sickle cell trait	0 (0 to 0)	0 (0 to 0)	−0.38 (−0.4 to −0.16)	4.41 (1.94 to 7.06)	2.38 (1.02 to 3.81)	−2.33 (−2.41 to −2.24)
American Samoa	G6PD deficiency	0 (0 to 0)	0 (0 to 0)	−0.26 (−0.29 to −0.24)	154.65 (119.52 to 192.81)	99.36 (76.79 to 123.49)	−1.79 (−2.05 to −1.52)
American	G6PD trait	0	0	−0.32	475.87	281	−2.09

Regions	Causes	Cases in 1990 No.×103 (95% UI)	Cases in 2019 No.×103 (95% UI)	Change in absolute number (%)	Rates in 1990 per 100000 (95% UI)	Rates in 2019 per 100000 (95% UI)	EAPC from 1990 and 2019 (95%CI)
Samoa		(0 to 0)	(0 to 0)	(-0.35 to -0.3)	(428.14 to 524.86)	(254.05 to 308.17)	(-2.29 to -1.89)
Andorra	Inherited anemias	0 (0 to 0)	0 (0 to 0)	0 (-0.03 to 0.03)	204.27 (183.16 to 226.19)	132.66 (118.72 to 147.75)	-1.62 (-1.85 to -1.39)
Andorra	Thalassemias	0 (0 to 0)	0 (0 to 0)	-0.07 (-0.2 to 0.06)	0.14 (0.09 to 0.19)	0.08 (0.06 to 0.12)	-2.69 (-3.55 to -1.83)
Andorra	Thalassemias trait	0 (0 to 0)	0 (0 to 0)	0.02 (-0.04 to 0.09)	17.64 (15.16 to 20.17)	11.71 (10.1 to 13.43)	-1.75 (-2.08 to -1.42)
Andorra	Sickle cell disorders	0 (0 to 0)	0 (0 to 0)	-0.04 (-0.13 to 0.04)	0.16 (0.12 to 0.21)	0.1 (0.07 to 0.13)	-1.79 (-2.09 to -1.49)
Andorra	Sickle cell trait	0 (0 to 0)	0 (0 to 0)	-0.03 (-0.09 to 0.03)	21.48 (17.85 to 25.74)	13.53 (11.26 to 16.07)	-1.73 (-2 to -1.46)
Andorra	G6PD deficiency	0 (0 to 0)	0 (0 to 0)	0.05 (0.01 to 0.11)	32.26 (25.06 to 39.91)	22.02 (16.94 to 27.64)	-1.46 (-1.64 to -1.28)
Andorra	G6PD trait	0 (0 to 0)	0 (0 to 0)	-0.01 (-0.05 to 0.03)	132.59 (118.85 to 146.78)	85.22 (76.27 to 94.26)	-1.62 (-1.85 to -1.4)
Angola	Inherited anemias	269 (245 to 296)	591 (540 to 646)	1.2 (1.12 to 1.28)	2608.52 (2372.45 to 2865.32)	1961.97 (1791.19 to 2143.92)	-0.93 (-1.1 to -0.76)
Angola	Thalassemias	0 (0 to 0)	0 (0 to 0)	3.46 (2.86 to 4.3)	0.12 (0.07 to 0.17)	0.18 (0.1 to 0.26)	1.26 (1.03 to 1.49)
Angola	Thalassemias trait	4 (3 to 5)	11 (9 to 13)	1.74 (1.52 to 1.99)	39.6 (33.21 to 46.77)	37.18 (30.51 to 44.6)	-0.28 (-0.45 to -0.1)
Angola	Sickle cell disorders	6 (5 to 8)	14 (11 to 18)	1.33 (1.03 to 1.66)	59.52 (46.53 to 78.1)	47.41 (38.04 to 60.65)	-0.6 (-0.81 to -0.39)
Angola	Sickle cell trait	99 (89 to 111)	220 (200 to 246)	1.23 (1.1 to 1.37)	956.55 (857.78 to 1080.03)	731.37 (664.03 to 816.78)	-0.82 (-1 to -0.65)
Angola	G6PD deficiency	59 (46 to 74)	129 (99 to 159)	1.16 (0.97 to 1.37)	576.42 (445.2 to 719.71)	426.64 (328.08 to 529.14)	-1.04 (-1.21 to -0.88)
Angola	G6PD trait	101 (93 to 108)	217 (199 to	1.15 (1.09 to	976.3 (899.35 to	719.2 (661.94 to	-1.01 (-1.18 to

Regions	Causes	Cases in 1990 No.×103 (95% UI)	Cases in 2019 No.×103 (95% UI)	Change in absolute number (%)	Rates in 1990 per 100000 (95% UI)	Rates in 2019 per 100000 (95% UI)	EAPC from 1990 and 2019 (95%CI)
			232)	1.22)	1043.82)	768.42)	-0.85)
Antigua and Barbuda	Inherited anemias	0 (0 to 0)	0 (0 to 0)	-0.24 (-0.27 to -0.22)	557.97 (491.56 to 629.71)	289.11 (254.81 to 326.1)	-2.61 (-2.74 to -2.49)
Antigua and Barbuda	Thalassemias	0 (0 to 0)	0 (0 to 0)	0.2 (-0.26 to 1.5)	0.01 (0.01 to 0.02)	0.01 (0 to 0.01)	-1.14 (-1.44 to -0.84)
Antigua and Barbuda	Thalassemias trait	0 (0 to 0)	0 (0 to 0)	-0.13 (-0.26 to 0.13)	7.78 (5.89 to 9.93)	4.64 (3.62 to 5.7)	-2.16 (-2.32 to -2)
Antigua and Barbuda	Sickle cell disorders	0 (0 to 0)	0 (0 to 0)	-0.15 (-0.24 to -0.05)	4.46 (3.26 to 6)	2.6 (1.87 to 3.53)	-2.29 (-2.54 to -2.03)
Antigua and Barbuda	Sickle cell trait	0 (0 to 0)	0 (0 to 0)	-0.22 (-0.27 to -0.17)	162.74 (136.32 to 192.69)	87.34 (72.17 to 104.23)	-2.49 (-2.65 to -2.34)
Antigua and Barbuda	G6PD deficiency	0 (0 to 0)	0 (0 to 0)	-0.26 (-0.32 to -0.2)	173.97 (134.12 to 214.74)	87.83 (67.85 to 108.62)	-2.69 (-2.81 to -2.57)
Antigua and Barbuda	G6PD trait	0 (0 to 0)	0 (0 to 0)	-0.26 (-0.28 to -0.23)	209.01 (186.51 to 233.16)	106.68 (95.02 to 118.34)	-2.67 (-2.8 to -2.54)
Argentina	Inherited anemias	69 (60 to 78)	66 (58 to 75)	-0.04 (-0.08 to 0.01)	207.12 (181.09 to 234.73)	146.26 (128.69 to 165.88)	-0.94 (-1.15 to -0.73)
Argentina	Thalassemias	0 (0 to 0)	0 (0 to 0)	-0.05 (-0.24 to 0.25)	0.27 (0.17 to 0.42)	0.19 (0.13 to 0.27)	-1.01 (-1.28 to -0.74)
Argentina	Thalassemias trait	3 (2 to 3)	5 (5 to 6)	0.99 (0.69 to 1.37)	8.21 (6.91 to 9.68)	12 (10.11 to 13.96)	1.95 (1.56 to 2.33)
Argentina	Sickle cell disorders	0 (0 to 0)	0 (0 to 0)	0.03 (0.03 to 0.03)	0 (0 to 0.01)	0 (0 to 0)	-0.71 (-0.85 to -0.57)
Argentina	Sickle cell trait	1 (1 to 2)	1 (1 to 2)	0.03 (0.03 to 0.03)	3.91 (2.2 to 5.95)	2.96 (1.69 to 4.49)	-0.71 (-0.85 to -0.56)
Argentina	G6PD deficiency	11 (8 to 13)	10 (8 to 13)	-0.04 (-0.09 to 0.03)	32.55 (25 to 40.43)	22.91 (17.7 to 29.14)	-1.04 (-1.24 to -0.83)
Argentina	G6PD trait	54 (48 to 60)	49 (43 to 55)	-0.09 (-0.13 to -0.04)	162.18 (143.79 to 181.56)	108.21 (95.73 to 121.64)	-1.13 (-1.37 to -0.9)

Regions	Causes	Cases in 1990 No.×103 (95% UI)	Cases in 2019 No.×103 (95% UI)	Change in absolute number (%)	Rates in 1990 per 100000 (95% UI)	Rates in 2019 per 100000 (95% UI)	EAPC from 1990 and 2019 (95%CI)
Armenia	Inherited anemias	11 (10 to 13)	5 (5 to 6)	−0.52 (−0.53 to −0.51)	330.66 (295.8 to 368.12)	178.23 (159.35 to 198.49)	-1.01 (-1.55 to -0.47)
Armenia	Thalassemias	0 (0 to 0)	0 (0 to 0)	−0.61 (−0.66 to −0.56)	2.15 (1.52 to 3.01)	0.94 (0.67 to 1.31)	-1.76 (-2.27 to -1.25)
Armenia	Thalassemias trait	4 (3 to 4)	2 (1 to 2)	−0.51 (−0.54 to −0.48)	105.14 (86.55 to 130.86)	58.08 (48.06 to 70.81)	-0.99 (-1.49 to -0.48)
Armenia	Sickle cell disorders	0 (0 to 0)	0 (0 to 0)	−0.52 (−0.62 to −0.4)	0.01 (0.01 to 0.02)	0.01 (0 to 0.01)	-0.97 (-1.49 to -0.44)
Armenia	Sickle cell trait	0 (0 to 0)	0 (0 to 0)	−0.52 (−0.57 to −0.45)	10.46 (8.3 to 13.07)	5.72 (4.51 to 7.16)	-0.98 (-1.5 to -0.45)
Armenia	G6PD deficiency	1 (1 to 1)	1 (0 to 1)	−0.5 (−0.54 to −0.46)	32.46 (24.99 to 41.15)	18.23 (14.14 to 23.18)	-0.83 (-1.36 to -0.3)
Armenia	G6PD trait	6 (5 to 7)	3 (3 to 3)	−0.53 (−0.53 to −0.53)	180.42 (160.53 to 202.04)	95.25 (84.75 to 106.66)	-1.06 (-1.62 to -0.5)
Australia	Inherited anemias	18 (16 to 20)	17 (15 to 20)	−0.01 (−0.08 to 0.04)	104.74 (92.97 to 117.38)	70.99 (61.29 to 81.19)	-1.17 (-1.45 to -0.89)
Australia	Thalassemias	0 (0 to 0)	0 (0 to 0)	0.14 (−0.01 to 0.31)	0.75 (0.49 to 1.18)	0.59 (0.39 to 0.9)	-0.69 (-0.85 to -0.53)
Australia	Thalassemias trait	3 (2 to 3)	3 (3 to 4)	0.26 (0.17 to 0.38)	15.27 (13.41 to 17.41)	13.24 (11.69 to 14.92)	-0.44 (-0.55 to -0.32)
Australia	Sickle cell disorders	0 (0 to 0)	0 (0 to 0)	0.27 (0.12 to 0.6)	0.02 (0.01 to 0.02)	0.01 (0.01 to 0.02)	-0.3 (-0.46 to -0.15)
Australia	Sickle cell trait	2 (1 to 2)	2 (2 to 2)	0.24 (0.16 to 0.41)	9.28 (7.8 to 11.03)	7.92 (6.65 to 9.39)	-0.34 (-0.51 to -0.17)
Australia	G6PD deficiency	1 (1 to 1)	3 (2 to 4)	1.64 (1.38 to 1.99)	6.8 (5.16 to 8.67)	12.3 (9.47 to 15.32)	2.38 (1.52 to 3.24)
Australia	G6PD trait	12 (11 to 14)	9 (7 to 11)	−0.26 (−0.35 to −0.2)	72.63 (63.28 to 82.94)	36.94 (30.12 to 43.6)	-2.11 (-2.65 to -1.57)
Austria	Inherited	16	16	−0.04	207.21	174.06	-0.71

Regions	Causes	Cases in 1990 No.×103 (95% UI)	Cases in 2019 No.×103 (95% UI)	Change in absolute number (%)	Rates in 1990 per 100000 (95% UI)	Rates in 2019 per 100000 (95% UI)	EAPC from 1990 and 2019 (95%CI)
	**anemias**	(14 to 18)	(14 to 17)	(-0.07 to 0)	(185.61 to 230.27)	(156.23 to 193.79)	(-0.96 to -0.46)
Austria	Thalassemias	0 (0 to 0)	0 (0 to 0)	-0.13 (-0.25 to 0.01)	0.16 (0.11 to 0.21)	0.12 (0.08 to 0.17)	-1.94 (-2.65 to -1.22)
Austria	Thalassemias trait	2 (1 to 2)	1 (1 to 2)	-0.05 (-0.11 to 0.01)	19.44 (16.82 to 22.27)	16.09 (13.96 to 18.46)	-1 (-1.24 to -0.75)
Austria	Sickle cell disorders	0 (0 to 0)	0 (0 to 0)	0.76 (0.56 to 1)	0.07 (0.05 to 0.08)	0.1 (0.08 to 0.13)	1.99 (1.6 to 2.39)
Austria	Sickle cell trait	1 (1 to 1)	2 (1 to 2)	0.32 (0.24 to 0.41)	16.25 (14.31 to 18.51)	18.72 (16.59 to 21.1)	0.74 (0.45 to 1.03)
Austria	G6PD deficiency	3 (2 to 3)	3 (2 to 3)	-0.02 (-0.07 to 0.03)	33.33 (25.68 to 41.52)	28.53 (21.87 to 36.04)	-0.67 (-0.97 to -0.38)
Austria	G6PD trait	11 (10 to 12)	10 (9 to 11)	-0.08 (-0.12 to -0.04)	137.97 (123.66 to 152.8)	110.49 (98.81 to 122.12)	-0.87 (-1.11 to -0.62)
Azerbaijan	**Inherited anemias**	31 (28 to 34)	23 (21 to 25)	-0.25 (-0.27 to -0.24)	420.15 (380.93 to 460.11)	223.74 (202.92 to 245.65)	-1.21 (-1.62 to -0.8)
Azerbaijan	Thalassemias	0 (0 to 0)	0 (0 to 0)	-0.38 (-0.44 to -0.32)	3.56 (2.7 to 4.68)	1.56 (1.21 to 2.01)	-1.88 (-2.28 to -1.48)
Azerbaijan	Thalassemias trait	11 (10 to 13)	8 (7 to 10)	-0.23 (-0.27 to -0.19)	150.16 (130.64 to 170.8)	82.58 (72.24 to 93.3)	-1.14 (-1.53 to -0.74)
Azerbaijan	Sickle cell disorders	0 (0 to 0)	0 (0 to 0)	-0.26 (-0.38 to -0.11)	0.02 (0.01 to 0.03)	0.01 (0.01 to 0.01)	-1.24 (-1.64 to -0.83)
Azerbaijan	Sickle cell trait	1 (1 to 1)	1 (1 to 1)	-0.25 (-0.32 to -0.17)	13.22 (10.3 to 16.54)	7.04 (5.51 to 8.89)	-1.21 (-1.61 to -0.81)
Azerbaijan	G6PD deficiency	3 (2 to 4)	2 (2 to 3)	-0.22 (-0.27 to -0.15)	38.52 (29.64 to 48.01)	21.48 (16.65 to 27.16)	-0.99 (-1.4 to -0.58)
Azerbaijan	G6PD trait	16 (14 to 18)	11 (10 to 13)	-0.27 (-0.27 to -0.27)	214.67 (191.53 to 238.73)	111.07 (99.09 to 123.52)	-1.3 (-1.72 to -0.88)
Bahamas	**Inherited anemias**	1 (1 to 2)	1 (1 to 1)	-0.17 (-0.2 to	549.95 (486.36 to	308.94 (271.94 to	-2.66 (-2.89 to

Regions	Causes	Cases in 1990 No.×103 (95% UI)	Cases in 2019 No.×103 (95% UI)	Change in absolute number (%)	Rates in 1990 per 100000 (95% UI)	Rates in 2019 per 100000 (95% UI)	EAPC from 1990 and 2019 (95%CI)
				-0.14)	619.12)	348.52)	-2.42)
Bahamas	Thalassemias	0 (0 to 0)	0 (0 to 0)	0.28 (-0.23 to 1.57)	0.01 (0.01 to 0.02)	0.01 (0 to 0.01)	-1.19 (-1.48 to -0.89)
Bahamas	Thalassemias trait	0 (0 to 0)	0 (0 to 0)	-0.08 (-0.23 to 0.18)	7.37 (5.64 to 9.32)	4.62 (3.59 to 5.62)	-2.23 (-2.43 to -2.04)
Bahamas	Sickle cell disorders	0 (0 to 0)	0 (0 to 0)	0.05 (-0.05 to 0.18)	7.23 (5.36 to 9.36)	5.18 (3.77 to 6.65)	-2.19 (-2.77 to -1.59)
Bahamas	Sickle cell trait	0 (0 to 1)	0 (0 to 1)	-0.09 (-0.14 to -0.04)	187.1 (159.25 to 216.37)	115.25 (97.98 to 133.97)	-2.5 (-2.88 to -2.11)
Bahamas	G6PD deficiency	0 (0 to 1)	0 (0 to 0)	-0.23 (-0.28 to -0.16)	156.46 (120.84 to 196.87)	81.82 (62.79 to 102.5)	-2.81 (-3 to -2.61)
Bahamas	G6PD trait	0 (0 to 1)	0 (0 to 0)	-0.22 (-0.25 to -0.2)	191.77 (170.95 to 214.18)	102.06 (90.8 to 113.89)	-2.75 (-2.94 to -2.55)
Bahrain	**Inherited anemias**	9 (8 to 10)	8 (7 to 9)	-0.09 (-0.13 to -0.06)	1751.44 (1612.79 to 1892.7)	559.89 (511.7 to 613.55)	-4.3 (-4.79 to -3.81)
Bahrain	Thalassemias	0 (0 to 0)	0 (0 to 0)	-0.01 (-0.12 to 0.1)	1.92 (1.34 to 2.75)	0.67 (0.46 to 0.95)	-3.7 (-3.8 to -3.6)
Bahrain	Thalassemias trait	1 (1 to 1)	1 (1 to 1)	-0.02 (-0.07 to 0.03)	150.2 (127.19 to 177.26)	52.05 (43.96 to 61.77)	-3.71 (-3.81 to -3.6)
Bahrain	Sickle cell disorders	0 (0 to 0)	0 (0 to 0)	-0.2 (-0.28 to -0.12)	30.36 (24.65 to 37.56)	8.58 (6.84 to 10.85)	-5.15 (-5.54 to -4.77)
Bahrain	Sickle cell trait	3 (2 to 3)	2 (2 to 3)	-0.12 (-0.16 to -0.08)	512.6 (467.9 to 563.44)	158.3 (143.23 to 176.05)	-4.42 (-4.6 to -4.24)
Bahrain	G6PD deficiency	2 (2 to 3)	2 (2 to 3)	-0.12 (-0.22 to -0.03)	454.78 (360.81 to 552.72)	140.73 (108.75 to 176.82)	-4.71 (-5.94 to -3.46)
Bahrain	G6PD trait	3 (3 to 3)	3 (3 to 3)	-0.06 (-0.08 to -0.03)	601.57 (565.91 to 630.99)	199.57 (187.41 to 209.32)	-3.93 (-4.2 to -3.66)
Bangladesh	**Inherited anemias**	1342 (1200 to 1501)	979 (878 to 1087)	-0.27 (-0.29 to -0.25)	1230.71 (1100.11 to 1376.68)	614.81 (551.38 to 682.46)	-2.34 (-2.37 to -2.32)

Regions	Causes	Cases in 1990 No.×103 (95% UI)	Cases in 2019 No.×103 (95% UI)	Change in absolute number (%)	Rates in 1990 per 100000 (95% UI)	Rates in 2019 per 100000 (95% UI)	EAPC from 1990 and 2019 (95%CI)
Bangladesh	Thalassemias	7 (6 to 9)	8 (7 to 11)	0.15 (0.06 to 0.24)	6.73 (5.49 to 8.55)	5.31 (4.26 to 6.69)	-0.78 (-0.82 to -0.75)
Bangladesh	Thalassemias trait	277 (253 to 304)	227 (208 to 246)	-0.18 (-0.21 to -0.16)	253.94 (232.42 to 278.51)	142.24 (130.52 to 154.54)	-1.98 (-2.01 to -1.95)
Bangladesh	Sickle cell disorders	0 (0 to 0)	0 (0 to 0)	-0.32 (-0.43 to -0.23)	0.06 (0.04 to 0.09)	0.03 (0.02 to 0.04)	-2.69 (-2.76 to -2.62)
Bangladesh	Sickle cell trait	29 (24 to 36)	20 (16 to 25)	-0.31 (-0.38 to -0.27)	26.73 (21.59 to 33.43)	12.6 (10.11 to 15.82)	-2.61 (-2.66 to -2.56)
Bangladesh	G6PD deficiency	387 (300 to 485)	268 (209 to 332)	-0.31 (-0.35 to -0.25)	354.84 (274.98 to 445.02)	168.05 (131.15 to 208.49)	-2.48 (-2.51 to -2.46)
Bangladesh	G6PD trait	642 (585 to 695)	456 (417 to 492)	-0.29 (-0.3 to -0.28)	588.4 (536.42 to 637.02)	286.58 (262.08 to 309.16)	-2.44 (-2.46 to -2.42)
Barbados	**Inherited anemias**	1 (1 to 1)	1 (1 to 1)	-0.3 (-0.33 to -0.28)	423.36 (374.89 to 479.25)	251.51 (222.79 to 285.5)	-1.83 (-1.97 to -1.69)
Barbados	Thalassemias	0 (0 to 0)	0 (0 to 0)	0.11 (-0.32 to 1.25)	0.01 (0 to 0.01)	0.01 (0 to 0.01)	-0.36 (-0.76 to 0.04)
Barbados	Thalassemias trait	0 (0 to 0)	0 (0 to 0)	-0.2 (-0.32 to 0.03)	5.75 (4.38 to 7.33)	3.93 (3.07 to 4.85)	-1.38 (-1.61 to -1.14)
Barbados	Sickle cell disorders	0 (0 to 0)	0 (0 to 0)	-0.2 (-0.29 to -0.09)	3.67 (2.7 to 4.84)	2.5 (1.77 to 3.31)	-1.49 (-1.65 to -1.33)
Barbados	Sickle cell trait	0 (0 to 0)	0 (0 to 0)	-0.27 (-0.32 to -0.22)	125.34 (105.26 to 146.52)	77.69 (64.86 to 92.1)	-1.71 (-1.82 to -1.61)
Barbados	G6PD deficiency	0 (0 to 0)	0 (0 to 0)	-0.32 (-0.37 to -0.27)	132.11 (102 to 163.94)	76.41 (58.89 to 94.81)	-1.9 (-2.07 to -1.73)
Barbados	G6PD trait	0 (0 to 0)	0 (0 to 0)	-0.32 (-0.35 to -0.29)	156.48 (139.25 to 174.57)	90.97 (81.13 to 100.06)	-1.89 (-2.05 to -1.73)
Belarus	**Inherited anemias**	17 (15 to 19)	13 (12 to 15)	-0.25 (-0.26 to -0.23)	166.42 (147.84 to 186.05)	137.95 (122.47 to 154.84)	0.54 (0.01 to 1.06)
Belarus	Thalassemi	0	0	-0.49	0.71	0.4	-0.76

Regions	Causes	Cases in 1990 No.×103 (95% UI)	Cases in 2019 No.×103 (95% UI)	Change in absolute number (%)	Rates in 1990 per 100000 (95% UI)	Rates in 2019 per 100000 (95% UI)	EAPC from 1990 and 2019 (95%CI)
	as	(0 to 0)	(0 to 0)	(−0.55 to −0.42)	(0.5 to 1.05)	(0.29 to 0.56)	(−1.3 to −0.22)
Belarus	Thalassemias trait	3 (3 to 4)	3 (2 to 3)	−0.24 (−0.28 to −0.18)	32.88 (27.16 to 40.03)	27.72 (23 to 33.81)	0.59 (0.06 to 1.13)
Belarus	Sickle cell disorders	0 (0 to 0)	0 (0 to 0)	−0.23 (−0.38 to −0.07)	0.01 (0.01 to 0.01)	0.01 (0 to 0.01)	0.62 (0.13 to 1.12)
Belarus	Sickle cell trait	1 (1 to 1)	0 (0 to 1)	−0.24 (−0.33 to −0.17)	6.28 (5.03 to 7.72)	5.24 (4.22 to 6.45)	0.59 (0.08 to 1.1)
Belarus	G6PD deficiency	3 (2 to 3)	2 (2 to 3)	−0.21 (−0.23 to −0.19)	26.14 (20.2 to 33.16)	22.8 (17.61 to 28.41)	0.41 (−0.09 to 0.91)
Belarus	G6PD trait	11 (9 to 12)	8 (7 to 9)	−0.26 (−0.26 to −0.26)	100.4 (89.17 to 112.22)	81.79 (72.64 to 91.42)	0.55 (0.02 to 1.09)
Belgium	**Inherited anemias**	23 (21 to 26)	21 (19 to 23)	−0.11 (−0.14 to −0.08)	235.05 (211.85 to 259.22)	182.56 (162.58 to 203.15)	−0.51 (−0.66 to −0.35)
Belgium	Thalassemias	0 (0 to 0)	0 (0 to 0)	−0.2 (−0.3 to −0.1)	0.2 (0.14 to 0.27)	0.14 (0.09 to 0.2)	−1.75 (−2.41 to −1.08)
Belgium	Thalassemias trait	1 (1 to 2)	1 (1 to 2)	−0.08 (−0.18 to 0.02)	14.11 (11.96 to 16.26)	11.39 (9.57 to 13.42)	−0.71 (−0.96 to −0.45)
Belgium	Sickle cell disorders	0 (0 to 0)	0 (0 to 0)	−0.64 (−0.7 to −0.58)	0.4 (0.3 to 0.53)	0.13 (0.09 to 0.17)	−1.65 (−2.23 to −1.07)
Belgium	Sickle cell trait	4 (3 to 5)	2 (2 to 3)	−0.43 (−0.49 to −0.37)	40.93 (34.76 to 47.89)	20.5 (16.76 to 24.52)	−1.05 (−1.41 to −0.69)
Belgium	G6PD deficiency	3 (3 to 4)	3 (3 to 4)	0.01 (−0.06 to 0.05)	34.7 (26.68 to 43.34)	30.51 (23.66 to 37.91)	−0.25 (−0.38 to −0.11)
Belgium	G6PD trait	14 (13 to 16)	14 (12 to 15)	−0.05 (−0.09 to −0.01)	144.71 (129.78 to 158.76)	119.89 (107.32 to 131.64)	−0.42 (−0.57 to −0.26)
Belize	**Inherited anemias**	2 (1 to 2)	2 (2 to 2)	0.32 (0.28 to 0.36)	877.93 (775.17 to 994.28)	525.08 (464.06 to 592.68)	−1.8 (−1.87 to −1.72)
Belize	Thalassemias	0 (0 to 0)	0 (0 to 0)	1.1 (0.28 to	0.02 (0.01 to	0.02 (0.01 to	−0.22 (−0.52 to

Regions	Causes	Cases in 1990 No.×103 (95% UI)	Cases in 2019 No.×103 (95% UI)	Change in absolute number (%)	Rates in 1990 per 100000 (95% UI)	Rates in 2019 per 100000 (95% UI)	EAPC from 1990 and 2019 (95%CI)
				3.1)	0.03)	0.02)	0.09)
Belize	Thalassemias trait	0 (0 to 0)	0 (0 to 0)	0.51 (0.29 to 0.91)	11.98 (9.27 to 15.06)	8.23 (6.38 to 9.92)	-1.32 (-1.46 to -1.17)
Belize	Sickle cell disorders	0 (0 to 0)	0 (0 to 0)	0.48 (0.32 to 0.65)	12.9 (9.29 to 17.29)	8.64 (6.16 to 11.54)	-1.51 (-1.72 to -1.3)
Belize	Sickle cell trait	1 (0 to 1)	1 (1 to 1)	0.36 (0.28 to 0.43)	304.32 (255.63 to 360.23)	187.16 (155.23 to 222)	-1.71 (-1.83 to -1.6)
Belize	G6PD deficiency	0 (0 to 1)	1 (0 to 1)	0.28 (0.24 to 0.3)	249.41 (191.91 to 312.55)	145.23 (111.48 to 182.28)	-1.86 (-1.95 to -1.78)
Belize	G6PD trait	1 (0 to 1)	1 (1 to 1)	0.3 (0.25 to 0.35)	299.31 (266.36 to 334.43)	175.81 (156.61 to 195.83)	-1.86 (-1.94 to -1.78)
Benin	**Inherited anemias**	117 (107 to 127)	230 (210 to 251)	0.97 (0.89 to 1.04)	2408.92 (2207.43 to 2607.35)	1816.53 (1657.21 to 1983.97)	-0.94 (-1.05 to -0.83)
Benin	Thalassemias	0 (0 to 0)	0 (0 to 0)	1.77 (1.36 to 2.28)	0.38 (0.26 to 0.57)	0.41 (0.28 to 0.56)	0.33 (0.25 to 0.4)
Benin	Thalassemias trait	5 (4 to 6)	11 (9 to 13)	1.37 (1.23 to 1.52)	94.58 (78.33 to 115.6)	85.87 (71.78 to 101.94)	-0.22 (-0.29 to -0.15)
Benin	Sickle cell disorders	8 (7 to 10)	12 (10 to 15)	0.47 (0.32 to 0.62)	169.46 (142.11 to 201.32)	95.64 (76.88 to 115.74)	-2.29 (-2.63 to -1.95)
Benin	Sickle cell trait	51 (46 to 56)	91 (80 to 104)	0.81 (0.67 to 0.94)	1043.27 (943.22 to 1156.97)	722.36 (634.24 to 817.69)	-1.29 (-1.46 to -1.11)
Benin	G6PD deficiency	16 (12 to 20)	35 (27 to 44)	1.2 (1.04 to 1.35)	330.22 (254.3 to 417.92)	278.4 (215.58 to 346.03)	-0.43 (-0.47 to -0.39)
Benin	G6PD trait	37 (34 to 41)	80 (73 to 87)	1.15 (1.07 to 1.19)	771.01 (697.99 to 836.18)	633.86 (575.28 to 686.66)	-0.56 (-0.63 to -0.5)
Bermuda	**Inherited anemias**	0 (0 to 0)	0 (0 to 0)	-0.41 (-0.43 to -0.4)	353.29 (310.06 to 400.11)	192.19 (169.68 to 217.93)	-2.08 (-2.27 to -1.89)
Bermuda	Thalassemias	0 (0 to 0)	0 (0 to 0)	-0.06 (-0.43 to 0.92)	0.01 (0 to 0.01)	0.01 (0 to 0.01)	-0.59 (-1 to -0.17)

Regions	Causes	Cases in 1990 No.×103 (95% UI)	Cases in 2019 No.×103 (95% UI)	Change in absolute number (%)	Rates in 1990 per 100000 (95% UI)	Rates in 2019 per 100000 (95% UI)	EAPC from 1990 and 2019 (95%CI)
Bermuda	Thalassemias trait	0 (0 to 0)	0 (0 to 0)	-0.32 (-0.43 to -0.13)	4.89 (3.83 to 6.1)	3.08 (2.45 to 3.73)	-1.62 (-1.9 to -1.34)
Bermuda	Sickle cell disorders	0 (0 to 0)	0 (0 to 0)	-0.35 (-0.42 to -0.27)	2.05 (1.5 to 2.76)	1.23 (0.88 to 1.66)	-1.81 (-1.94 to -1.69)
Bermuda	Sickle cell trait	0 (0 to 0)	0 (0 to 0)	-0.39 (-0.43 to -0.36)	98.19 (82.41 to 115.4)	55.29 (46.12 to 65.03)	-1.99 (-2.14 to -1.84)
Bermuda	G6PD deficiency	0 (0 to 0)	0 (0 to 0)	-0.43 (-0.43 to -0.43)	110.41 (85.44 to 137.98)	58.55 (45.39 to 73.16)	-2.15 (-2.37 to -1.93)
Bermuda	G6PD trait	0 (0 to 0)	0 (0 to 0)	-0.42 (-0.42 to -0.42)	137.74 (122.56 to 153.63)	74.03 (65.89 to 82.58)	-2.11 (-2.32 to -1.91)
Bhutan	**Inherited anemias**	5 (4 to 5)	3 (3 to 3)	-0.37 (-0.38 to -0.36)	807.07 (718.88 to 896.99)	412.5 (365.79 to 459.79)	-2.22 (-2.26 to -2.18)
Bhutan	Thalassemias	0 (0 to 0)	0 (0 to 0)	-0.31 (-0.38 to -0.25)	1.61 (1.16 to 2.16)	0.9 (0.62 to 1.23)	-1.89 (-1.92 to -1.86)
Bhutan	Thalassemias trait	1 (1 to 1)	1 (1 to 1)	-0.37 (-0.4 to -0.34)	162.44 (138.08 to 189.62)	83 (70.07 to 97.61)	-2.2 (-2.24 to -2.17)
Bhutan	Sickle cell disorders	0 (0 to 0)	0 (0 to 0)	-0.4 (-0.46 to -0.29)	0.06 (0.04 to 0.09)	0.03 (0.02 to 0.04)	-2.44 (-2.5 to -2.39)
Bhutan	Sickle cell trait	0 (0 to 0)	0 (0 to 0)	-0.4 (-0.43 to -0.34)	28.23 (22.76 to 35.02)	13.79 (11.12 to 17)	-2.4 (-2.45 to -2.35)
Bhutan	G6PD deficiency	1 (1 to 1)	1 (1 to 1)	-0.34 (-0.38 to -0.3)	169.58 (130.91 to 211.22)	91.36 (70.82 to 113.64)	-2.21 (-2.27 to -2.15)
Bhutan	G6PD trait	3 (2 to 3)	2 (2 to 2)	-0.38 (-0.4 to -0.37)	445.15 (398.87 to 487.08)	223.42 (200.16 to 244.62)	-2.23 (-2.27 to -2.18)
Bolivia (Plurinational State of)	**Inherited anemias**	38 (34 to 42)	57 (50 to 64)	0.51 (0.46 to 0.56)	586.8 (527.72 to 647.5)	473.68 (418.07 to 531.53)	-0.93 (-1.12 to -0.75)
Bolivia (Plurinational State	Thalassemias	0 (0 to 0)	0 (0 to 0)	0.61 (0.27 to 1.11)	0.07 (0.05 to 0.09)	0.06 (0.04 to 0.07)	-0.33 (-0.43 to -0.22)

Regions	Causes	Cases in 1990 No.×103 (95% UI)	Cases in 2019 No.×103 (95% UI)	Change in absolute number (%)	Rates in 1990 per 100000 (95% UI)	Rates in 2019 per 100000 (95% UI)	EAPC from 1990 and 2019 (95%CI)
of)							
Bolivia (Plurinational State of)	Thalassemias trait	2 (2 to 3)	3 (3 to 4)	0.43 (0.29 to 0.61)	33.87 (29.47 to 38.94)	25.95 (22.51 to 29.74)	-0.74 (-0.88 to -0.6)
Bolivia (Plurinational State of)	Sickle cell disorders	0 (0 to 0)	0 (0 to 0)	0.35 (0.21 to 0.5)	0.24 (0.16 to 0.33)	0.17 (0.12 to 0.24)	-0.98 (-1.11 to -0.86)
Bolivia (Plurinational State of)	Sickle cell trait	4 (3 to 4)	5 (4 to 6)	0.34 (0.27 to 0.42)	57.29 (46.6 to 69.04)	41.14 (33.8 to 49.6)	-0.99 (-1.13 to -0.85)
Bolivia (Plurinational State of)	G6PD deficiency	4 (3 to 5)	12 (9 to 15)	2.33 (2.1 to 2.52)	56.92 (44.28 to 72.43)	101.21 (78.52 to 126.22)	-0.24 (-0.96 to 0.49)
Bolivia (Plurinational State of)	G6PD trait	28 (25 to 31)	37 (33 to 41)	0.3 (0.24 to 0.36)	438.42 (394.02 to 484.65)	305.15 (273.41 to 338.4)	-1.08 (-1.23 to -0.93)
Bosnia and Herzegovina	Inherited anemias	9 (8 to 11)	4 (3 to 4)	-0.59 (-0.6 to -0.58)	208.26 (184.33 to 234.32)	117.11 (103.79 to 131.62)	-1.85 (-2.05 to -1.65)
Bosnia and Herzegovina	Thalassemias	0 (0 to 0)	0 (0 to 0)	-0.76 (-0.78 to -0.72)	1.08 (0.71 to 1.7)	0.36 (0.25 to 0.54)	-3.37 (-3.59 to -3.14)
Bosnia and Herzegovina	Thalassemias trait	1 (1 to 2)	0 (0 to 1)	-0.6 (-0.63 to -0.56)	27.29 (22.88 to 33.44)	15.13 (12.73 to 18.39)	-1.74 (-1.97 to -1.51)
Bosnia and Herzegovina	Sickle cell disorders	0 (0 to 0)	0 (0 to 0)	-0.56 (-0.6 to -0.5)	0.11 (0.08 to 0.15)	0.07 (0.05 to 0.09)	-1.46 (-1.6 to -1.31)
Bosnia and Herzegovina	Sickle cell trait	1 (1 to 1)	0 (0 to 1)	-0.58 (-0.6 to -0.56)	24.12 (20.74 to 27.79)	13.91 (11.91 to 15.98)	-1.58 (-1.76 to -1.4)
Bosnia and Herzegovina	G6PD deficiency	2 (2 to 3)	1 (1 to 1)	-0.57 (-0.6 to -0.53)	50.89 (39.45 to 63.51)	30.32 (23.47 to 37.67)	-2.39 (-2.73 to -2.04)
Bosnia and Herzegovina	G6PD trait	5 (4 to 5)	2 (2 to 2)	-0.6 (-0.6 to -0.6)	104.78 (92.95 to 117.92)	57.32 (50.85 to 64.51)	-1.7 (-1.92 to -1.49)
Botswana	Inherited	12	13	0.15	888.63	569.67	-1.36

Regions	Causes	Cases in 1990 No.×103 (95% UI)	Cases in 2019 No.×103 (95% UI)	Change in absolute number (%)	Rates in 1990 per 100000 (95% UI)	Rates in 2019 per 100000 (95% UI)	EAPC from 1990 and 2019 (95%CI)
	anemias	(10 to 13)	(12 to 15)	(0.12 to 0.18)	(775.93 to 1008.11)	(498.42 to 646.06)	(-1.46 to -1.27)
Botswana	Thalassemias	0 (0 to 0)	0 (0 to 0)	1.5 (1.03 to 2.08)	0.06 (0.04 to 0.09)	0.08 (0.05 to 0.13)	1 (0.84 to 1.15)
Botswana	Thalassemias trait	0 (0 to 0)	0 (0 to 1)	0.53 (0.38 to 0.68)	22.41 (19.03 to 26.31)	19.05 (15.52 to 22.53)	-0.52 (-0.59 to -0.45)
Botswana	Sickle cell disorders	0 (0 to 0)	0 (0 to 0)	0.18 (0.07 to 0.29)	0.09 (0.06 to 0.14)	0.06 (0.04 to 0.09)	-1.3 (-1.37 to -1.24)
Botswana	Sickle cell trait	0 (0 to 0)	0 (0 to 1)	0.19 (0.12 to 0.25)	28.61 (22.16 to 37.16)	18.91 (14.55 to 24.67)	-1.29 (-1.35 to -1.22)
Botswana	G6PD deficiency	4 (3 to 5)	4 (3 to 5)	0.11 (0.03 to 0.16)	287.06 (221.96 to 362.37)	176.59 (135.68 to 220.41)	-1.51 (-1.64 to -1.38)
Botswana	G6PD trait	7 (7 to 8)	8 (8 to 9)	0.16 (0.12 to 0.2)	550.39 (501.94 to 594.72)	354.97 (323.51 to 385.17)	-1.33 (-1.42 to -1.25)
Brazil	Inherited anemias	589 (576 to 603)	573 (560 to 587)	-0.03 (-0.03 to -0.02)	396.02 (386.75 to 405.37)	264.62 (258.45 to 270.84)	-0.92 (-1.17 to -0.66)
Brazil	Thalassemias	0 (0 to 0)	0 (0 to 0)	-0.11 (-0.25 to 0.09)	0.06 (0.04 to 0.08)	0.03 (0.03 to 0.05)	-1.49 (-1.6 to -1.39)
Brazil	Thalassemias trait	28 (24 to 33)	25 (22 to 29)	-0.1 (-0.17 to 0)	18.83 (15.93 to 21.99)	11.7 (9.99 to 13.48)	-1.45 (-1.53 to -1.37)
Brazil	Sickle cell disorders	1 (1 to 1)	1 (1 to 1)	-0.1 (-0.16 to -0.03)	0.6 (0.49 to 0.73)	0.37 (0.31 to 0.45)	-2.04 (-2.2 to -1.89)
Brazil	Sickle cell trait	77 (69 to 85)	78 (70 to 86)	0.02 (-0.02 to 0.05)	51.43 (46.03 to 57.27)	35.9 (32.38 to 39.69)	-1.07 (-1.16 to -0.98)
Brazil	G6PD deficiency	91 (87 to 94)	106 (102 to 110)	0.17 (0.15 to 0.19)	61.07 (58.75 to 63.2)	49.05 (47.16 to 50.93)	0.15 (-0.39 to 0.68)
Brazil	G6PD trait	393 (386 to 400)	363 (356 to 369)	-0.08 (-0.08 to -0.07)	264.02 (259.14 to 268.62)	167.56 (164.39 to 170.45)	-1.14 (-1.37 to -0.91)
Brunei Darussalam	Inherited anemias	1 (0 to 1)	0 (0 to 0)	-0.3 (-0.33 to	205.43 (175.33 to	85.19 (74.65 to	-3.26 (-3.49 to

Regions	Causes	Cases in 1990 No.×103 (95% UI)	Cases in 2019 No.×103 (95% UI)	Change in absolute number (%)	Rates in 1990 per 100000 (95% UI)	Rates in 2019 per 100000 (95% UI)	EAPC from 1990 and 2019 (95%CI)
				-0.26)	237.26)	96.88)	-3.04)
Brunei Darussalam	Thalassemias	0 (0 to 0)	0 (0 to 0)	-0.25 (-0.34 to -0.14)	1.25 (0.81 to 1.97)	0.56 (0.36 to 0.88)	-3.01 (-3.17 to -2.85)
Brunei Darussalam	Thalassemias trait	0 (0 to 0)	0 (0 to 0)	0.57 (0.3 to 0.89)	7.77 (6 to 9.91)	7.22 (5.93 to 8.72)	-0.7 (-1 to -0.39)
Brunei Darussalam	Sickle cell disorders	0 (0 to 0)	0 (0 to 0)	-0.08 (-0.27 to 0.04)	0.03 (0.02 to 0.04)	0.01 (0.01 to 0.02)	-2.21 (-2.38 to -2.05)
Brunei Darussalam	Sickle cell trait	0 (0 to 0)	0 (0 to 0)	-0.07 (-0.18 to 0.05)	12.74 (10.09 to 15.71)	7.03 (5.55 to 8.7)	-2.21 (-2.36 to -2.06)
Brunei Darussalam	G6PD deficiency	0 (0 to 0)	0 (0 to 0)	-0.76 (-0.78 to -0.73)	58.85 (44.99 to 73.59)	8.51 (6.51 to 10.76)	-6.6 (-7.85 to -5.35)
Brunei Darussalam	G6PD trait	0 (0 to 0)	0 (0 to 0)	-0.16 (-0.2 to -0.11)	124.79 (108.43 to 143.64)	61.86 (53.28 to 70.97)	-2.65 (-2.74 to -2.56)
Bulgaria	**Inherited anemias**	19 (17 to 21)	11 (10 to 12)	-0.41 (-0.42 to -0.4)	215.94 (192.58 to 242.37)	159.29 (142.67 to 177.49)	-0.2 (-0.54 to 0.16)
Bulgaria	Thalassemias	0 (0 to 0)	0 (0 to 0)	-0.72 (-0.77 to -0.66)	1.57 (1.15 to 2.12)	0.55 (0.39 to 0.8)	-2.87 (-3.39 to -2.34)
Bulgaria	Thalassemias trait	3 (2 to 3)	2 (1 to 2)	-0.38 (-0.41 to -0.34)	30.28 (25.89 to 35.49)	23.69 (20.18 to 27.52)	0.02 (-0.33 to 0.36)
Bulgaria	Sickle cell disorders	0 (0 to 0)	0 (0 to 0)	-0.23 (-0.32 to -0.13)	0.1 (0.08 to 0.13)	0.1 (0.07 to 0.13)	0.46 (0.16 to 0.76)
Bulgaria	Sickle cell trait	2 (2 to 2)	1 (1 to 1)	-0.32 (-0.36 to -0.28)	20.26 (17.39 to 23.44)	17.2 (14.63 to 19.92)	0.18 (-0.13 to 0.49)
Bulgaria	G6PD deficiency	4 (3 to 5)	2 (2 to 3)	-0.5 (-0.5 to -0.49)	48.28 (37.23 to 60.41)	30.44 (23.54 to 38)	-0.79 (-1.21 to -0.36)
Bulgaria	G6PD trait	10 (9 to 11)	6 (5 to 7)	-0.4 (-0.4 to -0.4)	115.44 (103 to 128.44)	87.32 (77.91 to 97.15)	-0.07 (-0.42 to 0.28)
Burkina Faso	**Inherited anemias**	305 (295 to 317)	588 (530 to 647)	0.92 (0.75 to 1.11)	3195.97 (3081.68 to 3312.35)	2590.21 (2337.45 to 2852.36)	-0.75 (-0.83 to -0.67)

Regions	Causes	Cases in 1990 No.×103 (95% UI)	Cases in 2019 No.×103 (95% UI)	Change in absolute number (%)	Rates in 1990 per 100000 (95% UI)	Rates in 2019 per 100000 (95% UI)	EAPC from 1990 and 2019 (95%CI)
Burkina Faso	Thalassemias	0 (0 to 0)	0 (0 to 0)	1.65 (1.33 to 2.05)	0.4 (0.3 to 0.53)	0.45 (0.33 to 0.59)	0.36 (0.31 to 0.42)
Burkina Faso	Thalassemias trait	9 (8 to 11)	21 (18 to 24)	1.28 (1.17 to 1.39)	95.91 (82.81 to 111.05)	91.91 (79.52 to 105.6)	-0.17 (-0.21 to -0.13)
Burkina Faso	Sickle cell disorders	15 (14 to 17)	22 (18 to 27)	0.45 (0.2 to 0.7)	161.95 (144.14 to 182.27)	98.79 (80.25 to 120.79)	-1.72 (-1.96 to -1.49)
Burkina Faso	Sickle cell trait	103 (96 to 110)	185 (164 to 209)	0.8 (0.61 to 0.99)	1074.27 (1000.12 to 1149.04)	814.2 (722.19 to 921.26)	-1.04 (-1.3 to -0.78)
Burkina Faso	G6PD deficiency	84 (79 to 89)	167 (129 to 207)	0.98 (0.54 to 1.46)	882.04 (829.8 to 936.05)	734.66 (568.79 to 912.74)	-0.6 (-0.78 to -0.41)
Burkina Faso	G6PD trait	94 (92 to 95)	193 (179 to 205)	1.06 (0.91 to 1.19)	981.4 (963.85 to 997.85)	850.22 (787.48 to 905.22)	-0.51 (-0.54 to -0.47)
Burundi	**Inherited anemias**	94 (84 to 105)	151 (135 to 168)	0.61 (0.57 to 0.65)	1687.06 (1500.45 to 1880.72)	1268.05 (1133.67 to 1411.39)	-0.77 (-0.91 to -0.63)
Burundi	Thalassemias	0 (0 to 0)	0 (0 to 0)	2.95 (2.37 to 3.75)	0.1 (0.06 to 0.15)	0.18 (0.1 to 0.28)	2.02 (1.86 to 2.18)
Burundi	Thalassemias trait	2 (1 to 2)	4 (3 to 5)	1.44 (1.24 to 1.7)	27.95 (22.01 to 34.06)	31.84 (24.84 to 39.11)	0.51 (0.43 to 0.59)
Burundi	Sickle cell disorders	0 (0 to 0)	0 (0 to 0)	0.6 (0.39 to 0.76)	4.07 (3.15 to 5.28)	3.04 (2.39 to 3.98)	-0.9 (-1 to -0.8)
Burundi	Sickle cell trait	15 (13 to 17)	25 (22 to 28)	0.68 (0.56 to 0.76)	265.11 (234.4 to 302.41)	207.56 (184.15 to 237.8)	-0.7 (-0.78 to -0.62)
Burundi	G6PD deficiency	28 (22 to 35)	40 (31 to 49)	0.43 (0.35 to 0.5)	499.83 (386.99 to 623.44)	333.9 (258.95 to 414.23)	-1.17 (-1.42 to -0.91)
Burundi	G6PD trait	50 (46 to 53)	83 (75 to 89)	0.66 (0.63 to 0.71)	890.01 (818.65 to 955.83)	691.52 (631.85 to 746.91)	-0.63 (-0.75 to -0.52)
Cabo Verde	**Inherited anemias**	3 (3 to 3)	3 (2 to 3)	-0.04 (-0.06 to -0.02)	802.56 (729.45 to 879.12)	480.2 (433.64 to 528.84)	-1.61 (-1.7 to -1.52)
Cabo Verde	Thalassemi	0	0	0.18	0.18	0.13	-0.95

Regions	Causes	Cases in 1990 No.×103 (95% UI)	Cases in 2019 No.×103 (95% UI)	Change in absolute number (%)	Rates in 1990 per 100000 (95% UI)	Rates in 2019 per 100000 (95% UI)	EAPC from 1990 and 2019 (95%CI)
	as	(0 to 0)	(0 to 0)	(0 to 0.42)	(0.13 to 0.27)	(0.09 to 0.19)	(-1.02 to -0.89)
Cabo Verde	Thalassemias trait	0 (0 to 0)	0 (0 to 0)	0.01 (-0.06 to 0.08)	53.75 (44.78 to 66.23)	33.84 (28.36 to 40.67)	-1.48 (-1.56 to -1.41)
Cabo Verde	Sickle cell disorders	0 (0 to 0)	0 (0 to 0)	-0.09 (-0.15 to -0.03)	2.6 (1.97 to 3.33)	1.47 (1.14 to 1.9)	-1.79 (-1.9 to -1.68)
Cabo Verde	Sickle cell trait	0 (0 to 0)	0 (0 to 0)	-0.08 (-0.12 to -0.05)	116.88 (101.92 to 134.03)	67.15 (58.06 to 77.26)	-1.73 (-1.84 to -1.62)
Cabo Verde	G6PD deficiency	0 (0 to 0)	0 (0 to 1)	0.09 (0.01 to 0.17)	111.32 (88.24 to 137.44)	75.64 (58.16 to 94.95)	-0.96 (-1.14 to -0.78)
Cabo Verde	G6PD trait	2 (2 to 2)	2 (2 to 2)	-0.07 (-0.08 to -0.04)	517.83 (469.27 to 563.32)	301.97 (273.75 to 329.53)	-1.75 (-1.84 to -1.66)
Cambodia	**Inherited anemias**	171 (153 to 191)	150 (135 to 166)	-0.12 (-0.14 to -0.1)	1648.13 (1474.12 to 1840.62)	905.67 (813.56 to 1002.68)	-1.61 (-1.93 to -1.29)
Cambodia	Thalassemias	3 (2 to 4)	3 (3 to 4)	0.2 (0.08 to 0.3)	27.4 (22.43 to 33.75)	20.63 (16.33 to 26.32)	-0.87 (-1.09 to -0.64)
Cambodia	Thalassemias trait	44 (40 to 49)	44 (40 to 48)	-0.01 (-0.05 to 0.02)	426.3 (387.57 to 473.2)	262.81 (239.85 to 289.34)	-1.56 (-1.76 to -1.36)
Cambodia	Sickle cell disorders	0 (0 to 0)	0 (0 to 0)	-0.16 (-0.25 to 0)	0.04 (0.03 to 0.06)	0.02 (0.01 to 0.03)	-2.1 (-2.32 to -1.87)
Cambodia	Sickle cell trait	2 (2 to 3)	2 (2 to 2)	-0.17 (-0.21 to -0.09)	23.78 (19.46 to 28.49)	12.41 (10.17 to 14.73)	-2.12 (-2.33 to -1.9)
Cambodia	G6PD deficiency	50 (38 to 62)	41 (32 to 51)	-0.16 (-0.23 to -0.1)	477.44 (369.01 to 593.06)	249.9 (192.77 to 309.91)	-1.65 (-2.02 to -1.28)
Cambodia	G6PD trait	72 (65 to 78)	60 (54 to 65)	-0.17 (-0.17 to -0.17)	693.18 (629.95 to 755.45)	359.9 (327.06 to 392.28)	-1.63 (-2.01 to -1.25)
Cameroon	**Inherited anemias**	186 (173 to 200)	372 (343 to 403)	1 (0.93 to 1.09)	1789.38 (1664.05 to 1921.73)	1279.35 (1178.35 to 1384)	-1.01 (-1.23 to -0.8)
Cameroon	Thalassemias	0 (0 to 0)	0 (0 to 0)	1.6 (1.24 to	0.34 (0.23 to	0.31 (0.22 to	-0.12 (-0.32 to

Regions	Causes	Cases in 1990 No.×103 (95% UI)	Cases in 2019 No.×103 (95% UI)	Change in absolute number (%)	Rates in 1990 per 100000 (95% UI)	Rates in 2019 per 100000 (95% UI)	EAPC from 1990 and 2019 (95%CI)
				2.06)	0.49)	0.44)	0.08)
Cameroon	Thalassemias trait	9 (7 to 11)	19 (16 to 23)	1.19 (1.07 to 1.33)	84.92 (70.02 to 103.28)	66.45 (55.69 to 79.48)	-0.69 (-0.9 to -0.49)
Cameroon	Sickle cell disorders	4 (3 to 4)	8 (7 to 10)	1.14 (0.79 to 1.58)	36.27 (32.79 to 40.06)	27.71 (22.61 to 35.32)	-0.89 (-1.12 to -0.65)
Cameroon	Sickle cell trait	75 (72 to 79)	152 (139 to 171)	1.03 (0.87 to 1.21)	722.87 (689.72 to 757.38)	523.58 (477.08 to 586.05)	-0.99 (-1.22 to -0.77)
Cameroon	G6PD deficiency	24 (19 to 30)	48 (37 to 60)	0.96 (0.89 to 1.08)	234.22 (180.98 to 291.23)	164.1 (126.61 to 206.75)	-1.06 (-1.26 to -0.86)
Cameroon	G6PD trait	74 (67 to 80)	145 (131 to 157)	0.96 (0.96 to 0.97)	710.77 (644.3 to 771.92)	497.19 (451.29 to 539.69)	-1.06 (-1.27 to -0.86)
Canada	**Inherited anemias**	72 (63 to 82)	57 (49 to 65)	-0.21 (-0.25 to -0.18)	264.48 (229.92 to 302.41)	155.59 (134.65 to 178.23)	-1.57 (-1.81 to -1.32)
Canada	Thalassemias	0 (0 to 0)	0 (0 to 0)	-0.21 (-0.34 to -0.07)	0.07 (0.05 to 0.09)	0.04 (0.03 to 0.06)	-2.18 (-2.38 to -1.97)
Canada	Thalassemias trait	2 (1 to 2)	1 (1 to 2)	-0.14 (-0.32 to 0.04)	5.56 (4.7 to 6.51)	3.59 (2.96 to 4.26)	-0.99 (-1.3 to -0.68)
Canada	Sickle cell disorders	0 (0 to 0)	0 (0 to 0)	-0.85 (-0.9 to -0.77)	0.08 (0.05 to 0.12)	0.01 (0.01 to 0.01)	-6.76 (-8.48 to -5.01)
Canada	Sickle cell trait	2 (1 to 2)	1 (1 to 2)	-0.16 (-0.35 to -0.07)	5.81 (4.85 to 6.86)	3.63 (2.9 to 4.43)	-1.31 (-1.6 to -1.03)
Canada	G6PD deficiency	19 (15 to 24)	15 (12 to 19)	-0.2 (-0.25 to -0.14)	70.03 (54.12 to 87.07)	41.65 (32.13 to 52.51)	-1.62 (-1.87 to -1.37)
Canada	G6PD trait	50 (45 to 55)	39 (35 to 43)	-0.22 (-0.26 to -0.19)	182.93 (164.38 to 202.04)	106.67 (95.49 to 118.3)	-1.57 (-1.82 to -1.33)
Central African Republic	**Inherited anemias**	65 (59 to 71)	101 (92 to 111)	0.55 (0.51 to 0.59)	2376.47 (2161.09 to 2599.21)	1908.83 (1732.81 to 2103.6)	-0.71 (-0.78 to -0.64)
Central African Republic	Thalassemias	0 (0 to 0)	0 (0 to 0)	2.2 (1.76 to 2.72)	0.11 (0.07 to 0.16)	0.18 (0.1 to 0.28)	1.56 (1.4 to 1.73)

Regions	Causes	Cases in 1990 No.×103 (95% UI)	Cases in 2019 No.×103 (95% UI)	Change in absolute number (%)	Rates in 1990 per 100000 (95% UI)	Rates in 2019 per 100000 (95% UI)	EAPC from 1990 and 2019 (95%CI)
Central African Republic	Thalassemias trait	1 (1 to 1)	2 (2 to 2)	0.98 (0.84 to 1.13)	37.55 (31.26 to 44.42)	38.48 (31.44 to 46.36)	0.05 (-0.03 to 0.13)
Central African Republic	Sickle cell disorders	1 (1 to 2)	2 (1 to 2)	0.52 (0.38 to 0.68)	45.34 (35.59 to 58.72)	35.74 (27.86 to 47.03)	-0.76 (-0.82 to -0.7)
Central African Republic	Sickle cell trait	23 (20 to 25)	35 (31 to 39)	0.54 (0.47 to 0.61)	822.03 (738.55 to 924.95)	653.99 (585.57 to 741.41)	-0.73 (-0.8 to -0.67)
Central African Republic	G6PD deficiency	15 (12 to 19)	23 (18 to 29)	0.56 (0.46 to 0.66)	546.31 (422.22 to 677.9)	440.79 (341.49 to 552.03)	-0.73 (-0.81 to -0.65)
Central African Republic	G6PD trait	25 (23 to 27)	39 (36 to 42)	0.54 (0.5 to 0.58)	925.13 (853.3 to 988.9)	739.64 (683.05 to 792.93)	-0.71 (-0.78 to -0.64)
Chad	**Inherited anemias**	108 (98 to 119)	265 (240 to 292)	1.46 (1.39 to 1.52)	1791.43 (1619.68 to 1968.92)	1617.72 (1461.37 to 1782.15)	-0.37 (-0.42 to -0.32)
Chad	Thalassemias	0 (0 to 0)	0 (0 to 0)	2.36 (1.92 to 2.89)	0.36 (0.27 to 0.48)	0.45 (0.32 to 0.6)	0.66 (0.6 to 0.72)
Chad	Thalassemias trait	6 (5 to 7)	16 (14 to 18)	1.76 (1.64 to 1.92)	95.78 (82.04 to 111.77)	97.21 (83.72 to 112.3)	0 (-0.04 to 0.05)
Chad	Sickle cell disorders	1 (1 to 1)	2 (1 to 2)	1.41 (1.21 to 1.6)	11.35 (9.21 to 13.71)	10.04 (8.27 to 12.21)	-0.43 (-0.48 to -0.38)
Chad	Sickle cell trait	27 (25 to 30)	67 (61 to 74)	1.44 (1.33 to 1.53)	455.8 (410.9 to 501)	407.73 (369.81 to 449.62)	-0.4 (-0.45 to -0.36)
Chad	G6PD deficiency	24 (19 to 30)	57 (44 to 71)	1.37 (1.18 to 1.58)	396.94 (307.63 to 492.97)	346.15 (267.56 to 432.04)	-0.45 (-0.52 to -0.38)
Chad	G6PD trait	50 (45 to 54)	124 (112 to 135)	1.48 (1.39 to 1.55)	831.19 (753.63 to 904.27)	756.14 (684.57 to 820.83)	-0.36 (-0.41 to -0.32)
Chile	**Inherited anemias**	30 (26 to 34)	22 (20 to 25)	-0.25 (-0.28 to -0.22)	225.26 (198.52 to 254.58)	122.87 (108.79 to 138.74)	-1.85 (-2.03 to -1.67)
Chile	Thalassemias	0 (0 to 0)	0 (0 to 0)	-0.22 (-0.36 to 0.02)	0.28 (0.18 to 0.41)	0.16 (0.12 to 0.22)	-1.64 (-1.99 to -1.3)
Chile	Thalassemi	2	3	0.36	15.55	15.47	0.47

Regions	Causes	Cases in 1990 No.×103 (95% UI)	Cases in 2019 No.×103 (95% UI)	Change in absolute number (%)	Rates in 1990 per 100000 (95% UI)	Rates in 2019 per 100000 (95% UI)	EAPC from 1990 and 2019 (95%CI)
	as trait	(2 to 2)	(2 to 3)	(0.21 to 0.53)	(13.31 to 18.21)	(13.63 to 17.39)	(0.21 to 0.73)
Chile	Sickle cell disorders	0 (0 to 0)	0 (0 to 0)	-0.25 (-0.5 to -0.12)	0 (0 to 0.01)	0 (0 to 0)	-1.53 (-1.82 to -1.24)
Chile	Sickle cell trait	1 (0 to 1)	0 (0 to 1)	-0.25 (-0.47 to -0.21)	4.17 (2.4 to 6.35)	2.28 (1.29 to 3.44)	-1.6 (-1.84 to -1.35)
Chile	G6PD deficiency	5 (4 to 6)	3 (3 to 4)	-0.27 (-0.31 to -0.23)	34.21 (26.52 to 43.62)	18.3 (14.15 to 22.91)	-2.02 (-2.19 to -1.84)
Chile	G6PD trait	23 (20 to 25)	16 (14 to 18)	-0.31 (-0.33 to -0.27)	171.04 (152.05 to 191.27)	86.66 (76.7 to 97.49)	-2.11 (-2.3 to -1.93)
China	**Inherited anemias**	6133 (5719 to 6696)	4025 (3790 to 4339)	-0.34 (-0.36 to -0.33)	518.17 (483.18 to 565.73)	282.95 (266.48 to 305.08)	-2.05 (-2.37 to -1.74)
China	Thalassemias	101 (72 to 143)	60 (43 to 86)	-0.4 (-0.44 to -0.36)	8.51 (6.06 to 12.08)	4.23 (3.04 to 6.04)	-2.34 (-2.85 to -1.84)
China	Thalassemias trait	2543 (2164 to 3051)	1475 (1261 to 1748)	-0.42 (-0.44 to -0.4)	214.83 (182.84 to 257.76)	103.73 (88.68 to 122.9)	-2.27 (-2.82 to -1.71)
China	Sickle cell disorders	1 (1 to 2)	0 (0 to 1)	-0.56 (-0.57 to -0.53)	0.09 (0.06 to 0.13)	0.03 (0.02 to 0.05)	-3.53 (-4.22 to -2.84)
China	Sickle cell trait	303 (249 to 365)	158 (132 to 190)	-0.48 (-0.49 to -0.46)	25.58 (20.99 to 30.8)	11.13 (9.31 to 13.33)	-2.8 (-3.41 to -2.19)
China	G6PD deficiency	841 (808 to 872)	724 (691 to 753)	-0.14 (-0.17 to -0.12)	71.04 (68.26 to 73.65)	50.89 (48.6 to 52.97)	-0.89 (-1.08 to -0.7)
China	G6PD trait	2345 (2303 to 2382)	1606 (1576 to 1635)	-0.32 (-0.32 to -0.31)	198.12 (194.6 to 201.24)	112.94 (110.79 to 114.96)	-2.23 (-2.43 to -2.03)
Colombia	**Inherited anemias**	210 (186 to 235)	180 (159 to 202)	-0.15 (-0.16 to -0.11)	646.5 (572.18 to 722.57)	376.47 (333.1 to 423.5)	-2.23 (-2.5 to -1.96)
Colombia	Thalassemias	0 (0 to 0)	0 (0 to 0)	0.36 (-0.15 to 1.49)	0.02 (0.01 to 0.03)	0.01 (0.01 to 0.02)	-0.46 (-0.7 to -0.22)
Colombia	Thalassemias trait	4 (3 to 5)	4 (3 to 4)	-0.01 (-0.15 to	11.78 (9.73 to	7.97 (6.62 to	-1.49 (-1.57 to

Regions	Causes	Cases in 1990 No.×103 (95% UI)	Cases in 2019 No.×103 (95% UI)	Change in absolute number (%)	Rates in 1990 per 100000 (95% UI)	Rates in 2019 per 100000 (95% UI)	EAPC from 1990 and 2019 (95%CI)
				0.21)	14.42)	9.36)	-1.41)
Colombia	Sickle cell disorders	0 (0 to 0)	0 (0 to 0)	-0.16 (-0.24 to -0.05)	0.46 (0.31 to 0.66)	0.26 (0.18 to 0.37)	-1.98 (-2.02 to -1.94)
Colombia	Sickle cell trait	9 (8 to 10)	7 (6 to 8)	-0.18 (-0.25 to -0.14)	26.52 (23.49 to 29.96)	14.74 (13.15 to 16.58)	-1.95 (-2.02 to -1.88)
Colombia	G6PD deficiency	48 (37 to 60)	41 (32 to 51)	-0.15 (-0.2 to -0.1)	147.51 (113.84 to 183.72)	85.5 (66.18 to 106.81)	-2.54 (-3.05 to -2.02)
Colombia	G6PD trait	150 (136 to 162)	128 (116 to 140)	-0.15 (-0.16 to -0.12)	460.21 (417.91 to 497.27)	267.98 (243.46 to 292.21)	-2.18 (-2.4 to -1.96)
Comoros	**Inherited anemias**	6 (6 to 7)	5 (4 to 5)	-0.24 (-0.26 to -0.22)	1361.57 (1187.7 to 1535.19)	670.83 (590.62 to 755.67)	-2.5 (-2.67 to -2.32)
Comoros	Thalassemias	0 (0 to 0)	0 (0 to 0)	0.83 (0.57 to 1.2)	0.08 (0.05 to 0.12)	0.1 (0.05 to 0.15)	0.2 (-0.02 to 0.41)
Comoros	Thalassemias trait	0 (0 to 0)	0 (0 to 0)	0.14 (0.05 to 0.26)	24.78 (19.46 to 30.4)	18.43 (14.56 to 22.44)	-1.26 (-1.39 to -1.13)
Comoros	Sickle cell disorders	0 (0 to 0)	0 (0 to 0)	-0.18 (-0.25 to -0.08)	0.54 (0.34 to 0.83)	0.29 (0.18 to 0.45)	-2.19 (-2.28 to -2.1)
Comoros	Sickle cell trait	0 (0 to 1)	0 (0 to 0)	-0.17 (-0.22 to -0.12)	88.56 (68.49 to 113.2)	47.67 (36.96 to 61.11)	-2.21 (-2.29 to -2.12)
Comoros	G6PD deficiency	2 (2 to 3)	1 (1 to 2)	-0.32 (-0.36 to -0.28)	444.16 (341.86 to 552.39)	195.47 (151.58 to 246.5)	-2.89 (-3.18 to -2.59)
Comoros	G6PD trait	4 (3 to 4)	3 (3 to 3)	-0.22 (-0.24 to -0.19)	803.46 (735.48 to 866.53)	408.87 (373.08 to 441.93)	-2.37 (-2.51 to -2.24)
Congo	**Inherited anemias**	50 (45 to 55)	71 (63 to 78)	0.41 (0.37 to 0.46)	2049.99 (1847.05 to 2258.65)	1342.47 (1203.96 to 1489.15)	-1.18 (-1.43 to -0.93)
Congo	Thalassemias	0 (0 to 0)	0 (0 to 0)	1.88 (1.44 to 2.39)	0.09 (0.06 to 0.14)	0.12 (0.07 to 0.18)	1.13 (0.87 to 1.4)
Congo	Thalassemias trait	1 (1 to 1)	1 (1 to 2)	0.81 (0.67 to 0.97)	32.22 (26.8 to 38.23)	27.01 (22.04 to 31.98)	-0.36 (-0.6 to -0.13)

Regions	Causes	Cases in 1990 No.×103 (95% UI)	Cases in 2019 No.×103 (95% UI)	Change in absolute number (%)	Rates in 1990 per 100000 (95% UI)	Rates in 2019 per 100000 (95% UI)	EAPC from 1990 and 2019 (95%CI)
Congo	Sickle cell disorders	1 (1 to 1)	1 (1 to 1)	0.23 (0.1 to 0.36)	27.04 (21.72 to 32.92)	15.49 (12.09 to 19.48)	-1.67 (-1.96 to -1.37)
Congo	Sickle cell trait	14 (12 to 15)	18 (16 to 20)	0.33 (0.24 to 0.4)	563.74 (501.76 to 622.74)	347.42 (305.17 to 388.39)	-1.4 (-1.66 to -1.14)
Congo	G6PD deficiency	14 (11 to 18)	21 (16 to 26)	0.46 (0.35 to 0.59)	587.04 (463.2 to 725.33)	397.26 (306.38 to 493.65)	-1.1 (-1.37 to -0.83)
Congo	G6PD trait	21 (19 to 22)	29 (27 to 31)	0.42 (0.42 to 0.44)	839.87 (775.05 to 897.59)	555.16 (511.91 to 593.41)	-1.11 (-1.35 to -0.86)
Cook Islands	**Inherited anemias**	0 (0 to 0)	0 (0 to 0)	-0.36 (-0.37 to -0.35)	514.26 (457.02 to 577.72)	347.86 (307.13 to 390.03)	-1.37 (-1.51 to -1.23)
Cook Islands	Thalassemias	0 (0 to 0)	0 (0 to 0)	-0.5 (-0.57 to -0.41)	1.99 (1.1 to 3.21)	1.06 (0.58 to 1.77)	-0.87 (-1.99 to 0.27)
Cook Islands	Thalassemias trait	0 (0 to 0)	0 (0 to 0)	-0.45 (-0.49 to -0.41)	71.72 (54.98 to 89.64)	41.8 (31.97 to 53.19)	-1.36 (-1.82 to -0.9)
Cook Islands	Sickle cell disorders	0 (0 to 0)	0 (0 to 0)	-0.42 (-0.43 to -0.29)	0 (0 to 0)	0 (0 to 0)	-1.78 (-1.84 to -1.71)
Cook Islands	Sickle cell trait	0 (0 to 0)	0 (0 to 0)	-0.42 (-0.43 to -0.34)	3.02 (1.33 to 4.87)	1.85 (0.8 to 2.99)	-1.8 (-1.87 to -1.74)
Cook Islands	G6PD deficiency	0 (0 to 0)	0 (0 to 0)	-0.31 (-0.34 to -0.26)	107.18 (83.24 to 134.41)	78.7 (60.51 to 98.29)	-1.18 (-1.35 to -1.01)
Cook Islands	G6PD trait	0 (0 to 0)	0 (0 to 0)	-0.36 (-0.37 to -0.35)	330.34 (297.17 to 363.11)	224.45 (202.56 to 244.39)	-1.46 (-1.56 to -1.35)
Costa Rica	**Inherited anemias**	14 (13 to 16)	11 (10 to 12)	-0.24 (-0.29 to -0.19)	472.53 (419.23 to 528.98)	230.93 (202.18 to 258.39)	-2.2 (-2.36 to -2.05)
Costa Rica	Thalassemias	0 (0 to 0)	0 (0 to 0)	0.38 (-0.18 to 2.02)	0.01 (0.01 to 0.02)	0.01 (0 to 0.01)	-0.26 (-0.4 to -0.12)
Costa Rica	Thalassemias trait	0 (0 to 0)	0 (0 to 0)	0.01 (-0.18 to 0.41)	6.55 (4.79 to 8.91)	4.28 (3.16 to 5.4)	-1.29 (-1.38 to -1.2)
Costa Rica	Sickle	0	0	-0.18	0.01	0	-2.24

Regions	Causes	Cases in 1990 No.×103 (95% UI)	Cases in 2019 No.×103 (95% UI)	Change in absolute number (%)	Rates in 1990 per 100000 (95% UI)	Rates in 2019 per 100000 (95% UI)	EAPC from 1990 and 2019 (95%CI)
	cell disorders	(0 to 0)	(0 to 0)	(−0.34 to 0.08)	(0 to 0.01)	(0 to 0.01)	(−2.72 to −1.76)
Costa Rica	Sickle cell trait	0 (0 to 0)	0 (0 to 0)	−0.2 (−0.46 to −0.18)	2.62 (1.18 to 4.12)	1.35 (0.63 to 2.15)	−2.38 (−3.03 to −1.73)
Costa Rica	G6PD deficiency	4 (3 to 5)	2 (2 to 3)	−0.46 (−0.53 to −0.38)	138.66 (112.31 to 167.23)	48.14 (37.32 to 59.7)	−3.19 (−3.5 to −2.89)
Costa Rica	G6PD trait	10 (9 to 11)	8 (8 to 9)	−0.15 (−0.21 to −0.11)	324.68 (296.36 to 353.9)	177.14 (159.11 to 193.39)	−1.88 (−2.08 to −1.67)
Côte d'Ivoire	**Inherited anemias**	241 (222 to 263)	384 (354 to 418)	0.59 (0.56 to 0.62)	1975.02 (1819.25 to 2148.72)	1467.09 (1353.87 to 1595.35)	−0.97 (−1.05 to −0.89)
Côte d'Ivoire	Thalassemias	0 (0 to 0)	1 (0 to 1)	0.64 (0.48 to 0.8)	2.84 (2.17 to 3.63)	2.17 (1.68 to 2.79)	−0.85 (−0.94 to −0.76)
Côte d'Ivoire	Thalassemias trait	29 (26 to 33)	48 (42 to 54)	0.63 (0.55 to 0.71)	240.87 (212.56 to 270.17)	183.15 (162.01 to 205.76)	−0.89 (−0.98 to −0.81)
Côte d'Ivoire	Sickle cell disorders	4 (3 to 4)	6 (5 to 7)	0.53 (0.46 to 0.61)	31.93 (27.9 to 36.18)	22.88 (19.88 to 25.96)	−1.1 (−1.18 to −1.01)
Côte d'Ivoire	Sickle cell trait	73 (68 to 79)	113 (104 to 122)	0.54 (0.49 to 0.59)	599.88 (555.56 to 646.89)	431.83 (398.12 to 467.91)	−1.08 (−1.16 to −0.99)
Côte d'Ivoire	G6PD deficiency	43 (33 to 54)	69 (53 to 87)	0.59 (0.56 to 0.63)	352.29 (272.18 to 437.69)	262.43 (202.87 to 331.01)	−0.89 (−0.99 to −0.78)
Côte d'Ivoire	G6PD trait	91 (83 to 99)	148 (134 to 161)	0.62 (0.58 to 0.65)	747.21 (676.98 to 813.74)	564.62 (513.68 to 614.69)	−0.95 (−1.02 to −0.88)
Croatia	**Inherited anemias**	8 (7 to 9)	5 (4 to 5)	−0.39 (−0.41 to −0.37)	156.61 (135.32 to 179.65)	110.46 (96.23 to 126.05)	−0.57 (−0.86 to −0.27)
Croatia	Thalassemias	0 (0 to 0)	0 (0 to 0)	−0.6 (−0.64 to −0.54)	0.8 (0.53 to 1.26)	0.37 (0.25 to 0.57)	−2.23 (−2.44 to −2.02)
Croatia	Thalassemias trait	1 (1 to 1)	1 (1 to 1)	−0.32 (−0.36 to −0.27)	19.62 (16.26 to 23.7)	15.46 (12.98 to 18.64)	−0.5 (−0.71 to −0.29)
Croatia	Sickle cell	0 (0 to 0)	0 (0 to 0)	−0.32 (−0.38 to	0.1 (0.08 to	0.08 (0.06 to	−0.59 (−0.85 to

Regions	Causes	Cases in 1990 No.×103 (95% UI)	Cases in 2019 No.×103 (95% UI)	Change in absolute number (%)	Rates in 1990 per 100000 (95% UI)	Rates in 2019 per 100000 (95% UI)	EAPC from 1990 and 2019 (95%CI)
	disorders			-0.26)	0.13)	0.1)	-0.33)
Croatia	Sickle cell trait	1 (1 to 1)	1 (1 to 1)	-0.31 (-0.34 to -0.28)	17.79 (15.23 to 20.49)	14.2 (12.22 to 16.36)	-0.49 (-0.72 to -0.26)
Croatia	G6PD deficiency	3 (2 to 4)	2 (1 to 2)	-0.47 (-0.52 to -0.42)	61.5 (47.54 to 77.49)	37.49 (28.86 to 46.57)	-0.63 (-1.09 to -0.17)
Croatia	G6PD trait	3 (2 to 3)	2 (2 to 2)	-0.35 (-0.35 to -0.35)	56.8 (49.68 to 64.89)	42.87 (37.49 to 48.97)	-0.53 (-0.76 to -0.3)
Cuba	**Inherited anemias**	32 (29 to 36)	20 (18 to 23)	-0.37 (-0.39 to -0.34)	297.17 (264.47 to 333.34)	179.56 (160.46 to 200.62)	-1.27 (-1.56 to -0.98)
Cuba	Thalassemias	0 (0 to 0)	0 (0 to 0)	-0.01 (-0.42 to 1.19)	0.01 (0 to 0.01)	0.01 (0 to 0.01)	0.14 (-0.18 to 0.45)
Cuba	Thalassemias trait	0 (0 to 1)	0 (0 to 0)	-0.28 (-0.42 to 0)	4.02 (2.95 to 5.41)	2.77 (2.07 to 3.44)	-0.86 (-1.11 to -0.6)
Cuba	Sickle cell disorders	0 (0 to 0)	0 (0 to 0)	0.11 (-0.05 to 0.3)	1.14 (0.96 to 1.35)	1.21 (0.92 to 1.55)	0.62 (-0.04 to 1.29)
Cuba	Sickle cell trait	8 (7 to 9)	6 (5 to 7)	-0.25 (-0.31 to -0.18)	72.28 (65.41 to 80.61)	52.03 (45 to 60.58)	-0.68 (-1.07 to -0.3)
Cuba	G6PD deficiency	7 (5 to 9)	4 (3 to 5)	-0.41 (-0.44 to -0.37)	64.96 (50.18 to 81.39)	36.71 (28.46 to 45.84)	-1.51 (-1.77 to -1.25)
Cuba	G6PD trait	17 (15 to 19)	10 (9 to 11)	-0.41 (-0.44 to -0.38)	154.77 (137.83 to 172.56)	86.85 (77.42 to 96.24)	-1.5 (-1.77 to -1.24)
Cyprus	**Inherited anemias**	5 (5 to 6)	5 (5 to 6)	0.05 (0.02 to 0.08)	658.64 (596.74 to 727.39)	408.55 (368.44 to 451.63)	-1.23 (-1.9 to -0.55)
Cyprus	Thalassemias	0 (0 to 0)	0 (0 to 0)	0.04 (-0.09 to 0.17)	4.42 (3.22 to 5.83)	2.72 (1.96 to 3.64)	-1.43 (-1.77 to -1.08)
Cyprus	Thalassemias trait	1 (1 to 2)	1 (1 to 2)	0.04 (-0.02 to 0.1)	182.59 (157.94 to 208.12)	112.81 (97.31 to 129.33)	-1.43 (-1.78 to -1.07)
Cyprus	Sickle cell disorders	0 (0 to 0)	0 (0 to 0)	0 (-0.1 to 0.12)	0.73 (0.52 to 0.99)	0.43 (0.31 to 0.58)	-1.46 (-1.77 to -1.15)

Regions	Causes	Cases in 1990 No.×103 (95% UI)	Cases in 2019 No.×103 (95% UI)	Change in absolute number (%)	Rates in 1990 per 100000 (95% UI)	Rates in 2019 per 100000 (95% UI)	EAPC from 1990 and 2019 (95%CI)
Cyprus	Sickle cell trait	0 (0 to 0)	0 (0 to 0)	-0.01 (-0.08 to 0.06)	41.73 (33.9 to 50.44)	24.37 (20.11 to 28.9)	-1.49 (-1.83 to -1.13)
Cyprus	G6PD deficiency	1 (1 to 1)	1 (1 to 1)	0.08 (0.01 to 0.15)	132.75 (102.77 to 164.65)	84.66 (65.54 to 106.09)	-0.89 (-2.1 to 0.33)
Cyprus	G6PD trait	2 (2 to 3)	2 (2 to 3)	0.05 (0.02 to 0.08)	296.41 (269.04 to 321.76)	183.56 (166.65 to 199.63)	-1.2 (-1.96 to -0.43)
Czechia	**Inherited anemias**	18 (16 to 20)	16 (14 to 18)	-0.12 (-0.14 to -0.1)	175.82 (155.23 to 197.76)	149.09 (131.13 to 168.97)	0.08 (-0.34 to 0.51)
Czechia	Thalassemias	0 (0 to 0)	0 (0 to 0)	-0.48 (-0.54 to -0.41)	0.9 (0.59 to 1.42)	0.45 (0.31 to 0.68)	-1.45 (-1.89 to -1.01)
Czechia	Thalassemias trait	2 (2 to 3)	2 (2 to 2)	-0.11 (-0.17 to -0.04)	21.87 (18.08 to 26.54)	18.89 (15.8 to 22.72)	0.33 (-0.1 to 0.76)
Czechia	Sickle cell disorders	0 (0 to 0)	0 (0 to 0)	-0.04 (-0.14 to 0.06)	0.09 (0.06 to 0.11)	0.08 (0.06 to 0.11)	0.49 (0.1 to 0.88)
Czechia	Sickle cell trait	2 (2 to 2)	2 (2 to 2)	-0.1 (-0.14 to -0.06)	19.96 (17.2 to 22.87)	17.39 (14.89 to 20.09)	0.35 (-0.07 to 0.76)
Czechia	G6PD deficiency	4 (3 to 6)	4 (3 to 5)	-0.08 (-0.15 to 0)	43.12 (33.36 to 53.47)	38.39 (29.67 to 48.65)	-0.52 (-1.04 to 0)
Czechia	G6PD trait	9 (8 to 10)	8 (7 to 9)	-0.15 (-0.15 to -0.15)	89.88 (79.3 to 100.74)	73.89 (65.19 to 82.81)	0.22 (-0.21 to 0.66)
Democratic People's Republic of Korea	**Inherited anemias**	164 (147 to 182)	76 (68 to 85)	-0.54 (-0.55 to -0.52)	779.55 (700.43 to 864.63)	290.11 (260.77 to 323.05)	-3.78 (-3.94 to -3.61)
Democratic People's Republic of Korea	Thalassemias	1 (1 to 1)	0 (0 to 1)	-0.54 (-0.6 to -0.47)	3.92 (2.87 to 5.27)	1.45 (1.03 to 2.01)	-3.77 (-3.94 to -3.6)
Democratic People's Republic of Korea	Thalassemias trait	42 (35 to 50)	21 (18 to 26)	-0.49 (-0.52 to -0.45)	198.87 (165.48 to 237.62)	81.63 (68.26 to 97.9)	-3.43 (-3.6 to -3.26)
Democratic	Sickle	0	0	-0.5	0.11	0.05	-3.21

Regions	Causes	Cases in 1990 No.×103 (95% UI)	Cases in 2019 No.×103 (95% UI)	Change in absolute number (%)	Rates in 1990 per 100000 (95% UI)	Rates in 2019 per 100000 (95% UI)	EAPC from 1990 and 2019 (95%CI)
People's Republic of Korea	cell disorders	(0 to 0)	(0 to 0)	(−0.57 to −0.43)	(0.08 to 0.16)	(0.03 to 0.06)	(−3.38 to −3.03)
Democratic People's Republic of Korea	Sickle cell trait	8 (7 to 10)	4 (3 to 5)	−0.53 (−0.56 to −0.49)	38.73 (32.54 to 46.24)	14.72 (12.09 to 17.49)	−3.53 (−3.67 to −3.39)
Democratic People's Republic of Korea	G6PD deficiency	28 (22 to 35)	13 (10 to 16)	−0.53 (−0.56 to −0.5)	132.79 (102.64 to 165.4)	49.62 (38.42 to 61.47)	−3.84 (−4.04 to −3.63)
Democratic People's Republic of Korea	G6PD trait	85 (77 to 94)	37 (33 to 41)	−0.56 (−0.58 to −0.55)	405.13 (363.97 to 444.56)	142.65 (127.39 to 156.98)	−3.97 (−4.13 to −3.8)
Democratic Republic of the Congo	**Inherited anemias**	1086 (986 to 1188)	1754 (1599 to 1923)	0.61 (0.56 to 0.68)	2815.29 (2555.37 to 3077.74)	2001.1 (1823.95 to 2193.21)	−1.1 (−1.25 to −0.94)
Democratic Republic of the Congo	Thalassemias	0 (0 to 0)	0 (0 to 0)	2.15 (1.75 to 2.74)	0.11 (0.07 to 0.17)	0.16 (0.09 to 0.24)	1.03 (0.82 to 1.24)
Democratic Republic of the Congo	Thalassemias trait	15 (13 to 18)	30 (24 to 35)	0.97 (0.84 to 1.13)	39.11 (32.58 to 46.51)	33.95 (27.94 to 40.43)	−0.46 (−0.61 to −0.3)
Democratic Republic of the Congo	Sickle cell disorders	30 (24 to 38)	55 (44 to 69)	0.8 (0.57 to 1.1)	78.92 (61.65 to 97.92)	62.68 (50.21 to 78.77)	−0.74 (−0.96 to −0.52)
Democratic Republic of the Congo	Sickle cell trait	405 (362 to 447)	673 (610 to 744)	0.66 (0.56 to 0.78)	1049.97 (937.47 to 1159.48)	767.53 (696.35 to 848.88)	−1 (−1.17 to −0.82)
Democratic Republic of the Congo	G6PD deficiency	260 (200 to 322)	411 (318 to 514)	0.58 (0.48 to 0.72)	672.49 (519.31 to 835.4)	468.64 (362.36 to 586.07)	−1.22 (−1.36 to −1.08)
Democratic Republic of the Congo	G6PD trait	376 (347 to 402)	586 (540 to 626)	0.56 (0.52 to 0.6)	974.69 (899.69 to 1041.09)	668.14 (615.43 to 714.13)	−1.18 (−1.34 to −1.03)
Denmark	**Inherited anemias**	12 (10 to 13)	11 (10 to 12)	−0.04 (−0.07 to −0.02)	224.44 (200.96 to 248.67)	190.6 (170.94 to 212.31)	−1.03 (−1.21 to −0.85)
Denmark	Thalassemias	0 (0 to 0)	0 (0 to 0)	−0.08 (−0.2 to 0.05)	0.16 (0.11 to 0.21)	0.13 (0.09 to 0.17)	−2 (−2.89 to −1.1)
Denmark	Thalassemi	1	1	0	19.85	17.51	−1.12

Regions	Causes	Cases in 1990 No.×103 (95% UI)	Cases in 2019 No.×103 (95% UI)	Change in absolute number (%)	Rates in 1990 per 100000 (95% UI)	Rates in 2019 per 100000 (95% UI)	EAPC from 1990 and 2019 (95%CI)
	as trait	(1 to 1)	(1 to 1)	(−0.07 to 0.06)	(17.16 to 22.55)	(15.28 to 20.11)	(−1.44 to −0.8)
Denmark	Sickle cell disorders	0<br>(0 to 0)	0<br>(0 to 0)	−0.22<br>(−0.31 to −0.12)	0.2<br>(0.14 to 0.29)	0.14<br>(0.1 to 0.19)	−1.6<br>(−2.05 to −1.14)
Denmark	Sickle cell trait	1<br>(1 to 2)	1<br>(1 to 2)	−0.15<br>(−0.2 to −0.09)	28.67<br>(23.14 to 34.98)	21.69<br>(17.91 to 26.11)	−1.35<br>(−1.66 to −1.05)
Denmark	G6PD deficiency	2<br>(1 to 2)	2<br>(1 to 2)	0.02<br>(−0.04 to 0.08)	33.75<br>(25.94 to 42)	30.52<br>(23.55 to 38.79)	−0.83<br>(−0.98 to −0.68)
Denmark	G6PD trait	7<br>(7 to 8)	7<br>(6 to 8)	−0.04<br>(−0.07 to 0)	141.8<br>(127.01 to 156.2)	120.62<br>(108.25 to 133.2)	−1<br>(−1.15 to −0.84)
Djibouti	**Inherited anemias**	7<br>(6 to 8)	10<br>(9 to 12)	0.46<br>(0.43 to 0.48)	1473.52<br>(1294.67 to 1650.57)	869.24<br>(768.56 to 971.13)	−1.64<br>(−1.98 to −1.29)
Djibouti	Thalassemias	0<br>(0 to 0)	0<br>(0 to 0)	2.55<br>(2.01 to 3.4)	0.09<br>(0.05 to 0.13)	0.13<br>(0.07 to 0.19)	1.17<br>(0.86 to 1.48)
Djibouti	Thalassemias trait	0<br>(0 to 0)	0<br>(0 to 0)	1.21<br>(1.02 to 1.48)	26.1<br>(20.58 to 32.09)	23.26<br>(18.43 to 28.43)	−0.31<br>(−0.57 to −0.05)
Djibouti	Sickle cell disorders	0<br>(0 to 0)	0<br>(0 to 0)	0.55<br>(0.4 to 0.69)	1.2<br>(0.88 to 1.64)	0.75<br>(0.54 to 1.03)	−2.75<br>(−3.31 to −2.19)
Djibouti	Sickle cell trait	1<br>(1 to 1)	1<br>(1 to 1)	0.57<br>(0.5 to 0.64)	142.39<br>(121.97 to 167.7)	90.44<br>(76.93 to 106.47)	−2.03<br>(−2.38 to −1.68)
Djibouti	G6PD deficiency	2<br>(2 to 3)	3<br>(2 to 4)	0.3<br>(0.23 to 0.37)	465.8<br>(358.94 to 577.42)	245.09<br>(188.28 to 307.7)	−1.96<br>(−2.43 to −1.49)
Djibouti	G6PD trait	4<br>(4 to 4)	6<br>(6 to 7)	0.51<br>(0.5 to 0.51)	837.94<br>(767.87 to 899.87)	509.57<br>(465.66 to 549.41)	−1.45<br>(−1.76 to −1.15)
Dominica	**Inherited anemias**	0<br>(0 to 0)	0<br>(0 to 0)	−0.48<br>(−0.5 to −0.45)	583.68<br>(515.83 to 660.33)	328.73<br>(289.57 to 372.25)	−2.29<br>(−2.39 to −2.18)
Dominica	Thalassemias	0<br>(0 to 0)	0<br>(0 to 0)	−0.2<br>(−0.52 to 0.55)	0.01<br>(0.01 to 0.02)	0.01<br>(0.01 to 0.01)	−0.9<br>(−1.13 to −0.67)
Dominica	Thalassemias trait	0<br>(0 to 0)	0<br>(0 to 0)	−0.43<br>(−0.52 to	8.62<br>(6.69 to	5.3<br>(4.15 to	−1.97<br>(−2.07 to

Regions	Causes	Cases in 1990 No.×103 (95% UI)	Cases in 2019 No.×103 (95% UI)	Change in absolute number (%)	Rates in 1990 per 100000 (95% UI)	Rates in 2019 per 100000 (95% UI)	EAPC from 1990 and 2019 (95%CI)
				-0.29)	10.96)	6.43)	-1.87)
Dominica	Sickle cell disorders	0 (0 to 0)	0 (0 to 0)	-0.28 (-0.38 to -0.16)	5.03 (3.74 to 6.6)	3.88 (2.74 to 5.22)	-1.49 (-1.77 to -1.21)
Dominica	Sickle cell trait	0 (0 to 0)	0 (0 to 0)	-0.39 (-0.43 to -0.33)	165.52 (140.09 to 192.5)	109.35 (90.64 to 130.15)	-1.89 (-2.07 to -1.7)
Dominica	G6PD deficiency	0 (0 to 0)	0 (0 to 0)	-0.52 (-0.57 to -0.47)	184.68 (142.66 to 229.12)	95.4 (73.42 to 117.54)	-2.51 (-2.62 to -2.41)
Dominica	G6PD trait	0 (0 to 0)	0 (0 to 0)	-0.52 (-0.53 to -0.49)	219.83 (196.42 to 242.27)	114.8 (102.38 to 128.14)	-2.48 (-2.59 to -2.36)
Dominican Republic	**Inherited anemias**	72 (64 to 80)	68 (61 to 76)	-0.05 (-0.07 to -0.02)	994.2 (888.68 to 1109.16)	627.57 (563.7 to 697.91)	-1.72 (-1.9 to -1.53)
Dominican Republic	Thalassemias	0 (0 to 0)	0 (0 to 0)	0.15 (-0.14 to 0.56)	0.05 (0.04 to 0.07)	0.04 (0.03 to 0.05)	-1.09 (-1.19 to -0.99)
Dominican Republic	Thalassemias trait	2 (2 to 2)	2 (2 to 2)	0.01 (-0.11 to 0.13)	28.87 (24.28 to 34.04)	19.25 (16.32 to 22.65)	-1.53 (-1.66 to -1.39)
Dominican Republic	Sickle cell disorders	1 (1 to 1)	1 (1 to 1)	-0.01 (-0.08 to 0.08)	15.98 (13.25 to 18.95)	10.51 (8.84 to 12.44)	-1.61 (-1.85 to -1.37)
Dominican Republic	Sickle cell trait	26 (23 to 29)	25 (23 to 28)	-0.03 (-0.07 to 0.03)	360.94 (322.16 to 399.98)	232.42 (209.57 to 256.52)	-1.66 (-1.86 to -1.45)
Dominican Republic	G6PD deficiency	19 (15 to 24)	18 (14 to 22)	-0.07 (-0.07 to -0.06)	267.4 (205.74 to 332.26)	165.46 (127.32 to 205.65)	-1.77 (-1.93 to -1.61)
Dominican Republic	G6PD trait	23 (21 to 26)	22 (19 to 24)	-0.06 (-0.1 to -0.02)	320.97 (284.97 to 356.46)	199.89 (178.05 to 221.77)	-1.76 (-1.93 to -1.59)
Ecuador	**Inherited anemias**	46 (42 to 51)	63 (56 to 71)	0.35 (0.31 to 0.41)	462.39 (416.65 to 510.78)	356.85 (316.75 to 401.21)	-1.35 (-1.48 to -1.23)
Ecuador	Thalassemias	0 (0 to 0)	0 (0 to 0)	0.64 (0.26 to 1.13)	0.05 (0.03 to 0.06)	0.04 (0.03 to 0.06)	-0.19 (-0.34 to -0.05)
Ecuador	Thalassemias trait	2 (2 to 3)	3 (3 to 4)	0.34 (0.2 to 0.51)	24.06 (20.83 to 27.76)	18.42 (15.92 to 21.13)	-0.96 (-1.02 to -0.9)

Regions	Causes	Cases in 1990 No.×103 (95% UI)	Cases in 2019 No.×103 (95% UI)	Change in absolute number (%)	Rates in 1990 per 100000 (95% UI)	Rates in 2019 per 100000 (95% UI)	EAPC from 1990 and 2019 (95%CI)
Ecuador	Sickle cell disorders	0 (0 to 0)	0 (0 to 0)	0.91 (0.71 to 1.12)	0.13 (0.11 to 0.16)	0.14 (0.11 to 0.17)	0.19 (0.1 to 0.28)
Ecuador	Sickle cell trait	4 (3 to 4)	6 (5 to 6)	0.52 (0.44 to 0.59)	37.27 (33.32 to 41.82)	32.27 (28.89 to 36.11)	-0.58 (-0.63 to -0.54)
Ecuador	G6PD deficiency	5 (4 to 6)	14 (11 to 17)	1.9 (1.7 to 2.13)	47.46 (36.64 to 60.35)	78.54 (61.71 to 98.21)	-0.75 (-1.46 to -0.04)
Ecuador	G6PD trait	35 (32 to 39)	40 (36 to 44)	0.13 (0.09 to 0.18)	353.44 (317.29 to 391.23)	227.43 (204.37 to 251.44)	-1.6 (-1.65 to -1.55)
Egypt	**Inherited anemias**	648 (605 to 698)	686 (633 to 742)	0.06 (0.03 to 0.09)	1164.09 (1086.77 to 1253.66)	692.15 (639.2 to 749.44)	-1.22 (-1.42 to -1.02)
Egypt	Thalassemias	1 (1 to 1)	1 (1 to 1)	0.16 (0.05 to 0.28)	1.48 (1.17 to 1.93)	0.97 (0.76 to 1.21)	-1.07 (-1.23 to -0.91)
Egypt	Thalassemias trait	86 (77 to 97)	98 (87 to 110)	0.14 (0.09 to 0.19)	154.22 (138.31 to 174.66)	98.66 (88.17 to 110.7)	-1.13 (-1.28 to -0.97)
Egypt	Sickle cell disorders	3 (3 to 4)	3 (3 to 4)	0.07 (-0.01 to 0.15)	5.7 (4.74 to 6.9)	3.42 (2.88 to 4.13)	-1.31 (-1.46 to -1.16)
Egypt	Sickle cell trait	150 (138 to 165)	161 (148 to 177)	0.07 (0.04 to 0.11)	269.75 (247.13 to 296.28)	162.93 (149.59 to 178.58)	-1.3 (-1.45 to -1.15)
Egypt	G6PD deficiency	96 (81 to 113)	81 (62 to 100)	-0.16 (-0.27 to -0.07)	173.03 (145.38 to 202.31)	81.59 (62.65 to 101.21)	-1.13 (-1.68 to -0.58)
Egypt	G6PD trait	312 (284 to 339)	341 (310 to 370)	0.09 (0.07 to 0.13)	559.92 (509.67 to 608.07)	344.59 (312.8 to 373.19)	-1.23 (-1.38 to -1.08)
El Salvador	**Inherited anemias**	29 (26 to 33)	17 (15 to 19)	-0.41 (-0.43 to -0.39)	558.82 (491.77 to 624.1)	277.4 (244.06 to 310.55)	-2.65 (-2.85 to -2.46)
El Salvador	Thalassemias	0 (0 to 0)	0 (0 to 0)	0.16 (-0.32 to 1.39)	0.01 (0.01 to 0.02)	0.01 (0.01 to 0.02)	-0.36 (-0.56 to -0.15)
El Salvador	Thalassemias trait	0 (0 to 1)	0 (0 to 0)	-0.15 (-0.32 to 0.17)	8.46 (6.09 to 11.46)	6.04 (4.49 to 7.74)	-1.41 (-1.62 to -1.21)
El Salvador	Sickle	0	0	-0.33	0.07	0.04	-2.19

Regions	Causes	Cases in 1990 No.×103 (95% UI)	Cases in 2019 No.×103 (95% UI)	Change in absolute number (%)	Rates in 1990 per 100000 (95% UI)	Rates in 2019 per 100000 (95% UI)	EAPC from 1990 and 2019 (95%CI)
	cell disorders	(0 to 0)	(0 to 0)	(-0.41 to -0.24)	(0.03 to 0.11)	(0.02 to 0.06)	(-2.48 to -1.91)
El Salvador	Sickle cell trait	0 (0 to 0)	0 (0 to 0)	-0.34 (-0.45 to -0.32)	3.48 (1.41 to 6.08)	1.93 (0.8 to 3.07)	-2.12 (-2.37 to -1.87)
El Salvador	G6PD deficiency	6 (5 to 7)	3 (2 to 4)	-0.49 (-0.51 to -0.45)	111.49 (85.92 to 137.92)	47.87 (36.91 to 60.33)	-3.1 (-3.27 to -2.92)
El Salvador	G6PD trait	23 (21 to 25)	14 (12 to 15)	-0.4 (-0.42 to -0.37)	435.31 (392.4 to 475.44)	221.51 (198.94 to 242.49)	-2.58 (-2.8 to -2.36)
Equatorial Guinea	**Inherited anemias**	11 (10 to 12)	19 (18 to 20)	0.65 (0.55 to 0.76)	2640.52 (2426.37 to 2869.86)	1322.2 (1247.2 to 1406.4)	-2.47 (-2.63 to -2.31)
Equatorial Guinea	Thalassemias	0 (0 to 0)	0 (0 to 0)	2.4 (1.88 to 3)	0.13 (0.08 to 0.18)	0.13 (0.07 to 0.2)	-0.34 (-0.58 to -0.09)
Equatorial Guinea	Thalassemias trait	0 (0 to 0)	0 (0 to 0)	1.08 (0.85 to 1.25)	42.94 (35.86 to 50.85)	27.06 (22.03 to 31.98)	-1.87 (-2.04 to -1.69)
Equatorial Guinea	Sickle cell disorders	1 (0 to 1)	1 (1 to 1)	0.65 (0.53 to 0.79)	126.34 (100.42 to 156.53)	63.25 (50.87 to 78.56)	-2.26 (-2.45 to -2.07)
Equatorial Guinea	Sickle cell trait	5 (4 to 5)	8 (7 to 9)	0.64 (0.58 to 0.72)	1099.69 (987.53 to 1237.01)	547.22 (488.85 to 612.39)	-2.38 (-2.55 to -2.22)
Equatorial Guinea	G6PD deficiency	2 (1 to 2)	3 (2 to 3)	0.62 (0.31 to 1.08)	375.44 (289.39 to 472.07)	184.3 (170.53 to 198.59)	-2.66 (-2.84 to -2.47)
Equatorial Guinea	G6PD trait	4 (4 to 5)	7 (7 to 7)	0.66 (0.55 to 0.79)	995.98 (913.7 to 1076.37)	500.25 (485.34 to 515.57)	-2.55 (-2.72 to -2.38)
Eritrea	**Inherited anemias**	44 (39 to 50)	60 (53 to 66)	0.34 (0.31 to 0.38)	1480.77 (1305.66 to 1658.81)	889.52 (792.39 to 989.47)	-1.77 (-1.93 to -1.61)
Eritrea	Thalassemias	0 (0 to 0)	0 (0 to 0)	2.29 (1.81 to 3.03)	0.09 (0.05 to 0.13)	0.13 (0.07 to 0.2)	0.96 (0.74 to 1.19)
Eritrea	Thalassemias trait	1 (1 to 1)	2 (1 to 2)	1.03 (0.87 to 1.28)	26.11 (20.72 to 31.93)	23.72 (18.8 to 28.97)	-0.52 (-0.64 to -0.4)
Eritrea	Sickle cell	0 (0 to 0)	0 (0 to 0)	0.47 (0.33 to	1.59 (1.13 to	1.04 (0.74 to	-1.5 (-1.57 to

Regions	Causes	Cases in 1990 No.×103 (95% UI)	Cases in 2019 No.×103 (95% UI)	Change in absolute number (%)	Rates in 1990 per 100000 (95% UI)	Rates in 2019 per 100000 (95% UI)	EAPC from 1990 and 2019 (95%CI)
	disorders			0.62)	2.24)	1.44)	-1.43)
Eritrea	Sickle cell trait	5 (4 to 6)	7 (6 to 8)	0.47 (0.39 to 0.54)	163.04 (136.92 to 194.52)	106.86 (89.52 to 126.45)	-1.5 (-1.57 to -1.43)
Eritrea	G6PD deficiency	14 (11 to 17)	16 (13 to 21)	0.19 (0.12 to 0.26)	456.52 (351.39 to 567.72)	242.97 (188.06 to 305.85)	-2.19 (-2.48 to -1.9)
Eritrea	G6PD trait	25 (23 to 27)	35 (32 to 37)	0.38 (0.34 to 0.42)	833.42 (762.18 to 897.05)	514.8 (470.86 to 555.58)	-1.66 (-1.79 to -1.54)
Estonia	**Inherited anemias**	3 (2 to 3)	2 (1 to 2)	-0.36 (-0.37 to -0.35)	165.66 (147.15 to 185.66)	126.82 (112.47 to 142.34)	0.21 (-0.22 to 0.65)
Estonia	Thalassemias	0 (0 to 0)	0 (0 to 0)	-0.57 (-0.62 to -0.5)	0.66 (0.45 to 1)	0.34 (0.24 to 0.48)	-1.08 (-1.52 to -0.64)
Estonia	Thalassemias trait	0 (0 to 1)	0 (0 to 0)	-0.35 (-0.39 to -0.3)	31.52 (26.08 to 38.14)	24.49 (20.44 to 29.8)	0.27 (-0.16 to 0.69)
Estonia	Sickle cell disorders	0 (0 to 0)	0 (0 to 0)	-0.34 (-0.45 to -0.19)	0.01 (0.01 to 0.01)	0.01 (0 to 0.01)	0.28 (-0.14 to 0.71)
Estonia	Sickle cell trait	0 (0 to 0)	0 (0 to 0)	-0.36 (-0.42 to -0.28)	6.26 (5.07 to 7.74)	4.81 (3.89 to 5.92)	0.26 (-0.17 to 0.69)
Estonia	G6PD deficiency	0 (0 to 1)	0 (0 to 0)	-0.32 (-0.32 to -0.32)	25.95 (19.87 to 32.31)	20.99 (16.07 to 26.13)	0.1 (-0.35 to 0.55)
Estonia	G6PD trait	2 (1 to 2)	1 (1 to 1)	-0.37 (-0.37 to -0.37)	101.27 (90.12 to 113.15)	76.17 (67.79 to 85.11)	0.23 (-0.21 to 0.68)
Eswatini	**Inherited anemias**	9 (8 to 10)	8 (7 to 9)	-0.1 (-0.12 to -0.07)	1140.83 (997.98 to 1294.1)	725.27 (636.76 to 819.29)	-1.4 (-1.52 to -1.28)
Eswatini	Thalassemias	0 (0 to 0)	0 (0 to 0)	0.96 (0.69 to 1.3)	0.09 (0.06 to 0.13)	0.12 (0.07 to 0.19)	1.01 (0.85 to 1.16)
Eswatini	Thalassemias trait	0 (0 to 0)	0 (0 to 0)	0.2 (0.1 to 0.31)	31.03 (25.78 to 37.1)	26.22 (21.51 to 31.13)	-0.53 (-0.61 to -0.45)
Eswatini	Sickle cell disorders	0 (0 to 0)	0 (0 to 0)	-0.06 (-0.13 to 0.01)	0.15 (0.1 to 0.22)	0.1 (0.07 to 0.15)	-1.31 (-1.39 to -1.23)

Regions	Causes	Cases in 1990 No.×103 (95% UI)	Cases in 2019 No.×103 (95% UI)	Change in absolute number (%)	Rates in 1990 per 100000 (95% UI)	Rates in 2019 per 100000 (95% UI)	EAPC from 1990 and 2019 (95%CI)
Eswatini	Sickle cell trait	0 (0 to 0)	0 (0 to 0)	-0.06 (-0.1 to -0.02)	41.34 (32.16 to 53.03)	27.31 (21.34 to 34.81)	-1.31 (-1.39 to -1.23)
Eswatini	G6PD deficiency	3 (2 to 4)	3 (2 to 3)	-0.14 (-0.18 to -0.09)	363.3 (280.83 to 456.72)	221.76 (171.2 to 276.15)	-1.55 (-1.7 to -1.39)
Eswatini	G6PD trait	6 (5 to 6)	5 (5 to 6)	-0.1 (-0.12 to -0.06)	704.91 (644.13 to 761.16)	449.76 (410.34 to 487.11)	-1.37 (-1.48 to -1.27)
Ethiopia	**Inherited anemias**	786 (762 to 810)	1067 (1038 to 1100)	0.36 (0.35 to 0.37)	1528.58 (1483.7 to 1576.91)	992.13 (964.53 to 1022.7)	-1.36 (-1.46 to -1.26)
Ethiopia	Thalassemias	0 (0 to 1)	1 (1 to 1)	0.65 (0.51 to 0.83)	0.95 (0.63 to 1.43)	0.75 (0.51 to 1.06)	-0.94 (-1 to -0.87)
Ethiopia	Thalassemias trait	75 (62 to 91)	119 (99 to 142)	0.59 (0.53 to 0.64)	145.96 (120.41 to 177.2)	110.59 (92.15 to 131.65)	-1.05 (-1.11 to -0.99)
Ethiopia	Sickle cell disorders	0 (0 to 0)	0 (0 to 0)	0.5 (0.45 to 0.58)	0.42 (0.28 to 0.63)	0.3 (0.2 to 0.45)	-1.21 (-1.27 to -1.15)
Ethiopia	Sickle cell trait	42 (33 to 53)	63 (50 to 80)	0.5 (0.46 to 0.53)	81.84 (64.37 to 102.75)	58.49 (46.1 to 74.38)	-1.22 (-1.27 to -1.16)
Ethiopia	G6PD deficiency	209 (199 to 218)	199 (190 to 208)	-0.05 (-0.06 to -0.03)	406.27 (387.46 to 424.1)	185.32 (176.61 to 193.46)	-1.93 (-2.24 to -1.63)
Ethiopia	G6PD trait	459 (452 to 465)	685 (674 to 695)	0.49 (0.49 to 0.5)	893.14 (879.68 to 905.08)	636.69 (626.79 to 645.81)	-1.19 (-1.24 to -1.14)
Fiji	**Inherited anemias**	4 (3 to 4)	4 (3 to 4)	0.03 (0 to 0.06)	474.01 (425.64 to 526.15)	408.34 (367.41 to 451.61)	-0.14 (-0.33 to 0.05)
Fiji	Thalassemias	0 (0 to 0)	0 (0 to 0)	-0.15 (-0.27 to -0.01)	2.85 (1.57 to 4.24)	2.03 (1.13 to 3.08)	0.28 (-0.73 to 1.31)
Fiji	Thalassemias trait	1 (0 to 1)	1 (0 to 1)	-0.09 (-0.15 to -0.03)	80.44 (61.3 to 97.75)	60.92 (46.61 to 74.77)	-0.15 (-0.62 to 0.33)
Fiji	Sickle cell disorders	0 (0 to 0)	0 (0 to 0)	-0.05 (-0.07 to 0.29)	0 (0 to 0)	0 (0 to 0)	-0.36 (-0.62 to -0.11)
Fiji	Sickle	0	0	-0.06	3.43	2.7	-0.44

Regions	Causes	Cases in 1990 No.×103 (95% UI)	Cases in 2019 No.×103 (95% UI)	Change in absolute number (%)	Rates in 1990 per 100000 (95% UI)	Rates in 2019 per 100000 (95% UI)	EAPC from 1990 and 2019 (95%CI)
	cell trait	(0 to 0)	(0 to 0)	(-0.07 to 0.2)	(1.43 to 5.17)	(1.1 to 4.24)	(-0.65 to -0.23)
Fiji	G6PD deficiency	0 (0 to 1)	1 (0 to 1)	0.17 (0.13 to 0.21)	59.56 (46.07 to 74.12)	57.9 (44.86 to 71.65)	0.19 (-0.04 to 0.42)
Fiji	G6PD trait	2 (2 to 3)	3 (2 to 3)	0.04 (0 to 0.07)	327.73 (294.9 to 360.31)	284.78 (258.23 to 309.22)	-0.22 (-0.34 to -0.09)
Finland	**Inherited anemias**	12 (11 to 13)	9 (8 to 10)	-0.27 (-0.29 to -0.25)	236.56 (213.1 to 262.05)	156.09 (139.83 to 173.22)	-1.19 (-1.4 to -0.98)
Finland	Thalassemias	0 (0 to 0)	0 (0 to 0)	-0.31 (-0.4 to -0.21)	0.17 (0.11 to 0.23)	0.1 (0.07 to 0.14)	-2.22 (-2.9 to -1.54)
Finland	Thalassemias trait	1 (1 to 1)	1 (1 to 1)	-0.24 (-0.29 to -0.19)	21.05 (18.18 to 24.2)	14.44 (12.46 to 16.62)	-1.29 (-1.46 to -1.11)
Finland	Sickle cell disorders	0 (0 to 0)	0 (0 to 0)	-0.47 (-0.54 to -0.41)	0.24 (0.17 to 0.33)	0.11 (0.08 to 0.15)	-1.88 (-2.5 to -1.25)
Finland	Sickle cell trait	2 (1 to 2)	1 (1 to 1)	-0.4 (-0.44 to -0.36)	31.18 (25.74 to 37.77)	16.98 (14.03 to 20.16)	-1.59 (-2.06 to -1.12)
Finland	G6PD deficiency	2 (1 to 2)	1 (1 to 2)	-0.21 (-0.26 to -0.17)	34.68 (26.92 to 43.19)	24.72 (19.06 to 31.24)	-0.97 (-1.16 to -0.78)
Finland	G6PD trait	7 (7 to 8)	6 (5 to 6)	-0.26 (-0.28 to -0.24)	149.25 (133.59 to 165.05)	99.74 (89.31 to 110.25)	-1.15 (-1.36 to -0.94)
France	**Inherited anemias**	120 (109 to 131)	98 (89 to 108)	-0.18 (-0.2 to -0.16)	207.56 (189.48 to 225.97)	148.11 (134.71 to 162.69)	-0.79 (-0.96 to -0.62)
France	Thalassemias	0 (0 to 0)	0 (0 to 0)	-0.12 (-0.25 to 0.01)	0.17 (0.11 to 0.23)	0.13 (0.09 to 0.18)	-1.54 (-2.39 to -0.69)
France	Thalassemias trait	12 (11 to 14)	12 (10 to 13)	-0.05 (-0.11 to 0.01)	21.25 (18.58 to 24.1)	17.7 (15.46 to 20.09)	-0.63 (-0.91 to -0.35)
France	Sickle cell disorders	1 (0 to 1)	0 (0 to 0)	-0.62 (-0.66 to -0.56)	1 (0.84 to 1.2)	0.34 (0.27 to 0.42)	-2.35 (-2.75 to -1.96)
France	Sickle cell trait	34 (30 to 38)	19 (16 to 22)	-0.44 (-0.49 to	58.59 (52.63 to	28.56 (24.85 to	-1.62 (-1.9 to

Regions	Causes	Cases in 1990 No.×103 (95% UI)	Cases in 2019 No.×103 (95% UI)	Change in absolute number (%)	Rates in 1990 per 100000 (95% UI)	Rates in 2019 per 100000 (95% UI)	EAPC from 1990 and 2019 (95%CI)
				-0.4)	65.93)	32.71)	-1.34)
France	G6PD deficiency	13 (10 to 16)	10 (8 to 12)	-0.24 (-0.29 to -0.18)	22.06 (17.05 to 27.45)	14.69 (11.44 to 18.45)	-1.17 (-1.55 to -0.8)
France	G6PD trait	60 (54 to 67)	57 (51 to 64)	-0.05 (-0.09 to -0.02)	104.48 (92.99 to 116.25)	86.69 (77.11 to 96.33)	-0.34 (-0.48 to -0.2)
Gabon	**Inherited anemias**	22 (20 to 24)	23 (21 to 25)	0.06 (0.03 to 0.1)	2172.28 (1975.65 to 2381.62)	1309.12 (1178.53 to 1444.6)	-1.74 (-1.8 to -1.68)
Gabon	Thalassemias	0 (0 to 0)	0 (0 to 0)	1.11 (0.77 to 1.53)	0.08 (0.05 to 0.12)	0.1 (0.06 to 0.15)	0.35 (0.2 to 0.51)
Gabon	Thalassemias trait	0 (0 to 0)	0 (0 to 0)	0.35 (0.23 to 0.48)	29.87 (25.17 to 35.16)	22.77 (18.68 to 27.1)	-1.07 (-1.13 to -1.01)
Gabon	Sickle cell disorders	1 (0 to 1)	1 (0 to 1)	-0.03 (-0.1 to 0.06)	61.27 (47.24 to 77.78)	33.81 (26.2 to 42.34)	-2.06 (-2.12 to -2.01)
Gabon	Sickle cell trait	6 (5 to 7)	6 (5 to 7)	0.03 (-0.02 to 0.08)	590.58 (526.82 to 671.28)	345 (309.54 to 390.68)	-1.65 (-1.79 to -1.51)
Gabon	G6PD deficiency	6 (5 to 8)	7 (5 to 8)	0.1 (0.01 to 0.2)	619.8 (495.75 to 763.43)	385.74 (299.24 to 477.1)	-1.71 (-1.75 to -1.66)
Gabon	G6PD trait	9 (8 to 9)	9 (9 to 10)	0.06 (0.04 to 0.08)	870.68 (810.53 to 920.18)	521.71 (485.84 to 553)	-1.83 (-1.86 to -1.8)
Gambia	**Inherited anemias**	18 (16 to 19)	24 (22 to 26)	0.35 (0.31 to 0.38)	1787.19 (1633.76 to 1951.13)	1062.23 (973.37 to 1152.3)	-1.89 (-2.07 to -1.71)
Gambia	Thalassemias	0 (0 to 0)	0 (0 to 0)	0.9 (0.63 to 1.23)	0.35 (0.24 to 0.51)	0.3 (0.2 to 0.41)	-0.68 (-0.8 to -0.55)
Gambia	Thalassemias trait	1 (1 to 1)	1 (1 to 2)	0.61 (0.5 to 0.71)	92.07 (76.38 to 111.52)	65.58 (54.73 to 78.45)	-1.25 (-1.37 to -1.12)
Gambia	Sickle cell disorders	0 (0 to 0)	0 (0 to 1)	0.31 (0.2 to 0.42)	33.18 (27.36 to 40)	19.23 (15.52 to 23.67)	-2.08 (-2.25 to -1.92)
Gambia	Sickle cell trait	7 (6 to 7)	9 (8 to 11)	0.41 (0.34 to 0.48)	676.78 (613.5 to 750.46)	421.1 (377.5 to 469.59)	-1.74 (-1.9 to -1.59)

Regions	Causes	Cases in 1990 No.×103 (95% UI)	Cases in 2019 No.×103 (95% UI)	Change in absolute number (%)	Rates in 1990 per 100000 (95% UI)	Rates in 2019 per 100000 (95% UI)	EAPC from 1990 and 2019 (95%CI)
Gambia	G6PD deficiency	3 (2 to 4)	3 (2 to 3)	-0.11 (-0.17 to -0.05)	290.29 (224.5 to 359.25)	114.17 (88.48 to 141.72)	-3.34 (-3.82 to -2.86)
Gambia	G6PD trait	7 (6 to 8)	10 (9 to 11)	0.44 (0.4 to 0.49)	694.52 (625.12 to 758.98)	441.86 (397.64 to 482.79)	-1.61 (-1.73 to -1.49)
Georgia	Inherited anemias	12 (11 to 13)	7 (6 to 8)	-0.41 (-0.42 to -0.39)	220.42 (198.45 to 244.32)	196.3 (176.72 to 218.03)	0.81 (0.33 to 1.29)
Georgia	Thalassemias	0 (0 to 0)	0 (0 to 0)	-0.41 (-0.49 to -0.31)	1.83 (1.35 to 2.52)	1.62 (1.18 to 2.21)	0.92 (0.42 to 1.43)
Georgia	Thalassemias trait	4 (4 to 5)	3 (2 to 3)	-0.36 (-0.4 to -0.32)	77.58 (65.3 to 92.17)	74.83 (63.5 to 87.94)	1.09 (0.63 to 1.56)
Georgia	Sickle cell disorders	0 (0 to 0)	0 (0 to 0)	-0.42 (-0.52 to -0.28)	0.01 (0.01 to 0.01)	0.01 (0 to 0.01)	0.72 (0.26 to 1.18)
Georgia	Sickle cell trait	0 (0 to 0)	0 (0 to 0)	-0.43 (-0.49 to -0.36)	6.56 (5.21 to 8.28)	5.66 (4.51 to 7.06)	0.65 (0.19 to 1.11)
Georgia	G6PD deficiency	1 (1 to 1)	1 (0 to 1)	-0.42 (-0.46 to -0.39)	20.38 (15.66 to 25.96)	17.66 (13.6 to 22.31)	0.69 (0.23 to 1.15)
Georgia	G6PD trait	6 (6 to 7)	4 (3 to 4)	-0.44 (-0.44 to -0.44)	114.06 (101.76 to 126.84)	96.52 (86.11 to 107.34)	0.63 (0.13 to 1.14)
Germany	Inherited anemias	173 (157 to 190)	149 (135 to 164)	-0.14 (-0.17 to -0.12)	216.79 (196.03 to 238.24)	175.25 (158.44 to 192.89)	-0.66 (-0.84 to -0.48)
Germany	Thalassemias	0 (0 to 1)	0 (0 to 0)	-0.17 (-0.34 to 0.02)	0.55 (0.39 to 0.74)	0.43 (0.32 to 0.55)	-1.64 (-2.52 to -0.75)
Germany	Thalassemias trait	33 (28 to 38)	32 (28 to 37)	-0.03 (-0.1 to 0.04)	41.67 (35.59 to 48.07)	38.02 (32.95 to 43.39)	-0.43 (-0.79 to -0.07)
Germany	Sickle cell disorders	0 (0 to 0)	0 (0 to 0)	-0.01 (-0.17 to 0.19)	0.09 (0.06 to 0.12)	0.08 (0.07 to 0.1)	1.22 (0.73 to 1.71)
Germany	Sickle cell trait	14 (11 to 16)	12 (10 to 13)	-0.15 (-0.23 to -0.05)	17.22 (14.25 to 20.44)	13.85 (12.2 to 15.66)	0.12 (-0.21 to 0.45)
Germany	G6PD	24	21	-0.13	30.43	24.95	-0.66

Regions	Causes	Cases in 1990 No.×103 (95% UI)	Cases in 2019 No.×103 (95% UI)	Change in absolute number (%)	Rates in 1990 per 100000 (95% UI)	Rates in 2019 per 100000 (95% UI)	EAPC from 1990 and 2019 (95%CI)
	deficiency	(19 to 30)	(16 to 26)	(-0.16 to -0.09)	(23.39 to 37.99)	(19.24 to 30.96)	(-0.9 to -0.43)
Germany	G6PD trait	101 (91 to 111)	83 (75 to 91)	-0.18 (-0.21 to -0.15)	126.83 (113.86 to 139.1)	97.93 (87.76 to 107.11)	-0.83 (-1.02 to -0.63)
Ghana	**Inherited anemias**	300 (273 to 327)	401 (365 to 439)	0.34 (0.29 to 0.39)	1996.15 (1815.77 to 2179.78)	1271.75 (1158.04 to 1392.48)	-1.03 (-1.24 to -0.82)
Ghana	Thalassemias	0 (0 to 0)	0 (0 to 0)	1.46 (1 to 1.92)	0.12 (0.08 to 0.16)	0.14 (0.08 to 0.2)	0.47 (0.36 to 0.58)
Ghana	Thalassemias trait	6 (5 to 8)	11 (9 to 13)	0.67 (0.54 to 0.8)	43.19 (36.27 to 51.06)	34.34 (28.61 to 40.45)	-0.77 (-0.86 to -0.69)
Ghana	Sickle cell disorders	12 (11 to 13)	16 (13 to 19)	0.34 (0.18 to 0.51)	78.63 (72 to 86.92)	50.11 (42.77 to 58.86)	-1.41 (-1.53 to -1.3)
Ghana	Sickle cell trait	113 (105 to 121)	154 (137 to 173)	0.36 (0.26 to 0.47)	751.01 (702.46 to 808.59)	487.51 (434.26 to 547.65)	-1.36 (-1.47 to -1.26)
Ghana	G6PD deficiency	71 (55 to 87)	82 (63 to 102)	0.16 (0.07 to 0.25)	469.68 (363.72 to 580.6)	258.85 (200.16 to 322.33)	-0.36 (-0.96 to 0.23)
Ghana	G6PD trait	98 (89 to 107)	139 (126 to 151)	0.42 (0.37 to 0.45)	653.52 (592.73 to 710.5)	440.81 (400.64 to 478.1)	-1.01 (-1.2 to -0.81)
Greece	**Inherited anemias**	21 (21 to 22)	18 (16 to 19)	-0.17 (-0.25 to -0.1)	206.35 (198.4 to 215.22)	171.26 (155.22 to 188.52)	-0.38 (-0.77 to 0)
Greece	Thalassemias	0 (0 to 0)	0 (0 to 0)	-0.16 (-0.25 to -0.06)	0.73 (0.55 to 0.93)	0.62 (0.46 to 0.8)	-0.38 (-0.63 to -0.12)
Greece	Thalassemias trait	6 (5 to 6)	5 (4 to 6)	-0.15 (-0.2 to -0.1)	55.25 (48.06 to 62.24)	47.19 (40.87 to 53.52)	-0.26 (-0.48 to -0.03)
Greece	Sickle cell disorders	0 (0 to 0)	0 (0 to 0)	-0.09 (-0.17 to 0.01)	0.09 (0.06 to 0.13)	0.09 (0.06 to 0.12)	0.04 (-0.13 to 0.22)
Greece	Sickle cell trait	1 (1 to 1)	1 (1 to 1)	0.04 (-0.04 to 0.13)	7.94 (6.9 to 9.19)	8.33 (7.02 to 9.81)	0.49 (0.35 to 0.64)
Greece	G6PD deficiency	3 (3 to 3)	3 (2 to 3)	-0.23 (-0.39 to	31.34 (30.2 to	24.29 (18.86 to	-0.04 (-0.83 to

Regions	Causes	Cases in 1990 No.×103 (95% UI)	Cases in 2019 No.×103 (95% UI)	Change in absolute number (%)	Rates in 1990 per 100000 (95% UI)	Rates in 2019 per 100000 (95% UI)	EAPC from 1990 and 2019 (95%CI)
				-0.04)	32.7)	30.42)	0.77)
Greece	G6PD trait	12 (11 to 12)	9 (8 to 10)	-0.19 (-0.27 to -0.11)	110.98 (108.13 to 114.24)	90.73 (81.21 to 100.3)	-0.63 (-1.02 to -0.23)
Greenland	**Inherited anemias**	0 (0 to 0)	0 (0 to 0)	-0.47 (-0.49 to -0.45)	390.39 (338.56 to 441.65)	203.64 (175.95 to 234.35)	-2.28 (-2.42 to -2.14)
Greenland	Thalassemias	0 (0 to 0)	0 (0 to 0)	-0.62 (-0.66 to -0.58)	0.27 (0.16 to 0.42)	0.1 (0.06 to 0.16)	-3.31 (-3.56 to -3.07)
Greenland	Thalassemias trait	0 (0 to 0)	0 (0 to 0)	-0.24 (-0.37 to 0.19)	4.23 (2.45 to 5.97)	3.19 (2.16 to 4.31)	-0.93 (-1.18 to -0.68)
Greenland	Sickle cell disorders	0 (0 to 0)	0 (0 to 0)	-0.37 (-0.45 to -0.3)	0.08 (0.05 to 0.12)	0.05 (0.03 to 0.08)	-1.63 (-1.87 to -1.38)
Greenland	Sickle cell trait	0 (0 to 0)	0 (0 to 0)	-0.37 (-0.37 to -0.26)	3.31 (1.41 to 5.83)	2.08 (0.88 to 3.61)	-1.61 (-1.85 to -1.37)
Greenland	G6PD deficiency	0 (0 to 0)	0 (0 to 0)	-0.46 (-0.49 to -0.42)	103.33 (79.74 to 128.51)	55.65 (43.11 to 69.48)	-2.23 (-2.38 to -2.08)
Greenland	G6PD trait	0 (0 to 0)	0 (0 to 0)	-0.48 (-0.5 to -0.46)	279.18 (250.32 to 305.53)	142.58 (127.02 to 158.85)	-2.33 (-2.47 to -2.2)
Grenada	**Inherited anemias**	1 (0 to 1)	0 (0 to 0)	-0.26 (-0.29 to -0.23)	611.7 (536.95 to 692.22)	375.36 (331.23 to 424.73)	-1.86 (-1.93 to -1.78)
Grenada	Thalassemias	0 (0 to 0)	0 (0 to 0)	0.17 (-0.29 to 1.34)	0.01 (0.01 to 0.02)	0.01 (0.01 to 0.02)	-0.33 (-0.57 to -0.09)
Grenada	Thalassemias trait	0 (0 to 0)	0 (0 to 0)	-0.16 (-0.29 to 0.08)	8.26 (6.29 to 10.5)	5.76 (4.45 to 7.02)	-1.37 (-1.45 to -1.29)
Grenada	Sickle cell disorders	0 (0 to 0)	0 (0 to 0)	-0.15 (-0.24 to -0.04)	6.66 (4.79 to 8.89)	4.71 (3.37 to 6.36)	-1.74 (-2 to -1.47)
Grenada	Sickle cell trait	0 (0 to 0)	0 (0 to 0)	-0.22 (-0.28 to -0.16)	196.58 (165.46 to 234.23)	127.25 (105.15 to 150.6)	-1.83 (-1.98 to -1.68)
Grenada	G6PD deficiency	0 (0 to 0)	0 (0 to 0)	-0.29 (-0.34 to -0.23)	183.37 (141.48 to 227.88)	108.58 (83.83 to 136.26)	-1.88 (-1.92 to -1.85)

Regions	Causes	Cases in 1990 No.×103 (95% UI)	Cases in 2019 No.×103 (95% UI)	Change in absolute number (%)	Rates in 1990 per 100000 (95% UI)	Rates in 2019 per 100000 (95% UI)	EAPC from 1990 and 2019 (95%CI)
Grenada	G6PD trait	0 (0 to 0)	0 (0 to 0)	-0.28 (-0.31 to -0.26)	216.82 (193.17 to 241.3)	129.05 (114.81 to 143.75)	-1.88 (-1.93 to -1.84)
Guam	**Inherited anemias**	1 (1 to 1)	1 (1 to 1)	-0.06 (-0.09 to -0.02)	592.05 (526.05 to 659.26)	446.99 (396.23 to 499.37)	-1.08 (-1.48 to -0.68)
Guam	Thalassemias	0 (0 to 0)	0 (0 to 0)	-0.23 (-0.33 to -0.12)	2.62 (1.39 to 4.14)	1.62 (0.86 to 2.53)	-0.64 (-1.82 to 0.56)
Guam	Thalassemias trait	0 (0 to 0)	0 (0 to 0)	-0.17 (-0.22 to -0.12)	87.14 (66.11 to 108.06)	57.9 (44.17 to 71.39)	-1.08 (-1.68 to -0.48)
Guam	Sickle cell disorders	0 (0 to 0)	0 (0 to 0)	-0.11 (-0.15 to 0.42)	0 (0 to 0)	0 (0 to 0)	-1.33 (-1.65 to -1.01)
Guam	Sickle cell trait	0 (0 to 0)	0 (0 to 0)	-0.13 (-0.15 to 0.18)	3.48 (1.53 to 5.59)	2.44 (1.04 to 3.86)	-1.38 (-1.68 to -1.08)
Guam	G6PD deficiency	0 (0 to 0)	0 (0 to 0)	0.03 (-0.03 to 0.08)	122.51 (94.51 to 151.54)	101.01 (78.06 to 126.39)	-0.86 (-1.28 to -0.43)
Guam	G6PD trait	1 (0 to 1)	0 (0 to 1)	-0.06 (-0.09 to -0.02)	376.3 (338.37 to 410.43)	284.03 (256.94 to 309.01)	-1.17 (-1.53 to -0.8)
Guatemala	**Inherited anemias**	60 (53 to 68)	64 (56 to 71)	0.05 (0.02 to 0.08)	759.38 (666.87 to 849.16)	358.75 (315.72 to 399.73)	-2.69 (-2.78 to -2.6)
Guatemala	Thalassemias	0 (0 to 0)	0 (0 to 0)	1.09 (0.2 to 3.36)	0.02 (0.01 to 0.04)	0.02 (0.01 to 0.03)	-0.41 (-0.65 to -0.17)
Guatemala	Thalassemias trait	1 (1 to 1)	1 (1 to 2)	0.52 (0.2 to 1.09)	11.75 (8.43 to 15.76)	8 (5.89 to 10.08)	-1.47 (-1.56 to -1.39)
Guatemala	Sickle cell disorders	0 (0 to 0)	0 (0 to 0)	0.23 (0.13 to 0.41)	0.1 (0.05 to 0.16)	0.05 (0.03 to 0.09)	-2.21 (-2.32 to -2.09)
Guatemala	Sickle cell trait	0 (0 to 1)	0 (0 to 1)	0.15 (-0.32 to 0.2)	4.91 (1.95 to 8.25)	2.54 (1.03 to 4.44)	-2.34 (-2.44 to -2.23)
Guatemala	G6PD deficiency	12 (9 to 15)	11 (8 to 13)	-0.1 (-0.15 to -0.06)	150.76 (115.96 to 188.57)	60.77 (46.74 to 75.33)	-3.18 (-3.4 to -2.96)
Guatemala	G6PD trait	47	51	0.08	591.83	287.37	-2.61

Regions	Causes	Cases in 1990 No.×103 (95% UI)	Cases in 2019 No.×103 (95% UI)	Change in absolute number (%)	Rates in 1990 per 100000 (95% UI)	Rates in 2019 per 100000 (95% UI)	EAPC from 1990 and 2019 (95%CI)
		(42 to 51)	(46 to 56)	(0.05 to 0.11)	(532.13 to 646.09)	(258.02 to 313.75)	(−2.68 to −2.54)
Guinea	Inherited anemias	130 (120 to 141)	203 (187 to 220)	0.56 (0.52 to 0.6)	2105.97 (1940.88 to 2279.32)	1605.14 (1480.38 to 1739.12)	−0.86 (−0.91 to −0.8)
Guinea	Thalassemias	0 (0 to 0)	0 (0 to 0)	1.04 (0.77 to 1.34)	0.41 (0.28 to 0.58)	0.41 (0.28 to 0.57)	0.06 (−0.05 to 0.16)
Guinea	Thalassemias trait	6 (5 to 7)	11 (9 to 13)	0.73 (0.63 to 0.83)	98.91 (82.26 to 118.45)	83.76 (70.44 to 100.29)	−0.5 (−0.57 to −0.43)
Guinea	Sickle cell disorders	6 (5 to 7)	9 (7 to 11)	0.54 (0.42 to 0.68)	92.46 (75.86 to 111.36)	69.76 (56.59 to 84.33)	−0.89 (−0.94 to −0.84)
Guinea	Sickle cell trait	67 (61 to 73)	103 (93 to 113)	0.54 (0.48 to 0.61)	1083.04 (980.81 to 1185.09)	817.21 (739.51 to 894.26)	−0.89 (−0.94 to −0.84)
Guinea	G6PD deficiency	16 (12 to 20)	25 (19 to 31)	0.56 (0.45 to 0.68)	259.93 (200.88 to 328.36)	198.08 (152.89 to 247.01)	−0.76 (−0.84 to −0.68)
Guinea	G6PD trait	35 (32 to 39)	55 (50 to 60)	0.56 (0.5 to 0.62)	571.23 (511.31 to 631.78)	435.91 (392.17 to 476.8)	−0.9 (−0.96 to −0.83)
Guinea-Bissau	Inherited anemias	14 (12 to 15)	19 (17 to 21)	0.35 (0.32 to 0.38)	1369.25 (1222.09 to 1527.38)	979.13 (873.1 to 1088.89)	−1.09 (−1.2 to −0.97)
Guinea-Bissau	Thalassemias	0 (0 to 0)	0 (0 to 0)	0.76 (0.5 to 1.06)	0.39 (0.26 to 0.57)	0.36 (0.25 to 0.5)	−0.2 (−0.29 to −0.1)
Guinea-Bissau	Thalassemias trait	1 (1 to 1)	1 (1 to 2)	0.51 (0.41 to 0.61)	92.58 (76.76 to 113.41)	73.84 (61.87 to 88.25)	−0.73 (−0.82 to −0.63)
Guinea-Bissau	Sickle cell disorders	0 (0 to 0)	0 (0 to 0)	0.34 (0.25 to 0.44)	2.82 (2.22 to 3.52)	2 (1.56 to 2.5)	−1.26 (−1.46 to −1.05)
Guinea-Bissau	Sickle cell trait	2 (2 to 2)	3 (2 to 3)	0.34 (0.29 to 0.4)	196.56 (171.01 to 224.04)	139.97 (122.08 to 160.13)	−1.19 (−1.35 to −1.02)
Guinea-Bissau	G6PD deficiency	4 (3 to 4)	5 (4 to 6)	0.3 (0.24 to 0.37)	348.96 (270.16 to 441.21)	240.4 (184.97 to 301.1)	−1.16 (−1.29 to −1.03)
Guinea-Bissau	G6PD trait	7 (7 to 8)	10 (9 to 11)	0.35 (0.32 to	727.95 (662.21 to	522.57 (473.78 to	−1.08 (−1.17 to

Regions	Causes	Cases in 1990 No.×103 (95% UI)	Cases in 2019 No.×103 (95% UI)	Change in absolute number (%)	Rates in 1990 per 100000 (95% UI)	Rates in 2019 per 100000 (95% UI)	EAPC from 1990 and 2019 (95%CI)
				0.4)	790.34)	568.2)	-0.98)
Guyana	Inherited anemias	6 (6 to 7)	4 (4 to 5)	-0.35 (-0.38 to -0.33)	817.73 (724.2 to 925.47)	527.65 (463.65 to 596.34)	-1.61 (-1.85 to -1.37)
Guyana	Thalassemias	0 (0 to 0)	0 (0 to 0)	0.05 (-0.37 to 1.01)	0.02 (0.01 to 0.03)	0.02 (0.01 to 0.02)	-0.03 (-0.16 to 0.09)
Guyana	Thalassemias trait	0 (0 to 0)	0 (0 to 0)	-0.25 (-0.37 to -0.07)	11.22 (8.61 to 14.19)	8.4 (6.46 to 10.07)	-1.14 (-1.27 to -1)
Guyana	Sickle cell disorders	0 (0 to 0)	0 (0 to 0)	-0.29 (-0.37 to -0.2)	9.2 (6.67 to 12.23)	6.54 (4.7 to 8.79)	-1.3 (-1.7 to -0.9)
Guyana	Sickle cell trait	2 (2 to 2)	1 (1 to 2)	-0.33 (-0.38 to -0.29)	267.5 (224.62 to 315.82)	178.29 (147.28 to 211.67)	-1.49 (-1.8 to -1.19)
Guyana	G6PD deficiency	2 (1 to 2)	1 (1 to 1)	-0.37 (-0.43 to -0.32)	246.5 (189.74 to 305.61)	154.26 (118.94 to 192.13)	-1.72 (-1.92 to -1.51)
Guyana	G6PD trait	2 (2 to 2)	1 (1 to 2)	-0.36 (-0.39 to -0.33)	283.3 (252.19 to 313.19)	180.13 (160.34 to 200.68)	-1.67 (-1.89 to -1.46)
Haiti	Inherited anemias	86 (75 to 97)	117 (103 to 132)	0.36 (0.3 to 0.41)	1347.88 (1181.23 to 1528.27)	941.32 (827.08 to 1066.97)	-1.17 (-1.21 to -1.12)
Haiti	Thalassemias	0 (0 to 0)	0 (0 to 0)	0.73 (0.32 to 1.37)	0.06 (0.04 to 0.08)	0.05 (0.04 to 0.07)	-0.34 (-0.48 to -0.2)
Haiti	Thalassemias trait	2 (2 to 2)	3 (3 to 4)	0.48 (0.32 to 0.67)	32.25 (27.1 to 37.95)	24.47 (20.78 to 28.62)	-0.85 (-0.92 to -0.78)
Haiti	Sickle cell disorders	1 (1 to 2)	2 (2 to 3)	0.41 (0.3 to 0.53)	22.61 (18.82 to 27.59)	16.37 (13.64 to 20.34)	-0.99 (-1.07 to -0.91)
Haiti	Sickle cell trait	30 (27 to 34)	42 (38 to 47)	0.38 (0.31 to 0.45)	475.88 (428.96 to 534.47)	336.91 (303.06 to 378.73)	-1.06 (-1.12 to -1)
Haiti	G6PD deficiency	36 (28 to 46)	49 (38 to 60)	0.34 (0.2 to 0.46)	569.05 (440.34 to 716.84)	392.03 (302.68 to 483.51)	-1.25 (-1.29 to -1.21)
Haiti	G6PD trait	16 (14 to 18)	21 (19 to 24)	0.35 (0.29 to 0.42)	248.03 (218.32 to 279.57)	171.49 (150.57 to 193.18)	-1.24 (-1.28 to -1.2)

Regions	Causes	Cases in 1990 No.×103 (95% UI)	Cases in 2019 No.×103 (95% UI)	Change in absolute number (%)	Rates in 1990 per 100000 (95% UI)	Rates in 2019 per 100000 (95% UI)	EAPC from 1990 and 2019 (95%CI)
Honduras	Inherited anemias	32 (28 to 36)	36 (32 to 40)	0.13 (0.09 to 0.17)	678.05 (596.13 to 757.26)	368.24 (325.01 to 411.58)	-2.23 (-2.29 to -2.16)
Honduras	Thalassemias	0 (0 to 0)	0 (0 to 0)	1.25 (0.29 to 3.74)	0.02 (0.01 to 0.03)	0.02 (0.01 to 0.03)	0.07 (-0.14 to 0.28)
Honduras	Thalassemias trait	0 (0 to 1)	1 (1 to 1)	0.63 (0.29 to 1.23)	10.42 (7.54 to 14.05)	8.12 (6.05 to 10.3)	-1.01 (-1.07 to -0.94)
Honduras	Sickle cell disorders	0 (0 to 0)	0 (0 to 0)	0.31 (0.15 to 0.94)	0.02 (0.01 to 0.04)	0.01 (0.01 to 0.02)	-1.71 (-1.85 to -1.58)
Honduras	Sickle cell trait	0 (0 to 0)	0 (0 to 0)	0.21 (-0.28 to 0.29)	3.91 (1.48 to 6.22)	2.26 (0.92 to 3.63)	-1.87 (-2.02 to -1.73)
Honduras	G6PD deficiency	6 (5 to 8)	6 (5 to 8)	-0.03 (-0.07 to 0.02)	136.05 (104.87 to 168.52)	63.27 (48.75 to 78.8)	-2.71 (-2.9 to -2.53)
Honduras	G6PD trait	25 (22 to 27)	29 (26 to 32)	0.16 (0.12 to 0.2)	527.63 (474.52 to 575.26)	294.55 (265.11 to 322.72)	-2.14 (-2.2 to -2.08)
Hungary	Inherited anemias	18 (16 to 20)	12 (11 to 14)	-0.32 (-0.34 to -0.3)	170.38 (150.01 to 191.74)	124.56 (109.69 to 140.57)	-0.95 (-1.13 to -0.76)
Hungary	Thalassemias	0 (0 to 0)	0 (0 to 0)	-0.59 (-0.64 to -0.52)	0.92 (0.59 to 1.46)	0.4 (0.27 to 0.64)	-2.42 (-2.62 to -2.22)
Hungary	Thalassemias trait	2 (2 to 3)	2 (1 to 2)	-0.3 (-0.35 to -0.25)	21.94 (18.16 to 27.02)	16.41 (13.73 to 19.81)	-0.69 (-0.9 to -0.48)
Hungary	Sickle cell disorders	0 (0 to 0)	0 (0 to 0)	-0.34 (-0.4 to -0.29)	0.09 (0.07 to 0.12)	0.06 (0.05 to 0.08)	-1.12 (-1.29 to -0.94)
Hungary	Sickle cell trait	2 (2 to 2)	1 (1 to 2)	-0.34 (-0.36 to -0.31)	19.58 (16.95 to 22.58)	13.94 (12 to 16.14)	-0.93 (-1.1 to -0.76)
Hungary	G6PD deficiency	4 (3 to 5)	3 (2 to 4)	-0.28 (-0.35 to -0.21)	41.48 (32.09 to 52.58)	32.21 (24.89 to 40.32)	-1.49 (-1.83 to -1.15)
Hungary	G6PD trait	9 (8 to 10)	6 (5 to 7)	-0.34 (-0.34 to -0.34)	86.36 (76.03 to 96.92)	61.54 (54.17 to 69.06)	-0.78 (-0.98 to -0.57)
Iceland	Inherited	1	1	-0.08	318.69	215.07	-1.14

Regions	Causes	Cases in 1990 No.×103 (95% UI)	Cases in 2019 No.×103 (95% UI)	Change in absolute number (%)	Rates in 1990 per 100000 (95% UI)	Rates in 2019 per 100000 (95% UI)	EAPC from 1990 and 2019 (95%CI)
	anemias	(1 to 1)	(1 to 1)	(-0.12 to -0.05)	(286.18 to 353.99)	(192.18 to 239.49)	(-1.3 to -0.98)
Iceland	Thalassemias	0 (0 to 0)	0 (0 to 0)	-0.15 (-0.25 to -0.03)	0.22 (0.15 to 0.3)	0.14 (0.1 to 0.19)	-2.2 (-2.87 to -1.52)
Iceland	Thalassemias trait	0 (0 to 0)	0 (0 to 0)	-0.07 (-0.12 to -0.01)	28.86 (24.8 to 32.99)	19.73 (17.15 to 22.56)	-1.29 (-1.44 to -1.15)
Iceland	Sickle cell disorders	0 (0 to 0)	0 (0 to 0)	-0.12 (-0.22 to -0.02)	0.19 (0.13 to 0.27)	0.12 (0.08 to 0.17)	-1.29 (-1.47 to -1.1)
Iceland	Sickle cell trait	0 (0 to 0)	0 (0 to 0)	-0.1 (-0.15 to -0.05)	35.53 (28.99 to 42.8)	23.58 (19.43 to 28.11)	-1.19 (-1.36 to -1.01)
Iceland	G6PD deficiency	0 (0 to 0)	0 (0 to 0)	-0.04 (-0.08 to 0.02)	48.1 (36.88 to 59.7)	33.95 (26.05 to 42.56)	-1 (-1.17 to -0.83)
Iceland	G6PD trait	1 (0 to 1)	0 (0 to 1)	-0.09 (-0.13 to -0.05)	205.8 (184.26 to 227.8)	137.55 (122.51 to 152.07)	-1.14 (-1.31 to -0.96)
India	Inherited anemias	9437 (8826 to 10149)	10728 (10107 to 11424)	0.14 (0.12 to 0.15)	1102.97 (1031.63 to 1186.22)	771.38 (726.72 to 821.43)	-1.64 (-1.97 to -1.31)
India	Thalassemias	14 (10 to 20)	22 (15 to 32)	0.55 (0.39 to 0.7)	1.68 (1.2 to 2.34)	1.6 (1.1 to 2.3)	0.63 (0.07 to 1.19)
India	Thalassemias trait	1307 (1101 to 1549)	1516 (1275 to 1805)	0.16 (0.12 to 0.2)	152.76 (128.68 to 181.04)	109.03 (91.68 to 129.79)	-0.79 (-1.08 to -0.51)
India	Sickle cell disorders	112 (75 to 163)	124 (83 to 179)	0.11 (0.07 to 0.16)	13.06 (8.79 to 19.01)	8.94 (5.98 to 12.88)	-1.42 (-1.76 to -1.09)
India	Sickle cell trait	3161 (2639 to 3778)	3131 (2613 to 3717)	-0.01 (-0.03 to 0.01)	369.51 (308.39 to 441.51)	225.15 (187.87 to 267.3)	-1.76 (-1.95 to -1.58)
India	G6PD deficiency	1240 (1190 to 1286)	1956 (1873 to 2034)	0.58 (0.53 to 0.61)	144.94 (139.13 to 150.3)	140.66 (134.65 to 146.27)	-1.75 (-2.56 to -0.93)
India	G6PD trait	3602 (3543 to 3654)	3978 (3914 to 4034)	0.1 (0.09 to 0.11)	421.01 (414.16 to 427.11)	286.01 (281.41 to 290.09)	-1.76 (-2.12 to -1.41)
Indonesia	Inherited anemias	1311 (1261 to	878 (838 to	-0.33 (-0.34 to	707.23 (680 to	338.38 (323.08 to	-2.52 (-2.72 to

Regions	Causes	Cases in 1990 No.×103 (95% UI)	Cases in 2019 No.×103 (95% UI)	Change in absolute number (%)	Rates in 1990 per 100000 (95% UI)	Rates in 2019 per 100000 (95% UI)	EAPC from 1990 and 2019 (95%CI)
		1367)	923)	-0.32)	737.38)	355.6)	-2.32)
Indonesia	Thalassemias	9 (7 to 12)	6 (5 to 8)	-0.34 (-0.38 to -0.29)	4.94 (3.73 to 6.64)	2.33 (1.76 to 3.11)	-2.46 (-2.61 to -2.32)
Indonesia	Thalassemias trait	297 (254 to 346)	240 (205 to 279)	-0.19 (-0.21 to -0.18)	160.48 (137.03 to 186.44)	92.31 (78.98 to 107.59)	-1.78 (-1.93 to -1.63)
Indonesia	Sickle cell disorders	0 (0 to 0)	0 (0 to 0)	-0.18 (-0.22 to -0.12)	0.02 (0.02 to 0.03)	0.01 (0.01 to 0.02)	-1.75 (-1.89 to -1.61)
Indonesia	Sickle cell trait	26 (22 to 31)	21 (18 to 25)	-0.19 (-0.21 to -0.15)	14.23 (11.95 to 16.84)	8.24 (6.91 to 9.77)	-1.76 (-1.91 to -1.62)
Indonesia	G6PD deficiency	329 (316 to 341)	161 (153 to 168)	-0.51 (-0.53 to -0.5)	177.55 (170.56 to 184.21)	62.01 (59.14 to 64.75)	-3.72 (-4.3 to -3.13)
Indonesia	G6PD trait	649 (637 to 659)	450 (441 to 458)	-0.31 (-0.31 to -0.3)	350 (343.62 to 355.64)	173.47 (170.06 to 176.6)	-2.45 (-2.71 to -2.2)
Iran (Islamic Republic of)	**Inherited anemias**	582 (553 to 615)	498 (477 to 523)	-0.14 (-0.16 to -0.13)	993.37 (944.92 to 1049.98)	590.88 (566.09 to 620.96)	-1.38 (-1.55 to -1.2)
Iran (Islamic Republic of)	Thalassemias	2 (1 to 3)	2 (1 to 2)	-0.19 (-0.22 to -0.16)	3.23 (2.14 to 4.86)	1.81 (1.22 to 2.69)	-1.46 (-1.67 to -1.24)
Iran (Islamic Republic of)	Thalassemias trait	117 (98 to 143)	95 (79 to 114)	-0.19 (-0.21 to -0.18)	200.55 (166.63 to 243.59)	112.35 (94.05 to 135.31)	-1.45 (-1.66 to -1.23)
Iran (Islamic Republic of)	Sickle cell disorders	4 (3 to 5)	2 (2 to 3)	-0.49 (-0.5 to -0.46)	6.59 (5.07 to 8.59)	2.35 (1.82 to 3.06)	-3.78 (-4.15 to -3.4)
Iran (Islamic Republic of)	Sickle cell trait	153 (133 to 174)	96 (84 to 110)	-0.37 (-0.38 to -0.36)	260.91 (228.04 to 297.49)	113.86 (99.39 to 130.12)	-2.69 (-2.84 to -2.55)
Iran (Islamic Republic of)	G6PD deficiency	100 (96 to 104)	89 (85 to 93)	-0.12 (-0.15 to -0.09)	171.56 (164.76 to 177.97)	105.21 (100.25 to 110.03)	-1 (-1.42 to -0.58)

Regions	Causes	Cases in 1990 No.×103 (95% UI)	Cases in 2019 No.×103 (95% UI)	Change in absolute number (%)	Rates in 1990 per 100000 (95% UI)	Rates in 2019 per 100000 (95% UI)	EAPC from 1990 and 2019 (95%CI)
Iran (Islamic Republic of)	G6PD trait	205 (201 to 209)	215 (211 to 219)	0.05 (0.03 to 0.06)	350.53 (344.18 to 356.19)	255.31 (250.84 to 259.38)	−0.76 (−0.99 to −0.52)
Iraq	Inherited anemias	265 (242 to 289)	351 (322 to 383)	0.33 (0.3 to 0.35)	1506.19 (1376.53 to 1643.71)	834.32 (763.61 to 908.79)	−2.2 (−2.28 to −2.13)
Iraq	Thalassemias	0 (0 to 1)	1 (0 to 1)	0.39 (0.26 to 0.53)	2.38 (1.85 to 3.06)	1.38 (1.08 to 1.77)	−2.19 (−2.28 to −2.09)
Iraq	Thalassemias trait	37 (33 to 42)	51 (45 to 57)	0.37 (0.31 to 0.44)	210.54 (187.38 to 236.34)	120.75 (107.32 to 136.13)	−2.2 (−2.29 to −2.11)
Iraq	Sickle cell disorders	2 (1 to 2)	2 (2 to 2)	0.29 (0.21 to 0.38)	8.89 (7.37 to 10.86)	4.81 (3.95 to 5.86)	−2.12 (−2.36 to −1.88)
Iraq	Sickle cell trait	64 (59 to 71)	84 (76 to 92)	0.3 (0.25 to 0.34)	365.44 (334.34 to 401.87)	198.4 (180.77 to 218.41)	−2.23 (−2.37 to −2.1)
Iraq	G6PD deficiency	46 (35 to 57)	61 (47 to 75)	0.32 (0.25 to 0.4)	260.08 (200.54 to 321.87)	143.64 (111.17 to 178.34)	−1.98 (−2.11 to −1.86)
Iraq	G6PD trait	116 (105 to 125)	154 (140 to 167)	0.33 (0.3 to 0.38)	658.87 (598.31 to 711.92)	365.33 (331.54 to 396.56)	−2.28 (−2.36 to −2.19)
Ireland	Inherited anemias	9 (8 to 10)	11 (10 to 12)	0.14 (0.11 to 0.18)	261.07 (234.01 to 289.78)	218.64 (195.8 to 242.68)	−0.2 (−0.5 to 0.1)
Ireland	Thalassemias	0 (0 to 0)	0 (0 to 0)	0.09 (−0.06 to 0.26)	0.18 (0.13 to 0.25)	0.15 (0.1 to 0.2)	−1.16 (−2.02 to −0.29)
Ireland	Thalassemias trait	1 (1 to 1)	1 (1 to 1)	0.17 (0.1 to 0.25)	23.3 (20.34 to 26.63)	20.07 (17.21 to 23.02)	−0.24 (−0.62 to 0.15)
Ireland	Sickle cell disorders	0 (0 to 0)	0 (0 to 0)	−0.07 (−0.18 to 0.04)	0.25 (0.18 to 0.35)	0.17 (0.13 to 0.23)	−1.67 (−2.09 to −1.25)
Ireland	Sickle cell trait	1 (1 to 1)	1 (1 to 1)	0 (−0.07 to 0.07)	32.75 (26.92 to 39.96)	24.11 (19.78 to 29.05)	−1.13 (−1.42 to −0.85)
Ireland	G6PD deficiency	1 (1 to 2)	2 (1 to 2)	0.21 (0.17 to 0.26)	39.66 (30.34 to 49.36)	35.22 (27.05 to 43.95)	0.05 (−0.23 to 0.34)

Regions	Causes	Cases in 1990 No.×103 (95% UI)	Cases in 2019 No.×103 (95% UI)	Change in absolute number (%)	Rates in 1990 per 100000 (95% UI)	Rates in 2019 per 100000 (95% UI)	EAPC from 1990 and 2019 (95%CI)
Ireland	G6PD trait	6 (5 to 7)	7 (6 to 8)	0.15 (0.1 to 0.2)	164.92 (147.61 to 180.99)	138.92 (124.26 to 153.91)	-0.08 (-0.41 to 0.26)
Israel	**Inherited anemias**	22 (20 to 24)	43 (38 to 49)	0.96 (0.89 to 1.03)	443.23 (395.23 to 492.85)	463.55 (410.22 to 521.67)	0.19 (0.1 to 0.28)
Israel	Thalassemias	0 (0 to 0)	0 (0 to 0)	0.74 (0.46 to 1.01)	0.24 (0.16 to 0.34)	0.22 (0.15 to 0.32)	-1.29 (-2.22 to -0.34)
Israel	Thalassemias trait	1 (1 to 1)	2 (2 to 3)	0.92 (0.79 to 1.05)	25.05 (21.66 to 28.67)	25.68 (22.01 to 29.75)	-0.25 (-0.54 to 0.05)
Israel	Sickle cell disorders	0 (0 to 0)	0 (0 to 0)	0.35 (0.1 to 0.63)	1.25 (0.95 to 1.6)	0.9 (0.64 to 1.27)	-1.1 (-1.4 to -0.8)
Israel	Sickle cell trait	5 (4 to 5)	7 (6 to 9)	0.55 (0.38 to 0.73)	93.25 (80.22 to 106.51)	77.16 (62.36 to 93.15)	-0.63 (-0.81 to -0.45)
Israel	G6PD deficiency	5 (4 to 6)	11 (9 to 14)	1.19 (1.05 to 1.37)	101.27 (78.3 to 125.24)	118.37 (92.02 to 148.2)	0.43 (0.26 to 0.61)
Israel	G6PD trait	11 (10 to 12)	22 (20 to 25)	1.04 (0.97 to 1.12)	222.17 (199.27 to 243.68)	241.21 (216.59 to 265.51)	0.43 (0.35 to 0.52)
Italy	**Inherited anemias**	229 (216 to 243)	141 (135 to 148)	-0.38 (-0.41 to -0.36)	403.05 (379.9 to 428.35)	233.67 (223.14 to 245.54)	-1.82 (-2.13 to -1.5)
Italy	Thalassemias	1 (1 to 2)	1 (1 to 1)	-0.24 (-0.28 to -0.19)	2.59 (1.88 to 3.45)	1.85 (1.38 to 2.4)	-0.81 (-1.05 to -0.57)
Italy	Thalassemias trait	53 (46 to 61)	41 (35 to 47)	-0.23 (-0.24 to -0.21)	92.97 (80.17 to 107.14)	67.51 (58.76 to 77.51)	-0.88 (-1.09 to -0.67)
Italy	Sickle cell disorders	7 (4 to 10)	0 (0 to 0)	-0.98 (-0.98 to -0.98)	11.73 (7.9 to 16.78)	0.21 (0.16 to 0.28)	-13.58 (-15.82 to -11.29)
Italy	Sickle cell trait	55 (47 to 63)	11 (10 to 13)	-0.79 (-0.81 to -0.77)	96.1 (82.34 to 111.64)	18.64 (16.57 to 21.09)	-5.38 (-6.14 to -4.61)
Italy	G6PD deficiency	27 (26 to 28)	23 (22 to 24)	-0.13 (-0.16 to -0.11)	47.16 (45.36 to 48.95)	38.6 (36.78 to 40.24)	-1.75 (-2.78 to -0.71)
Italy	G6PD trait	87	64	-0.26	152.5	106.87	-1.25

Regions	Causes	Cases in 1990 No.×103 (95% UI)	Cases in 2019 No.×103 (95% UI)	Change in absolute number (%)	Rates in 1990 per 100000 (95% UI)	Rates in 2019 per 100000 (95% UI)	EAPC from 1990 and 2019 (95%CI)
		(85 to 88)	(63 to 66)	(-0.26 to -0.25)	(149.99 to 154.96)	(104.77 to 108.85)	(-1.51 to -0.98)
Jamaica	**Inherited anemias**	19 (17 to 21)	12 (11 to 14)	-0.36 (-0.38 to -0.33)	816.28 (735.19 to 897.58)	439.65 (393.34 to 490.25)	-2.2 (-2.32 to -2.07)
Jamaica	Thalassemias	0 (0 to 0)	0 (0 to 0)	0 (-0.37 to 0.85)	0.01 (0.01 to 0.02)	0.01 (0.01 to 0.02)	-0.74 (-0.83 to -0.65)
Jamaica	Thalassemias trait	0 (0 to 0)	0 (0 to 0)	-0.26 (-0.37 to -0.09)	10.01 (8.1 to 12.11)	6.25 (5.07 to 7.45)	-1.69 (-1.78 to -1.61)
Jamaica	Sickle cell disorders	0 (0 to 0)	0 (0 to 0)	-0.39 (-0.47 to -0.29)	13.61 (12.46 to 14.87)	7.02 (5.72 to 8.51)	-2.25 (-2.38 to -2.13)
Jamaica	Sickle cell trait	6 (6 to 7)	4 (3 to 4)	-0.38 (-0.43 to -0.32)	262.47 (248.45 to 276.82)	136.78 (121.85 to 153.36)	-2.3 (-2.41 to -2.2)
Jamaica	G6PD deficiency	5 (4 to 6)	3 (3 to 4)	-0.35 (-0.4 to -0.28)	210.91 (162.27 to 260.39)	115.65 (89.02 to 143.23)	-2.16 (-2.29 to -2.02)
Jamaica	G6PD trait	8 (7 to 8)	5 (4 to 5)	-0.35 (-0.35 to -0.35)	319.26 (287.2 to 349.09)	173.94 (156.72 to 190.13)	-2.15 (-2.29 to -2.01)
Japan	**Inherited anemias**	85 (79 to 91)	64 (60 to 69)	-0.24 (-0.29 to -0.2)	67.29 (62.68 to 72.25)	50.28 (46.7 to 54.08)	-0.9 (-1.2 to -0.61)
Japan	Thalassemias	1 (1 to 1)	1 (1 to 1)	0.2 (0.05 to 0.39)	0.6 (0.44 to 0.86)	0.71 (0.51 to 1)	0.64 (-0.08 to 1.36)
Japan	Thalassemias trait	24 (18 to 30)	22 (18 to 26)	-0.07 (-0.25 to 0.08)	18.82 (14.38 to 23.58)	17.26 (13.81 to 20.66)	0.01 (-0.57 to 0.6)
Japan	Sickle cell disorders	0 (0 to 0)	0 (0 to 0)	-0.2 (-0.52 to 0.14)	0 (0 to 0.01)	0 (0 to 0)	-0.62 (-0.89 to -0.34)
Japan	Sickle cell trait	3 (2 to 4)	2 (2 to 3)	-0.19 (-0.25 to -0.08)	2.26 (1.32 to 3.2)	1.79 (1.18 to 2.43)	-0.61 (-0.74 to -0.49)
Japan	G6PD deficiency	4 (3 to 4)	3 (3 to 3)	-0.08 (-0.1 to -0.06)	2.88 (2.76 to 3.02)	2.61 (2.48 to 2.74)	-0.77 (-1.04 to -0.5)
Japan	G6PD trait	54 (52 to 55)	36 (35 to 37)	-0.34 (-0.35 to	42.73 (41.64 to	27.92 (27.1 to	-1.4 (-1.59 to

Regions	Causes	Cases in 1990 No.×103 (95% UI)	Cases in 2019 No.×103 (95% UI)	Change in absolute number (%)	Rates in 1990 per 100000 (95% UI)	Rates in 2019 per 100000 (95% UI)	EAPC from 1990 and 2019 (95%CI)
				-0.32)	43.88)	28.74)	-1.21)
Jordan	Inherited anemias	49 (44 to 53)	83 (76 to 90)	0.7 (0.66 to 0.74)	1286.87 (1177.41 to 1399.85)	709.25 (649.32 to 772.35)	-1.97 (-2.05 to -1.9)
Jordan	Thalassemias	0 (0 to 0)	0 (0 to 0)	0.8 (0.62 to 1)	2.23 (1.66 to 2.93)	1.3 (0.97 to 1.7)	-1.82 (-1.89 to -1.75)
Jordan	Thalassemias trait	7 (6 to 8)	13 (11 to 15)	0.81 (0.73 to 0.9)	189.38 (165.4 to 215.87)	111.17 (97.39 to 126.59)	-1.77 (-1.84 to -1.7)
Jordan	Sickle cell disorders	0 (0 to 0)	0 (0 to 0)	0.68 (0.55 to 0.81)	4 (3.07 to 5.03)	2.18 (1.68 to 2.75)	-2.67 (-2.94 to -2.39)
Jordan	Sickle cell trait	9 (8 to 10)	15 (13 to 17)	0.71 (0.64 to 0.77)	229.66 (201.6 to 258.21)	127.08 (112.29 to 143.05)	-2.27 (-2.41 to -2.12)
Jordan	G6PD deficiency	8 (6 to 10)	13 (10 to 16)	0.61 (0.51 to 0.71)	207.71 (162.01 to 257.59)	108.63 (84.38 to 136.44)	-2.05 (-2.16 to -1.94)
Jordan	G6PD trait	25 (23 to 27)	42 (38 to 45)	0.69 (0.64 to 0.74)	653.9 (598.18 to 704.27)	358.9 (328.01 to 387.13)	-1.9 (-1.99 to -1.81)
Kazakhstan	Inherited anemias	51 (45 to 56)	51 (45 to 56)	-0.01 (-0.03 to 0.01)	311.51 (277.64 to 345.02)	275.08 (246.24 to 305.97)	0.74 (0.27 to 1.2)
Kazakhstan	Thalassemias	0 (0 to 1)	0 (0 to 0)	-0.21 (-0.3 to -0.1)	2.31 (1.65 to 3.18)	1.62 (1.17 to 2.27)	-0.06 (-0.53 to 0.41)
Kazakhstan	Thalassemias trait	17 (14 to 21)	17 (14 to 21)	0 (-0.06 to 0.07)	103.84 (86.12 to 126)	92.46 (76.8 to 113.74)	0.74 (0.28 to 1.21)
Kazakhstan	Sickle cell disorders	0 (0 to 0)	0 (0 to 0)	-0.01 (-0.21 to 0.11)	0.01 (0.01 to 0.02)	0.01 (0.01 to 0.02)	0.75 (0.28 to 1.22)
Kazakhstan	Sickle cell trait	2 (1 to 2)	2 (1 to 2)	-0.01 (-0.12 to 0.06)	9.8 (7.74 to 12.25)	8.63 (6.76 to 10.75)	0.74 (0.28 to 1.21)
Kazakhstan	G6PD deficiency	5 (4 to 6)	5 (4 to 6)	0.01 (-0.03 to 0.08)	29.09 (22.6 to 36.35)	26.19 (20.18 to 33.08)	0.87 (0.39 to 1.35)
Kazakhstan	G6PD trait	27 (24 to 30)	27 (24 to 30)	-0.01 (-0.01 to -0.01)	166.46 (148.14 to 185.56)	146.16 (130.08 to 162.93)	0.72 (0.26 to 1.19)

Regions	Causes	Cases in 1990 No.×103 (95% UI)	Cases in 2019 No.×103 (95% UI)	Change in absolute number (%)	Rates in 1990 per 100000 (95% UI)	Rates in 2019 per 100000 (95% UI)	EAPC from 1990 and 2019 (95%CI)
Kenya	Inherited anemias	574 (533 to 618)	692 (643 to 746)	0.21 (0.2 to 0.22)	2475.46 (2298.91 to 2665.59)	1378.44 (1279.25 to 1485.61)	-1.99 (-2.12 to -1.86)
Kenya	Thalassemias	0 (0 to 0)	0 (0 to 0)	1.93 (1.65 to 2.37)	0.09 (0.05 to 0.13)	0.12 (0.07 to 0.18)	0.75 (0.55 to 0.95)
Kenya	Thalassemias trait	6 (5 to 7)	11 (9 to 13)	0.82 (0.7 to 0.95)	25.83 (20.72 to 31.31)	21.7 (17.17 to 26.49)	-0.73 (-0.84 to -0.61)
Kenya	Sickle cell disorders	19 (14 to 27)	24 (17 to 33)	0.22 (0.18 to 0.26)	83.81 (58.35 to 116.76)	47.06 (33.47 to 64.81)	-1.7 (-1.82 to -1.59)
Kenya	Sickle cell trait	234 (201 to 269)	299 (260 to 344)	0.28 (0.26 to 0.3)	1007.62 (866.5 to 1158.24)	596.17 (516.72 to 684.21)	-1.67 (-1.77 to -1.58)
Kenya	G6PD deficiency	108 (103 to 113)	128 (122 to 133)	0.18 (0.17 to 0.21)	466.68 (445.04 to 486.36)	255.19 (243.77 to 265.43)	-2.29 (-2.42 to -2.15)
Kenya	G6PD trait	207 (204 to 209)	230 (226 to 234)	0.11 (0.11 to 0.12)	891.43 (879.12 to 902.79)	458.2 (450.57 to 465.47)	-2.31 (-2.53 to -2.09)
Kiribati	Inherited anemias	1 (1 to 1)	1 (1 to 1)	0.29 (0.25 to 0.33)	769.72 (683.21 to 859.31)	620.91 (551.89 to 692.42)	-0.51 (-0.73 to -0.28)
Kiribati	Thalassemias	0 (0 to 0)	0 (0 to 0)	0.05 (-0.08 to 0.18)	4.9 (2.69 to 7.37)	3.23 (1.78 to 4.81)	-0.07 (-1.06 to 0.93)
Kiribati	Thalassemias trait	0 (0 to 0)	0 (0 to 0)	0.14 (0.07 to 0.2)	132.35 (101.74 to 159.97)	93.84 (71.51 to 113.58)	-0.52 (-0.95 to -0.09)
Kiribati	Sickle cell disorders	0 (0 to 0)	0 (0 to 0)	0.24 (0.16 to 1.48)	0 (0 to 0.01)	0 (0 to 0)	-0.68 (-0.86 to -0.5)
Kiribati	Sickle cell trait	0 (0 to 0)	0 (0 to 0)	0.21 (0.16 to 0.82)	4.96 (2.11 to 7.48)	3.75 (1.58 to 6.36)	-0.78 (-0.93 to -0.63)
Kiribati	G6PD deficiency	0 (0 to 0)	0 (0 to 0)	0.42 (0.32 to 0.52)	157.56 (121.17 to 198.34)	139.64 (108.13 to 175.78)	-0.26 (-0.53 to 0)
Kiribati	G6PD trait	0 (0 to 0)	0 (0 to 0)	0.3 (0.25 to 0.34)	469.94 (422.35 to 517.13)	380.46 (345.3 to 412.73)	-0.6 (-0.78 to -0.42)
Kuwait	Inherited	9	14	0.55	526.25	323.96	-1.85

Regions	Causes	Cases in 1990 No.×103 (95% UI)	Cases in 2019 No.×103 (95% UI)	Change in absolute number (%)	Rates in 1990 per 100000 (95% UI)	Rates in 2019 per 100000 (95% UI)	EAPC from 1990 and 2019 (95%CI)
	**anemias**	(8 to 10)	(13 to 16)	(0.52 to 0.59)	(475.09 to 581.09)	(292.31 to 357.94)	(-2.02 to -1.69)
Kuwait	Thalassemias	0 (0 to 0)	0 (0 to 0)	0.82 (0.62 to 1.05)	0.31 (0.21 to 0.43)	0.22 (0.16 to 0.3)	-1.23 (-1.47 to -0.99)
Kuwait	Thalassemias trait	1 (1 to 1)	2 (1 to 2)	0.67 (0.58 to 0.75)	57.39 (48.56 to 68.19)	38.01 (32.47 to 44.8)	-1.49 (-1.73 to -1.25)
Kuwait	Sickle cell disorders	0 (0 to 0)	0 (0 to 0)	0.55 (0.44 to 0.66)	1.7 (1.34 to 2.13)	1.05 (0.82 to 1.32)	-1.69 (-1.93 to -1.45)
Kuwait	Sickle cell trait	2 (2 to 2)	3 (3 to 4)	0.54 (0.48 to 0.59)	119.52 (106.46 to 133.77)	73 (65.04 to 81.8)	-1.72 (-1.96 to -1.48)
Kuwait	G6PD deficiency	2 (1 to 2)	3 (2 to 3)	0.54 (0.48 to 0.63)	97.7 (75.93 to 122.06)	59.75 (46.19 to 74.51)	-1.39 (-1.65 to -1.13)
Kuwait	G6PD trait	4 (4 to 5)	7 (6 to 7)	0.53 (0.47 to 0.59)	249.63 (222.95 to 274.46)	151.93 (136 to 168.5)	-2.18 (-2.47 to -1.9)
Kyrgyzstan	**Inherited anemias**	18 (16 to 20)	21 (19 to 24)	0.19 (0.16 to 0.22)	405.46 (363.32 to 453.23)	328.12 (293.81 to 364.99)	0.14 (-0.29 to 0.57)
Kyrgyzstan	Thalassemias	0 (0 to 0)	0 (0 to 0)	0 (-0.12 to 0.14)	3.06 (2.19 to 4.28)	2.08 (1.49 to 2.88)	-0.46 (-0.9 to -0.02)
Kyrgyzstan	Thalassemias trait	6 (5 to 7)	7 (6 to 9)	0.23 (0.15 to 0.32)	136.18 (113.86 to 166.1)	114.74 (95.55 to 139.2)	0.26 (-0.17 to 0.69)
Kyrgyzstan	Sickle cell disorders	0 (0 to 0)	0 (0 to 0)	0.17 (0 to 0.43)	0.02 (0.01 to 0.02)	0.01 (0.01 to 0.02)	0.12 (-0.31 to 0.56)
Kyrgyzstan	Sickle cell trait	1 (0 to 1)	1 (1 to 1)	0.17 (0.07 to 0.3)	12.8 (10.09 to 15.93)	10.23 (8.11 to 12.7)	0.1 (-0.33 to 0.54)
Kyrgyzstan	G6PD deficiency	2 (1 to 2)	2 (2 to 3)	0.19 (0.12 to 0.28)	38.13 (29.27 to 47.4)	31.01 (24.06 to 39.03)	0.22 (-0.24 to 0.67)
Kyrgyzstan	G6PD trait	10 (9 to 11)	11 (10 to 12)	0.16 (0.16 to 0.16)	215.27 (191.46 to 238.68)	170.05 (151.24 to 188.54)	0.06 (-0.36 to 0.49)

Regions	Causes	Cases in 1990 No.×103 (95% UI)	Cases in 2019 No.×103 (95% UI)	Change in absolute number (%)	Rates in 1990 per 100000 (95% UI)	Rates in 2019 per 100000 (95% UI)	EAPC from 1990 and 2019 (95%CI)
Lao People's Democratic Republic	Inherited anemias	49 (45 to 54)	51 (47 to 57)	0.04 (0.01 to 0.07)	1191.21 (1079.46 to 1309.6)	718.27 (651.45 to 791.38)	-1.93 (-2 to -1.85)
Lao People's Democratic Republic	Thalassemias	1 (1 to 1)	1 (1 to 2)	0.63 (0.49 to 0.78)	21.38 (16.51 to 27.21)	20.21 (15.71 to 26.56)	-0.37 (-0.45 to -0.29)
Lao People's Democratic Republic	Thalassemias trait	15 (14 to 17)	19 (17 to 21)	0.26 (0.21 to 0.3)	366.35 (328.32 to 410.01)	267.09 (242.66 to 296.82)	-1.26 (-1.33 to -1.19)
Lao People's Democratic Republic	Sickle cell disorders	0 (0 to 0)	0 (0 to 0)	0.05 (-0.07 to 0.21)	0.04 (0.02 to 0.05)	0.02 (0.01 to 0.03)	-1.87 (-1.92 to -1.81)
Lao People's Democratic Republic	Sickle cell trait	1 (1 to 1)	1 (1 to 1)	0.05 (-0.01 to 0.13)	22.43 (18.45 to 27.13)	13.63 (11.22 to 16.43)	-1.87 (-1.93 to -1.8)
Lao People's Democratic Republic	G6PD deficiency	9 (7 to 11)	9 (7 to 11)	-0.01 (-0.08 to 0.06)	214.08 (164.77 to 266.4)	123.02 (95.4 to 153.59)	-2.14 (-2.22 to -2.06)
Lao People's Democratic Republic	G6PD trait	24 (21 to 26)	21 (19 to 23)	-0.1 (-0.14 to -0.08)	566.94 (510.69 to 618.45)	294.31 (265.12 to 324.67)	-2.42 (-2.57 to -2.28)
Latvia	Inherited anemias	5 (4 to 5)	2 (2 to 3)	-0.46 (-0.47 to -0.46)	170.47 (150.79 to 191.29)	126.78 (111.85 to 141.56)	0.17 (-0.4 to 0.75)
Latvia	Thalassemias	0 (0 to 0)	0 (0 to 0)	-0.64 (-0.67 to -0.59)	0.71 (0.49 to 1.04)	0.36 (0.26 to 0.51)	-1.11 (-1.7 to -0.52)
Latvia	Thalassemias trait	1 (1 to 1)	0 (0 to 1)	-0.45 (-0.49 to -0.42)	33.06 (27.45 to 40.6)	25.11 (20.95 to 29.97)	0.24 (-0.35 to 0.83)
Latvia	Sickle cell disorders	0 (0 to 0)	0 (0 to 0)	-0.44 (-0.54 to -0.32)	0.01 (0.01 to 0.01)	0.01 (0 to 0.01)	0.34 (-0.19 to 0.88)
Latvia	Sickle cell trait	0 (0 to 0)	0 (0 to 0)	-0.46 (-0.52 to -0.41)	6.44 (5.18 to 7.97)	4.79 (3.9 to 5.92)	0.23 (-0.33 to 0.79)

Regions	Causes	Cases in 1990 No.×103 (95% UI)	Cases in 2019 No.×103 (95% UI)	Change in absolute number (%)	Rates in 1990 per 100000 (95% UI)	Rates in 2019 per 100000 (95% UI)	EAPC from 1990 and 2019 (95%CI)
Latvia	G6PD deficiency	1 (1 to 1)	0 (0 to 0)	−0.43 (−0.46 to −0.41)	26.6 (20.4 to 33.03)	20.97 (16.08 to 26.01)	0.07 (−0.49 to 0.63)
Latvia	G6PD trait	3 (2 to 3)	1 (1 to 2)	−0.48 (−0.48 to −0.48)	103.66 (91.74 to 115.99)	75.54 (66.85 to 84.53)	0.18 (−0.4 to 0.77)
Lebanon	Inherited anemias	35 (32 to 38)	34 (31 to 36)	−0.03 (−0.06 to −0.01)	1063.95 (978.01 to 1155.03)	649.95 (598.4 to 703.83)	−1.32 (−1.48 to −1.16)
Lebanon	Thalassemias	0 (0 to 0)	0 (0 to 0)	−0.02 (−0.12 to 0.11)	3.1 (2.23 to 4.21)	1.92 (1.41 to 2.55)	−1.33 (−1.47 to −1.19)
Lebanon	Thalassemias trait	7 (6 to 8)	7 (6 to 8)	−0.02 (−0.07 to 0.04)	214.11 (183.89 to 247.27)	132.65 (115.77 to 152.06)	−1.31 (−1.45 to −1.16)
Lebanon	Sickle cell disorders	0 (0 to 0)	0 (0 to 0)	0.06 (−0.02 to 0.14)	2.33 (1.85 to 2.94)	1.56 (1.24 to 1.93)	−1.02 (−1.14 to −0.9)
Lebanon	Sickle cell trait	6 (5 to 6)	6 (5 to 6)	0 (−0.04 to 0.03)	173.74 (155.52 to 195.04)	109.37 (98.07 to 122.07)	−1.22 (−1.34 to −1.09)
Lebanon	G6PD deficiency	4 (3 to 5)	4 (3 to 5)	−0.04 (−0.09 to 0.01)	117.89 (91.2 to 147.47)	71.3 (55.27 to 88.36)	−1.32 (−1.8 to −0.85)
Lebanon	G6PD trait	18 (16 to 20)	17 (16 to 19)	−0.05 (−0.08 to −0.02)	552.79 (501.49 to 601.58)	333.15 (303.27 to 361.48)	−1.37 (−1.51 to −1.22)
Lesotho	Inherited anemias	18 (16 to 20)	13 (11 to 15)	−0.27 (−0.3 to −0.26)	982.6 (859.19 to 1112.3)	615.81 (539.93 to 694.52)	−1.58 (−1.68 to −1.48)
Lesotho	Thalassemias	0 (0 to 0)	0 (0 to 0)	0.56 (0.35 to 0.85)	0.08 (0.05 to 0.12)	0.11 (0.06 to 0.16)	0.82 (0.66 to 0.98)
Lesotho	Thalassemias trait	0 (0 to 1)	0 (0 to 1)	−0.04 (−0.11 to 0.04)	27.16 (22.71 to 32.3)	22.51 (18.45 to 26.77)	−0.72 (−0.79 to −0.65)
Lesotho	Sickle cell disorders	0 (0 to 0)	0 (0 to 0)	−0.25 (−0.31 to −0.18)	0.12 (0.08 to 0.17)	0.08 (0.05 to 0.11)	−1.49 (−1.55 to −1.43)
Lesotho	Sickle cell trait	1 (0 to 1)	0 (0 to 1)	−0.25 (−0.29 to −0.22)	34.18 (26.45 to 43.54)	22.12 (17.17 to 28.19)	−1.5 (−1.56 to −1.44)

Regions	Causes	Cases in 1990 No.×103 (95% UI)	Cases in 2019 No.×103 (95% UI)	Change in absolute number (%)	Rates in 1990 per 100000 (95% UI)	Rates in 2019 per 100000 (95% UI)	EAPC from 1990 and 2019 (95%CI)
Lesotho	G6PD deficiency	6 (4 to 7)	4 (3 to 5)	-0.31 (-0.35 to -0.26)	312.17 (242.35 to 393.09)	187.13 (145.36 to 233.02)	-1.74 (-1.88 to -1.6)
Lesotho	G6PD trait	11 (10 to 12)	8 (7 to 9)	-0.27 (-0.29 to -0.25)	608.89 (557.73 to 657.37)	383.88 (350 to 415.54)	-1.55 (-1.64 to -1.47)
Liberia	Inherited anemias	39 (36 to 43)	61 (56 to 66)	0.54 (0.52 to 0.57)	2006.89 (1841.24 to 2190.34)	1270.33 (1163.38 to 1385.61)	-1.63 (-1.85 to -1.4)
Liberia	Thalassemias	0 (0 to 0)	0 (0 to 0)	0.59 (0.45 to 0.76)	6.54 (4.97 to 8.68)	4.26 (3.26 to 5.71)	-1.55 (-1.78 to -1.32)
Liberia	Thalassemias trait	7 (6 to 8)	11 (10 to 12)	0.59 (0.52 to 0.66)	346.52 (306.4 to 394.64)	225.48 (200.16 to 257.55)	-1.55 (-1.78 to -1.32)
Liberia	Sickle cell disorders	0 (0 to 1)	1 (1 to 1)	0.55 (0.47 to 0.64)	22.07 (18.61 to 25.78)	14 (11.82 to 16.51)	-1.63 (-1.86 to -1.4)
Liberia	Sickle cell trait	10 (9 to 11)	16 (15 to 17)	0.54 (0.49 to 0.59)	522.1 (480.23 to 565.71)	328.74 (303.09 to 356.84)	-1.65 (-1.88 to -1.42)
Liberia	G6PD deficiency	8 (6 to 10)	12 (9 to 15)	0.53 (0.43 to 0.63)	398.24 (307 to 498.41)	250.65 (193.84 to 315.29)	-1.61 (-1.82 to -1.39)
Liberia	G6PD trait	14 (13 to 15)	21 (19 to 23)	0.53 (0.53 to 0.53)	711.44 (644.9 to 772.73)	447.21 (405.46 to 485.6)	-1.66 (-1.89 to -1.44)
Libya	Inherited anemias	74 (68 to 80)	38 (35 to 41)	-0.48 (-0.49 to -0.47)	1745.25 (1604.29 to 1887.46)	567.07 (521.76 to 614.78)	-3.85 (-3.92 to -3.77)
Libya	Thalassemias	0 (0 to 0)	0 (0 to 0)	-0.38 (-0.45 to -0.28)	2.18 (1.42 to 3.3)	0.85 (0.57 to 1.27)	-3.32 (-3.4 to -3.24)
Libya	Thalassemias trait	8 (7 to 10)	5 (4 to 6)	-0.42 (-0.45 to -0.38)	187.44 (154.22 to 228.74)	68.26 (56.88 to 82.43)	-3.52 (-3.59 to -3.46)
Libya	Sickle cell disorders	1 (1 to 1)	1 (0 to 1)	-0.44 (-0.49 to -0.38)	22.99 (17.06 to 31.7)	8.16 (6.11 to 11.1)	-3.49 (-3.64 to -3.35)
Libya	Sickle cell trait	23 (20 to 26)	12 (11 to 14)	-0.46 (-0.48 to -0.43)	532.55 (465.86 to 617.91)	181.38 (159.27 to 209.05)	-3.67 (-3.75 to -3.58)

Regions	Causes	Cases in 1990 No.×103 (95% UI)	Cases in 2019 No.×103 (95% UI)	Change in absolute number (%)	Rates in 1990 per 100000 (95% UI)	Rates in 2019 per 100000 (95% UI)	EAPC from 1990 and 2019 (95%CI)
Libya	G6PD deficiency	10 (8 to 13)	5 (4 to 6)	-0.54 (-0.56 to -0.51)	238.79 (184.15 to 301.86)	69.53 (53.54 to 86.89)	-4.23 (-4.38 to -4.08)
Libya	G6PD trait	32 (30 to 34)	16 (15 to 17)	-0.5 (-0.51 to -0.49)	761.3 (707.35 to 808.97)	238.89 (220.36 to 255.29)	-3.96 (-4.05 to -3.88)
Lithuania	Inherited anemias	7 (6 to 8)	3 (3 to 4)	-0.51 (-0.52 to -0.5)	188.22 (166.58 to 210.84)	121.91 (107.83 to 136.9)	-0.75 (-1.24 to -0.25)
Lithuania	Thalassemias	0 (0 to 0)	0 (0 to 0)	-0.67 (-0.7 to -0.62)	0.75 (0.52 to 1.1)	0.33 (0.24 to 0.46)	-2.04 (-2.55 to -1.53)
Lithuania	Thalassemias trait	1 (1 to 2)	1 (1 to 1)	-0.5 (-0.53 to -0.47)	35.73 (29.7 to 43.75)	23.57 (19.71 to 28.5)	-0.69 (-1.19 to -0.19)
Lithuania	Sickle cell disorders	0 (0 to 0)	0 (0 to 0)	-0.51 (-0.6 to -0.42)	0.01 (0.01 to 0.01)	0.01 (0 to 0.01)	-0.75 (-1.22 to -0.28)
Lithuania	Sickle cell trait	0 (0 to 0)	0 (0 to 0)	-0.51 (-0.57 to -0.47)	7.11 (5.74 to 8.83)	4.56 (3.69 to 5.65)	-0.74 (-1.22 to -0.26)
Lithuania	G6PD deficiency	1 (1 to 1)	1 (0 to 1)	-0.48 (-0.52 to -0.45)	29.67 (22.94 to 36.89)	20.3 (15.75 to 25.81)	-0.87 (-1.35 to -0.39)
Lithuania	G6PD trait	4 (4 to 5)	2 (2 to 2)	-0.52 (-0.52 to -0.52)	114.96 (102.07 to 128.28)	73.16 (64.96 to 81.64)	-0.73 (-1.23 to -0.23)
Luxembourg	Inherited anemias	1 (1 to 1)	1 (1 to 1)	0.29 (0.25 to 0.33)	232.02 (208.89 to 256.93)	183.98 (164.34 to 204.22)	-1.1 (-1.2 to -1)
Luxembourg	Thalassemias	0 (0 to 0)	0 (0 to 0)	0.22 (0.08 to 0.4)	0.16 (0.11 to 0.22)	0.12 (0.09 to 0.17)	-2.12 (-2.92 to -1.31)
Luxembourg	Thalassemias trait	0 (0 to 0)	0 (0 to 0)	0.33 (0.25 to 0.41)	20.46 (17.61 to 23.46)	16.82 (14.58 to 19.27)	-1.19 (-1.43 to -0.96)
Luxembourg	Sickle cell disorders	0 (0 to 0)	0 (0 to 0)	0.21 (0.05 to 0.38)	0.53 (0.38 to 0.72)	0.4 (0.28 to 0.53)	-1.18 (-1.36 to -1)
Luxembourg	Sickle cell trait	0 (0 to 0)	0 (0 to 0)	0.09 (0 to 0.17)	29.3 (23.6 to 35.79)	19.61 (16.05 to 23.52)	-1.55 (-1.72 to -1.37)

Regions	Causes	Cases in 1990 No.×103 (95% UI)	Cases in 2019 No.×103 (95% UI)	Change in absolute number (%)	Rates in 1990 per 100000 (95% UI)	Rates in 2019 per 100000 (95% UI)	EAPC from 1990 and 2019 (95%CI)
Luxembourg	G6PD deficiency	0 (0 to 0)	0 (0 to 0)	0.38 (0.32 to 0.45)	35.27 (27.33 to 44.02)	29.97 (23.01 to 37.3)	-0.9 (-1.02 to -0.79)
Luxembourg	G6PD trait	1 (0 to 1)	1 (1 to 1)	0.3 (0.25 to 0.35)	146.29 (131.12 to 161.96)	117.05 (104.89 to 128.65)	-1.05 (-1.17 to -0.94)
Madagascar	Inherited anemias	253 (226 to 282)	330 (299 to 363)	0.3 (0.27 to 0.34)	2114.4 (1890.96 to 2355.65)	1234.68 (1121.44 to 1358.68)	-1.7 (-2 to -1.4)
Madagascar	Thalassemias	0 (0 to 0)	0 (0 to 0)	2.61 (2.04 to 3.25)	0.09 (0.05 to 0.14)	0.15 (0.08 to 0.23)	1.25 (1.01 to 1.49)
Madagascar	Thalassemias trait	3 (2 to 4)	7 (5 to 9)	1.22 (1.04 to 1.42)	26.34 (20.77 to 31.87)	26.18 (20.5 to 32.13)	-0.24 (-0.38 to -0.1)
Madagascar	Sickle cell disorders	2 (2 to 3)	3 (3 to 4)	0.56 (0.41 to 0.72)	18.11 (14.43 to 23.03)	12.65 (10.15 to 16.17)	-1.45 (-1.63 to -1.26)
Madagascar	Sickle cell trait	64 (57 to 71)	101 (91 to 113)	0.58 (0.5 to 0.65)	533.12 (480.22 to 597.3)	376.84 (340.34 to 423.52)	-1.33 (-1.46 to -1.19)
Madagascar	G6PD deficiency	78 (60 to 97)	70 (54 to 88)	-0.1 (-0.17 to -0.05)	652.39 (505.18 to 810.96)	262.47 (203.77 to 329.44)	-2.58 (-3.25 to -1.91)
Madagascar	G6PD trait	106 (97 to 113)	149 (136 to 161)	0.41 (0.37 to 0.44)	884.34 (815.07 to 947.23)	556.39 (507.75 to 601.7)	-1.47 (-1.69 to -1.25)
Malawi	Inherited anemias	166 (148 to 185)	178 (159 to 198)	0.08 (0.06 to 0.09)	1733.65 (1544.03 to 1934.37)	965.65 (864.22 to 1076.29)	-2.19 (-2.48 to -1.9)
Malawi	Thalassemias	0 (0 to 0)	0 (0 to 0)	1.61 (1.31 to 1.98)	0.1 (0.06 to 0.15)	0.14 (0.08 to 0.21)	0.53 (0.22 to 0.84)
Malawi	Thalassemias trait	3 (2 to 3)	4 (4 to 5)	0.61 (0.51 to 0.74)	29 (23.06 to 35.64)	24.23 (19.21 to 29.34)	-0.97 (-1.22 to -0.73)
Malawi	Sickle cell disorders	0 (0 to 1)	1 (0 to 1)	0.18 (0.08 to 0.29)	4.61 (3.66 to 5.97)	2.81 (2.23 to 3.64)	-1.78 (-2.05 to -1.5)
Malawi	Sickle cell trait	27 (25 to 31)	32 (29 to 37)	0.17 (0.12 to 0.22)	287.5 (257.06 to 327.54)	173.99 (155.53 to 197.96)	-1.88 (-2.12 to -1.64)

Regions	Causes	Cases in 1990 No.×103 (95% UI)	Cases in 2019 No.×103 (95% UI)	Change in absolute number (%)	Rates in 1990 per 100000 (95% UI)	Rates in 2019 per 100000 (95% UI)	EAPC from 1990 and 2019 (95%CI)
Malawi	G6PD deficiency	48 (37 to 60)	45 (35 to 56)	-0.05 (-0.07 to -0.04)	498.72 (384.49 to 624.52)	244.27 (189.09 to 304.49)	-2.65 (-3.05 to -2.24)
Malawi	G6PD trait	87 (80 to 94)	96 (87 to 104)	0.1 (0.09 to 0.1)	913.73 (836.13 to 983.96)	520.22 (474.35 to 562.61)	-2.11 (-2.37 to -1.85)
Malaysia	Inherited anemias	148 (133 to 166)	94 (84 to 104)	-0.37 (-0.4 to -0.34)	841 (751.66 to 938.62)	299.22 (267.46 to 332.39)	-3.95 (-4.44 to -3.45)
Malaysia	Thalassemias	0 (0 to 0)	0 (0 to 0)	0.2 (0.04 to 0.36)	1.58 (1.27 to 1.99)	1.07 (0.83 to 1.37)	-1.75 (-2.04 to -1.45)
Malaysia	Thalassemias trait	14 (13 to 16)	18 (16 to 20)	0.27 (0.22 to 0.31)	81.4 (72.1 to 90.61)	58.33 (51.46 to 64.65)	-1.54 (-1.81 to -1.28)
Malaysia	Sickle cell disorders	0 (0 to 0)	0 (0 to 0)	0.06 (-0.29 to 0.3)	0.02 (0.01 to 0.02)	0.01 (0.01 to 0.01)	-2.09 (-2.36 to -1.82)
Malaysia	Sickle cell trait	2 (2 to 3)	2 (2 to 3)	0.08 (-0.11 to 0.21)	12.87 (10.36 to 15.33)	7.83 (6.38 to 9.34)	-2.07 (-2.33 to -1.81)
Malaysia	G6PD deficiency	42 (34 to 53)	18 (14 to 22)	-0.58 (-0.62 to -0.53)	240.65 (190.77 to 301.46)	56.41 (43.8 to 70.3)	-5.29 (-6.23 to -4.34)
Malaysia	G6PD trait	89 (82 to 96)	55 (49 to 61)	-0.38 (-0.42 to -0.35)	504.48 (466.14 to 542.06)	175.57 (156.88 to 193.33)	-4.01 (-4.48 to -3.54)
Maldives	Inherited anemias	3 (3 to 4)	3 (3 to 3)	-0.12 (-0.16 to -0.09)	1498.82 (1359.28 to 1645.81)	584.5 (527.89 to 645.75)	-2.7 (-3 to -2.39)
Maldives	Thalassemias	0 (0 to 0)	0 (0 to 0)	-0.13 (-0.24 to 0.01)	27.01 (20.34 to 35.18)	10.49 (8.01 to 13.62)	-2.77 (-3.12 to -2.43)
Maldives	Thalassemias trait	1 (1 to 2)	1 (1 to 2)	-0.05 (-0.11 to 0.02)	637.17 (559.15 to 723.49)	269.41 (236.96 to 306.7)	-2.44 (-2.75 to -2.13)
Maldives	Sickle cell disorders	0 (0 to 0)	0 (0 to 0)	-0.06 (-0.36 to 0.17)	0.03 (0.02 to 0.04)	0.01 (0.01 to 0.02)	-2.34 (-2.66 to -2.01)
Maldives	Sickle cell trait	0 (0 to 0)	0 (0 to 0)	-0.05 (-0.19 to 0.07)	20.25 (16.87 to 24.48)	8.61 (7.15 to 10.44)	-2.33 (-2.63 to -2.04)

Regions	Causes	Cases in 1990 No.×103 (95% UI)	Cases in 2019 No.×103 (95% UI)	Change in absolute number (%)	Rates in 1990 per 100000 (95% UI)	Rates in 2019 per 100000 (95% UI)	EAPC from 1990 and 2019 (95%CI)
Maldives	G6PD deficiency	1 (0 to 1)	0 (0 to 1)	-0.19 (-0.24 to -0.12)	238.81 (185.2 to 301.31)	86.43 (66.59 to 107.77)	-2.91 (-3.26 to -2.57)
Maldives	G6PD trait	1 (1 to 1)	1 (1 to 1)	-0.18 (-0.21 to -0.16)	575.54 (518.83 to 627.34)	209.56 (188.32 to 231.33)	-2.92 (-3.25 to -2.58)
Mali	**Inherited anemias**	141 (128 to 154)	302 (276 to 331)	1.15 (1.09 to 1.2)	1622.17 (1473.83 to 1778.26)	1379.93 (1259.26 to 1510.83)	-0.59 (-0.61 to -0.57)
Mali	Thalassemias	0 (0 to 0)	0 (0 to 0)	2.88 (2.36 to 3.39)	0.16 (0.11 to 0.22)	0.24 (0.15 to 0.35)	1.3 (1.17 to 1.43)
Mali	Thalassemias trait	5 (4 to 6)	12 (10 to 14)	1.57 (1.42 to 1.74)	55.36 (46.74 to 65.21)	56.34 (47.77 to 65.97)	-0.03 (-0.07 to 0.02)
Mali	Sickle cell disorders	3 (3 to 3)	6 (5 to 7)	1.12 (1 to 1.27)	33.86 (29.17 to 39.24)	28.43 (24.48 to 32.91)	-0.65 (-0.68 to -0.63)
Mali	Sickle cell trait	49 (45 to 54)	105 (95 to 116)	1.13 (1.04 to 1.25)	567.75 (513.92 to 628.11)	479.53 (432.18 to 531.29)	-0.62 (-0.64 to -0.6)
Mali	G6PD deficiency	25 (19 to 31)	52 (40 to 66)	1.12 (0.94 to 1.31)	283.37 (218.27 to 353.9)	237.94 (183.94 to 299.39)	-0.63 (-0.64 to -0.61)
Mali	G6PD trait	59 (53 to 65)	127 (114 to 138)	1.14 (1.08 to 1.2)	681.66 (613.24 to 749.45)	577.44 (519.41 to 631.47)	-0.6 (-0.61 to -0.58)
Malta	**Inherited anemias**	1 (1 to 1)	1 (1 to 1)	-0.23 (-0.25 to -0.21)	272.42 (245.13 to 301.5)	176.98 (158.35 to 196.44)	-1.34 (-1.68 to -1)
Malta	Thalassemias	0 (0 to 0)	0 (0 to 0)	-0.28 (-0.38 to -0.16)	0.19 (0.13 to 0.26)	0.12 (0.08 to 0.17)	-2.38 (-3 to -1.75)
Malta	Thalassemias trait	0 (0 to 0)	0 (0 to 0)	-0.22 (-0.27 to -0.17)	23.98 (20.58 to 27.51)	15.74 (13.65 to 18.06)	-1.5 (-1.77 to -1.23)
Malta	Sickle cell disorders	0 (0 to 0)	0 (0 to 0)	-0.15 (-0.24 to -0.05)	0.21 (0.15 to 0.3)	0.15 (0.11 to 0.22)	-1.03 (-1.32 to -0.74)
Malta	Sickle cell trait	0 (0 to 0)	0 (0 to 0)	-0.19 (-0.24 to -0.14)	32.11 (26.24 to 38.75)	21.87 (17.76 to 26.68)	-1.19 (-1.52 to -0.86)

Regions	Causes	Cases in 1990 No.×103 (95% UI)	Cases in 2019 No.×103 (95% UI)	Change in absolute number (%)	Rates in 1990 per 100000 (95% UI)	Rates in 2019 per 100000 (95% UI)	EAPC from 1990 and 2019 (95%CI)
Malta	G6PD deficiency	0 (0 to 0)	0 (0 to 0)	-0.19 (-0.24 to -0.14)	42.73 (32.96 to 53.14)	29.05 (22.33 to 36.23)	-1.19 (-1.58 to -0.79)
Malta	G6PD trait	1 (1 to 1)	0 (0 to 1)	-0.25 (-0.27 to -0.22)	173.19 (155.77 to 191.36)	110.05 (98.57 to 121.57)	-1.39 (-1.73 to -1.04)
Marshall Islands	Inherited anemias	0 (0 to 0)	0 (0 to 0)	-0.08 (-0.1 to -0.07)	706.18 (626.81 to 790.95)	523.78 (462.99 to 583.13)	-0.74 (-0.85 to -0.63)
Marshall Islands	Thalassemias	0 (0 to 0)	0 (0 to 0)	-0.24 (-0.33 to -0.14)	3.94 (2.06 to 6.15)	2.4 (1.29 to 3.66)	-0.33 (-1.26 to 0.6)
Marshall Islands	Thalassemias trait	0 (0 to 0)	0 (0 to 0)	-0.19 (-0.24 to -0.15)	115.74 (87.68 to 142)	75.23 (57.22 to 91.49)	-0.78 (-1.16 to -0.4)
Marshall Islands	Sickle cell disorders	0 (0 to 0)	0 (0 to 0)	-0.13 (-0.18 to 0.55)	0 (0 to 0)	0 (0 to 0)	-0.87 (-1.04 to -0.7)
Marshall Islands	Sickle cell trait	0 (0 to 0)	0 (0 to 0)	-0.14 (-0.18 to 0.36)	4.41 (1.9 to 6.8)	3.05 (1.26 to 4.62)	-0.96 (-1.11 to -0.82)
Marshall Islands	G6PD deficiency	0 (0 to 0)	0 (0 to 0)	0 (-0.06 to 0.05)	145.54 (111.78 to 181.73)	116.91 (90.23 to 144.9)	-0.55 (-0.67 to -0.42)
Marshall Islands	G6PD trait	0 (0 to 0)	0 (0 to 0)	-0.07 (-0.08 to -0.07)	436.56 (392.47 to 477.5)	326.19 (294.57 to 354.95)	-0.81 (-0.89 to -0.74)
Mauritania	Inherited anemias	29 (27 to 33)	36 (32 to 40)	0.22 (0.19 to 0.24)	1424.92 (1283.95 to 1577.76)	893.16 (804.88 to 986.19)	-1.57 (-1.69 to -1.45)
Mauritania	Thalassemias	0 (0 to 0)	0 (0 to 0)	0.6 (0.37 to 0.87)	0.29 (0.2 to 0.4)	0.24 (0.17 to 0.33)	-0.69 (-0.79 to -0.59)
Mauritania	Thalassemias trait	2 (1 to 2)	2 (2 to 3)	0.34 (0.26 to 0.43)	77.97 (65.27 to 92.82)	53.97 (45.29 to 64.53)	-1.26 (-1.38 to -1.15)
Mauritania	Sickle cell disorders	0 (0 to 0)	0 (0 to 0)	0.28 (0.18 to 0.4)	8.22 (6.5 to 10.2)	5.4 (4.31 to 6.67)	-1.37 (-1.5 to -1.24)
Mauritania	Sickle cell trait	7 (6 to 8)	9 (8 to 10)	0.24 (0.19 to 0.31)	335.78 (296.32 to 377.8)	215.16 (190.81 to 241.21)	-1.48 (-1.6 to -1.36)

Regions	Causes	Cases in 1990 No.×103 (95% UI)	Cases in 2019 No.×103 (95% UI)	Change in absolute number (%)	Rates in 1990 per 100000 (95% UI)	Rates in 2019 per 100000 (95% UI)	EAPC from 1990 and 2019 (95%CI)
Mauritania	G6PD deficiency	7 (5 to 8)	8 (6 to 10)	0.17 (0.09 to 0.25)	321.66 (248.29 to 406.93)	193.18 (149.32 to 239.38)	-1.68 (-1.83 to -1.53)
Mauritania	G6PD trait	14 (13 to 15)	17 (15 to 19)	0.21 (0.19 to 0.26)	681 (617.61 to 743.35)	425.21 (385.09 to 462.01)	-1.61 (-1.73 to -1.49)
Mauritius	**Inherited anemias**	5 (5 to 6)	3 (3 to 3)	-0.46 (-0.48 to -0.44)	476.8 (429.05 to 525.67)	221.82 (199.5 to 244.63)	-3.01 (-3.19 to -2.83)
Mauritius	Thalassemias	0 (0 to 0)	0 (0 to 0)	-0.39 (-0.48 to -0.29)	2.51 (1.86 to 3.43)	1.33 (0.94 to 1.8)	-2.53 (-2.69 to -2.37)
Mauritius	Thalassemias trait	1 (1 to 1)	1 (1 to 1)	-0.37 (-0.4 to -0.34)	104.13 (88.77 to 122.63)	56.82 (48.47 to 66.22)	-2.42 (-2.57 to -2.27)
Mauritius	Sickle cell disorders	0 (0 to 0)	0 (0 to 0)	-0.42 (-0.54 to -0.32)	0.01 (0.01 to 0.02)	0.01 (0 to 0.01)	-2.76 (-2.9 to -2.62)
Mauritius	Sickle cell trait	0 (0 to 0)	0 (0 to 0)	-0.42 (-0.49 to -0.36)	9.5 (7.97 to 11.35)	4.72 (3.95 to 5.63)	-2.74 (-2.89 to -2.6)
Mauritius	G6PD deficiency	1 (1 to 1)	0 (0 to 1)	-0.41 (-0.45 to -0.37)	72.95 (56.03 to 90.96)	37.31 (28.7 to 46.36)	-2.9 (-3.09 to -2.7)
Mauritius	G6PD trait	3 (3 to 3)	2 (1 to 2)	-0.51 (-0.53 to -0.49)	287.68 (259.1 to 313.84)	121.64 (109.04 to 133.17)	-3.29 (-3.52 to -3.07)
Mexico	**Inherited anemias**	425 (414 to 436)	245 (238 to 253)	-0.42 (-0.43 to -0.42)	496.96 (484.47 to 510.6)	195.9 (190.21 to 202.59)	-3.19 (-3.56 to -2.82)
Mexico	Thalassemias	0 (0 to 0)	0 (0 to 0)	0.31 (-0.2 to 1.51)	0.02 (0.01 to 0.03)	0.02 (0.01 to 0.02)	-0.41 (-0.59 to -0.23)
Mexico	Thalassemias trait	10 (6 to 16)	10 (6 to 15)	-0.04 (-0.24 to 0.26)	12.05 (7.22 to 18.54)	7.9 (5.04 to 11.62)	-1.53 (-1.62 to -1.45)
Mexico	Sickle cell disorders	0 (0 to 0)	0 (0 to 0)	-0.36 (-0.51 to -0.21)	0.08 (0.05 to 0.13)	0.04 (0.02 to 0.06)	-2.47 (-2.73 to -2.2)
Mexico	Sickle cell trait	20 (15 to 27)	15 (12 to 20)	-0.24 (-0.32 to -0.16)	23.67 (17.61 to 31.43)	12.26 (9.54 to 16.02)	-2.1 (-2.18 to -2.02)

Regions	Causes	Cases in 1990 No.×103 (95% UI)	Cases in 2019 No.×103 (95% UI)	Change in absolute number (%)	Rates in 1990 per 100000 (95% UI)	Rates in 2019 per 100000 (95% UI)	EAPC from 1990 and 2019 (95%CI)
Mexico	G6PD deficiency	54 (51 to 56)	23 (22 to 24)	-0.57 (-0.58 to -0.56)	62.71 (60.17 to 65.08)	18.45 (17.54 to 19.31)	-3.65 (-4.24 to -3.05)
Mexico	G6PD trait	341 (334 to 346)	196 (192 to 200)	-0.42 (-0.43 to -0.42)	398.44 (390.86 to 404.94)	157.24 (153.81 to 160.44)	-3.26 (-3.65 to -2.87)
Micronesia (Federated States of)	Inherited anemias	1 (1 to 1)	0 (0 to 1)	-0.35 (-0.36 to -0.34)	688.71 (613.51 to 766.46)	457.07 (406.67 to 510.72)	-1.38 (-1.61 to -1.14)
Micronesia (Federated States of)	Thalassemias	0 (0 to 0)	0 (0 to 0)	-0.49 (-0.57 to -0.41)	3.99 (2.09 to 6.05)	2.06 (1.1 to 3.12)	-1.22 (-2.14 to -0.28)
Micronesia (Federated States of)	Thalassemias trait	0 (0 to 0)	0 (0 to 0)	-0.46 (-0.5 to -0.42)	117.61 (89.26 to 142.36)	65.08 (49.9 to 78.91)	-1.61 (-1.98 to -1.23)
Micronesia (Federated States of)	Sickle cell disorders	0 (0 to 0)	0 (0 to 0)	-0.41 (-0.45 to 0.14)	0 (0 to 0)	0 (0 to 0)	-1.63 (-1.77 to -1.5)
Micronesia (Federated States of)	Sickle cell trait	0 (0 to 0)	0 (0 to 0)	-0.42 (-0.45 to 0.12)	4.46 (1.82 to 6.82)	2.66 (1.09 to 4.16)	-1.73 (-1.84 to -1.63)
Micronesia (Federated States of)	G6PD deficiency	0 (0 to 0)	0 (0 to 0)	-0.13 (-0.18 to -0.09)	115.6 (88.8 to 144.18)	102.26 (79.24 to 127.85)	-0.43 (-0.95 to 0.09)
Micronesia (Federated States of)	G6PD trait	0 (0 to 1)	0 (0 to 0)	-0.38 (-0.38 to -0.37)	447.04 (401.88 to 488.9)	285 (258.07 to 310.03)	-1.61 (-1.77 to -1.44)
Monaco	Inherited anemias	0 (0 to 0)	0 (0 to 0)	-0.12 (-0.15 to -0.09)	193.77 (173.92 to 214.12)	137.46 (122.97 to 152.61)	-1.32 (-1.42 to -1.21)
Monaco	Thalassemias	0 (0 to 0)	0 (0 to 0)	-0.17 (-0.27 to -0.04)	0.16 (0.11 to 0.22)	0.11 (0.07 to 0.15)	-2.35 (-3.15 to -1.55)
Monaco	Thalassemias trait	0 (0 to 0)	0 (0 to 0)	-0.1 (-0.16 to -0.04)	17.89 (15.52 to 20.58)	13 (11.25 to 14.97)	-1.45 (-1.65 to -1.24)
Monaco	Sickle cell disorders	0 (0 to 0)	0 (0 to 0)	-0.15 (-0.25 to -0.05)	0.21 (0.15 to 0.28)	0.14 (0.1 to 0.2)	-1.46 (-1.57 to -1.35)
Monaco	Sickle cell trait	0 (0 to 0)	0 (0 to 0)	-0.14 (-0.2 to -0.07)	24.96 (20.43 to 30.3)	17.37 (14.18 to 21.04)	-1.39 (-1.5 to -1.28)

**Regions**	**Causes**	**Cases in 1990 No.×103 (95% UI)**	**Cases in 2019 No.×103 (95% UI)**	**Change in absolute number (%)**	**Rates in 1990 per 100000 (95% UI)**	**Rates in 2019 per 100000 (95% UI)**	**EAPC from 1990 and 2019 (95%CI)**
Monaco	G6PD deficiency	0 (0 to 0)	0 (0 to 0)	-0.08 (-0.11 to -0.04)	29.43 (22.64 to 36.67)	21.92 (16.84 to 27.52)	-1.15 (-1.3 to -1.01)
Monaco	G6PD trait	0 (0 to 0)	0 (0 to 0)	-0.13 (-0.17 to -0.09)	121.13 (108.6 to 134.13)	84.92 (76.01 to 93.5)	-1.32 (-1.42 to -1.22)
Mongolia	Inherited anemias	11 (10 to 12)	12 (11 to 14)	0.12 (0.08 to 0.15)	519.01 (464.99 to 578.24)	368.07 (329 to 408.49)	-0.23 (-0.69 to 0.24)
Mongolia	Thalassemias	0 (0 to 0)	0 (0 to 0)	-0.12 (-0.22 to 0)	4.48 (3.24 to 6.29)	2.51 (1.82 to 3.43)	-1.02 (-1.51 to -0.54)
Mongolia	Thalassemias trait	4 (3 to 5)	4 (4 to 5)	0.12 (0.04 to 0.21)	183.19 (152.34 to 223.11)	130.71 (109.27 to 157.63)	-0.23 (-0.71 to 0.24)
Mongolia	Sickle cell disorders	0 (0 to 0)	0 (0 to 0)	0.12 (-0.08 to 0.27)	0.02 (0.01 to 0.03)	0.02 (0.01 to 0.02)	-0.22 (-0.69 to 0.25)
Mongolia	Sickle cell trait	0 (0 to 0)	0 (0 to 0)	0.12 (0.01 to 0.2)	16.29 (12.82 to 20.19)	11.57 (9.09 to 14.37)	-0.23 (-0.69 to 0.24)
Mongolia	G6PD deficiency	1 (1 to 1)	1 (1 to 1)	0.13 (0.05 to 0.2)	46.68 (36.06 to 59.08)	33.67 (26.04 to 42.66)	-0.11 (-0.59 to 0.38)
Mongolia	G6PD trait	6 (5 to 6)	6 (6 to 7)	0.11 (0.11 to 0.11)	268.35 (239.05 to 299.06)	189.59 (168.89 to 211.29)	-0.23 (-0.69 to 0.24)
Montenegro	Inherited anemias	1 (1 to 2)	1 (1 to 1)	-0.29 (-0.3 to -0.29)	223.36 (197.11 to 252.05)	158.96 (140.22 to 179.87)	-1.31 (-1.38 to -1.24)
Montenegro	Thalassemias	0 (0 to 0)	0 (0 to 0)	-0.57 (-0.62 to -0.5)	1.21 (0.79 to 1.89)	0.52 (0.36 to 0.79)	-2.8 (-2.84 to -2.75)
Montenegro	Thalassemias trait	0 (0 to 0)	0 (0 to 0)	-0.28 (-0.33 to -0.23)	28.78 (23.97 to 35.47)	20.89 (17.5 to 25.05)	-1.07 (-1.11 to -1.02)
Montenegro	Sickle cell disorders	0 (0 to 0)	0 (0 to 0)	-0.23 (-0.3 to -0.14)	0.13 (0.1 to 0.17)	0.1 (0.07 to 0.13)	-0.88 (-1.08 to -0.67)
Montenegro	Sickle cell trait	0 (0 to 0)	0 (0 to 0)	-0.27 (-0.31 to -0.24)	26.59 (22.82 to 30.69)	19.48 (16.72 to 22.54)	-1.02 (-1.13 to -0.92)

Regions	Causes	Cases in 1990 No.×103 (95% UI)	Cases in 2019 No.×103 (95% UI)	Change in absolute number (%)	Rates in 1990 per 100000 (95% UI)	Rates in 2019 per 100000 (95% UI)	EAPC from 1990 and 2019 (95%CI)
Montenegro	G6PD deficiency	0 (0 to 0)	0 (0 to 0)	-0.25 (-0.26 to -0.25)	54.82 (42.44 to 68.64)	41.38 (32.02 to 51.8)	-1.86 (-2.2 to -1.51)
Montenegro	G6PD trait	1 (1 to 1)	0 (0 to 1)	-0.32 (-0.32 to -0.32)	111.84 (99.09 to 125.12)	76.58 (67.85 to 85.68)	-1.2 (-1.24 to -1.15)
Morocco	Inherited anemias	261 (238 to 285)	199 (182 to 218)	-0.24 (-0.25 to -0.22)	1030.88 (942.05 to 1126.59)	553.66 (506.5 to 605.88)	-2.04 (-2.22 to -1.85)
Morocco	Thalassemias	1 (1 to 1)	1 (1 to 1)	-0.23 (-0.3 to -0.15)	3.69 (2.93 to 4.77)	2.01 (1.57 to 2.58)	-2 (-2.19 to -1.8)
Morocco	Thalassemias trait	56 (51 to 64)	44 (39 to 49)	-0.22 (-0.25 to -0.19)	222.7 (200.31 to 251.51)	122.1 (109.07 to 137.33)	-1.98 (-2.17 to -1.79)
Morocco	Sickle cell disorders	0 (0 to 0)	0 (0 to 0)	-0.25 (-0.29 to -0.2)	1.11 (0.93 to 1.31)	0.59 (0.49 to 0.7)	-2.08 (-2.27 to -1.89)
Morocco	Sickle cell trait	29 (27 to 32)	22 (20 to 24)	-0.25 (-0.27 to -0.22)	114.64 (105.02 to 125.02)	60.82 (55.69 to 66.58)	-2.08 (-2.27 to -1.89)
Morocco	G6PD deficiency	45 (35 to 56)	34 (26 to 43)	-0.24 (-0.28 to -0.2)	178.75 (137.69 to 222.85)	95.31 (73.67 to 120.14)	-2.05 (-2.23 to -1.86)
Morocco	G6PD trait	129 (117 to 140)	98 (89 to 107)	-0.24 (-0.26 to -0.22)	510 (462.17 to 552.9)	272.84 (247.72 to 296.71)	-2.05 (-2.23 to -1.87)
Mozambique	Inherited anemias	206 (193 to 221)	371 (323 to 422)	0.8 (0.68 to 0.91)	1579.19 (1473.38 to 1692.03)	1255.2 (1094.38 to 1429.86)	-0.9 (-1.08 to -0.72)
Mozambique	Thalassemias	0 (0 to 0)	0 (0 to 0)	3.2 (2.69 to 3.93)	0.1 (0.06 to 0.15)	0.18 (0.1 to 0.27)	1.71 (1.44 to 1.98)
Mozambique	Thalassemias trait	4 (3 to 4)	9 (7 to 11)	1.59 (1.41 to 1.8)	27.2 (21.46 to 33.22)	31.18 (24.58 to 37.9)	0.2 (0.02 to 0.38)
Mozambique	Sickle cell disorders	0 (0 to 0)	0 (0 to 0)	0.9 (0.79 to 1.01)	1 (0.81 to 1.21)	0.85 (0.69 to 1.02)	-0.72 (-0.85 to -0.58)
Mozambique	Sickle cell trait	17 (15 to 19)	31 (28 to 35)	0.88 (0.82 to 0.94)	127.87 (114.37 to 141.77)	106.54 (95.58 to 117.8)	-0.76 (-0.9 to -0.62)

Regions	Causes	Cases in 1990 No.×103 (95% UI)	Cases in 2019 No.×103 (95% UI)	Change in absolute number (%)	Rates in 1990 per 100000 (95% UI)	Rates in 2019 per 100000 (95% UI)	EAPC from 1990 and 2019 (95%CI)
Mozambique	G6PD deficiency	75 (69 to 81)	134 (104 to 169)	0.79 (0.48 to 1.11)	570.42 (526.84 to 618.17)	453.18 (351.45 to 572.59)	-0.92 (-1.11 to -0.73)
Mozambique	G6PD trait	111 (102 to 120)	196 (179 to 211)	0.76 (0.72 to 0.79)	852.6 (780.68 to 916.97)	663.27 (604.95 to 714.75)	-0.96 (-1.14 to -0.77)
Myanmar	Inherited anemias	467 (418 to 517)	382 (344 to 422)	-0.18 (-0.21 to -0.16)	1135.73 (1017.39 to 1257.77)	697.79 (629.24 to 771)	-0.84 (-1.28 to -0.39)
Myanmar	Thalassemias	4 (3 to 5)	5 (4 to 6)	0.29 (0.19 to 0.39)	8.86 (7.03 to 11.31)	8.58 (6.65 to 11.11)	-0.07 (-0.11 to -0.04)
Myanmar	Thalassemias trait	104 (95 to 115)	96 (87 to 106)	-0.08 (-0.11 to -0.05)	252.24 (230.25 to 279.87)	175.06 (159.55 to 194.15)	-1.23 (-1.26 to -1.2)
Myanmar	Sickle cell disorders	0 (0 to 0)	0 (0 to 0)	-0.21 (-0.31 to -0.03)	0.03 (0.02 to 0.04)	0.02 (0.01 to 0.02)	-1.75 (-1.8 to -1.7)
Myanmar	Sickle cell trait	8 (6 to 9)	6 (5 to 7)	-0.21 (-0.26 to -0.12)	18.31 (15.05 to 21.93)	10.83 (8.95 to 12.79)	-1.77 (-1.8 to -1.73)
Myanmar	G6PD deficiency	120 (93 to 150)	94 (73 to 117)	-0.21 (-0.26 to -0.15)	291.25 (225.81 to 364.79)	172.65 (133.72 to 214.17)	0.15 (-0.87 to 1.18)
Myanmar	G6PD trait	232 (212 to 251)	181 (165 to 195)	-0.22 (-0.25 to -0.2)	565.04 (515.39 to 610.37)	330.65 (302.27 to 356.67)	-1.13 (-1.5 to -0.76)
Namibia	Inherited anemias	14 (12 to 16)	18 (16 to 20)	0.26 (0.22 to 0.3)	1008.23 (884.88 to 1128.88)	746.38 (656.12 to 843.43)	-0.91 (-0.98 to -0.84)
Namibia	Thalassemias	0 (0 to 0)	0 (0 to 0)	1.74 (1.3 to 2.26)	0.07 (0.05 to 0.1)	0.11 (0.07 to 0.17)	1.47 (1.33 to 1.62)
Namibia	Thalassemias trait	0 (0 to 0)	1 (0 to 1)	0.68 (0.54 to 0.83)	25.47 (21.42 to 29.81)	25.07 (20.46 to 29.56)	-0.06 (-0.12 to 0)
Namibia	Sickle cell disorders	0 (0 to 0)	0 (0 to 0)	0.21 (0.11 to 0.3)	0.27 (0.2 to 0.36)	0.19 (0.14 to 0.25)	-0.87 (-1.14 to -0.61)
Namibia	Sickle cell trait	1 (1 to 1)	1 (1 to 1)	0.25 (0.19 to 0.31)	56.4 (48.16 to 65.96)	41.45 (35.28 to 48.25)	-0.85 (-1.01 to -0.69)

Regions	Causes	Cases in 1990 No.×103 (95% UI)	Cases in 2019 No.×103 (95% UI)	Change in absolute number (%)	Rates in 1990 per 100000 (95% UI)	Rates in 2019 per 100000 (95% UI)	EAPC from 1990 and 2019 (95%CI)
Namibia	G6PD deficiency	4 (3 to 5)	5 (4 to 7)	0.21 (0.13 to 0.3)	314.76 (243.74 to 388.48)	223.09 (172.9 to 281.47)	-1.07 (-1.17 to -0.98)
Namibia	G6PD trait	9 (8 to 9)	11 (10 to 12)	0.27 (0.23 to 0.31)	611.27 (560.23 to 659.57)	456.46 (416.59 to 495.47)	-0.88 (-0.94 to -0.82)
Nauru	**Inherited anemias**	0 (0 to 0)	0 (0 to 0)	-0.23 (-0.25 to -0.21)	926.81 (822.79 to 1032.51)	695.82 (617.09 to 777.52)	-0.99 (-1.12 to -0.86)
Nauru	Thalassemias	0 (0 to 0)	0 (0 to 0)	-0.37 (-0.44 to -0.27)	5.43 (3.01 to 8.14)	3.31 (1.79 to 5.11)	-0.6 (-1.52 to 0.34)
Nauru	Thalassemias trait	0 (0 to 0)	0 (0 to 0)	-0.33 (-0.36 to -0.28)	153.61 (118.26 to 185.17)	100.75 (77.29 to 123.17)	-1.03 (-1.4 to -0.66)
Nauru	Sickle cell disorders	0 (0 to 0)	0 (0 to 0)	-0.28 (-0.31 to 0.08)	0 (0 to 0.01)	0 (0 to 0)	-1.18 (-1.37 to -1)
Nauru	Sickle cell trait	0 (0 to 0)	0 (0 to 0)	-0.29 (-0.31 to -0.02)	5.69 (2.44 to 8.79)	3.93 (1.64 to 5.97)	-1.25 (-1.39 to -1.11)
Nauru	G6PD deficiency	0 (0 to 0)	0 (0 to 0)	-0.16 (-0.21 to -0.11)	191.98 (148.31 to 238.3)	157.2 (121.34 to 198.57)	-0.77 (-0.95 to -0.59)
Nauru	G6PD trait	0 (0 to 0)	0 (0 to 0)	-0.22 (-0.25 to -0.2)	570.09 (512.78 to 623.39)	430.63 (389.59 to 468.6)	-1.06 (-1.17 to -0.96)
Nepal	**Inherited anemias**	104 (93 to 116)	82 (74 to 91)	-0.21 (-0.23 to -0.19)	532.1 (476.62 to 594.08)	270.22 (242.11 to 300.64)	-2.38 (-2.52 to -2.24)
Nepal	Thalassemias	0 (0 to 0)	0 (0 to 0)	0.23 (0.13 to 0.32)	0.78 (0.58 to 1.01)	0.62 (0.45 to 0.82)	-1.11 (-1.22 to -1)
Nepal	Thalassemias trait	19 (16 to 21)	16 (14 to 18)	-0.13 (-0.16 to -0.1)	95.3 (83.47 to 108.24)	53.51 (46.77 to 60.51)	-2.26 (-2.37 to -2.16)
Nepal	Sickle cell disorders	0 (0 to 0)	0 (0 to 0)	-0.08 (-0.23 to 0.16)	0.09 (0.06 to 0.12)	0.05 (0.04 to 0.07)	-1.79 (-1.91 to -1.66)
Nepal	Sickle cell trait	6 (5 to 7)	4 (3 to 6)	-0.25 (-0.3 to -0.18)	30.16 (23.53 to 38.18)	14.53 (11.44 to 18.34)	-2.74 (-2.83 to -2.65)

Regions	Causes	Cases in 1990 No.×103 (95% UI)	Cases in 2019 No.×103 (95% UI)	Change in absolute number (%)	Rates in 1990 per 100000 (95% UI)	Rates in 2019 per 100000 (95% UI)	EAPC from 1990 and 2019 (95%CI)
Nepal	G6PD deficiency	14 (11 to 18)	12 (9 to 15)	-0.18 (-0.22 to -0.12)	73.05 (56.58 to 90.86)	38.47 (29.65 to 48.01)	-1.99 (-2.31 to -1.66)
Nepal	G6PD trait	65 (58 to 72)	50 (44 to 56)	-0.24 (-0.27 to -0.21)	332.72 (296.84 to 369.94)	163.04 (145.28 to 182.61)	-2.46 (-2.6 to -2.33)
Netherlands	Inherited anemias	32 (29 to 36)	29 (26 to 32)	-0.11 (-0.15 to -0.07)	216.72 (192.79 to 241.95)	166.94 (148.95 to 186.74)	-1 (-1.18 to -0.82)
Netherlands	Thalassemias	0 (0 to 0)	0 (0 to 0)	-0.19 (-0.33 to -0.03)	0.15 (0.1 to 0.22)	0.11 (0.07 to 0.16)	-2.21 (-3.15 to -1.26)
Netherlands	Thalassemias trait	3 (2 to 3)	2 (2 to 3)	-0.1 (-0.16 to -0.04)	17.61 (15.09 to 20.3)	13.77 (11.83 to 15.87)	-1.09 (-1.49 to -0.69)
Netherlands	Sickle cell disorders	0 (0 to 0)	0 (0 to 0)	-0.09 (-0.19 to 0.03)	0.03 (0.02 to 0.03)	0.02 (0.02 to 0.03)	1.44 (0.22 to 2.67)
Netherlands	Sickle cell trait	1 (1 to 2)	1 (1 to 1)	-0.09 (-0.14 to 0.01)	9.16 (7.74 to 10.98)	7.22 (6.14 to 8.67)	0.58 (-0.2 to 1.37)
Netherlands	G6PD deficiency	5 (4 to 7)	5 (4 to 6)	-0.07 (-0.13 to -0.02)	36.51 (28.03 to 45.89)	29.56 (22.97 to 36.94)	-0.95 (-1.09 to -0.82)
Netherlands	G6PD trait	23 (20 to 25)	20 (18 to 22)	-0.13 (-0.17 to -0.08)	153.26 (137.35 to 169.69)	116.25 (104.19 to 128.74)	-1.13 (-1.27 to -0.98)
New Zealand	Inherited anemias	9 (8 to 11)	7 (6 to 8)	-0.28 (-0.32 to -0.22)	277.74 (242.26 to 322.23)	152.61 (137.95 to 170.27)	-2.18 (-2.87 to -1.49)
New Zealand	Thalassemias	0 (0 to 0)	0 (0 to 0)	-0.03 (-0.12 to 0.07)	1.17 (0.8 to 1.8)	0.86 (0.59 to 1.31)	-0.89 (-1.03 to -0.76)
New Zealand	Thalassemias trait	1 (1 to 1)	1 (1 to 1)	0.07 (0 to 0.15)	26.39 (22.13 to 30.83)	21.52 (18.49 to 24.69)	-0.59 (-0.72 to -0.46)
New Zealand	Sickle cell disorders	0 (0 to 1)	0 (0 to 0)	-0.74 (-0.78 to -0.67)	14.61 (9.56 to 22.07)	2.93 (2.07 to 4.19)	-6.97 (-9.25 to -4.63)
New Zealand	Sickle cell trait	7 (5 to 8)	4 (3 to 5)	-0.39 (-0.44 to -0.34)	190.97 (160.2 to 227.76)	87.86 (75.12 to 104.17)	-3.29 (-4.31 to -2.26)

Regions	Causes	Cases in 1990 No.×103 (95% UI)	Cases in 2019 No.×103 (95% UI)	Change in absolute number (%)	Rates in 1990 per 100000 (95% UI)	Rates in 2019 per 100000 (95% UI)	EAPC from 1990 and 2019 (95%CI)
New Zealand	G6PD deficiency	0 (0 to 0)	0 (0 to 0)	0.13 (0.09 to 0.16)	2.57 (2.44 to 2.72)	2.2 (2.08 to 2.33)	-0.41 (-0.58 to -0.24)
New Zealand	G6PD trait	1 (1 to 2)	2 (2 to 2)	0.17 (0.12 to 0.21)	42.02 (40.03 to 43.91)	37.23 (35.72 to 38.6)	-0.21 (-0.42 to -0.01)
Nicaragua	Inherited anemias	26 (23 to 29)	20 (18 to 23)	-0.21 (-0.23 to -0.18)	659.48 (579.1 to 740.09)	312.48 (274.91 to 348.66)	-2.28 (-2.45 to -2.12)
Nicaragua	Thalassemias	0 (0 to 0)	0 (0 to 0)	0.56 (-0.09 to 2.35)	0.02 (0.01 to 0.03)	0.02 (0.01 to 0.02)	0.02 (-0.14 to 0.19)
Nicaragua	Thalassemias trait	0 (0 to 1)	0 (0 to 1)	0.14 (-0.09 to 0.57)	10.03 (7.17 to 13.56)	6.82 (5.09 to 8.65)	-1.06 (-1.16 to -0.95)
Nicaragua	Sickle cell disorders	0 (0 to 0)	0 (0 to 0)	-0.05 (-0.16 to 0.06)	0.12 (0.06 to 0.19)	0.07 (0.03 to 0.11)	-1.71 (-1.92 to -1.5)
Nicaragua	Sickle cell trait	0 (0 to 0)	0 (0 to 0)	-0.17 (-0.49 to -0.09)	4.26 (1.69 to 7.17)	2.12 (0.9 to 3.41)	-1.92 (-2.16 to -1.67)
Nicaragua	G6PD deficiency	5 (4 to 6)	3 (3 to 4)	-0.32 (-0.35 to -0.28)	132.08 (101.62 to 166.28)	53.75 (41.28 to 66.53)	-2.77 (-3.01 to -2.52)
Nicaragua	G6PD trait	20 (18 to 22)	16 (15 to 18)	-0.18 (-0.21 to -0.16)	512.97 (461.36 to 559.22)	249.71 (224.4 to 272.55)	-2.2 (-2.36 to -2.04)
Niger	Inherited anemias	195 (180 to 212)	494 (453 to 536)	1.53 (1.47 to 1.59)	2436.43 (2245.05 to 2644.02)	2119.65 (1945.98 to 2302.29)	-0.66 (-0.76 to -0.56)
Niger	Thalassemias	0 (0 to 0)	0 (0 to 0)	1.87 (1.59 to 2.19)	1.32 (1 to 1.75)	1.31 (1.01 to 1.68)	-0.21 (-0.29 to -0.12)
Niger	Thalassemias trait	15 (13 to 17)	41 (36 to 46)	1.76 (1.63 to 1.89)	184.91 (162.39 to 211.8)	175.51 (154.79 to 199.03)	-0.36 (-0.45 to -0.26)
Niger	Sickle cell disorders	7 (6 to 8)	13 (11 to 16)	0.94 (0.73 to 1.14)	85.4 (71.8 to 100.28)	56.92 (46.71 to 68.78)	-1.88 (-2.11 to -1.64)
Niger	Sickle cell trait	71 (65 to 79)	178 (161 to 200)	1.5 (1.36 to 1.65)	886.22 (807.06 to 979.78)	762.55 (689.58 to 859.85)	-0.71 (-0.81 to -0.61)

Regions	Causes	Cases in 1990 No.×103 (95% UI)	Cases in 2019 No.×103 (95% UI)	Change in absolute number (%)	Rates in 1990 per 100000 (95% UI)	Rates in 2019 per 100000 (95% UI)	EAPC from 1990 and 2019 (95%CI)
Niger	G6PD deficiency	33 (25 to 42)	82 (63 to 104)	1.48 (1.47 to 1.5)	411.83 (317.54 to 520.67)	352.2 (272.47 to 445.31)	-0.64 (-0.75 to -0.54)
Niger	G6PD trait	70 (63 to 76)	180 (163 to 195)	1.58 (1.56 to 1.61)	866.75 (785.2 to 942.01)	771.17 (698.11 to 836.5)	-0.58 (-0.67 to -0.48)
Nigeria	**Inherited anemias**	2657 (2472 to 2863)	4519 (4182 to 4891)	0.7 (0.68 to 0.72)	2945.58 (2740.66 to 3174.94)	2103.52 (1946.62 to 2276.91)	-1.04 (-1.18 to -0.91)
Nigeria	Thalassemias	0 (0 to 0)	0 (0 to 1)	2.13 (1.69 to 2.51)	0.16 (0.11 to 0.23)	0.22 (0.14 to 0.31)	0.69 (0.51 to 0.88)
Nigeria	Thalassemias trait	50 (43 to 60)	107 (90 to 126)	1.13 (1.04 to 1.22)	55.92 (47.19 to 66.76)	50.04 (42.08 to 58.85)	-0.52 (-0.67 to -0.37)
Nigeria	Sickle cell disorders	128 (94 to 171)	213 (153 to 289)	0.66 (0.58 to 0.74)	141.93 (104.68 to 189.61)	98.98 (71.04 to 134.42)	-1.4 (-1.48 to -1.31)
Nigeria	Sickle cell trait	1005 (858 to 1167)	1798 (1525 to 2090)	0.79 (0.76 to 0.82)	1114.7 (951.59 to 1293.65)	836.95 (709.77 to 972.78)	-1.06 (-1.17 to -0.95)
Nigeria	G6PD deficiency	645 (618 to 670)	1106 (1057 to 1149)	0.71 (0.67 to 0.74)	715.49 (684.99 to 742.83)	514.72 (492.11 to 535.02)	-1.04 (-1.17 to -0.9)
Nigeria	G6PD trait	827 (815 to 838)	1295 (1273 to 1314)	0.56 (0.56 to 0.57)	917.37 (903.94 to 928.92)	602.62 (592.72 to 611.54)	-1.01 (-1.23 to -0.79)
Niue	**Inherited anemias**	0 (0 to 0)	0 (0 to 0)	-0.48 (-0.5 to -0.47)	485.7 (432.92 to 544.03)	348.88 (308.53 to 390.86)	-1.18 (-1.43 to -0.93)
Niue	Thalassemias	0 (0 to 0)	0 (0 to 0)	-0.59 (-0.64 to -0.54)	2.19 (1.21 to 3.52)	1.24 (0.68 to 1.95)	-0.71 (-1.79 to 0.37)
Niue	Thalassemias trait	0 (0 to 0)	0 (0 to 0)	-0.55 (-0.58 to -0.53)	72.66 (55.82 to 91.15)	45.14 (34.57 to 55.87)	-1.19 (-1.66 to -0.71)
Niue	Sickle cell disorders	0 (0 to 0)	0 (0 to 0)	-0.52 (-0.55 to -0.32)	0 (0 to 0)	0 (0 to 0)	-1.57 (-1.71 to -1.42)
Niue	Sickle cell trait	0 (0 to 0)	0 (0 to 0)	-0.53 (-0.54 to -0.36)	2.9 (1.27 to 4.69)	1.91 (0.82 to 3.03)	-1.57 (-1.7 to -1.44)

Regions	Causes	Cases in 1990 No.×103 (95% UI)	Cases in 2019 No.×103 (95% UI)	Change in absolute number (%)	Rates in 1990 per 100000 (95% UI)	Rates in 2019 per 100000 (95% UI)	EAPC from 1990 and 2019 (95%CI)
Niue	G6PD deficiency	0 (0 to 0)	0 (0 to 0)	−0.44 (−0.46 to −0.42)	100.12 (77.28 to 125.37)	78.07 (60.14 to 96.91)	−0.98 (−1.27 to −0.69)
Niue	G6PD trait	0 (0 to 0)	0 (0 to 0)	−0.48 (−0.5 to −0.47)	307.83 (276.98 to 338.44)	222.51 (200.82 to 242.59)	−1.26 (−1.48 to −1.04)
North Macedonia	**Inherited anemias**	5 (5 to 6)	3 (3 to 4)	−0.35 (−0.36 to −0.34)	258.91 (229.08 to 292.3)	158.01 (140.18 to 178.42)	−1.84 (−2.07 to −1.61)
North Macedonia	Thalassemias	0 (0 to 0)	0 (0 to 0)	−0.61 (−0.66 to −0.55)	1.58 (1.02 to 2.57)	0.58 (0.39 to 0.89)	−3.31 (−3.57 to −3.05)
North Macedonia	Thalassemias trait	1 (1 to 1)	0 (0 to 1)	−0.34 (−0.38 to −0.29)	34.45 (28.96 to 41.88)	21.35 (17.88 to 25.54)	−1.61 (−1.88 to −1.33)
North Macedonia	Sickle cell disorders	0 (0 to 0)	0 (0 to 0)	−0.29 (−0.36 to −0.21)	0.18 (0.13 to 0.23)	0.12 (0.09 to 0.16)	−1.51 (−1.75 to −1.26)
North Macedonia	Sickle cell trait	1 (1 to 1)	0 (0 to 1)	−0.33 (−0.36 to −0.3)	33.18 (28.67 to 38.32)	20.79 (17.91 to 24.01)	−1.61 (−1.85 to −1.36)
North Macedonia	G6PD deficiency	1 (1 to 2)	1 (1 to 1)	−0.32 (−0.32 to −0.31)	62.94 (48.69 to 78.79)	40.27 (31.22 to 50.43)	−2.41 (−2.73 to −2.09)
North Macedonia	G6PD trait	3 (2 to 3)	2 (1 to 2)	−0.37 (−0.38 to −0.35)	126.58 (112 to 142.07)	74.91 (66.28 to 84.09)	−1.7 (−1.96 to −1.45)
Northern Mariana Islands	**Inherited anemias**	0 (0 to 0)	0 (0 to 0)	−0.56 (−0.56 to −0.55)	580.54 (515.32 to 649.09)	274.48 (243.52 to 306.72)	−3.44 (−4.02 to −2.84)
Northern Mariana Islands	Thalassemias	0 (0 to 0)	0 (0 to 0)	−0.65 (−0.7 to −0.61)	2.44 (1.29 to 3.86)	0.91 (0.48 to 1.43)	−3.05 (−3.89 to −2.21)
Northern Mariana Islands	Thalassemias trait	0 (0 to 0)	0 (0 to 0)	−0.62 (−0.64 to −0.6)	83.75 (63.64 to 104.08)	34.32 (25.88 to 42.58)	−3.47 (−4.02 to −2.9)
Northern Mariana Islands	Sickle cell disorders	0 (0 to 0)	0 (0 to 0)	−0.6 (−0.61 to −0.5)	0 (0 to 0)	0 (0 to 0)	−3.81 (−4.44 to −3.17)
Northern Mariana Islands	Sickle cell trait	0 (0 to 0)	0 (0 to 0)	−0.6 (−0.61 to −0.54)	3.41 (1.49 to 5.48)	1.47 (0.64 to 2.39)	−3.83 (−4.47 to −3.19)

Regions	Causes	Cases in 1990 No.×103 (95% UI)	Cases in 2019 No.×103 (95% UI)	Change in absolute number (%)	Rates in 1990 per 100000 (95% UI)	Rates in 2019 per 100000 (95% UI)	EAPC from 1990 and 2019 (95%CI)
Northern Mariana Islands	G6PD deficiency	0 (0 to 0)	0 (0 to 0)	-0.52 (-0.54 to -0.5)	121.98 (93.91 to 151.59)	62.25 (47.86 to 77.75)	-3.26 (-3.87 to -2.64)
Northern Mariana Islands	G6PD trait	0 (0 to 0)	0 (0 to 0)	-0.55 (-0.56 to -0.55)	368.96 (331.93 to 405.48)	175.53 (158.47 to 191.09)	-3.5 (-4.12 to -2.88)
Norway	**Inherited anemias**	11 (11 to 12)	11 (11 to 11)	-0.04 (-0.06 to -0.02)	269.51 (258.95 to 281.55)	206.11 (197.93 to 214.91)	-0.89 (-1.03 to -0.76)
Norway	Thalassemias	0 (0 to 0)	0 (0 to 0)	-0.12 (-0.17 to -0.04)	0.65 (0.47 to 0.89)	0.46 (0.34 to 0.6)	-1.45 (-1.7 to -1.2)
Norway	Thalassemias trait	2 (2 to 2)	2 (2 to 2)	-0.08 (-0.11 to -0.04)	47.61 (40.96 to 55.2)	34.87 (30.25 to 39.8)	-1.15 (-1.26 to -1.03)
Norway	Sickle cell disorders	0 (0 to 0)	0 (0 to 0)	0.93 (0.4 to 1.45)	0.14 (0.09 to 0.22)	0.22 (0.13 to 0.34)	1.59 (0.62 to 2.56)
Norway	Sickle cell trait	1 (1 to 1)	1 (1 to 2)	0.37 (0.16 to 0.55)	22.79 (17.13 to 29.63)	24.75 (19.25 to 31.37)	0.35 (-0.16 to 0.86)
Norway	G6PD deficiency	2 (2 to 2)	2 (1 to 2)	-0.03 (-0.04 to -0.02)	37.49 (35.73 to 39.01)	28.97 (27.69 to 30.16)	-0.88 (-0.97 to -0.79)
Norway	G6PD trait	7 (7 to 7)	6 (6 to 6)	-0.08 (-0.09 to -0.08)	160.83 (157.65 to 163.82)	116.86 (114.53 to 118.97)	-1.04 (-1.17 to -0.92)
Oman	**Inherited anemias**	37 (35 to 38)	42 (38 to 46)	0.14 (0.04 to 0.25)	1887.85 (1822.21 to 1955.85)	915.61 (829.49 to 1007.03)	-2.23 (-2.42 to -2.03)
Oman	Thalassemias	0 (0 to 0)	0 (0 to 0)	0.32 (0.17 to 0.51)	0.83 (0.6 to 1.17)	0.46 (0.34 to 0.62)	-1.74 (-1.96 to -1.53)
Oman	Thalassemias trait	2 (2 to 3)	3 (2 to 3)	0.26 (0.19 to 0.33)	115.19 (98.62 to 135.36)	61.41 (52.82 to 71.15)	-1.89 (-2.11 to -1.67)
Oman	Sickle cell disorders	0 (0 to 0)	0 (0 to 0)	0.22 (0.11 to 0.33)	17.63 (14.99 to 20.39)	9.09 (7.57 to 10.88)	-1.96 (-2.17 to -1.75)
Oman	Sickle cell trait	9 (8 to 9)	11 (10 to 11)	0.19 (0.14 to 0.24)	454.47 (421.83 to 486.44)	229.75 (211.35 to 249.81)	-2.04 (-2.25 to -1.82)

Regions	Causes	Cases in 1990 No.×103 (95% UI)	Cases in 2019 No.×103 (95% UI)	Change in absolute number (%)	Rates in 1990 per 100000 (95% UI)	Rates in 2019 per 100000 (95% UI)	EAPC from 1990 and 2019 (95%CI)
Oman	G6PD deficiency	11 (10 to 12)	12 (9 to 15)	0.04 (-0.19 to 0.27)	575.73 (537.56 to 613.56)	252.82 (195.44 to 317.05)	-2.67 (-2.82 to -2.52)
Oman	G6PD trait	14 (14 to 14)	17 (15 to 18)	0.18 (0.1 to 0.25)	724 (704.27 to 741.71)	362.08 (337.81 to 384.01)	-2.08 (-2.3 to -1.85)
Pakistan	Inherited anemias	815 (751 to 893)	1147 (1059 to 1257)	0.41 (0.38 to 0.43)	722.34 (665.95 to 791.59)	512 (472.63 to 561.06)	-1.33 (-1.46 to -1.2)
Pakistan	Thalassemias	5 (4 to 8)	7 (5 to 11)	0.37 (0.25 to 0.47)	4.83 (3.17 to 7.41)	3.32 (2.17 to 5.08)	-1.4 (-1.54 to -1.25)
Pakistan	Thalassemias trait	336 (278 to 411)	460 (380 to 563)	0.37 (0.32 to 0.42)	297.57 (246.73 to 364.2)	205.09 (169.61 to 251.3)	-1.39 (-1.54 to -1.24)
Pakistan	Sickle cell disorders	0 (0 to 0)	0 (0 to 0)	0.36 (0.24 to 0.56)	0.03 (0.02 to 0.04)	0.02 (0.01 to 0.03)	-1.42 (-1.6 to -1.24)
Pakistan	Sickle cell trait	21 (17 to 25)	28 (23 to 34)	0.36 (0.3 to 0.49)	18.41 (15.09 to 22.31)	12.65 (10.38 to 15.24)	-1.4 (-1.57 to -1.23)
Pakistan	G6PD deficiency	147 (140 to 154)	214 (205 to 224)	0.46 (0.43 to 0.49)	129.92 (123.98 to 136.11)	95.53 (91.29 to 99.86)	-1.52 (-1.79 to -1.26)
Pakistan	G6PD trait	306 (297 to 315)	438 (423 to 455)	0.43 (0.41 to 0.46)	271.58 (263.59 to 279.24)	195.39 (188.74 to 202.9)	-1.17 (-1.41 to -0.94)
Palau	Inherited anemias	0 (0 to 0)	0 (0 to 0)	-0.43 (-0.45 to -0.41)	506.62 (452.17 to 565.89)	246.48 (218.51 to 274.43)	-2.35 (-2.57 to -2.12)
Palau	Thalassemias	0 (0 to 0)	0 (0 to 0)	-0.54 (-0.6 to -0.48)	2.2 (1.22 to 3.39)	0.86 (0.47 to 1.32)	-1.9 (-3.03 to -0.75)
Palau	Thalassemias trait	0 (0 to 0)	0 (0 to 0)	-0.5 (-0.53 to -0.47)	73.89 (56.95 to 90.97)	31.3 (24.06 to 38.21)	-2.35 (-2.89 to -1.81)
Palau	Sickle cell disorders	0 (0 to 0)	0 (0 to 0)	-0.49 (-0.51 to -0.42)	0 (0 to 0)	0 (0 to 0)	-2.73 (-2.94 to -2.51)
Palau	Sickle cell trait	0 (0 to 0)	0 (0 to 0)	-0.49 (-0.49 to -0.44)	2.98 (1.3 to 4.85)	1.3 (0.57 to 2.11)	-2.76 (-2.98 to -2.55)

Regions	Causes	Cases in 1990 No.×103 (95% UI)	Cases in 2019 No.×103 (95% UI)	Change in absolute number (%)	Rates in 1990 per 100000 (95% UI)	Rates in 2019 per 100000 (95% UI)	EAPC from 1990 and 2019 (95%CI)
Palau	G6PD deficiency	0 (0 to 0)	0 (0 to 0)	-0.38 (-0.43 to -0.34)	106.66 (82.71 to 132.67)	56.32 (43.62 to 69.97)	-2.15 (-2.34 to -1.95)
Palau	G6PD trait	0 (0 to 0)	0 (0 to 0)	-0.43 (-0.45 to -0.41)	320.89 (289.18 to 351.54)	156.69 (141.9 to 170.1)	-2.43 (-2.59 to -2.26)
Palestine	Inherited anemias	32 (29 to 35)	43 (39 to 46)	0.33 (0.31 to 0.36)	1545.18 (1403.63 to 1689.88)	859.69 (782.61 to 937.47)	-2.09 (-2.26 to -1.93)
Palestine	Thalassemias	0 (0 to 0)	0 (0 to 0)	0.4 (0.22 to 0.58)	3.18 (2.09 to 4.82)	1.85 (1.25 to 2.76)	-1.91 (-2.07 to -1.75)
Palestine	Thalassemias trait	5 (4 to 6)	7 (6 to 9)	0.38 (0.29 to 0.46)	253 (209.84 to 306.53)	145.39 (121.06 to 174.99)	-1.95 (-2.12 to -1.78)
Palestine	Sickle cell disorders	0 (0 to 0)	0 (0 to 0)	0.4 (0.29 to 0.51)	4.82 (3.67 to 6.11)	2.81 (2.14 to 3.6)	-2.42 (-2.66 to -2.18)
Palestine	Sickle cell trait	6 (5 to 7)	8 (7 to 9)	0.35 (0.3 to 0.4)	287.33 (252.16 to 323.31)	161.89 (142.13 to 183.01)	-2.26 (-2.44 to -2.07)
Palestine	G6PD deficiency	5 (4 to 7)	7 (5 to 9)	0.31 (0.24 to 0.38)	260.43 (201.06 to 324.38)	142.51 (110.16 to 179.19)	-2.08 (-2.24 to -1.91)
Palestine	G6PD trait	15 (14 to 17)	20 (18 to 22)	0.32 (0.31 to 0.33)	736.42 (667.84 to 799.48)	405.24 (367.49 to 439.01)	-2.08 (-2.24 to -1.92)
Panama	Inherited anemias	11 (9 to 12)	12 (10 to 13)	0.13 (0.1 to 0.15)	441.15 (386.84 to 494.72)	285.61 (251.68 to 321.87)	-1.4 (-1.53 to -1.28)
Panama	Thalassemias	0 (0 to 0)	0 (0 to 0)	1.22 (0.29 to 3.74)	0.01 (0.01 to 0.02)	0.01 (0.01 to 0.02)	0.84 (0.61 to 1.06)
Panama	Thalassemias trait	0 (0 to 0)	0 (0 to 0)	0.62 (0.29 to 1.22)	6.53 (4.65 to 8.79)	6.06 (4.47 to 7.8)	-0.21 (-0.3 to -0.12)
Panama	Sickle cell disorders	0 (0 to 0)	0 (0 to 0)	0.31 (0.14 to 0.54)	0.06 (0.03 to 0.11)	0.05 (0.03 to 0.08)	-0.95 (-1.06 to -0.84)
Panama	Sickle cell trait	0 (0 to 0)	0 (0 to 0)	0.22 (-0.23 to 0.29)	2.68 (1.1 to 4.23)	1.87 (0.82 to 3.04)	-1.05 (-1.17 to -0.94)

Regions	Causes	Cases in 1990 No.×103 (95% UI)	Cases in 2019 No.×103 (95% UI)	Change in absolute number (%)	Rates in 1990 per 100000 (95% UI)	Rates in 2019 per 100000 (95% UI)	EAPC from 1990 and 2019 (95%CI)
Panama	G6PD deficiency	2 (2 to 3)	2 (2 to 3)	-0.03 (-0.07 to 0.02)	89.53 (69.2 to 112.95)	49.77 (38.26 to 62.55)	-1.89 (-2.14 to -1.64)
Panama	G6PD trait	8 (7 to 9)	9 (9 to 10)	0.16 (0.13 to 0.19)	342.33 (308.21 to 373.07)	227.84 (204.76 to 251.56)	-1.32 (-1.42 to -1.21)
Papua New Guinea	Inherited anemias	40 (37 to 43)	95 (86 to 105)	1.37 (1.27 to 1.49)	984.1 (909.67 to 1060.2)	967.75 (869.59 to 1068.84)	-0.18 (-0.34 to -0.01)
Papua New Guinea	Thalassemias	0 (0 to 0)	1 (0 to 1)	1.06 (0.87 to 1.27)	7.44 (4.99 to 10.24)	6.35 (4.31 to 8.9)	0.35 (-0.37 to 1.08)
Papua New Guinea	Thalassemias trait	12 (11 to 14)	27 (23 to 31)	1.18 (1.1 to 1.27)	302.19 (261.47 to 344.91)	273.17 (235.45 to 313.31)	-0.11 (-0.31 to 0.09)
Papua New Guinea	Sickle cell disorders	0 (0 to 0)	0 (0 to 0)	1.32 (1.21 to 2.4)	0 (0 to 0.01)	0 (0 to 0.01)	-0.06 (-0.16 to 0.05)
Papua New Guinea	Sickle cell trait	0 (0 to 0)	0 (0 to 1)	1.28 (1.21 to 2.17)	5.27 (2.28 to 8.76)	4.99 (2.09 to 8.64)	-0.14 (-0.22 to -0.06)
Papua New Guinea	G6PD deficiency	10 (9 to 11)	18 (14 to 23)	0.85 (0.48 to 1.23)	242.23 (224.09 to 260.24)	185.22 (143.19 to 231.7)	-0.94 (-1.19 to -0.68)
Papua New Guinea	G6PD trait	17 (16 to 19)	49 (44 to 54)	1.82 (1.74 to 1.91)	426.95 (382.22 to 472.04)	498.01 (450.43 to 545.96)	0.08 (-0.18 to 0.33)
Paraguay	Inherited anemias	23 (20 to 25)	23 (20 to 25)	0 (-0.02 to 0.04)	558.38 (497.58 to 621.15)	327.34 (292.2 to 364.94)	-1.71 (-1.81 to -1.62)
Paraguay	Thalassemias	0 (0 to 0)	0 (0 to 0)	0.36 (-0.07 to 1.08)	0.03 (0.02 to 0.04)	0.02 (0.01 to 0.03)	-0.68 (-0.79 to -0.57)
Paraguay	Thalassemias trait	1 (1 to 1)	1 (1 to 1)	0.1 (-0.06 to 0.29)	18.11 (15.42 to 21.31)	11.6 (9.8 to 13.44)	-1.38 (-1.43 to -1.32)
Paraguay	Sickle cell disorders	0 (0 to 0)	0 (0 to 0)	0.05 (-0.04 to 0.15)	0.6 (0.47 to 0.76)	0.37 (0.28 to 0.47)	-2 (-2.21 to -1.79)
Paraguay	Sickle cell trait	3 (3 to 4)	3 (3 to 4)	0.02 (-0.03 to 0.08)	75.78 (65.55 to 87.73)	45.28 (38.89 to 52.38)	-1.9 (-2.04 to -1.75)

Regions	Causes	Cases in 1990 No.×103 (95% UI)	Cases in 2019 No.×103 (95% UI)	Change in absolute number (%)	Rates in 1990 per 100000 (95% UI)	Rates in 2019 per 100000 (95% UI)	EAPC from 1990 and 2019 (95%CI)
Paraguay	G6PD deficiency	4 (3 to 5)	4 (3 to 5)	-0.01 (-0.04 to 0.03)	95.03 (72.85 to 119.42)	55.14 (42.41 to 69.12)	-1.69 (-1.79 to -1.6)
Paraguay	G6PD trait	15 (13 to 16)	15 (13 to 16)	0 (-0.03 to 0.03)	368.83 (329.86 to 407.56)	214.93 (192.94 to 237.63)	-1.7 (-1.79 to -1.6)
Peru	Inherited anemias	103 (93 to 114)	113 (99 to 127)	0.09 (0.05 to 0.13)	475.11 (426.74 to 525.42)	331.3 (292.57 to 373.64)	-1.54 (-1.64 to -1.44)
Peru	Thalassemias	0 (0 to 0)	0 (0 to 0)	0.35 (-0.04 to 1.1)	0.03 (0.02 to 0.04)	0.02 (0.02 to 0.03)	-0.45 (-0.59 to -0.3)
Peru	Thalassemias trait	4 (3 to 5)	4 (4 to 5)	0.07 (-0.07 to 0.24)	19.02 (16.01 to 22.4)	13 (10.87 to 15.19)	-1.25 (-1.29 to -1.21)
Peru	Sickle cell disorders	0 (0 to 0)	0 (0 to 0)	-0.02 (-0.12 to 0.07)	0.16 (0.11 to 0.22)	0.1 (0.07 to 0.14)	-1.41 (-1.48 to -1.34)
Peru	Sickle cell trait	9 (8 to 11)	9 (7 to 11)	-0.03 (-0.08 to 0.01)	42.58 (35.35 to 50.91)	26.44 (21.85 to 31.64)	-1.51 (-1.55 to -1.47)
Peru	G6PD deficiency	10 (8 to 13)	25 (19 to 31)	1.4 (1.23 to 1.55)	48.03 (37.48 to 60.01)	73.57 (57.18 to 92.44)	-0.85 (-1.54 to -0.16)
Peru	G6PD trait	79 (71 to 88)	74 (66 to 82)	-0.07 (-0.1 to -0.02)	365.29 (327.83 to 404.83)	218.17 (194.98 to 241.3)	-1.7 (-1.73 to -1.67)
Philippines	Inherited anemias	400 (377 to 430)	485 (455 to 526)	0.21 (0.2 to 0.23)	631.29 (595.33 to 679.73)	432.76 (406.12 to 468.75)	-1.14 (-1.24 to -1.04)
Philippines	Thalassemias	2 (2 to 4)	4 (3 to 5)	0.44 (0.39 to 0.49)	3.92 (2.79 to 5.58)	3.18 (2.27 to 4.51)	-0.5 (-0.64 to -0.37)
Philippines	Thalassemias trait	135 (114 to 163)	182 (154 to 219)	0.35 (0.33 to 0.37)	213.21 (179.59 to 256.83)	162.43 (137.09 to 195.4)	-0.82 (-0.89 to -0.75)
Philippines	Sickle cell disorders	0 (0 to 0)	0 (0 to 0)	0.22 (0.06 to 0.33)	0.03 (0.02 to 0.04)	0.02 (0.01 to 0.03)	-1.3 (-1.38 to -1.23)
Philippines	Sickle cell trait	11 (10 to 14)	14 (12 to 17)	0.25 (0.17 to 0.31)	18.14 (15.29 to 21.44)	12.79 (10.91 to 15.01)	-1.19 (-1.23 to -1.15)

Regions	Causes	Cases in 1990 No.×103 (95% UI)	Cases in 2019 No.×103 (95% UI)	Change in absolute number (%)	Rates in 1990 per 100000 (95% UI)	Rates in 2019 per 100000 (95% UI)	EAPC from 1990 and 2019 (95%CI)
Philippines	G6PD deficiency	50 (48 to 52)	62 (59 to 64)	0.24 (0.22 to 0.26)	78.71 (75.13 to 81.97)	55.11 (52.57 to 57.33)	-1.02 (-1.1 to -0.93)
Philippines	G6PD trait	201 (197 to 204)	223 (219 to 227)	0.11 (0.11 to 0.12)	317.29 (310.81 to 322.78)	199.24 (195.02 to 202.84)	-1.41 (-1.57 to -1.26)
Poland	Inherited anemias	76 (74 to 79)	53 (52 to 55)	-0.3 (-0.3 to -0.3)	199.93 (193.09 to 207.3)	138.87 (134.27 to 143.96)	-0.89 (-1.23 to -0.55)
Poland	Thalassemias	0 (0 to 1)	0 (0 to 0)	-0.58 (-0.6 to -0.55)	1.22 (0.81 to 1.92)	0.51 (0.35 to 0.77)	-2.4 (-2.76 to -2.03)
Poland	Thalassemias trait	10 (9 to 13)	7 (6 to 9)	-0.29 (-0.32 to -0.27)	26.73 (22.34 to 32.85)	18.73 (15.78 to 22.56)	-0.66 (-1.02 to -0.3)
Poland	Sickle cell disorders	0 (0 to 0)	0 (0 to 0)	-0.28 (-0.31 to -0.25)	0.13 (0.1 to 0.17)	0.09 (0.07 to 0.12)	-0.95 (-1.26 to -0.64)
Poland	Sickle cell trait	10 (8 to 11)	7 (6 to 8)	-0.3 (-0.31 to -0.29)	25.09 (21.71 to 28.7)	17.45 (15.02 to 20.07)	-0.83 (-1.16 to -0.51)
Poland	G6PD deficiency	18 (17 to 19)	13 (13 to 14)	-0.26 (-0.27 to -0.26)	47.22 (45.1 to 49.25)	34.61 (33.01 to 36.06)	-1.44 (-1.86 to -1.01)
Poland	G6PD trait	38 (37 to 39)	26 (25 to 27)	-0.32 (-0.32 to -0.32)	99.53 (97.3 to 101.73)	67.47 (65.97 to 68.97)	-0.72 (-1.08 to -0.36)
Portugal	Inherited anemias	24 (22 to 27)	16 (15 to 18)	-0.33 (-0.35 to -0.3)	240.16 (218.39 to 263.32)	153.59 (139.11 to 168.36)	-1.65 (-1.82 to -1.47)
Portugal	Thalassemias	0 (0 to 0)	0 (0 to 0)	-0.34 (-0.45 to -0.24)	0.13 (0.08 to 0.18)	0.08 (0.05 to 0.12)	-2.72 (-3.85 to -1.58)
Portugal	Thalassemias trait	1 (1 to 1)	1 (1 to 1)	-0.29 (-0.35 to -0.22)	11.2 (9.44 to 13.01)	7.56 (6.31 to 9.06)	-1.78 (-2.29 to -1.27)
Portugal	Sickle cell disorders	0 (0 to 0)	0 (0 to 0)	-0.44 (-0.53 to -0.34)	1.61 (1.13 to 2.28)	0.86 (0.61 to 1.2)	-2.43 (-2.58 to -2.29)
Portugal	Sickle cell trait	8 (7 to 10)	5 (4 to 6)	-0.38 (-0.43 to -0.33)	80.23 (67.21 to 95.41)	47.32 (39.59 to 56.17)	-1.97 (-2.07 to -1.86)

Regions	Causes	Cases in 1990 No.×103 (95% UI)	Cases in 2019 No.×103 (95% UI)	Change in absolute number (%)	Rates in 1990 per 100000 (95% UI)	Rates in 2019 per 100000 (95% UI)	EAPC from 1990 and 2019 (95%CI)
Portugal	G6PD deficiency	1 (1 to 2)	1 (1 to 2)	-0.07 (-0.15 to 0)	13.95 (11.46 to 16.9)	12.39 (9.52 to 15.74)	-0.84 (-1.11 to -0.57)
Portugal	G6PD trait	13 (12 to 15)	9 (8 to 10)	-0.33 (-0.35 to -0.3)	133.04 (119.6 to 147)	85.38 (76.45 to 94.51)	-1.55 (-1.75 to -1.35)
Puerto Rico	**Inherited anemias**	16 (14 to 18)	6 (6 to 7)	-0.6 (-0.62 to -0.59)	444.38 (390.44 to 503.75)	180.24 (158.19 to 204.37)	-3.19 (-3.41 to -2.97)
Puerto Rico	Thalassemias	0 (0 to 0)	0 (0 to 0)	-0.37 (-0.61 to 0.36)	0.01 (0 to 0.01)	0 (0 to 0.01)	-1.75 (-2.2 to -1.31)
Puerto Rico	Thalassemias trait	0 (0 to 0)	0 (0 to 0)	-0.54 (-0.61 to -0.4)	5.99 (4.58 to 7.46)	2.81 (2.19 to 3.43)	-2.76 (-3.06 to -2.45)
Puerto Rico	Sickle cell disorders	0 (0 to 0)	0 (0 to 0)	-0.56 (-0.61 to -0.5)	2.77 (2.05 to 3.62)	1.25 (0.91 to 1.68)	-2.89 (-3 to -2.78)
Puerto Rico	Sickle cell trait	4 (4 to 5)	2 (2 to 2)	-0.59 (-0.61 to -0.56)	124.26 (105.23 to 144.52)	52.2 (43.53 to 61.35)	-3.09 (-3.25 to -2.92)
Puerto Rico	G6PD deficiency	5 (4 to 6)	2 (2 to 2)	-0.61 (-0.64 to -0.59)	141.96 (109.7 to 177.5)	56.52 (43.45 to 69.89)	-3.24 (-3.5 to -2.99)
Puerto Rico	G6PD trait	6 (5 to 7)	2 (2 to 3)	-0.61 (-0.62 to -0.6)	169.4 (150.71 to 189.03)	67.46 (60.02 to 75.22)	-3.24 (-3.48 to -3)
Qatar	**Inherited anemias**	3 (3 to 4)	9 (8 to 9)	1.58 (1.52 to 1.64)	757.46 (688.61 to 830.24)	303.1 (275.42 to 331.44)	-3.17 (-3.36 to -2.97)
Qatar	Thalassemias	0 (0 to 0)	0 (0 to 0)	1.78 (1.45 to 2.16)	0.62 (0.42 to 0.89)	0.27 (0.19 to 0.37)	-3 (-3.19 to -2.8)
Qatar	Thalassemias trait	0 (0 to 0)	1 (1 to 1)	1.69 (1.54 to 1.85)	82.07 (68.85 to 97.53)	34.27 (28.88 to 40.36)	-3.07 (-3.27 to -2.87)
Qatar	Sickle cell disorders	0 (0 to 0)	0 (0 to 0)	1.55 (1.36 to 1.73)	2.72 (2.1 to 3.47)	1.08 (0.83 to 1.37)	-3.15 (-3.37 to -2.93)
Qatar	Sickle cell trait	1 (1 to 1)	2 (2 to 2)	1.54 (1.44 to 1.63)	154.23 (136.04 to 173.99)	60.84 (53.62 to 68.66)	-3.19 (-3.4 to -2.98)

Regions	Causes	Cases in 1990 No.×103 (95% UI)	Cases in 2019 No.×103 (95% UI)	Change in absolute number (%)	Rates in 1990 per 100000 (95% UI)	Rates in 2019 per 100000 (95% UI)	EAPC from 1990 and 2019 (95%CI)
Qatar	G6PD deficiency	1 (0 to 1)	2 (1 to 2)	1.55 (1.43 to 1.68)	136.83 (105.46 to 169.9)	54.21 (41.92 to 68.26)	-3.19 (-3.39 to -3)
Qatar	G6PD trait	2 (2 to 2)	4 (4 to 5)	1.57 (1.51 to 1.66)	380.99 (345.93 to 413.41)	152.44 (138.89 to 164.74)	-3.17 (-3.36 to -2.97)
Republic of Korea	**Inherited anemias**	52 (46 to 58)	30 (26 to 33)	-0.43 (-0.45 to -0.4)	116.68 (103.36 to 131.16)	55.53 (49.26 to 62.14)	-2.96 (-3.06 to -2.85)
Republic of Korea	Thalassemias	0 (0 to 0)	0 (0 to 0)	-0.5 (-0.56 to -0.41)	0.69 (0.46 to 1.07)	0.29 (0.2 to 0.43)	-3.12 (-3.27 to -2.97)
Republic of Korea	Thalassemias trait	3 (3 to 4)	3 (3 to 4)	0.07 (-0.05 to 0.19)	7.18 (5.66 to 8.86)	6.36 (4.85 to 7.78)	-0.67 (-0.96 to -0.39)
Republic of Korea	Sickle cell disorders	0 (0 to 0)	0 (0 to 0)	-0.42 (-0.49 to -0.33)	0.02 (0.01 to 0.02)	0.01 (0.01 to 0.01)	-2.44 (-2.7 to -2.19)
Republic of Korea	Sickle cell trait	3 (3 to 4)	2 (2 to 2)	-0.42 (-0.46 to -0.33)	7.44 (6.02 to 9.14)	3.6 (2.88 to 4.35)	-2.51 (-2.72 to -2.3)
Republic of Korea	G6PD deficiency	3 (3 to 4)	2 (1 to 2)	-0.52 (-0.56 to -0.49)	7.82 (5.93 to 10.02)	3.1 (2.33 to 3.99)	-4.77 (-5.23 to -4.31)
Republic of Korea	G6PD trait	41 (36 to 47)	23 (20 to 26)	-0.46 (-0.48 to -0.43)	93.53 (81.89 to 105.49)	42.17 (36.67 to 47.82)	-3.11 (-3.21 to -3.01)
Republic of Moldova	**Inherited anemias**	10 (9 to 11)	4 (4 to 5)	-0.57 (-0.58 to -0.57)	223.49 (198.54 to 250.51)	114.65 (102.07 to 128.35)	-1.57 (-1.9 to -1.25)
Republic of Moldova	Thalassemias	0 (0 to 0)	0 (0 to 0)	-0.71 (-0.74 to -0.67)	1.06 (0.73 to 1.55)	0.37 (0.27 to 0.53)	-2.81 (-3.14 to -2.47)
Republic of Moldova	Thalassemias trait	2 (2 to 2)	1 (1 to 1)	-0.56 (-0.59 to -0.53)	45.71 (38 to 55.49)	24.24 (20.21 to 29)	-1.46 (-1.77 to -1.14)
Republic of Moldova	Sickle cell disorders	0 (0 to 0)	0 (0 to 0)	-0.56 (-0.62 to -0.47)	0.01 (0.01 to 0.02)	0.01 (0 to 0.01)	-1.5 (-1.79 to -1.21)
Republic of Moldova	Sickle cell trait	0 (0 to 0)	0 (0 to 0)	-0.57 (-0.6 to -0.53)	8.59 (6.85 to 10.52)	4.42 (3.56 to 5.4)	-1.54 (-1.85 to -1.22)

Regions	Causes	Cases in 1990 No.×103 (95% UI)	Cases in 2019 No.×103 (95% UI)	Change in absolute number (%)	Rates in 1990 per 100000 (95% UI)	Rates in 2019 per 100000 (95% UI)	EAPC from 1990 and 2019 (95%CI)
Republic of Moldova	G6PD deficiency	2 (1 to 2)	1 (1 to 1)	-0.55 (-0.55 to -0.55)	34.85 (26.75 to 43.36)	18.87 (14.49 to 23.48)	-1.7 (-2.02 to -1.37)
Republic of Moldova	G6PD trait	6 (5 to 7)	2 (2 to 3)	-0.58 (-0.58 to -0.58)	133.28 (118.43 to 149.2)	66.75 (59.31 to 74.72)	-1.58 (-1.91 to -1.24)
Romania	Inherited anemias	35 (31 to 40)	25 (22 to 28)	-0.3 (-0.32 to -0.29)	151.06 (134.31 to 169.07)	128.03 (112.78 to 144.34)	-0.54 (-0.74 to -0.33)
Romania	Thalassemias	0 (0 to 0)	0 (0 to 0)	-0.64 (-0.68 to -0.59)	1.17 (0.74 to 1.87)	0.51 (0.33 to 0.8)	-2.3 (-2.48 to -2.12)
Romania	Thalassemias trait	3 (2 to 3)	2 (2 to 2)	-0.34 (-0.4 to -0.27)	12.11 (10.26 to 14.51)	9.67 (8.24 to 11.28)	-0.31 (-0.5 to -0.12)
Romania	Sickle cell disorders	0 (0 to 0)	0 (0 to 0)	-0.29 (-0.36 to -0.21)	0.13 (0.1 to 0.17)	0.11 (0.08 to 0.15)	-0.19 (-0.37 to -0.01)
Romania	Sickle cell trait	5 (5 to 6)	4 (3 to 4)	-0.35 (-0.38 to -0.31)	23.11 (19.85 to 26.67)	18.23 (15.56 to 21.15)	-0.37 (-0.54 to -0.2)
Romania	G6PD deficiency	6 (4 to 7)	7 (5 to 8)	0.14 (0.06 to 0.21)	24.86 (19.07 to 30.92)	34.38 (26.38 to 42.93)	-1.09 (-1.87 to -0.29)
Romania	G6PD trait	21 (19 to 23)	13 (11 to 14)	-0.4 (-0.4 to -0.4)	89.68 (79.11 to 100.41)	65.13 (57.46 to 72.93)	-0.52 (-0.71 to -0.33)
Russian Federation	Inherited anemias	241 (231 to 253)	215 (206 to 225)	-0.11 (-0.11 to -0.1)	159.55 (153.01 to 167.71)	146.34 (140.38 to 153.57)	1.3 (0.78 to 1.82)
Russian Federation	Thalassemias	1 (1 to 2)	1 (1 to 1)	-0.41 (-0.45 to -0.35)	0.81 (0.56 to 1.18)	0.49 (0.36 to 0.7)	-0.07 (-0.59 to 0.45)
Russian Federation	Thalassemias trait	50 (41 to 61)	45 (38 to 55)	-0.09 (-0.11 to -0.06)	32.96 (27.43 to 40.3)	30.95 (25.92 to 37.62)	1.38 (0.87 to 1.89)
Russian Federation	Sickle cell disorders	0 (0 to 0)	0 (0 to 0)	-0.09 (-0.13 to -0.01)	0.01 (0.01 to 0.01)	0.01 (0.01 to 0.01)	1.33 (0.85 to 1.82)
Russian Federation	Sickle cell trait	9 (8 to 11)	8 (7 to 10)	-0.11 (-0.13 to -0.07)	6.2 (5.01 to 7.54)	5.69 (4.66 to 6.93)	1.32 (0.82 to 1.83)

Regions	Causes	Cases in 1990 No.×103 (95% UI)	Cases in 2019 No.×103 (95% UI)	Change in absolute number (%)	Rates in 1990 per 100000 (95% UI)	Rates in 2019 per 100000 (95% UI)	EAPC from 1990 and 2019 (95%CI)
Russian Federation	G6PD deficiency	37 (35 to 38)	35 (33 to 36)	-0.06 (-0.06 to -0.06)	24.32 (23.21 to 25.35)	23.52 (22.45 to 24.52)	1.18 (0.68 to 1.68)
Russian Federation	G6PD trait	144 (141 to 147)	126 (123 to 128)	-0.13 (-0.13 to -0.13)	95.26 (93.1 to 97.25)	85.67 (83.75 to 87.47)	1.31 (0.78 to 1.84)
Rwanda	Inherited anemias	70 (62 to 78)	70 (63 to 79)	0.01 (-0.02 to 0.04)	972.78 (861.79 to 1092.44)	554.94 (495.17 to 622.33)	-1.95 (-2.13 to -1.77)
Rwanda	Thalassemias	0 (0 to 0)	0 (0 to 0)	1.52 (1.21 to 2.1)	0.09 (0.05 to 0.13)	0.12 (0.07 to 0.19)	0.8 (0.57 to 1.03)
Rwanda	Thalassemias trait	2 (1 to 2)	3 (2 to 3)	0.62 (0.51 to 0.84)	21.65 (17.04 to 26.91)	19.84 (15.25 to 24.83)	-0.56 (-0.72 to -0.4)
Rwanda	Sickle cell disorders	0 (0 to 0)	0 (0 to 0)	0.03 (-0.07 to 0.14)	1.16 (0.86 to 1.54)	0.68 (0.5 to 0.91)	-1.75 (-2.21 to -1.3)
Rwanda	Sickle cell trait	10 (9 to 12)	11 (9 to 12)	0.07 (0.01 to 0.12)	139.12 (120.14 to 160.92)	83.98 (72.1 to 97.77)	-1.73 (-2.01 to -1.44)
Rwanda	G6PD deficiency	16 (13 to 20)	13 (10 to 17)	-0.17 (-0.23 to -0.1)	225.8 (174.42 to 283.79)	105.61 (81.84 to 132.01)	-2.47 (-2.76 to -2.18)
Rwanda	G6PD trait	42 (38 to 46)	44 (39 to 48)	0.04 (0.01 to 0.08)	584.96 (525.49 to 638.97)	344.7 (309.55 to 381)	-1.89 (-2.02 to -1.76)
Saint Kitts and Nevis	Inherited anemias	0 (0 to 0)	0 (0 to 0)	-0.2 (-0.23 to -0.17)	570.75 (503.15 to 646.04)	317.02 (278.36 to 358.03)	-2.03 (-2.06 to -2.01)
Saint Kitts and Nevis	Thalassemias	0 (0 to 0)	0 (0 to 0)	0.28 (-0.23 to 1.5)	0.01 (0.01 to 0.02)	0.01 (0.01 to 0.01)	-0.5 (-0.79 to -0.2)
Saint Kitts and Nevis	Thalassemias trait	0 (0 to 0)	0 (0 to 0)	-0.08 (-0.23 to 0.15)	8.05 (6.24 to 10.17)	5.14 (4.02 to 6.25)	-1.58 (-1.71 to -1.45)
Saint Kitts and Nevis	Sickle cell disorders	0 (0 to 0)	0 (0 to 0)	-0.1 (-0.2 to 0.01)	5.82 (4.23 to 7.79)	3.65 (2.59 to 4.94)	-1.71 (-1.87 to -1.56)
Saint Kitts and Nevis	Sickle cell trait	0 (0 to 0)	0 (0 to 0)	-0.17 (-0.22 to -0.12)	182.98 (152.32 to 215.71)	105.89 (87.87 to 126.49)	-1.91 (-1.97 to -1.86)

Regions	Causes	Cases in 1990 No.×103 (95% UI)	Cases in 2019 No.×103 (95% UI)	Change in absolute number (%)	Rates in 1990 per 100000 (95% UI)	Rates in 2019 per 100000 (95% UI)	EAPC from 1990 and 2019 (95%CI)
Saint Kitts and Nevis	G6PD deficiency	0 (0 to 0)	0 (0 to 0)	-0.22 (-0.29 to -0.15)	170.17 (131.37 to 211.35)	91.74 (70.87 to 113.56)	-2.12 (-2.18 to -2.06)
Saint Kitts and Nevis	G6PD trait	0 (0 to 0)	0 (0 to 0)	-0.22 (-0.25 to -0.18)	203.71 (182.03 to 224.53)	110.59 (98.5 to 123.18)	-2.1 (-2.15 to -2.06)
Saint Lucia	**Inherited anemias**	1 (1 to 1)	0 (0 to 1)	-0.53 (-0.55 to -0.52)	757.24 (667.25 to 856.73)	279 (245.86 to 314.91)	-3.58 (-3.72 to -3.44)
Saint Lucia	Thalassemias	0 (0 to 0)	0 (0 to 0)	-0.23 (-0.53 to 0.54)	0.01 (0.01 to 0.02)	0.01 (0 to 0.01)	-1.95 (-2.13 to -1.77)
Saint Lucia	Thalassemias trait	0 (0 to 0)	0 (0 to 0)	-0.45 (-0.53 to -0.29)	9.93 (7.53 to 12.75)	4.3 (3.34 to 5.21)	-2.99 (-3.04 to -2.93)
Saint Lucia	Sickle cell disorders	0 (0 to 0)	0 (0 to 0)	-0.51 (-0.57 to -0.44)	9.1 (6.59 to 12.23)	3.51 (2.5 to 4.66)	-3.77 (-4.14 to -3.39)
Saint Lucia	Sickle cell trait	0 (0 to 0)	0 (0 to 0)	-0.53 (-0.56 to -0.5)	253.67 (210.32 to 298.6)	93.25 (77.6 to 109.73)	-3.75 (-4 to -3.51)
Saint Lucia	G6PD deficiency	0 (0 to 0)	0 (0 to 0)	-0.54 (-0.56 to -0.5)	220.73 (170.43 to 278.26)	80.71 (62.34 to 99.95)	-3.51 (-3.6 to -3.43)
Saint Lucia	G6PD trait	0 (0 to 0)	0 (0 to 0)	-0.53 (-0.56 to -0.51)	263.8 (234.77 to 294.71)	97.22 (86.71 to 106.93)	-3.49 (-3.59 to -3.4)
Saint Vincent and the Grenadines	**Inherited anemias**	1 (1 to 1)	0 (0 to 0)	-0.44 (-0.46 to -0.42)	679.95 (603.66 to 768.78)	369.83 (325.38 to 417.44)	-2.09 (-2.16 to -2.02)
Saint Vincent and the Grenadines	Thalassemias	0 (0 to 0)	0 (0 to 0)	-0.04 (-0.43 to 0.84)	0.01 (0.01 to 0.02)	0.01 (0.01 to 0.02)	-0.44 (-0.68 to -0.19)
Saint Vincent and the Grenadines	Thalassemias trait	0 (0 to 0)	0 (0 to 0)	-0.32 (-0.43 to -0.15)	8.92 (6.94 to 11.23)	5.89 (4.59 to 7.15)	-1.52 (-1.6 to -1.45)
Saint Vincent and the Grenadines	Sickle cell disorders	0 (0 to 0)	0 (0 to 0)	-0.46 (-0.52 to -0.39)	8.93 (6.47 to 12.2)	4.7 (3.34 to 6.36)	-2.12 (-2.43 to -1.81)

Regions	Causes	Cases in 1990 No.×103 (95% UI)	Cases in 2019 No.×103 (95% UI)	Change in absolute number (%)	Rates in 1990 per 100000 (95% UI)	Rates in 2019 per 100000 (95% UI)	EAPC from 1990 and 2019 (95%CI)
Saint Vincent and the Grenadines	Sickle cell trait	0 (0 to 0)	0 (0 to 0)	-0.47 (-0.5 to -0.44)	239.89 (200.09 to 287.38)	124.5 (103.26 to 148.03)	-2.17 (-2.35 to -1.99)
Saint Vincent and the Grenadines	G6PD deficiency	0 (0 to 0)	0 (0 to 0)	-0.43 (-0.47 to -0.4)	193.14 (149.67 to 241.61)	106.56 (82.39 to 133.6)	-2.08 (-2.11 to -2.05)
Saint Vincent and the Grenadines	G6PD trait	0 (0 to 0)	0 (0 to 0)	-0.42 (-0.45 to -0.4)	229.07 (204.62 to 254.14)	128.17 (114.29 to 142.09)	-2.04 (-2.07 to -2.01)
Samoa	**Inherited anemias**	1 (1 to 1)	1 (1 to 1)	0.26 (0.24 to 0.29)	398.49 (354.89 to 445.34)	389.96 (346.72 to 436.58)	0.17 (-0.15 to 0.49)
Samoa	Thalassemias	0 (0 to 0)	0 (0 to 0)	0.02 (-0.09 to 0.17)	1.98 (1.08 to 3.07)	1.57 (0.87 to 2.41)	0.56 (-0.48 to 1.61)
Samoa	Thalassemias trait	0 (0 to 0)	0 (0 to 0)	0.1 (0.04 to 0.17)	62.37 (48.02 to 76.16)	53.21 (41.03 to 64.8)	0.12 (-0.36 to 0.61)
Samoa	Sickle cell disorders	0 (0 to 0)	0 (0 to 0)	0.23 (0.13 to 1.78)	0 (0 to 0)	0 (0 to 0)	0.01 (-0.32 to 0.34)
Samoa	Sickle cell trait	0 (0 to 0)	0 (0 to 0)	0.21 (0.13 to 1.27)	2.37 (1.03 to 3.83)	2.23 (0.96 to 3.43)	-0.04 (-0.35 to 0.26)
Samoa	G6PD deficiency	0 (0 to 0)	0 (0 to 0)	0.37 (0.31 to 0.46)	82.67 (63.91 to 102.53)	87.89 (68.1 to 109.55)	0.38 (0.01 to 0.75)
Samoa	G6PD trait	0 (0 to 0)	1 (0 to 1)	0.27 (0.25 to 0.3)	249.1 (223.93 to 272.51)	245.06 (222.19 to 266.49)	0.09 (-0.2 to 0.39)
San Marino	**Inherited anemias**	0 (0 to 0)	0 (0 to 0)	0.17 (0.14 to 0.2)	200.39 (179.41 to 222.43)	167.17 (149.56 to 186.1)	-0.85 (-1.04 to -0.67)
San Marino	Thalassemias	0 (0 to 0)	0 (0 to 0)	0.11 (-0.06 to 0.27)	0.14 (0.1 to 0.2)	0.11 (0.08 to 0.15)	-1.84 (-2.74 to -0.92)
San Marino	Thalassemias trait	0 (0 to 0)	0 (0 to 0)	0.2 (0.13 to 0.28)	17.71 (15.35 to 20.34)	15.16 (13.04 to 17.41)	-0.95 (-1.28 to -0.61)

Regions	Causes	Cases in 1990 No.×103 (95% UI)	Cases in 2019 No.×103 (95% UI)	Change in absolute number (%)	Rates in 1990 per 100000 (95% UI)	Rates in 2019 per 100000 (95% UI)	EAPC from 1990 and 2019 (95%CI)
San Marino	Sickle cell disorders	0 (0 to 0)	0 (0 to 0)	0.09 (-0.02 to 0.2)	0.16 (0.11 to 0.21)	0.12 (0.09 to 0.16)	-1.15 (-1.36 to -0.93)
San Marino	Sickle cell trait	0 (0 to 0)	0 (0 to 0)	0.12 (0.04 to 0.18)	21.87 (17.68 to 26.05)	17.41 (14.33 to 20.95)	-1.04 (-1.24 to -0.84)
San Marino	G6PD deficiency	0 (0 to 0)	0 (0 to 0)	0.23 (0.2 to 0.28)	31.37 (24.12 to 39.07)	27.55 (21.18 to 34.55)	-0.68 (-0.83 to -0.54)
San Marino	G6PD trait	0 (0 to 0)	0 (0 to 0)	0.16 (0.12 to 0.21)	129.14 (115.68 to 142.89)	106.82 (95.51 to 118.18)	-0.85 (-1.03 to -0.67)
Sao Tome and Principe	**Inherited anemias**	2 (2 to 2)	2 (2 to 2)	-0.07 (-0.1 to -0.05)	1714.01 (1547.72 to 1887.84)	940.34 (852.04 to 1025.99)	-2.15 (-2.44 to -1.85)
Sao Tome and Principe	Thalassemias	0 (0 to 0)	0 (0 to 0)	0.3 (0.12 to 0.51)	0.26 (0.18 to 0.38)	0.2 (0.14 to 0.28)	-1.02 (-1.26 to -0.77)
Sao Tome and Principe	Thalassemias trait	0 (0 to 0)	0 (0 to 0)	0.1 (0.04 to 0.17)	69.96 (58.25 to 84.58)	45.62 (38.27 to 54.58)	-1.58 (-1.82 to -1.33)
Sao Tome and Principe	Sickle cell disorders	0 (0 to 0)	0 (0 to 0)	-0.29 (-0.37 to -0.19)	40.46 (30.52 to 52.9)	17.01 (12.74 to 22.12)	-2.98 (-3.49 to -2.47)
Sao Tome and Principe	Sickle cell trait	1 (1 to 1)	1 (1 to 1)	-0.16 (-0.2 to -0.1)	680.97 (597.13 to 773.93)	338.83 (291.94 to 386.61)	-2.45 (-2.82 to -2.08)
Sao Tome and Principe	G6PD deficiency	0 (0 to 0)	0 (0 to 0)	-0.04 (-0.11 to 0.03)	304.6 (235.47 to 377.86)	172.83 (133.49 to 214.68)	-1.99 (-2.26 to -1.73)
Sao Tome and Principe	G6PD trait	1 (1 to 1)	1 (1 to 1)	0 (-0.03 to 0.03)	617.77 (560.24 to 670.66)	365.85 (332.1 to 397.06)	-1.92 (-2.16 to -1.67)
Saudi Arabia	**Inherited anemias**	306 (297 to 314)	211 (192 to 230)	-0.31 (-0.37 to -0.25)	1904.66 (1852.19 to 1956.93)	589.5 (538.6 to 643.96)	-4.05 (-4.27 to -3.84)
Saudi Arabia	Thalassemias	0 (0 to 0)	0 (0 to 0)	-0.11 (-0.19 to 0)	2.05 (1.55 to 2.57)	0.82 (0.63 to 1.06)	-3.17 (-3.24 to -3.1)
Saudi Arabia	Thalassemias trait	28 (25 to 31)	25 (22 to 28)	-0.11 (-0.15 to -0.06)	174.44 (153.36 to 194.23)	69.38 (61.41 to 78.72)	-3.19 (-3.26 to -3.13)

Regions	Causes	Cases in 1990 No.×103 (95% UI)	Cases in 2019 No.×103 (95% UI)	Change in absolute number (%)	Rates in 1990 per 100000 (95% UI)	Rates in 2019 per 100000 (95% UI)	EAPC from 1990 and 2019 (95%CI)
Saudi Arabia	Sickle cell disorders	8 (7 to 8)	1 (1 to 2)	-0.81 (-0.84 to -0.78)	47.19 (43.53 to 50.81)	4 (3.3 to 4.78)	-9.51 (-10.43 to -8.57)
Saudi Arabia	Sickle cell trait	111 (107 to 115)	48 (44 to 52)	-0.57 (-0.6 to -0.53)	692.78 (668.55 to 714.87)	134.07 (122.55 to 145.92)	-6.16 (-6.63 to -5.7)
Saudi Arabia	G6PD deficiency	51 (48 to 55)	45 (35 to 57)	-0.12 (-0.31 to 0.09)	320.81 (299.99 to 344.04)	126.83 (97.64 to 159.62)	-2.01 (-2.59 to -1.42)
Saudi Arabia	G6PD trait	107 (104 to 110)	91 (84 to 97)	-0.15 (-0.21 to -0.1)	667.39 (649.7 to 685.3)	254.4 (234.96 to 271.58)	-3.33 (-3.39 to -3.27)
Senegal	**Inherited anemias**	159 (152 to 167)	211 (190 to 235)	0.33 (0.21 to 0.45)	2092.52 (1992.41 to 2186.83)	1396.93 (1257.59 to 1551.14)	-1.34 (-1.64 to -1.03)
Senegal	Thalassemias	0 (0 to 0)	0 (0 to 0)	1.25 (0.91 to 1.65)	0.16 (0.11 to 0.21)	0.18 (0.12 to 0.24)	0.44 (0.34 to 0.54)
Senegal	Thalassemias trait	4 (3 to 5)	7 (6 to 8)	0.6 (0.51 to 0.7)	54.66 (45.91 to 64.66)	43.92 (37.13 to 51.7)	-0.67 (-0.75 to -0.59)
Senegal	Sickle cell disorders	2 (1 to 2)	2 (2 to 3)	0.35 (0.24 to 0.47)	23.15 (18.59 to 27.96)	15.76 (12.69 to 19.07)	-1.22 (-1.32 to -1.13)
Senegal	Sickle cell trait	42 (38 to 47)	57 (51 to 64)	0.35 (0.29 to 0.42)	555.23 (492.95 to 614.38)	378.27 (334.67 to 420.02)	-1.21 (-1.3 to -1.11)
Senegal	G6PD deficiency	47 (43 to 50)	59 (46 to 74)	0.27 (0.01 to 0.56)	611.95 (560.43 to 661.88)	391.42 (302.25 to 487.81)	-1.58 (-2.39 to -0.77)
Senegal	G6PD trait	65 (63 to 66)	86 (79 to 92)	0.33 (0.22 to 0.43)	847.37 (822.03 to 869.98)	567.39 (519.08 to 611.14)	-1.31 (-1.59 to -1.03)
Serbia	**Inherited anemias**	20 (18 to 23)	12 (11 to 14)	-0.4 (-0.42 to -0.39)	215.37 (190.77 to 241.91)	138.4 (122.26 to 155.22)	-1.64 (-1.87 to -1.41)
Serbia	Thalassemias	0 (0 to 0)	0 (0 to 0)	-0.64 (-0.68 to -0.59)	1.27 (0.82 to 2.01)	0.49 (0.34 to 0.74)	-3.12 (-3.37 to -2.86)
Serbia	Thalassemias trait	3 (2 to 3)	2 (1 to 2)	-0.39 (-0.43 to -0.35)	28.42 (23.9 to 34.6)	18.6 (15.48 to 22.54)	-1.39 (-1.66 to -1.13)

Regions	Causes	Cases in 1990 No.×103 (95% UI)	Cases in 2019 No.×103 (95% UI)	Change in absolute number (%)	Rates in 1990 per 100000 (95% UI)	Rates in 2019 per 100000 (95% UI)	EAPC from 1990 and 2019 (95%CI)
Serbia	Sickle cell disorders	0 (0 to 0)	0 (0 to 0)	-0.37 (-0.43 to -0.31)	0.14 (0.11 to 0.19)	0.1 (0.07 to 0.13)	-1.39 (-1.69 to -1.09)
Serbia	Sickle cell trait	3 (2 to 3)	2 (1 to 2)	-0.4 (-0.42 to -0.37)	27.56 (23.83 to 31.8)	17.85 (15.14 to 20.64)	-1.44 (-1.71 to -1.17)
Serbia	G6PD deficiency	5 (4 to 6)	3 (2 to 4)	-0.37 (-0.41 to -0.32)	51.69 (40.02 to 64.75)	35.14 (27.21 to 43.66)	-2.2 (-2.53 to -1.87)
Serbia	G6PD trait	10 (9 to 11)	6 (5 to 6)	-0.42 (-0.42 to -0.42)	106.28 (94.06 to 118.18)	66.23 (58.5 to 73.73)	-1.51 (-1.76 to -1.25)
Seychelles	**Inherited anemias**	0 (0 to 0)	0 (0 to 0)	-0.21 (-0.23 to -0.19)	612.8 (546.84 to 682.25)	345.62 (310.26 to 384.69)	-1.85 (-1.95 to -1.74)
Seychelles	Thalassemias	0 (0 to 0)	0 (0 to 0)	-0.06 (-0.2 to 0.07)	3.03 (2.26 to 4.19)	2.03 (1.45 to 2.78)	-1.3 (-1.39 to -1.2)
Seychelles	Thalassemias trait	0 (0 to 0)	0 (0 to 0)	-0.03 (-0.08 to 0.01)	121.77 (103.96 to 141.47)	84.34 (71.93 to 97.89)	-1.15 (-1.23 to -1.07)
Seychelles	Sickle cell disorders	0 (0 to 0)	0 (0 to 0)	-0.12 (-0.26 to 0.07)	0.02 (0.01 to 0.02)	0.01 (0.01 to 0.01)	-1.48 (-1.57 to -1.38)
Seychelles	Sickle cell trait	0 (0 to 0)	0 (0 to 0)	-0.13 (-0.2 to -0.04)	11.25 (9.39 to 13.32)	7.03 (5.8 to 8.4)	-1.49 (-1.57 to -1.4)
Seychelles	G6PD deficiency	0 (0 to 0)	0 (0 to 0)	-0.27 (-0.28 to -0.25)	138.52 (106.67 to 174.37)	72.57 (55.97 to 90.45)	-2.08 (-2.23 to -1.93)
Seychelles	G6PD trait	0 (0 to 0)	0 (0 to 0)	-0.26 (-0.28 to -0.23)	338.22 (305.37 to 367.93)	179.64 (161.35 to 198.54)	-2.05 (-2.18 to -1.92)
Sierra Leone	**Inherited anemias**	89 (82 to 96)	151 (139 to 163)	0.7 (0.65 to 0.74)	2435.17 (2250.94 to 2624.74)	1820.84 (1678.31 to 1962.05)	-1 (-1.17 to -0.82)
Sierra Leone	Thalassemias	0 (0 to 0)	0 (0 to 0)	1.22 (0.92 to 1.56)	0.38 (0.27 to 0.57)	0.38 (0.26 to 0.53)	-0.06 (-0.23 to 0.11)
Sierra Leone	Thalassemias trait	3 (3 to 4)	6 (5 to 8)	0.9 (0.8 to 1)	91.25 (76.29 to 111.83)	76.4 (63.78 to 91.17)	-0.62 (-0.79 to -0.44)

Regions	Causes	Cases in 1990 No.×103 (95% UI)	Cases in 2019 No.×103 (95% UI)	Change in absolute number (%)	Rates in 1990 per 100000 (95% UI)	Rates in 2019 per 100000 (95% UI)	EAPC from 1990 and 2019 (95%CI)
Sierra Leone	Sickle cell disorders	4 (4 to 5)	7 (6 to 9)	0.66 (0.5 to 0.8)	120.57 (99.96 to 147.69)	88 (71.64 to 106.62)	-1.08 (-1.25 to -0.9)
Sierra Leone	Sickle cell trait	42 (39 to 47)	72 (65 to 78)	0.68 (0.61 to 0.75)	1163.42 (1065.57 to 1276.13)	863.46 (785.13 to 946.56)	-1.03 (-1.2 to -0.85)
Sierra Leone	G6PD deficiency	13 (10 to 16)	21 (16 to 26)	0.65 (0.6 to 0.72)	345.66 (267.3 to 430.92)	251.05 (193.65 to 314.88)	-1.05 (-1.25 to -0.85)
Sierra Leone	G6PD trait	26 (24 to 28)	45 (41 to 49)	0.72 (0.68 to 0.78)	713.88 (647.79 to 775.13)	541.55 (490.87 to 587.94)	-0.96 (-1.13 to -0.79)
Singapore	**Inherited anemias**	3 (3 to 4)	3 (3 to 4)	0.01 (-0.05 to 0.07)	107.75 (94.61 to 122.41)	58.58 (50.28 to 67.27)	-2.13 (-2.29 to -1.97)
Singapore	Thalassemias	0 (0 to 0)	0 (0 to 0)	-0.23 (-0.35 to -0.08)	0.56 (0.39 to 0.83)	0.23 (0.17 to 0.33)	-3.1 (-3.34 to -2.85)
Singapore	Thalassemias trait	0 (0 to 0)	1 (0 to 1)	0.61 (0.45 to 0.78)	11.69 (10.03 to 13.48)	10.14 (8.46 to 12.01)	-0.65 (-0.88 to -0.42)
Singapore	Sickle cell disorders	0 (0 to 0)	0 (0 to 0)	0.12 (-0.08 to 0.68)	0.01 (0.01 to 0.02)	0.01 (0.01 to 0.01)	-1.47 (-1.85 to -1.1)
Singapore	Sickle cell trait	0 (0 to 0)	0 (0 to 0)	0.1 (0.01 to 0.42)	7.6 (6.02 to 9.15)	4.49 (3.54 to 5.47)	-1.68 (-1.99 to -1.37)
Singapore	G6PD deficiency	1 (1 to 1)	1 (1 to 1)	-0.09 (-0.21 to 0.05)	27.05 (22.18 to 32.87)	13.3 (10.2 to 16.71)	-2.38 (-2.76 to -2)
Singapore	G6PD trait	2 (2 to 2)	2 (1 to 2)	-0.07 (-0.14 to 0)	60.84 (52 to 70.79)	30.42 (25.19 to 35.87)	-2.44 (-2.6 to -2.28)
Slovakia	**Inherited anemias**	11 (10 to 13)	8 (7 to 9)	-0.28 (-0.29 to -0.26)	212.92 (188.28 to 240.51)	149.51 (131.55 to 168.26)	-0.86 (-1.25 to -0.48)
Slovakia	Thalassemias	0 (0 to 0)	0 (0 to 0)	-0.56 (-0.61 to -0.51)	1.18 (0.79 to 1.84)	0.5 (0.34 to 0.76)	-2.35 (-2.75 to -1.95)
Slovakia	Thalassemias trait	1 (1 to 2)	1 (1 to 1)	-0.27 (-0.31 to -0.22)	27.85 (23.25 to 33.74)	19.8 (16.57 to 23.82)	-0.61 (-1.02 to -0.2)

Regions	Causes	Cases in 1990 No.×103 (95% UI)	Cases in 2019 No.×103 (95% UI)	Change in absolute number (%)	Rates in 1990 per 100000 (95% UI)	Rates in 2019 per 100000 (95% UI)	EAPC from 1990 and 2019 (95%CI)
Slovakia	Sickle cell disorders	0 (0 to 0)	0 (0 to 0)	-0.28 (-0.34 to -0.22)	0.13 (0.1 to 0.16)	0.09 (0.07 to 0.11)	-0.96 (-1.37 to -0.55)
Slovakia	Sickle cell trait	1 (1 to 1)	1 (1 to 1)	-0.28 (-0.31 to -0.25)	24.43 (20.97 to 28.1)	17 (14.61 to 19.61)	-0.81 (-1.2 to -0.42)
Slovakia	G6PD deficiency	3 (2 to 3)	2 (2 to 3)	-0.23 (-0.3 to -0.17)	51.49 (39.81 to 64.26)	38.32 (29.6 to 47.69)	-1.41 (-1.86 to -0.95)
Slovakia	G6PD trait	6 (5 to 6)	4 (4 to 4)	-0.3 (-0.3 to -0.3)	107.84 (95.28 to 120.82)	73.8 (65.21 to 82.69)	-0.7 (-1.1 to -0.3)
Slovenia	**Inherited anemias**	3 (3 to 4)	3 (2 to 3)	-0.12 (-0.13 to -0.1)	157.25 (138.08 to 177.63)	132.23 (116.39 to 148.75)	-0.03 (-0.34 to 0.28)
Slovenia	Thalassemias	0 (0 to 0)	0 (0 to 0)	-0.48 (-0.55 to -0.4)	0.77 (0.51 to 1.21)	0.38 (0.26 to 0.57)	-1.6 (-1.92 to -1.28)
Slovenia	Thalassemias trait	0 (0 to 0)	0 (0 to 0)	-0.1 (-0.16 to -0.04)	19.04 (15.61 to 23.38)	16.21 (13.56 to 19.39)	0.22 (-0.09 to 0.52)
Slovenia	Sickle cell disorders	0 (0 to 0)	0 (0 to 0)	-0.03 (-0.13 to 0.07)	0.08 (0.06 to 0.1)	0.07 (0.05 to 0.09)	0.38 (0.07 to 0.69)
Slovenia	Sickle cell trait	0 (0 to 0)	0 (0 to 0)	-0.09 (-0.14 to -0.04)	17.61 (15.15 to 20.29)	15.21 (13.07 to 17.5)	0.23 (-0.08 to 0.54)
Slovenia	G6PD deficiency	1 (1 to 1)	1 (1 to 1)	-0.07 (-0.14 to -0.02)	39.17 (30.04 to 49.12)	34.51 (26.61 to 42.98)	-0.63 (-1.07 to -0.19)
Slovenia	G6PD trait	2 (1 to 2)	1 (1 to 2)	-0.14 (-0.14 to -0.14)	80.58 (71.37 to 90.62)	65.84 (58.32 to 74.05)	0.12 (-0.2 to 0.43)
Solomon Islands	**Inherited anemias**	4 (4 to 5)	6 (6 to 7)	0.5 (0.45 to 0.56)	1246.39 (1085.28 to 1421.76)	972.06 (842.39 to 1107.94)	-0.73 (-0.97 to -0.49)
Solomon Islands	Thalassemias	0 (0 to 0)	0 (0 to 0)	0.34 (0.14 to 0.56)	5.38 (2.9 to 7.98)	3.75 (2.1 to 5.69)	-0.22 (-1.12 to 0.69)
Solomon Islands	Thalassemias trait	1 (0 to 1)	1 (1 to 1)	0.42 (0.32 to 0.52)	154.73 (119.71 to 186.64)	114.22 (89.46 to 138.81)	-0.64 (-1 to -0.27)

Regions	Causes	Cases in 1990 No.×103 (95% UI)	Cases in 2019 No.×103 (95% UI)	Change in absolute number (%)	Rates in 1990 per 100000 (95% UI)	Rates in 2019 per 100000 (95% UI)	EAPC from 1990 and 2019 (95%CI)
Solomon Islands	Sickle cell disorders	0 (0 to 0)	0 (0 to 0)	0.53 (0.44 to 2.12)	0 (0 to 0.01)	0 (0 to 0.01)	-0.66 (-0.79 to -0.53)
Solomon Islands	Sickle cell trait	0 (0 to 0)	0 (0 to 0)	0.51 (0.44 to 1.84)	5.9 (2.47 to 8.93)	4.61 (1.94 to 7.12)	-0.79 (-0.91 to -0.67)
Solomon Islands	G6PD deficiency	1 (1 to 2)	2 (2 to 3)	0.61 (0.49 to 0.75)	435.76 (337.96 to 550.26)	364.93 (282.1 to 450.53)	-0.34 (-0.79 to 0.11)
Solomon Islands	G6PD trait	2 (2 to 2)	3 (3 to 3)	0.45 (0.4 to 0.49)	644.62 (582.6 to 704.86)	484.56 (438.12 to 526.09)	-1.06 (-1.21 to -0.92)
Somalia	**Inherited anemias**	104 (92 to 118)	248 (218 to 279)	1.37 (1.32 to 1.43)	1460.54 (1281.99 to 1646.07)	1217.7 (1074.06 to 1371.27)	-0.66 (-0.87 to -0.45)
Somalia	Thalassemias	0 (0 to 0)	0 (0 to 0)	4.84 (4.22 to 6.02)	0.1 (0.06 to 0.15)	0.2 (0.11 to 0.3)	2.12 (1.88 to 2.37)
Somalia	Thalassemias trait	2 (2 to 2)	7 (6 to 9)	2.61 (2.36 to 2.96)	27.57 (21.68 to 33.65)	34.94 (27.69 to 42.54)	0.62 (0.47 to 0.78)
Somalia	Sickle cell disorders	0 (0 to 0)	0 (0 to 0)	1.64 (1.41 to 1.89)	0.41 (0.27 to 0.62)	0.38 (0.25 to 0.58)	-0.34 (-0.47 to -0.22)
Somalia	Sickle cell trait	6 (5 to 7)	15 (12 to 19)	1.62 (1.5 to 1.76)	80.35 (63.1 to 102.09)	73.97 (58.29 to 94.25)	-0.36 (-0.48 to -0.23)
Somalia	G6PD deficiency	35 (27 to 44)	74 (57 to 92)	1.11 (1.07 to 1.16)	489.42 (378.49 to 610.64)	363.44 (281.54 to 451.2)	-1.06 (-1.38 to -0.74)
Somalia	G6PD trait	62 (57 to 66)	152 (139 to 163)	1.46 (1.4 to 1.52)	862.69 (792.16 to 926.16)	744.77 (681.67 to 803.17)	-0.52 (-0.69 to -0.36)
South Africa	**Inherited anemias**	274 (266 to 280)	283 (276 to 290)	0.03 (0.03 to 0.04)	743.27 (723.26 to 760.41)	509.49 (496.12 to 521.49)	-1.11 (-1.22 to -1.01)
South Africa	Thalassemias	0 (0 to 0)	0 (0 to 0)	1.24 (0.97 to 1.53)	0.06 (0.04 to 0.09)	0.09 (0.05 to 0.13)	1.26 (1.09 to 1.43)
South Africa	Thalassemias trait	8 (6 to 9)	10 (8 to 12)	0.37 (0.28 to 0.45)	20.5 (17.35 to 23.99)	18.63 (15.26 to 21.99)	-0.26 (-0.35 to -0.18)

Regions	Causes	Cases in 1990 No.×103 (95% UI)	Cases in 2019 No.×103 (95% UI)	Change in absolute number (%)	Rates in 1990 per 100000 (95% UI)	Rates in 2019 per 100000 (95% UI)	EAPC from 1990 and 2019 (95%CI)
South Africa	Sickle cell disorders	0 (0 to 0)	0 (0 to 0)	0.06 (0.02 to 0.1)	0.08 (0.06 to 0.12)	0.06 (0.04 to 0.09)	-1.08 (-1.14 to -1.02)
South Africa	Sickle cell trait	9 (7 to 12)	10 (8 to 13)	0.07 (0.05 to 0.1)	25.15 (19.58 to 32.37)	17.86 (13.85 to 22.87)	-1.04 (-1.11 to -0.98)
South Africa	G6PD deficiency	86 (83 to 90)	86 (82 to 89)	-0.01 (-0.02 to 0)	234.76 (224.11 to 244.17)	153.91 (146.83 to 160.4)	-1.27 (-1.41 to -1.13)
South Africa	G6PD trait	170 (168 to 173)	177 (174 to 180)	0.04 (0.03 to 0.05)	462.72 (455.13 to 469.62)	318.94 (313.59 to 323.69)	-1.08 (-1.17 to -0.99)
South Sudan	**Inherited anemias**	85 (75 to 96)	112 (100 to 125)	0.32 (0.29 to 0.36)	1450.82 (1281.55 to 1630.98)	1208.88 (1075.05 to 1350.27)	-0.63 (-0.87 to -0.38)
South Sudan	Thalassemias	0 (0 to 0)	0 (0 to 0)	2.24 (1.88 to 2.9)	0.09 (0.05 to 0.13)	0.18 (0.1 to 0.28)	2.17 (1.91 to 2.42)
South Sudan	Thalassemias trait	1 (1 to 2)	3 (2 to 4)	1 (0.87 to 1.19)	25.29 (20.08 to 30.72)	31.97 (25.31 to 39.14)	0.65 (0.48 to 0.83)
South Sudan	Sickle cell disorders	0 (0 to 0)	0 (0 to 0)	0.36 (0.22 to 0.54)	2.01 (1.4 to 2.74)	1.72 (1.23 to 2.4)	-0.52 (-0.66 to -0.38)
South Sudan	Sickle cell trait	10 (9 to 12)	15 (12 to 17)	0.4 (0.33 to 0.49)	177.46 (148.21 to 207.68)	156.73 (132.77 to 185.72)	-0.43 (-0.58 to -0.29)
South Sudan	G6PD deficiency	26 (20 to 33)	31 (24 to 39)	0.18 (0.12 to 0.24)	446.21 (342.81 to 560.36)	331.28 (256.5 to 417.37)	-1.03 (-1.4 to -0.66)
South Sudan	G6PD trait	47 (43 to 51)	64 (58 to 69)	0.36 (0.32 to 0.42)	799.76 (732.43 to 862.15)	686.99 (627.78 to 742.85)	-0.51 (-0.71 to -0.31)
Spain	**Inherited anemias**	47 (42 to 52)	42 (37 to 46)	-0.11 (-0.14 to -0.08)	121.19 (109.05 to 133.84)	90.68 (81.22 to 100.63)	-0.42 (-0.76 to -0.07)
Spain	Thalassemias	0 (0 to 0)	0 (0 to 0)	-0.15 (-0.31 to -0.02)	0.1 (0.07 to 0.15)	0.07 (0.05 to 0.11)	-1.73 (-2.81 to -0.64)
Spain	Thalassemias trait	3 (3 to 4)	3 (2 to 4)	-0.05 (-0.13 to 0.03)	8.17 (6.57 to 9.84)	6.56 (5.34 to 7.86)	-0.72 (-1.28 to -0.15)

Regions	Causes	Cases in 1990 No.×103 (95% UI)	Cases in 2019 No.×103 (95% UI)	Change in absolute number (%)	Rates in 1990 per 100000 (95% UI)	Rates in 2019 per 100000 (95% UI)	EAPC from 1990 and 2019 (95%CI)
Spain	Sickle cell disorders	0 (0 to 0)	0 (0 to 0)	-0.34 (-0.42 to -0.25)	0.22 (0.15 to 0.3)	0.12 (0.09 to 0.16)	-0.6 (-1.12 to -0.08)
Spain	Sickle cell trait	10 (8 to 12)	7 (6 to 9)	-0.26 (-0.31 to -0.2)	25.64 (20.65 to 31.04)	15.94 (13.05 to 19.27)	-0.48 (-0.91 to -0.04)
Spain	G6PD deficiency	4 (3 to 5)	4 (3 to 5)	-0.01 (-0.09 to 0.06)	10.58 (8.15 to 13.24)	8.82 (6.76 to 11.11)	-0.15 (-0.46 to 0.17)
Spain	G6PD trait	30 (26 to 33)	27 (24 to 31)	-0.08 (-0.12 to -0.04)	76.48 (68.05 to 85.31)	59.17 (52.5 to 66.32)	-0.4 (-0.74 to -0.06)
Sri Lanka	**Inherited anemias**	104 (90 to 119)	79 (68 to 90)	-0.24 (-0.26 to -0.23)	603.63 (522.64 to 692.85)	359.31 (310.94 to 413.1)	-1.48 (-1.77 to -1.18)
Sri Lanka	Thalassemias	0 (0 to 0)	0 (0 to 0)	-0.37 (-0.45 to -0.28)	1.53 (1.08 to 2.27)	0.76 (0.57 to 1.05)	-2.07 (-2.27 to -1.87)
Sri Lanka	Thalassemias trait	10 (9 to 11)	9 (8 to 10)	-0.11 (-0.15 to -0.08)	58.36 (51.39 to 66.56)	40.7 (36 to 45.86)	-0.92 (-1.14 to -0.71)
Sri Lanka	Sickle cell disorders	0 (0 to 0)	0 (0 to 0)	-0.17 (-0.36 to 0.06)	0.01 (0.01 to 0.02)	0.01 (0.01 to 0.01)	-1.16 (-1.36 to -0.95)
Sri Lanka	Sickle cell trait	2 (1 to 2)	1 (1 to 2)	-0.17 (-0.27 to -0.06)	9.92 (8.27 to 11.81)	6.47 (5.33 to 7.77)	-1.17 (-1.38 to -0.95)
Sri Lanka	G6PD deficiency	40 (31 to 51)	32 (25 to 40)	-0.21 (-0.22 to -0.2)	233.19 (180.32 to 293.39)	145.34 (112.33 to 183.03)	-1.3 (-1.55 to -1.05)
Sri Lanka	G6PD trait	52 (47 to 56)	36 (33 to 40)	-0.3 (-0.32 to -0.28)	300.62 (270.91 to 327.74)	166.03 (149.07 to 181.79)	-1.75 (-2.11 to -1.39)
Sudan	**Inherited anemias**	348 (325 to 375)	402 (375 to 432)	0.15 (0.13 to 0.18)	1724.65 (1607.14 to 1856.54)	985.55 (919.32 to 1059.48)	-2.22 (-2.45 to -2)
Sudan	Thalassemias	1 (1 to 2)	1 (1 to 2)	0.17 (0.06 to 0.29)	5.74 (4.44 to 7.54)	3.33 (2.6 to 4.3)	-1.92 (-2.06 to -1.78)
Sudan	Thalassemias trait	72 (64 to 82)	85 (76 to 97)	0.18 (0.13 to 0.24)	357.55 (317.75 to 405.22)	209.48 (187.19 to 236.89)	-1.9 (-2.04 to -1.76)

Regions	Causes	Cases in 1990 No.×103 (95% UI)	Cases in 2019 No.×103 (95% UI)	Change in absolute number (%)	Rates in 1990 per 100000 (95% UI)	Rates in 2019 per 100000 (95% UI)	EAPC from 1990 and 2019 (95%CI)
Sudan	Sickle cell disorders	5 (4 to 6)	5 (4 to 7)	0.12 (0.02 to 0.22)	23.41 (18.58 to 29.52)	12.99 (10.31 to 16.31)	-2.36 (-3.07 to -1.65)
Sudan	Sickle cell trait	131 (118 to 146)	149 (134 to 166)	0.13 (0.08 to 0.18)	649.74 (584.81 to 724.9)	364.15 (327.18 to 405.76)	-2.17 (-2.56 to -1.77)
Sudan	G6PD deficiency	24 (18 to 29)	27 (21 to 34)	0.16 (0.08 to 0.23)	116.45 (90.55 to 145.15)	66.62 (51.18 to 83.32)	-2.81 (-3.06 to -2.57)
Sudan	G6PD trait	115 (103 to 127)	134 (120 to 148)	0.16 (0.12 to 0.21)	571.77 (511.41 to 628.3)	328.98 (294.41 to 363.72)	-2.41 (-2.56 to -2.26)
Suriname	**Inherited anemias**	3 (2 to 3)	3 (2 to 3)	-0.04 (-0.08 to -0.01)	703.6 (616.02 to 797.19)	451.11 (396.38 to 507.81)	-1.39 (-1.49 to -1.29)
Suriname	Thalassemias	0 (0 to 0)	0 (0 to 0)	0.57 (-0.05 to 2.05)	0.01 (0.01 to 0.02)	0.01 (0.01 to 0.02)	0.25 (-0.04 to 0.54)
Suriname	Thalassemias trait	0 (0 to 0)	0 (0 to 0)	0.13 (-0.05 to 0.43)	9.42 (7.19 to 11.9)	7.13 (5.53 to 8.56)	-0.86 (-0.99 to -0.72)
Suriname	Sickle cell disorders	0 (0 to 0)	0 (0 to 0)	0.11 (-0.02 to 0.28)	8.27 (6.05 to 10.99)	6.17 (4.35 to 8.46)	-0.99 (-1.22 to -0.76)
Suriname	Sickle cell trait	1 (1 to 1)	1 (1 to 1)	0.02 (-0.06 to 0.1)	223.76 (187.98 to 265.83)	152.96 (125.08 to 182.44)	-1.2 (-1.35 to -1.06)
Suriname	G6PD deficiency	1 (1 to 1)	1 (1 to 1)	-0.13 (-0.21 to -0.06)	223.25 (172.67 to 279.18)	131.13 (100.99 to 161.89)	-1.62 (-1.77 to -1.47)
Suriname	G6PD trait	1 (1 to 1)	1 (1 to 1)	-0.04 (-0.07 to -0.01)	238.88 (213.25 to 264.87)	153.71 (136.79 to 171.3)	-1.4 (-1.5 to -1.3)
Sweden	**Inherited anemias**	22 (21 to 23)	21 (21 to 22)	-0.03 (-0.04 to -0.01)	256.43 (248.35 to 264.81)	209.14 (201.94 to 216.96)	-0.2 (-0.54 to 0.15)
Sweden	Thalassemias	0 (0 to 0)	0 (0 to 0)	-0.13 (-0.22 to -0.04)	0.19 (0.13 to 0.26)	0.14 (0.1 to 0.19)	-1.43 (-2.14 to -0.72)
Sweden	Thalassemias trait	2 (2 to 2)	2 (2 to 2)	-0.05 (-0.1 to 0)	23.26 (20.08 to 26.56)	18.54 (16.11 to 21.17)	-0.46 (-0.77 to -0.16)

Regions	Causes	Cases in 1990 No.×103 (95% UI)	Cases in 2019 No.×103 (95% UI)	Change in absolute number (%)	Rates in 1990 per 100000 (95% UI)	Rates in 2019 per 100000 (95% UI)	EAPC from 1990 and 2019 (95%CI)
Sweden	Sickle cell disorders	0 (0 to 0)	0 (0 to 0)	0.5 (0.36 to 0.68)	0.21 (0.15 to 0.3)	0.27 (0.19 to 0.38)	1.1 (0.73 to 1.46)
Sweden	Sickle cell trait	3 (2 to 3)	3 (3 to 4)	0.2 (0.14 to 0.28)	32.17 (26.6 to 39.01)	32.52 (26.91 to 38.78)	0.46 (0.11 to 0.8)
Sweden	G6PD deficiency	3 (3 to 3)	3 (3 to 3)	-0.01 (-0.03 to 0.01)	37.84 (36.08 to 39.51)	31.43 (29.98 to 32.75)	-0.13 (-0.5 to 0.25)
Sweden	G6PD trait	14 (14 to 14)	13 (13 to 13)	-0.08 (-0.09 to -0.06)	162.76 (159.36 to 166.02)	126.23 (123.7 to 128.68)	-0.32 (-0.68 to 0.04)
Switzerland	**Inherited anemias**	15 (14 to 17)	16 (14 to 17)	0.02 (0 to 0.05)	223.22 (200.84 to 248.11)	178.8 (160.04 to 198.01)	-0.78 (-0.99 to -0.57)
Switzerland	Thalassemias	0 (0 to 0)	0 (0 to 0)	-0.04 (-0.17 to 0.09)	0.16 (0.1 to 0.21)	0.12 (0.08 to 0.17)	-1.84 (-2.48 to -1.2)
Switzerland	Thalassemias trait	1 (1 to 2)	1 (1 to 2)	0.04 (-0.02 to 0.11)	19.72 (17.09 to 22.38)	16.1 (13.96 to 18.51)	-0.91 (-1.09 to -0.74)
Switzerland	Sickle cell disorders	0 (0 to 0)	0 (0 to 0)	0.17 (0.05 to 0.28)	0.31 (0.23 to 0.4)	0.28 (0.21 to 0.36)	-0.4 (-0.64 to -0.16)
Switzerland	Sickle cell trait	2 (1 to 2)	2 (1 to 2)	0.02 (-0.04 to 0.08)	24.65 (20.07 to 29.64)	19.65 (16.1 to 23.69)	-0.79 (-1 to -0.57)
Switzerland	G6PD deficiency	2 (2 to 3)	3 (2 to 3)	0.08 (0.03 to 0.15)	34.6 (26.67 to 43.37)	29.22 (22.48 to 36.46)	-0.61 (-0.87 to -0.35)
Switzerland	G6PD trait	10 (9 to 11)	10 (9 to 11)	0.01 (-0.02 to 0.04)	143.78 (128.77 to 158.87)	113.43 (101.59 to 125.33)	-0.8 (-1.02 to -0.59)
Syrian Arab Republic	**Inherited anemias**	157 (144 to 171)	72 (66 to 78)	-0.54 (-0.55 to -0.53)	1221.06 (1118.79 to 1327.49)	496 (455.34 to 537.4)	-3.09 (-3.32 to -2.86)
Syrian Arab Republic	Thalassemias	1 (0 to 1)	0 (0 to 0)	-0.53 (-0.58 to -0.49)	4.75 (3.67 to 6.19)	1.97 (1.54 to 2.55)	-3.09 (-3.32 to -2.86)
Syrian Arab Republic	Thalassemias trait	36 (32 to 41)	17 (15 to 19)	-0.53 (-0.55 to -0.51)	280.27 (249.61 to 316.96)	116.39 (103.71 to 130.99)	-3.07 (-3.3 to -2.84)

Regions	Causes	Cases in 1990 No.×103 (95% UI)	Cases in 2019 No.×103 (95% UI)	Change in absolute number (%)	Rates in 1990 per 100000 (95% UI)	Rates in 2019 per 100000 (95% UI)	EAPC from 1990 and 2019 (95%CI)
Syrian Arab Republic	Sickle cell disorders	0 (0 to 0)	0 (0 to 0)	-0.54 (-0.57 to -0.51)	1.36 (1.09 to 1.65)	0.55 (0.45 to 0.67)	-3.15 (-3.37 to -2.94)
Syrian Arab Republic	Sickle cell trait	18 (16 to 20)	8 (7 to 9)	-0.55 (-0.56 to -0.53)	141.86 (126.81 to 156.43)	57.08 (51.29 to 63.1)	-3.16 (-3.39 to -2.94)
Syrian Arab Republic	G6PD deficiency	21 (16 to 26)	9 (7 to 12)	-0.55 (-0.57 to -0.53)	159.44 (122.6 to 200.2)	64.14 (49.44 to 79.83)	-2.87 (-3.13 to -2.6)
Syrian Arab Republic	G6PD trait	82 (74 to 89)	37 (34 to 40)	-0.55 (-0.56 to -0.53)	633.39 (574.63 to 690.69)	255.88 (232.42 to 277.01)	-3.13 (-3.35 to -2.92)
Taiwan (Province of China)	**Inherited anemias**	55 (50 to 60)	29 (26 to 33)	-0.47 (-0.5 to -0.44)	269.44 (246.88 to 294.44)	123.06 (109.61 to 137.75)	-2.74 (-3.12 to -2.37)
Taiwan (Province of China)	Thalassemias	0 (0 to 0)	0 (0 to 0)	-0.62 (-0.66 to -0.58)	0.64 (0.5 to 0.85)	0.21 (0.17 to 0.26)	-3.88 (-4.29 to -3.46)
Taiwan (Province of China)	Thalassemias trait	8 (7 to 9)	4 (4 to 5)	-0.46 (-0.5 to -0.41)	37.55 (32.87 to 43.07)	17.63 (15.9 to 19.49)	-2.64 (-3.06 to -2.23)
Taiwan (Province of China)	Sickle cell disorders	0 (0 to 0)	0 (0 to 0)	-0.17 (-0.3 to -0.01)	0.04 (0.03 to 0.05)	0.03 (0.02 to 0.03)	-0.8 (-1.47 to -0.12)
Taiwan (Province of China)	Sickle cell trait	3 (3 to 3)	2 (2 to 2)	-0.35 (-0.39 to -0.28)	14.39 (12.35 to 16.89)	8.13 (7.1 to 9.25)	-1.84 (-2.35 to -1.32)
Taiwan (Province of China)	G6PD deficiency	10 (9 to 12)	5 (4 to 7)	-0.48 (-0.56 to -0.39)	51.13 (44.49 to 58.33)	22.95 (17.68 to 28.72)	-2.85 (-3.29 to -2.41)
Taiwan (Province of China)	G6PD trait	34 (30 to 37)	18 (16 to 19)	-0.48 (-0.51 to -0.45)	165.71 (149.06 to 183.03)	74.12 (66.07 to 82.44)	-2.82 (-3.16 to -2.48)
Tajikistan	**Inherited anemias**	34 (31 to 37)	42 (38 to 46)	0.24 (0.21 to 0.26)	624.59 (567.65 to 684.56)	437.42 (398 to 479.79)	-0.85 (-1.08 to -0.62)
Tajikistan	Thalassemias	0 (0 to 0)	0 (0 to 0)	0.05 (-0.06 to 0.18)	6.26 (4.84 to 8.11)	3.73 (2.92 to 4.78)	-1.39 (-1.63 to -1.16)
Tajikistan	Thalassemias trait	13 (12 to 15)	17 (15 to 19)	0.26 (0.2 to 0.32)	246.68 (217.58 to 278.04)	176.18 (156.47 to 200.38)	-0.8 (-1.03 to -0.57)

Regions	Causes	Cases in 1990 No.×103 (95% UI)	Cases in 2019 No.×103 (95% UI)	Change in absolute number (%)	Rates in 1990 per 100000 (95% UI)	Rates in 2019 per 100000 (95% UI)	EAPC from 1990 and 2019 (95%CI)
Tajikistan	Sickle cell disorders	0 (0 to 0)	0 (0 to 0)	0.23 (0.15 to 0.42)	0.02 (0.02 to 0.04)	0.02 (0.01 to 0.03)	-0.87 (-1.11 to -0.64)
Tajikistan	Sickle cell trait	1 (1 to 1)	1 (1 to 2)	0.23 (0.22 to 0.33)	18.3 (14.29 to 22.7)	12.74 (9.91 to 15.84)	-0.88 (-1.11 to -0.65)
Tajikistan	G6PD deficiency	3 (2 to 4)	4 (3 to 5)	0.25 (0.19 to 0.33)	53.61 (41.13 to 66.76)	37.91 (29.36 to 48.29)	-0.76 (-0.98 to -0.54)
Tajikistan	G6PD trait	16 (14 to 18)	20 (17 to 22)	0.22 (0.22 to 0.22)	299.72 (266.93 to 332.69)	206.84 (184.21 to 229.59)	-0.9 (-1.13 to -0.67)
Thailand	**Inherited anemias**	307 (291 to 324)	178 (163 to 195)	-0.42 (-0.45 to -0.39)	540.17 (510.97 to 568.92)	253.95 (232.07 to 278.11)	-2.51 (-2.59 to -2.42)
Thailand	Thalassemias	17 (14 to 22)	8 (7 to 9)	-0.53 (-0.6 to -0.47)	29.28 (23.89 to 37.95)	11.08 (9.29 to 13.47)	-3.25 (-3.32 to -3.18)
Thailand	Thalassemias trait	114 (105 to 124)	72 (66 to 79)	-0.36 (-0.39 to -0.34)	200.02 (183.79 to 218.84)	103.13 (94.43 to 112.91)	-2.2 (-2.27 to -2.12)
Thailand	Sickle cell disorders	0 (0 to 0)	0 (0 to 0)	-0.46 (-0.58 to -0.39)	0.01 (0.01 to 0.02)	0.01 (0 to 0.01)	-2.72 (-2.79 to -2.65)
Thailand	Sickle cell trait	5 (4 to 6)	3 (2 to 4)	-0.45 (-0.51 to -0.41)	9.3 (7.58 to 11.36)	4.12 (3.37 to 4.99)	-2.7 (-2.77 to -2.63)
Thailand	G6PD deficiency	54 (50 to 59)	33 (25 to 41)	-0.4 (-0.52 to -0.28)	95.71 (87.83 to 103.71)	46.64 (36.06 to 58.4)	-2.23 (-2.44 to -2.03)
Thailand	G6PD trait	117 (109 to 125)	62 (56 to 68)	-0.47 (-0.51 to -0.43)	205.84 (190.79 to 220.26)	88.98 (79.74 to 97.4)	-2.87 (-2.93 to -2.81)
Timor-Leste	**Inherited anemias**	11 (10 to 12)	10 (9 to 11)	-0.07 (-0.09 to -0.04)	1380.62 (1232.49 to 1529.03)	755.06 (678.46 to 835.57)	-2.37 (-2.51 to -2.23)
Timor-Leste	Thalassemias	0 (0 to 0)	0 (0 to 0)	0.06 (-0.08 to 0.2)	11.17 (8.42 to 15.2)	6.92 (5.1 to 9.05)	-1.96 (-2.07 to -1.85)
Timor-Leste	Thalassemias trait	3 (2 to 3)	3 (3 to 4)	0.11 (0.05 to 0.16)	352.55 (301.66 to 408)	229.47 (197.2 to 265.79)	-1.79 (-1.89 to -1.68)

Regions	Causes	Cases in 1990 No.×103 (95% UI)	Cases in 2019 No.×103 (95% UI)	Change in absolute number (%)	Rates in 1990 per 100000 (95% UI)	Rates in 2019 per 100000 (95% UI)	EAPC from 1990 and 2019 (95%CI)
Timor-Leste	Sickle cell disorders	0 (0 to 0)	0 (0 to 0)	0.01 (-0.21 to 0.16)	0.04 (0.03 to 0.06)	0.03 (0.02 to 0.04)	-2.05 (-2.14 to -1.97)
Timor-Leste	Sickle cell trait	0 (0 to 0)	0 (0 to 0)	0.02 (-0.09 to 0.09)	27.26 (22.54 to 32.42)	16.24 (13.33 to 19.32)	-2.05 (-2.14 to -1.96)
Timor-Leste	G6PD deficiency	2 (2 to 3)	2 (1 to 2)	-0.15 (-0.21 to -0.08)	288.53 (223.36 to 358.18)	144.48 (111.7 to 180.19)	-2.65 (-2.84 to -2.46)
Timor-Leste	G6PD trait	5 (5 to 6)	5 (4 to 5)	-0.13 (-0.16 to -0.09)	701.07 (634.82 to 762.43)	357.91 (321.49 to 395.6)	-2.6 (-2.78 to -2.42)
Togo	**Inherited anemias**	76 (70 to 83)	110 (101 to 119)	0.44 (0.4 to 0.48)	2081.8 (1921.66 to 2261.14)	1386.94 (1272.93 to 1507.7)	-1.18 (-1.35 to -1.01)
Togo	Thalassemias	0 (0 to 0)	0 (0 to 0)	1.01 (0.63 to 1.52)	0.27 (0.14 to 0.53)	0.25 (0.14 to 0.42)	0.05 (-0.14 to 0.24)
Togo	Thalassemias trait	3 (2 to 4)	4 (3 to 6)	0.64 (0.54 to 0.77)	73.55 (50.96 to 106.74)	55.93 (39.78 to 77.32)	-0.69 (-0.86 to -0.51)
Togo	Sickle cell disorders	3 (3 to 4)	5 (4 to 6)	0.44 (0.31 to 0.57)	91.04 (80.1 to 105.35)	60.54 (51.14 to 70.69)	-1.16 (-1.35 to -0.97)
Togo	Sickle cell trait	32 (29 to 35)	45 (41 to 49)	0.42 (0.34 to 0.5)	869.08 (803.64 to 943.45)	569.74 (512.65 to 621.71)	-1.22 (-1.39 to -1.06)
Togo	G6PD deficiency	13 (10 to 16)	18 (14 to 22)	0.41 (0.33 to 0.48)	342.64 (265.06 to 429)	222.66 (172.08 to 281)	-1.23 (-1.43 to -1.03)
Togo	G6PD trait	26 (24 to 28)	38 (34 to 41)	0.47 (0.44 to 0.5)	705.23 (641.81 to 766.02)	477.82 (433.77 to 521.36)	-1.15 (-1.32 to -0.98)
Tokelau	**Inherited anemias**	0 (0 to 0)	0 (0 to 0)	-0.34 (-0.35 to -0.33)	791.83 (704.15 to 884.8)	628.71 (558.08 to 701.1)	-0.83 (-1.05 to -0.61)
Tokelau	Thalassemias	0 (0 to 0)	0 (0 to 0)	-0.46 (-0.53 to -0.38)	4.13 (2.25 to 6.42)	2.65 (1.44 to 4.05)	-0.47 (-1.45 to 0.52)
Tokelau	Thalassemias trait	0 (0 to 0)	0 (0 to 0)	-0.42 (-0.45 to -0.38)	125.87 (97.9 to 153.71)	87.04 (66.79 to 106.27)	-0.89 (-1.32 to -0.45)

Regions	Causes	Cases in 1990 No.×103 (95% UI)	Cases in 2019 No.×103 (95% UI)	Change in absolute number (%)	Rates in 1990 per 100000 (95% UI)	Rates in 2019 per 100000 (95% UI)	EAPC from 1990 and 2019 (95%CI)
Tokelau	Sickle cell disorders	0 (0 to 0)	0 (0 to 0)	-0.4 (-0.41 to -0.18)	0 (0 to 0.01)	0 (0 to 0)	-1.04 (-1.24 to -0.84)
Tokelau	Sickle cell trait	0 (0 to 0)	0 (0 to 0)	-0.4 (-0.41 to -0.23)	4.88 (2.01 to 7.66)	3.53 (1.52 to 5.44)	-1.11 (-1.26 to -0.95)
Tokelau	G6PD deficiency	0 (0 to 0)	0 (0 to 0)	-0.28 (-0.3 to -0.27)	163.57 (126.26 to 203.65)	141.26 (109.11 to 175.93)	-0.62 (-0.88 to -0.36)
Tokelau	G6PD trait	0 (0 to 0)	0 (0 to 0)	-0.33 (-0.34 to -0.32)	493.37 (444.07 to 542.2)	394.24 (356.04 to 429)	-0.9 (-1.09 to -0.71)
Tonga	Inherited anemias	1 (0 to 1)	1 (0 to 1)	0 (-0.02 to 0.03)	545.71 (486.07 to 612.09)	517.02 (459.71 to 577)	-0.02 (-0.12 to 0.09)
Tonga	Thalassemias	0 (0 to 0)	0 (0 to 0)	-0.19 (-0.3 to -0.07)	2.79 (1.56 to 4.26)	2.14 (1.16 to 3.22)	0.4 (-0.58 to 1.39)
Tonga	Thalassemias trait	0 (0 to 0)	0 (0 to 0)	-0.12 (-0.18 to -0.07)	86.57 (67.34 to 105.4)	71.76 (55.04 to 86.75)	-0.04 (-0.44 to 0.35)
Tonga	Sickle cell disorders	0 (0 to 0)	0 (0 to 0)	-0.01 (-0.1 to 0.95)	0 (0 to 0)	0 (0 to 0)	-0.13 (-0.25 to -0.01)
Tonga	Sickle cell trait	0 (0 to 0)	0 (0 to 0)	-0.04 (-0.1 to 0.45)	3.4 (1.5 to 5.26)	3.09 (1.27 to 4.84)	-0.24 (-0.33 to -0.15)
Tonga	G6PD deficiency	0 (0 to 0)	0 (0 to 0)	0.09 (0.02 to 0.17)	110.99 (85.41 to 140.5)	114.17 (88.45 to 144.15)	0.19 (0.06 to 0.32)
Tonga	G6PD trait	0 (0 to 0)	0 (0 to 0)	0.01 (-0.01 to 0.03)	341.96 (307.87 to 376.93)	325.85 (294.77 to 354.77)	-0.09 (-0.15 to -0.03)
Trinidad and Tobago	Inherited anemias	6 (6 to 7)	4 (4 to 5)	-0.32 (-0.34 to -0.29)	532.8 (470.78 to 603.1)	315.06 (279.05 to 356.29)	-1.09 (-1.37 to -0.81)
Trinidad and Tobago	Thalassemias	0 (0 to 0)	0 (0 to 0)	0.09 (-0.35 to 1.22)	0.01 (0.01 to 0.02)	0.01 (0 to 0.01)	0.32 (0.08 to 0.56)
Trinidad and Tobago	Thalassemias trait	0 (0 to 0)	0 (0 to 0)	-0.22 (-0.34 to 0)	6.99 (5.32 to 8.93)	4.72 (3.63 to 5.74)	-0.74 (-0.94 to -0.55)

Regions	Causes	Cases in 1990 No.×103 (95% UI)	Cases in 2019 No.×103 (95% UI)	Change in absolute number (%)	Rates in 1990 per 100000 (95% UI)	Rates in 2019 per 100000 (95% UI)	EAPC from 1990 and 2019 (95%CI)
Trinidad and Tobago	Sickle cell disorders	0 (0 to 0)	0 (0 to 0)	-0.25 (-0.34 to -0.17)	6.54 (4.73 to 8.82)	4.24 (2.98 to 5.76)	-0.66 (-1.14 to -0.19)
Trinidad and Tobago	Sickle cell trait	2 (2 to 3)	2 (1 to 2)	-0.3 (-0.35 to -0.26)	184.55 (154.26 to 216.5)	111.28 (92.73 to 131.72)	-0.94 (-1.31 to -0.57)
Trinidad and Tobago	G6PD deficiency	2 (1 to 2)	1 (1 to 2)	-0.31 (-0.38 to -0.25)	149.72 (115.44 to 187.65)	89.19 (68.94 to 111.02)	-1.09 (-1.35 to -0.84)
Trinidad and Tobago	G6PD trait	2 (2 to 2)	1 (1 to 2)	-0.34 (-0.37 to -0.32)	184.99 (164.99 to 203.53)	105.63 (94.18 to 116.43)	-1.27 (-1.5 to -1.03)
Tunisia	**Inherited anemias**	89 (82 to 98)	61 (55 to 66)	-0.32 (-0.34 to -0.3)	1057.92 (970.61 to 1158.8)	523.1 (476.61 to 574.07)	-2.14 (-2.26 to -2.01)
Tunisia	Thalassemias	0 (0 to 0)	0 (0 to 0)	-0.42 (-0.49 to -0.35)	3.73 (2.77 to 4.95)	1.57 (1.19 to 2.03)	-2.46 (-2.73 to -2.18)
Tunisia	Thalassemias trait	18 (15 to 20)	12 (10 to 13)	-0.35 (-0.38 to -0.31)	209.85 (183 to 240.43)	99.86 (87.87 to 112.8)	-2.17 (-2.36 to -1.98)
Tunisia	Sickle cell disorders	0 (0 to 0)	0 (0 to 0)	-0.45 (-0.5 to -0.41)	3.81 (3.11 to 4.65)	1.52 (1.2 to 1.9)	-2.38 (-2.68 to -2.09)
Tunisia	Sickle cell trait	17 (15 to 19)	10 (9 to 12)	-0.38 (-0.41 to -0.36)	200.49 (181.98 to 220.9)	90.26 (80.79 to 100.92)	-2.21 (-2.41 to -2.01)
Tunisia	G6PD deficiency	16 (13 to 20)	12 (9 to 14)	-0.28 (-0.34 to -0.22)	190.92 (150.89 to 240.78)	99.82 (76.77 to 124.97)	-2.32 (-2.49 to -2.15)
Tunisia	G6PD trait	38 (34 to 41)	27 (24 to 29)	-0.3 (-0.32 to -0.27)	449.12 (407.63 to 488.14)	230.08 (209.29 to 250.88)	-2.01 (-2.14 to -1.88)
Turkey	**Inherited anemias**	572 (554 to 589)	325 (296 to 358)	-0.43 (-0.48 to -0.38)	956.81 (926.94 to 985.17)	399.9 (363.36 to 439.6)	-2.9 (-3.23 to -2.57)
Turkey	Thalassemias	1 (1 to 1)	1 (0 to 1)	-0.25 (-0.34 to -0.15)	1.32 (1 to 1.72)	0.73 (0.55 to 0.94)	-2.24 (-2.56 to -1.93)
Turkey	Thalassemias trait	77 (68 to 88)	51 (45 to 58)	-0.33 (-0.37 to -0.29)	129.16 (113.15 to 147.9)	63.27 (55.66 to 71.67)	-2.77 (-2.98 to -2.56)

Regions	Causes	Cases in 1990 No.×103 (95% UI)	Cases in 2019 No.×103 (95% UI)	Change in absolute number (%)	Rates in 1990 per 100000 (95% UI)	Rates in 2019 per 100000 (95% UI)	EAPC from 1990 and 2019 (95%CI)
Turkey	Sickle cell disorders	1 (1 to 1)	0 (0 to 0)	-0.69 (-0.71 to -0.66)	1.58 (1.34 to 1.88)	0.36 (0.31 to 0.42)	-5.9 (-6.32 to -5.48)
Turkey	Sickle cell trait	75 (69 to 82)	32 (29 to 34)	-0.58 (-0.6 to -0.56)	125.53 (115.45 to 137.34)	38.77 (35.94 to 41.85)	-4.7 (-5 to -4.39)
Turkey	G6PD deficiency	113 (107 to 119)	66 (52 to 84)	-0.41 (-0.54 to -0.26)	188.66 (178.76 to 198.78)	81.64 (63.66 to 103.33)	-1.89 (-2.45 to -1.33)
Turkey	G6PD trait	305 (299 to 311)	175 (161 to 188)	-0.43 (-0.47 to -0.38)	510.57 (499.54 to 519.99)	215.12 (197.28 to 231.41)	-2.84 (-3.23 to -2.45)
Turkmenistan	**Inherited anemias**	20 (18 to 22)	17 (15 to 19)	-0.15 (-0.17 to -0.13)	535.74 (479.69 to 594.26)	330.6 (295.23 to 368.11)	-1.18 (-1.55 to -0.81)
Turkmenistan	Thalassemias	0 (0 to 0)	0 (0 to 0)	-0.32 (-0.4 to -0.22)	4.28 (3.08 to 5.91)	2.12 (1.51 to 2.96)	-1.98 (-2.34 to -1.61)
Turkmenistan	Thalassemias trait	7 (6 to 8)	6 (5 to 7)	-0.14 (-0.19 to -0.08)	181.22 (151.21 to 220.77)	113.9 (94.5 to 138.32)	-1.16 (-1.53 to -0.8)
Turkmenistan	Sickle cell disorders	0 (0 to 0)	0 (0 to 0)	-0.16 (-0.27 to -0.03)	0.02 (0.01 to 0.03)	0.01 (0.01 to 0.02)	-1.17 (-1.55 to -0.79)
Turkmenistan	Sickle cell trait	1 (0 to 1)	1 (0 to 1)	-0.16 (-0.22 to -0.09)	17.01 (13.4 to 21.1)	10.47 (8.24 to 13.03)	-1.17 (-1.55 to -0.8)
Turkmenistan	G6PD deficiency	2 (1 to 2)	2 (1 to 2)	-0.14 (-0.19 to -0.09)	50.13 (38.64 to 63.73)	31.53 (24.26 to 39.62)	-1.04 (-1.44 to -0.65)
Turkmenistan	G6PD trait	10 (9 to 12)	9 (8 to 10)	-0.16 (-0.18 to -0.16)	283.07 (252.27 to 317.08)	172.57 (153.87 to 192.26)	-1.21 (-1.58 to -0.84)
Tuvalu	**Inherited anemias**	0 (0 to 0)	0 (0 to 0)	-0.19 (-0.21 to -0.18)	683.21 (605.24 to 763.66)	435.91 (385.58 to 484.93)	-1.5 (-1.78 to -1.21)
Tuvalu	Thalassemias	0 (0 to 0)	0 (0 to 0)	-0.33 (-0.42 to -0.24)	3.87 (2.1 to 5.85)	2.05 (1.14 to 3.15)	-1.09 (-2.04 to -0.13)
Tuvalu	Thalassemias trait	0 (0 to 0)	0 (0 to 0)	-0.29 (-0.34 to -0.24)	112.35 (86.79 to 136.29)	63.24 (48.94 to 77.56)	-1.54 (-1.96 to -1.11)

Regions	Causes	Cases in 1990 No.×103 (95% UI)	Cases in 2019 No.×103 (95% UI)	Change in absolute number (%)	Rates in 1990 per 100000 (95% UI)	Rates in 2019 per 100000 (95% UI)	EAPC from 1990 and 2019 (95%CI)
Tuvalu	Sickle cell disorders	0 (0 to 0)	0 (0 to 0)	-0.19 (-0.28 to 1.05)	0 (0 to 0)	0 (0 to 0)	-1.43 (-1.65 to -1.21)
Tuvalu	Sickle cell trait	0 (0 to 0)	0 (0 to 0)	-0.22 (-0.28 to 0.67)	4.28 (1.82 to 6.56)	2.65 (1.13 to 4.53)	-1.6 (-1.8 to -1.41)
Tuvalu	G6PD deficiency	0 (0 to 0)	0 (0 to 0)	-0.13 (-0.18 to -0.08)	140.46 (108.48 to 176.68)	97.3 (75.45 to 120.99)	-1.29 (-1.63 to -0.94)
Tuvalu	G6PD trait	0 (0 to 0)	0 (0 to 0)	-0.19 (-0.2 to -0.18)	422.25 (380.12 to 464.03)	270.67 (244.46 to 294.49)	-1.57 (-1.84 to -1.3)
Uganda	**Inherited anemias**	437 (398 to 477)	776 (702 to 857)	0.78 (0.71 to 0.83)	2521.18 (2300.3 to 2753)	1887.75 (1706.89 to 2084.47)	-1.27 (-1.38 to -1.17)
Uganda	Thalassemias	0 (0 to 0)	0 (0 to 0)	2.81 (2.32 to 3.66)	0.11 (0.07 to 0.16)	0.18 (0.1 to 0.27)	1.22 (0.99 to 1.46)
Uganda	Thalassemias trait	5 (4 to 7)	13 (10 to 16)	1.36 (1.19 to 1.62)	31.6 (25.19 to 38.37)	31.35 (24.7 to 38.36)	-0.26 (-0.41 to -0.12)
Uganda	Sickle cell disorders	6 (5 to 8)	9 (7 to 11)	0.4 (0.15 to 0.66)	35.32 (28.23 to 46.4)	20.83 (16.22 to 25.69)	-2.29 (-2.48 to -2.1)
Uganda	Sickle cell trait	139 (125 to 157)	216 (192 to 238)	0.55 (0.42 to 0.68)	801.22 (724.19 to 909)	524.36 (467.09 to 578.72)	-1.73 (-1.83 to -1.63)
Uganda	G6PD deficiency	93 (72 to 116)	209 (161 to 260)	1.26 (1.13 to 1.38)	535.6 (413.96 to 670.91)	508.79 (392.74 to 631.39)	-0.69 (-0.88 to -0.49)
Uganda	G6PD trait	193 (179 to 206)	330 (305 to 351)	0.71 (0.7 to 0.71)	1117.32 (1033.75 to 1190.74)	802.25 (742.63 to 854.51)	-1.27 (-1.37 to -1.16)
Ukraine	**Inherited anemias**	82 (78 to 86)	51 (48 to 53)	-0.38 (-0.39 to -0.37)	155.35 (148.69 to 162.97)	114.72 (109.98 to 120.18)	-0.02 (-0.45 to 0.42)
Ukraine	Thalassemias	0 (0 to 1)	0 (0 to 0)	-0.58 (-0.63 to -0.52)	0.76 (0.53 to 1.11)	0.38 (0.27 to 0.55)	-1.26 (-1.72 to -0.81)
Ukraine	Thalassemias trait	17 (14 to 21)	11 (9 to 13)	-0.36 (-0.4 to -0.31)	31.96 (26.35 to 39.07)	24.42 (20.29 to 29.32)	0.12 (-0.31 to 0.55)

Regions	Causes	Cases in 1990 No.×103 (95% UI)	Cases in 2019 No.×103 (95% UI)	Change in absolute number (%)	Rates in 1990 per 100000 (95% UI)	Rates in 2019 per 100000 (95% UI)	EAPC from 1990 and 2019 (95%CI)
Ukraine	Sickle cell disorders	0 (0 to 0)	0 (0 to 0)	-0.38 (-0.48 to -0.24)	0.01 (0.01 to 0.01)	0.01 (0 to 0.01)	-0.02 (-0.45 to 0.42)
Ukraine	Sickle cell trait	3 (3 to 4)	2 (2 to 2)	-0.39 (-0.44 to -0.32)	6.05 (4.84 to 7.42)	4.42 (3.54 to 5.38)	-0.01 (-0.45 to 0.42)
Ukraine	G6PD deficiency	13 (12 to 13)	8 (8 to 9)	-0.35 (-0.36 to -0.34)	23.99 (22.85 to 25.06)	18.65 (17.76 to 19.43)	-0.14 (-0.57 to 0.29)
Ukraine	G6PD trait	49 (48 to 50)	29 (29 to 30)	-0.4 (-0.4 to -0.4)	92.59 (90.32 to 94.69)	66.85 (65.22 to 68.36)	-0.02 (-0.47 to 0.42)
United Arab Emirates	**Inherited anemias**	17 (16 to 18)	20 (18 to 22)	0.18 (0.08 to 0.26)	897.72 (841.26 to 944.8)	214.58 (193.63 to 236.6)	-4.38 (-4.58 to -4.17)
United Arab Emirates	Thalassemias	0 (0 to 0)	0 (0 to 0)	0.31 (0.15 to 0.47)	2.24 (1.65 to 3.01)	0.6 (0.45 to 0.82)	-3.98 (-4.2 to -3.77)
United Arab Emirates	Thalassemias trait	3 (2 to 3)	4 (3 to 4)	0.28 (0.21 to 0.35)	152.31 (132.08 to 174.82)	39.58 (34.77 to 45.67)	-4.07 (-4.29 to -3.85)
United Arab Emirates	Sickle cell disorders	0 (0 to 0)	0 (0 to 0)	0.19 (0.12 to 0.28)	0.73 (0.6 to 0.88)	0.18 (0.14 to 0.21)	-4.52 (-4.75 to -4.29)
United Arab Emirates	Sickle cell trait	2 (1 to 2)	2 (2 to 2)	0.2 (0.16 to 0.24)	83.27 (75.85 to 91.33)	20.25 (18.42 to 22.21)	-4.39 (-4.61 to -4.17)
United Arab Emirates	G6PD deficiency	4 (4 to 5)	5 (4 to 6)	0.05 (-0.16 to 0.26)	233.69 (198.76 to 260.3)	49.72 (38.43 to 62.8)	-4.88 (-5.02 to -4.74)
United Arab Emirates	G6PD trait	8 (8 to 8)	10 (9 to 10)	0.21 (0.12 to 0.3)	425.47 (408.17 to 447.7)	104.27 (95.15 to 112.51)	-4.23 (-4.47 to -3.99)
United Kingdom	**Inherited anemias**	146 (138 to 156)	148 (142 to 155)	0.01 (-0.01 to 0.03)	254.85 (240.95 to 271.5)	220.32 (210.67 to 231.06)	-0.23 (-0.43 to -0.03)
United Kingdom	Thalassemias	0 (0 to 0)	0 (0 to 0)	-0.06 (-0.13 to -0.01)	0.08 (0.06 to 0.11)	0.06 (0.04 to 0.09)	-0.63 (-0.78 to -0.49)
United Kingdom	Thalassemias trait	11 (10 to 14)	11 (9 to 14)	-0.01 (-0.04 to 0.01)	19.88 (16.67 to 24.38)	16.77 (13.91 to 20.71)	-0.4 (-0.56 to -0.24)

Regions	Causes	Cases in 1990 No.×103 (95% UI)	Cases in 2019 No.×103 (95% UI)	Change in absolute number (%)	Rates in 1990 per 100000 (95% UI)	Rates in 2019 per 100000 (95% UI)	EAPC from 1990 and 2019 (95%CI)
United Kingdom	Sickle cell disorders	1 (0 to 1)	1 (0 to 1)	0.04 (-0.08 to 0.16)	1.1 (0.77 to 1.55)	0.98 (0.72 to 1.29)	-0.12 (-0.4 to 0.16)
United Kingdom	Sickle cell trait	31 (24 to 40)	30 (24 to 36)	-0.05 (-0.13 to 0.04)	54.28 (42.14 to 69.23)	43.97 (35.93 to 52.88)	-0.37 (-0.59 to -0.15)
United Kingdom	G6PD deficiency	12 (11 to 12)	19 (18 to 20)	0.63 (0.61 to 0.66)	20.57 (19.6 to 21.51)	28.72 (27.44 to 29.88)	1.45 (0.86 to 2.06)
United Kingdom	G6PD trait	91 (90 to 93)	87 (86 to 89)	-0.04 (-0.05 to -0.04)	158.94 (155.75 to 161.99)	129.82 (127.29 to 132.13)	-0.44 (-0.64 to -0.25)
United Republic of Tanzania	**Inherited anemias**	553 (501 to 607)	877 (797 to 960)	0.59 (0.55 to 0.62)	2134.49 (1934.72 to 2344.4)	1546.02 (1404.45 to 1691.26)	-1.07 (-1.26 to -0.89)
United Republic of Tanzania	Thalassemias	0 (0 to 0)	0 (0 to 0)	2.81 (2.3 to 3.48)	0.1 (0.06 to 0.15)	0.17 (0.09 to 0.25)	1.63 (1.42 to 1.85)
United Republic of Tanzania	Thalassemias trait	7 (6 to 9)	17 (13 to 21)	1.35 (1.17 to 1.55)	27.8 (22.03 to 33.89)	29.77 (23.38 to 36.25)	0.12 (0 to 0.25)
United Republic of Tanzania	Sickle cell disorders	7 (6 to 9)	13 (10 to 16)	0.79 (0.62 to 0.99)	27.74 (21.73 to 34.23)	22.7 (18.12 to 28.1)	-0.71 (-0.92 to -0.5)
United Republic of Tanzania	Sickle cell trait	173 (155 to 191)	301 (271 to 332)	0.74 (0.66 to 0.82)	669.22 (598.21 to 738.77)	530.17 (478.51 to 585.45)	-0.8 (-0.94 to -0.66)
United Republic of Tanzania	G6PD deficiency	134 (104 to 166)	177 (137 to 223)	0.32 (0.24 to 0.41)	515.96 (400.06 to 641.5)	311.79 (241.04 to 392.18)	-1.68 (-2.03 to -1.33)
United Republic of Tanzania	G6PD trait	231 (212 to 249)	370 (337 to 399)	0.6 (0.59 to 0.61)	893.68 (819.86 to 960.59)	651.43 (594.23 to 703.22)	-1.02 (-1.17 to -0.87)
United States of America	**Inherited anemias**	1295 (1231 to 1367)	781 (761 to 800)	-0.4 (-0.43 to -0.37)	510.73 (485.32 to 539.15)	238.02 (232.03 to 243.78)	-1.85 (-2.29 to -1.42)
United States of America	Thalassemias	1 (1 to 1)	1 (1 to 1)	-0.02 (-0.11 to 0.09)	0.43 (0.32 to 0.57)	0.32 (0.26 to 0.42)	-0.65 (-0.82 to -0.48)
United States of America	Thalassemias trait	89 (76 to 103)	87 (78 to 98)	-0.02 (-0.07 to 0.04)	35.03 (29.99 to 40.8)	26.53 (23.67 to 29.74)	-0.68 (-0.82 to -0.54)

Regions	Causes	Cases in 1990 No.×103 (95% UI)	Cases in 2019 No.×103 (95% UI)	Change in absolute number (%)	Rates in 1990 per 100000 (95% UI)	Rates in 2019 per 100000 (95% UI)	EAPC from 1990 and 2019 (95%CI)
United States of America	Sickle cell disorders	44 (33 to 58)	2 (2 to 2)	-0.96 (-0.97 to -0.95)	17.25 (12.87 to 22.9)	0.52 (0.46 to 0.57)	-8.16 (-10.5 to -5.75)
United States of America	Sickle cell trait	524 (470 to 583)	117 (110 to 125)	-0.78 (-0.8 to -0.75)	206.67 (185.41 to 229.82)	35.81 (33.61 to 38.24)	-4.17 (-5.15 to -3.19)
United States of America	G6PD deficiency	212 (204 to 220)	163 (156 to 170)	-0.23 (-0.26 to -0.21)	83.66 (80.4 to 86.79)	49.78 (47.48 to 51.98)	-1.92 (-2.97 to -0.85)
United States of America	G6PD trait	425 (418 to 432)	410 (402 to 418)	-0.04 (-0.05 to -0.02)	167.7 (164.73 to 170.39)	125.07 (122.55 to 127.39)	-0.83 (-1 to -0.67)
United States Virgin Islands	**Inherited anemias**	1 (0 to 1)	0 (0 to 0)	-0.43 (-0.45 to -0.41)	532.53 (468.36 to 605.39)	311.7 (273.61 to 352.89)	-1.86 (-1.89 to -1.84)
United States Virgin Islands	Thalassemias	0 (0 to 0)	0 (0 to 0)	-0.08 (-0.45 to 0.87)	0.01 (0.01 to 0.02)	0.01 (0 to 0.01)	-0.36 (-0.6 to -0.12)
United States Virgin Islands	Thalassemias trait	0 (0 to 0)	0 (0 to 0)	-0.34 (-0.45 to -0.16)	7.28 (5.62 to 9.1)	4.9 (3.86 to 5.97)	-1.43 (-1.5 to -1.35)
United States Virgin Islands	Sickle cell disorders	0 (0 to 0)	0 (0 to 0)	-0.35 (-0.43 to -0.27)	3.7 (2.69 to 4.92)	2.43 (1.72 to 3.39)	-1.53 (-1.71 to -1.36)
United States Virgin Islands	Sickle cell trait	0 (0 to 0)	0 (0 to 0)	-0.4 (-0.44 to -0.36)	153.34 (128.96 to 179.6)	93.39 (75.9 to 112.08)	-1.75 (-1.83 to -1.66)
United States Virgin Islands	G6PD deficiency	0 (0 to 0)	0 (0 to 0)	-0.44 (-0.49 to -0.39)	167.27 (129.48 to 210.3)	95.25 (73.3 to 118.27)	-1.95 (-1.97 to -1.92)
United States Virgin Islands	G6PD trait	0 (0 to 0)	0 (0 to 0)	-0.44 (-0.46 to -0.4)	200.93 (178.92 to 224.01)	115.72 (102.98 to 129.04)	-1.91 (-1.93 to -1.9)
Uruguay	**Inherited anemias**	6 (5 to 6)	4 (4 to 5)	-0.22 (-0.25 to -0.19)	177.74 (156.31 to 201.29)	127.06 (111.76 to 143.92)	-1.25 (-1.34 to -1.15)

Regions	Causes	Cases in 1990 No.×103 (95% UI)	Cases in 2019 No.×103 (95% UI)	Change in absolute number (%)	Rates in 1990 per 100000 (95% UI)	Rates in 2019 per 100000 (95% UI)	EAPC from 1990 and 2019 (95%CI)
Uruguay	Thalassemias	0 (0 to 0)	0 (0 to 0)	-0.23 (-0.37 to 0.01)	0.23 (0.14 to 0.36)	0.16 (0.11 to 0.23)	-1.31 (-1.69 to -0.93)
Uruguay	Thalassemias trait	0 (0 to 0)	0 (0 to 0)	0.61 (0.36 to 0.94)	7 (5.73 to 8.4)	10.29 (8.74 to 12.11)	1.61 (1.17 to 2.05)
Uruguay	Sickle cell disorders	0 (0 to 0)	0 (0 to 0)	-0.12 (-0.16 to 0.32)	0 (0 to 0)	0 (0 to 0)	-0.75 (-0.82 to -0.68)
Uruguay	Sickle cell trait	0 (0 to 0)	0 (0 to 0)	-0.12 (-0.16 to 0.23)	3.24 (1.83 to 4.84)	2.59 (1.46 to 3.91)	-0.79 (-0.88 to -0.7)
Uruguay	G6PD deficiency	1 (1 to 1)	1 (1 to 1)	-0.22 (-0.26 to -0.17)	28.08 (21.75 to 34.99)	20.13 (15.59 to 25.54)	-1.3 (-1.39 to -1.22)
Uruguay	G6PD trait	4 (4 to 5)	3 (3 to 4)	-0.26 (-0.3 to -0.23)	139.2 (124.27 to 155.06)	93.89 (83.1 to 105.08)	-1.45 (-1.55 to -1.35)
Uzbekistan	**Inherited anemias**	92 (83 to 101)	92 (83 to 102)	0 (-0.01 to 0.02)	436.95 (394.14 to 483.45)	272.24 (244.98 to 301.41)	-1.21 (-1.49 to -0.92)
Uzbekistan	Thalassemias	1 (0 to 1)	0 (0 to 1)	-0.28 (-0.36 to -0.19)	2.59 (1.85 to 3.75)	1.16 (0.86 to 1.58)	-2.32 (-2.61 to -2.03)
Uzbekistan	Thalassemias trait	22 (19 to 26)	23 (20 to 26)	0.04 (-0.01 to 0.1)	106.2 (92.88 to 122.15)	68.92 (60.66 to 77.95)	-1.1 (-1.39 to -0.81)
Uzbekistan	Sickle cell disorders	0 (0 to 0)	0 (0 to 0)	0.02 (-0.13 to 0.22)	0.02 (0.01 to 0.03)	0.01 (0.01 to 0.02)	-1.13 (-1.42 to -0.84)
Uzbekistan	Sickle cell trait	3 (3 to 4)	3 (3 to 4)	0.01 (-0.08 to 0.11)	15.68 (12.39 to 19.54)	9.89 (7.81 to 12.33)	-1.16 (-1.45 to -0.88)
Uzbekistan	G6PD deficiency	10 (7 to 12)	10 (8 to 13)	0.03 (-0.03 to 0.1)	46.6 (35.69 to 59.31)	29.94 (23.04 to 37.98)	-1.04 (-1.34 to -0.74)
Uzbekistan	G6PD trait	56 (50 to 62)	55 (49 to 61)	-0.02 (-0.02 to -0.02)	265.85 (236.39 to 297.92)	162.32 (144.33 to 181.91)	-1.27 (-1.56 to -0.99)
Vanuatu	**Inherited anemias**	1 (1 to 1)	2 (2 to 2)	0.42 (0.36 to 0.49)	837.72 (770.22 to 908.99)	612.08 (543.98 to 681.91)	-1.06 (-1.17 to -0.95)

Regions	Causes	Cases in 1990 No.×103 (95% UI)	Cases in 2019 No.×103 (95% UI)	Change in absolute number (%)	Rates in 1990 per 100000 (95% UI)	Rates in 2019 per 100000 (95% UI)	EAPC from 1990 and 2019 (95%CI)
Vanuatu	Thalassemias	0 (0 to 0)	0 (0 to 0)	0.19 (0.01 to 0.37)	4.74 (2.62 to 7.24)	2.89 (1.58 to 4.3)	-0.58 (-1.52 to 0.38)
Vanuatu	Thalassemias trait	0 (0 to 0)	0 (0 to 0)	0.28 (0.2 to 0.37)	135.19 (103.75 to 164.79)	89.28 (68.81 to 107.4)	-0.99 (-1.37 to -0.6)
Vanuatu	Sickle cell disorders	0 (0 to 0)	0 (0 to 0)	0.44 (0.32 to 1.85)	0 (0 to 0.01)	0 (0 to 0)	-1.01 (-1.11 to -0.9)
Vanuatu	Sickle cell trait	0 (0 to 0)	0 (0 to 0)	0.4 (0.32 to 1.17)	5.1 (2.19 to 7.86)	3.67 (1.49 to 5.87)	-1.13 (-1.21 to -1.05)
Vanuatu	G6PD deficiency	0 (0 to 0)	0 (0 to 0)	0.34 (0.1 to 0.58)	193.29 (178.92 to 208.08)	132.78 (102.5 to 165.37)	-1.36 (-1.4 to -1.31)
Vanuatu	G6PD trait	1 (1 to 1)	1 (1 to 1)	0.49 (0.45 to 0.54)	499.39 (449.04 to 544.7)	383.46 (346.14 to 417.43)	-0.99 (-1.11 to -0.87)
Venezuela (Bolivarian Republic of)	**Inherited anemias**	117 (105 to 130)	82 (73 to 91)	-0.3 (-0.32 to -0.28)	623.71 (557.42 to 691.53)	292.36 (260.36 to 325.68)	-2.14 (-2.36 to -1.92)
Venezuela (Bolivarian Republic of)	Thalassemias	0 (0 to 0)	0 (0 to 0)	0.42 (-0.15 to 1.84)	0.01 (0.01 to 0.02)	0.01 (0.01 to 0.02)	0.25 (-0.07 to 0.57)
Venezuela (Bolivarian Republic of)	Thalassemias trait	2 (1 to 2)	2 (2 to 2)	0.02 (-0.15 to 0.31)	10.22 (7.96 to 12.78)	7.02 (5.46 to 8.57)	-0.84 (-1.05 to -0.63)
Venezuela (Bolivarian Republic of)	Sickle cell disorders	0 (0 to 0)	0 (0 to 0)	-0.59 (-0.65 to -0.52)	1.29 (1.03 to 1.58)	0.36 (0.27 to 0.45)	-4.24 (-4.54 to -3.94)
Venezuela (Bolivarian Republic of)	Sickle cell trait	17 (15 to 20)	7 (6 to 8)	-0.58 (-0.61 to -0.55)	92.5 (81.89 to 105.05)	26.24 (22.84 to 30.24)	-4.25 (-4.56 to -3.95)
Venezuela (Bolivarian Republic of)	G6PD deficiency	20 (16 to 25)	13 (10 to 16)	-0.36 (-0.39 to -0.32)	108.26 (83.15 to 135.29)	46.66 (36.05 to 58.04)	-2.37 (-2.76 to -1.97)

Regions	Causes	Cases in 1990 No.×103 (95% UI)	Cases in 2019 No.×103 (95% UI)	Change in absolute number (%)	Rates in 1990 per 100000 (95% UI)	Rates in 2019 per 100000 (95% UI)	EAPC from 1990 and 2019 (95%CI)
Venezuela (Bolivarian Republic of)	G6PD trait	77 (70 to 85)	60 (54 to 65)	-0.23 (-0.25 to -0.21)	411.43 (370.73 to 451.25)	212.07 (191.01 to 232.26)	-1.77 (-2.03 to -1.52)
Viet Nam	**Inherited anemias**	449 (409 to 490)	306 (280 to 334)	-0.32 (-0.34 to -0.3)	660.5 (602.22 to 721.6)	317.58 (290.51 to 346.36)	-2.11 (-2.32 to -1.9)
Viet Nam	Thalassemias	5 (4 to 6)	4 (3 to 4)	-0.3 (-0.37 to -0.22)	7.5 (6.24 to 9.22)	3.7 (3.15 to 4.4)	-1.99 (-2.16 to -1.83)
Viet Nam	Thalassemias trait	122 (111 to 134)	101 (93 to 111)	-0.17 (-0.2 to -0.14)	179.54 (163.98 to 197.16)	105.25 (96.12 to 115.22)	-1.41 (-1.57 to -1.25)
Viet Nam	Sickle cell disorders	0 (0 to 0)	0 (0 to 0)	-0.25 (-0.44 to -0.05)	0.02 (0.01 to 0.03)	0.01 (0.01 to 0.01)	-1.69 (-1.89 to -1.5)
Viet Nam	Sickle cell trait	9 (7 to 11)	7 (6 to 8)	-0.25 (-0.35 to -0.16)	13.38 (10.9 to 16.06)	7.04 (5.73 to 8.51)	-1.73 (-1.91 to -1.56)
Viet Nam	G6PD deficiency	56 (43 to 70)	34 (26 to 42)	-0.4 (-0.43 to -0.36)	82.84 (63.76 to 103.03)	35 (27.14 to 43.69)	-2.62 (-2.89 to -2.34)
Viet Nam	G6PD trait	256 (231 to 280)	161 (144 to 176)	-0.37 (-0.4 to -0.35)	377.23 (340.71 to 411.8)	166.58 (149.83 to 182.99)	-2.4 (-2.64 to -2.15)
Yemen	**Inherited anemias**	221 (200 to 243)	313 (283 to 345)	0.42 (0.39 to 0.44)	1609.29 (1454.34 to 1769.64)	994.97 (897.64 to 1094.65)	-1.53 (-1.77 to -1.29)
Yemen	Thalassemias	1 (0 to 1)	1 (1 to 1)	0.49 (0.33 to 0.7)	4.84 (3.2 to 7.19)	3.14 (2.17 to 4.57)	-1.43 (-1.65 to -1.2)
Yemen	Thalassemias trait	44 (36 to 53)	65 (55 to 77)	0.47 (0.39 to 0.56)	320.4 (264.93 to 386.3)	205.31 (173.14 to 244.51)	-1.44 (-1.68 to -1.21)
Yemen	Sickle cell disorders	0 (0 to 0)	0 (0 to 0)	0.4 (0.32 to 0.49)	1.59 (1.34 to 1.87)	0.97 (0.82 to 1.17)	-1.58 (-1.83 to -1.33)
Yemen	Sickle cell trait	23 (22 to 26)	33 (30 to 36)	0.4 (0.36 to 0.44)	170.78 (156.82 to 185.96)	103.84 (95.57 to 114.3)	-1.58 (-1.83 to -1.33)
Yemen	G6PD deficiency	42 (33 to 53)	59 (46 to 74)	0.39 (0.32 to 0.46)	309.42 (239.56 to 387.94)	187.22 (144.98 to 235.59)	-1.61 (-1.86 to -1.35)

Regions	Causes	Cases in 1990 No.×103 (95% UI)	Cases in 2019 No.×103 (95% UI)	Change in absolute number (%)	Rates in 1990 per 100000 (95% UI)	Rates in 2019 per 100000 (95% UI)	EAPC from 1990 and 2019 (95%CI)
Yemen	G6PD trait	110 (100 to 120)	156 (142 to 169)	0.41 (0.4 to 0.44)	802.25 (729.52 to 874)	494.49 (449.75 to 536.35)	-1.53 (-1.76 to -1.3)
Zambia	**Inherited anemias**	210 (188 to 232)	316 (284 to 349)	0.51 (0.45 to 0.56)	2637.68 (2370.15 to 2917.04)	1732.24 (1559.13 to 1914.22)	-1.47 (-1.64 to -1.3)
Zambia	Thalassemias	0 (0 to 0)	0 (0 to 0)	2.47 (2.04 to 3.08)	0.1 (0.06 to 0.16)	0.15 (0.09 to 0.23)	1.09 (0.86 to 1.32)
Zambia	Thalassemias trait	2 (2 to 3)	5 (4 to 6)	1.17 (1.02 to 1.36)	29.01 (22.77 to 35.5)	27.38 (21.75 to 33.29)	-0.38 (-0.53 to -0.23)
Zambia	Sickle cell disorders	4 (3 to 5)	5 (4 to 6)	0.28 (0.08 to 0.5)	50.15 (38.96 to 66.63)	28.05 (22.65 to 35.11)	-1.83 (-2.28 to -1.38)
Zambia	Sickle cell trait	70 (62 to 80)	100 (91 to 111)	0.43 (0.32 to 0.54)	881.5 (785.04 to 1005.69)	549.76 (498.96 to 610.93)	-1.58 (-1.84 to -1.32)
Zambia	G6PD deficiency	57 (44 to 71)	86 (67 to 106)	0.51 (0.39 to 0.62)	719.65 (558.35 to 893.7)	472.22 (365.39 to 583.62)	-1.51 (-1.65 to -1.36)
Zambia	G6PD trait	76 (70 to 81)	119 (110 to 128)	0.57 (0.52 to 0.62)	957.27 (880.26 to 1025.9)	654.67 (603.12 to 701.92)	-1.36 (-1.48 to -1.25)
Zimbabwe	**Inherited anemias**	125 (111 to 140)	138 (122 to 154)	0.1 (0.07 to 0.12)	1210.48 (1071.29 to 1356.02)	917.68 (813.25 to 1023.71)	-0.67 (-0.81 to -0.53)
Zimbabwe	Thalassemias	0 (0 to 0)	0 (0 to 0)	1.43 (1.04 to 1.97)	0.08 (0.05 to 0.12)	0.14 (0.08 to 0.21)	1.81 (1.64 to 1.98)
Zimbabwe	Thalassemias trait	3 (3 to 4)	4 (4 to 5)	0.48 (0.35 to 0.64)	29.12 (24.46 to 34.81)	29.72 (24.26 to 35.38)	0.29 (0.17 to 0.41)
Zimbabwe	Sickle cell disorders	0 (0 to 0)	0 (0 to 0)	-0.08 (-0.19 to 0.02)	1.69 (1.3 to 2.24)	1.07 (0.8 to 1.42)	-1.9 (-2.1 to -1.7)
Zimbabwe	Sickle cell trait	16 (14 to 18)	16 (14 to 19)	0.03 (-0.04 to 0.08)	153.73 (134.56 to 177.18)	108.56 (94.28 to 125.73)	-1.21 (-1.31 to -1.12)
Zimbabwe	G6PD deficiency	36 (28 to 46)	39 (30 to 49)	0.06 (0.03 to 0.1)	352.6 (271.28 to 441.64)	258.58 (198.98 to 324.49)	-0.75 (-0.94 to -0.56)

Regions	Causes	Cases in 1990 No.×103 (95% UI)	Cases in 2019 No.×103 (95% UI)	Change in absolute number (%)	Rates in 1990 per 100000 (95% UI)	Rates in 2019 per 100000 (95% UI)	EAPC from 1990 and 2019 (95%CI)
Zimbabwe	G6PD trait	70 (64 to 75)	78 (71 to 84)	0.12 (0.09 to 0.15)	673.26 (615.69 to 728.09)	519.61 (473.24 to 561.3)	-0.56 (-0.7 to -0.42)

Abbreviations: EAPC, estimated annual percentage change; UI, uncertainty interval. CI, confidence interval.
